# One-Pot (3 + 2) Cycloaddition–Isomerization–Oxidation of 2,2,2-Trifluorodiazoethane and Styryl Derivatives

**DOI:** 10.1021/acs.joc.3c00396

**Published:** 2023-07-21

**Authors:** Julia Altarejos, Estíbaliz Merino, David Sucunza, Juan J. Vaquero, Javier Carreras

**Affiliations:** † Universidad de Alcalá, Departamento de Química Orgánica y Química Inorgánica, Instituto de Investigación Química “Andrés M. del Río” (IQAR), 28805, Alcala de Henares, Madrid, Spain; ‡ Instituto Ramón y Cajal de Investigación Sanitaria (IRYCIS) 28034, Madrid, Spain

## Abstract

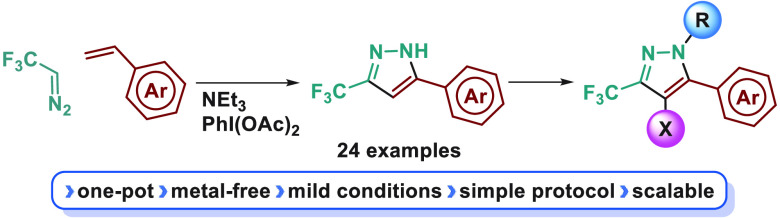

A facile access to 5-aryl-3-trifluoromethylpyrazoles has been developed by a one-pot (3 + 2) cycloaddition–isomerization–oxidation sequence employing 2,2,2-trifluorodiazoethane and styryl derivatives. A broad variety of functional groups and good yields are achieved under mild conditions. Additionally, the functionalization of 3-trifluoromethylpyrazoles was studied. DFT calculations of the cycloaddition transition state energies are consistent with the experimentally observed reactivity.

Pyrazole is a common heterocycle in bioactive compounds.^1^ Among them, fluorinated pyrazoles^2^ are interesting scaffolds due to the adjustments of physicochemical properties produced by the presence of C–F bonds. In the past decade, the number of reports related to trifluoromethyl derivatives has increased significantly,^2^ with applications in pharmaceuticals, agrochemicals, or ligands for transition metals. Particularly, 5-aryl-3-trifluoromethylpyrazoles have successfully led to marketed drugs such as Mavacoxib^3a^ (veterinary) or Celecoxib^3b^ (anti-inflammatory) and related structures have been investigated.^3c,3d^ This scaffold also exhibited herbicide activity,^2^ and it has been recently applied in coordination chemistry^4^ (Figure 1).

**Figure 1:**
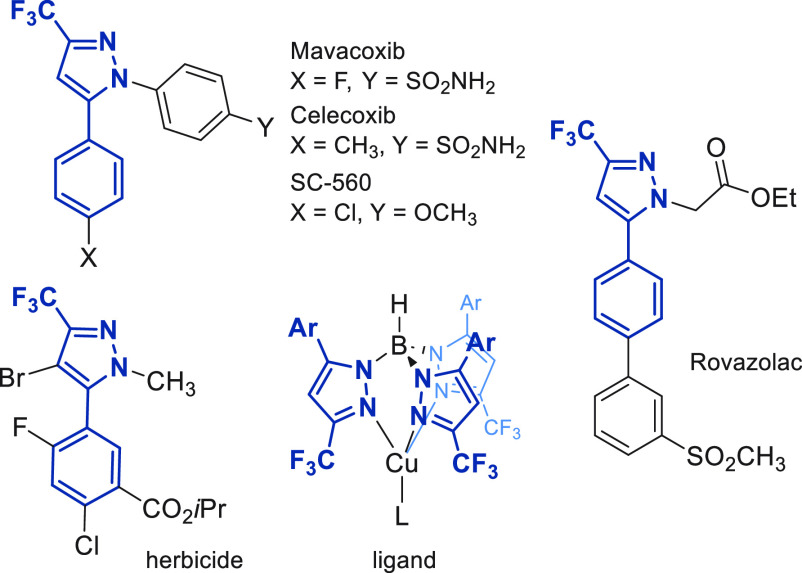
Applications of some 5-aryl-3-trifluoromethylpyrazoles.

Accordingly, various methodologies have reported the preparation of 5-aryl-3-trifluoromethylpyrazoles. The main strategies involved the condensation of 1,3-dicarbonyl compounds (or equivalents) with hydrazine,^5^ and (3 + 2) cycloaddition reactions, also known as 1,3-dipolar cycloadditions.^6−10^ In the latter strategy, 2,2,2-trifluorodiazoethane^7^ (CF_3_CHN_2_) is certainly a suitable reagent to prepare trifluoromethylpyrazoles by cycloaddition. The reactivity toward alkynes has been deeply studied over the past few years, employing silver oxide as an activator,^8a^ DBU,^8b^ or flow chemistry conditions^8c,8d^ (Scheme 1a). Recently, a *N*-triftosylhydrazone derivative^8e^ has been reported to form the diazo compound *in situ*. Noteworthy, equimolecular amounts of metal or relatively high temperatures (80–100 °C) were needed to proceed with the cycloaddition.

**Scheme 1:**
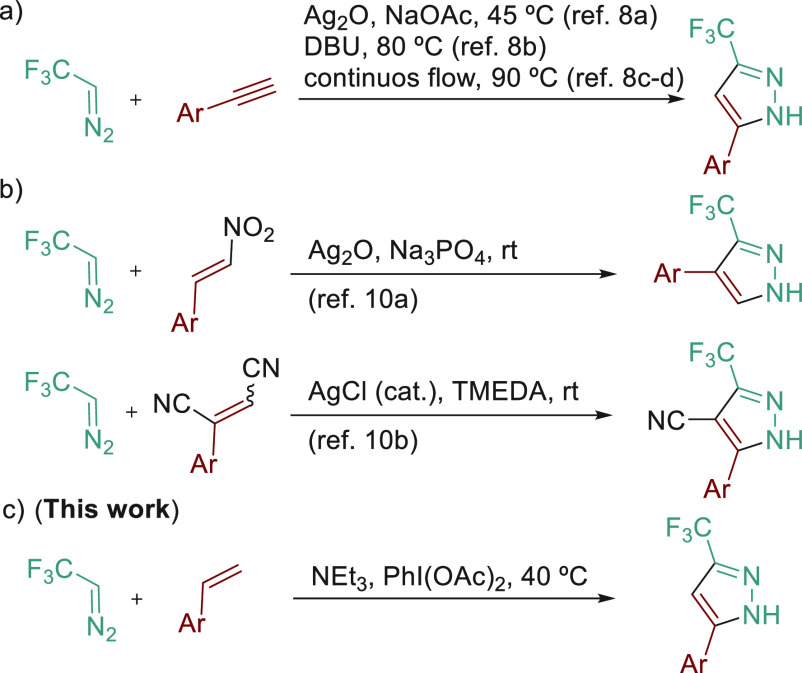
Reported (3 + 2) Cycloaddition Reactions with 2,2,2-Trifluorodiazoethane

Another possibility is the (3 + 2) cycloaddition of trifluorodiazoethane with alkenes to give pyrazolines, which can then be transformed into the corresponding pyrazoles with an additional step. The pioneering work on this reaction by Atherton and Fields revealed a limitation in the scope of the reaction: only electron-deficient alkenes smoothly react with pure 2,2,2-trifluorodiazoethane.^9a−9c^ Later, the cycloadditions of *in situ* generated CF_3_CHN_2_ with electron-deficient alkenes were studied by Mykhailiuk.^9d^ More recently, Ma’s group has also addressed the silver-catalyzed cycloaddition of trifluorodiazoethane with activated alkenes to yield aryl trifluoromethyl pyrazoles after *in situ* elimination of an electron-withdrawing group (−NO_2_ or −CN)^10^ (Scheme 1b).

Despite the interest in 5-aryl-3-trifluoromethylpyrazoles, there are no examples of cycloaddition reactions of trifluorodiazoethane with simple styrene derivatives without an additional electron-withdrawing group in the alkene. The reactivity described between these two reagents is limited to metal- or enzyme-catalyzed cyclopropanation reactions^11^ and one example of a photocatalyzed hydroalkylation reaction.^12^ In this context, we have focused our attention on the development of the cycloaddition of trifluorodiazoethane with styrene derivatives and *in situ* transformation to 5-aryl-3-trifluoromethylpyrazoles (Scheme 1c).

Our studies commenced by mixing 2,2,2-trifluorodiazoethane (**1**) in DCE solution with styrene (**2a**) as a benchmark reaction. The use of 2 equiv of styrene led to a *cis*–*trans* mixture (ca. 1:1) of 1-pyrazoline (**3a**) in low yield (Table 1, entry 1). Initial experiments showed that the higher the amount of styrene equivalents, the better the yields that were obtained (entries 1–3). We tested different solvent mixtures, such as toluene, decane, acetonitrile, THF, or DCM, and DCE was identified as the optimal solvent for this reaction.^13^ Furthermore, the increase of the temperature from 25 to 40 °C allowed the reduction of styrene equivalents with similar good yields (entries 3–4). Next, we investigated the effect of the reaction concentration, observing an improvement in yield with a higher concentration (entry 5). The use of an excess of trifluorodiazoethane produced unidentifiable byproducts resulting in lower yields of the pyrazoline **3a** (entry 6).

**Table 1:**
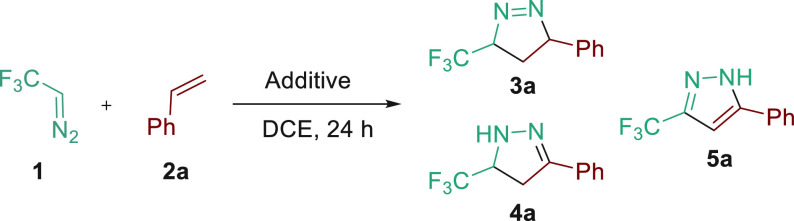
Reaction Optimization^a^

entry	*T*^a^	styrene (equiv)	conc [M]	additive	product	yield (%)^b^
1	25	2	0.5	–	**3a**	10
2	25	5	0.5	–	**3a**	64
3	25	10	0.5	–	**3a**	72
4	40	5	0.5	–	**3a**	76
5	40	5	0.75	–	**3a**	87
6	40	0.5	0.75	–	**3a**	52
7	40	5	0.75	NEt_3_^c^	**4a**	86
8	40	5	0.75	NEt_3_,^c^ PhI(OAc)_2_^d^	**5a**	79(63^e^)
9	40	5	0.75	PhI(OAc)_2_^d^	**4a**	8
10	40	5^f^	0.75	–	**5a**	27

a Reaction conditions: **1** (0.85 mmol), styrene (**2a**), DCE, 24 h.

b NMR yields were calculated by ^19^F NMR integration with trifluorotoluene as an internal standard.

c NEt_3_ (2.5 equiv).

d PhI(OAc)_2_ (1.5 equiv).

e Isolated yield.

f Equivalents of phenylacetylene.

Noteworthy, we noticed pyrazoline **3a** partially isomerized to 2-pyrazoline **4a** in the NMR tube in CDCl_3_. After screening several acidic and basic additives,^13^ we observed the selective formation of compound **4a** by treatment with NEt_3_. This cycloaddition–isomerization sequence could be performed in one-pot in good yield (Table 1, entry 7). We then focused on the direct synthesis of pyrazole **5a**, promoted by oxidants such as MnO_2_, DDQ, or halogen-based oxidations (I_2_, Br_2_, NBS, and NIS), but gave incomplete conversion. Gratifyingly, PhI(OAc)_2_ smoothly led to pyrazole **5a**, and the employment of NEt_3_ and PhI(OAc)_2_ as additives allowed the one-pot formation of pyrazole **5a** in 63% isolated yield after three chemical steps (entry 8). The addition only of the oxidant led to a low yield of 2-pyrazoline **4a** (entry 9). Reaction with phenylacetylene instead of styrene was also tested under similar conditions (entry 10), giving a low conversion to pyrazole **5a**.

With these optimized reaction conditions for the synthesis of pyrazoles, we moved on to explore the reaction substrate scope (Scheme 2). A wide range of substituted styrenes (alkyl, halogens, nitro, trifluoromethyl, ether, thioether, ciano, ester, and boronate substituents) were compatible with the reaction and afforded the 5-aryl-3-trifluoromethylpyrazoles products in moderate to good yields (50–75%). Furthermore, one *ortho* substituent did not display a negative effect on the product yield (**5b**, **5c**, **5t**), although a mesityl ring suppressed the reactivity (**5u**). For the highly deactivated substrates (**5f**, **5v**), a mixture of 2-pyrazoline and pyrazole was obtained under standard conditions, and it was necessary to increase the PhI(OAc)_2_ equivalents and temperature to achieve complete conversion to pyrazole. The reaction also exhibits tolerance to heterocycles such as pyridine (**5x**) or thiophene (**5y**), the latter obtaining the product in low yield. We have also examined *tert*-butyl acrylate, as a withdrawing group substituent in the olefin under our standard conditions, affording the pyrazole in 47% isolated yield (**5z**). No reaction was observed in the presence of aliphatic alkenes (1-hexene) or disubstituted olefins (stilbene or α-methylstyrene). In addition, a scale-up experiment (10 mmol) was performed under optimized conditions for styrene, and a 59% isolated yield was obtained. Notably, the *in situ* formation of the diazo compound from CF_3_CH_2_NH_2_·HCl^9d^ and NaNO_2_ also provides a practical result, and these conditions could be extended to the pentafluoroethyl group^14^ (**5aa**).

**Scheme 2:**
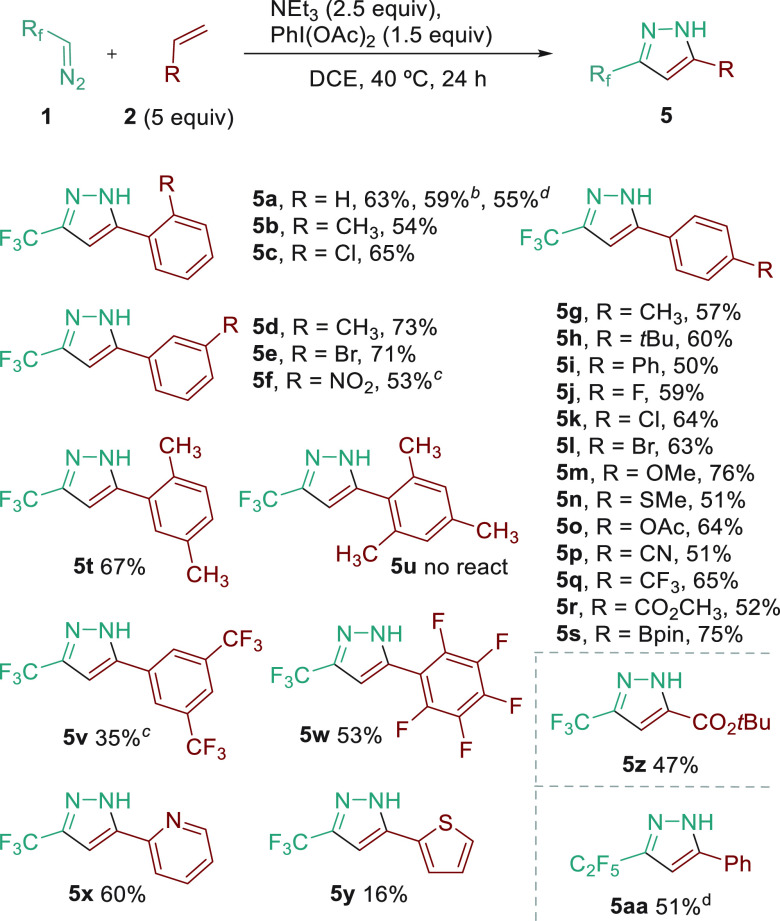
Substrate Scope of Pyrazole Synthesis Reaction conditions: **1** (0.85 mmol), **2** (5 equiv), NEt_3_ (2.5 equiv), PhI(OAc)_2_ (1.5 equiv), DCE (0.75 M), 40 °C, 24 h. Isolated yields. 10 mmol scale. PhI(OAc)_2_ (4 equiv), 60 °C. R_f_CH_2_NH_2_·HCl (0.85 mmol), NaNO_2_ (1.0 mmol), **2** (5 equiv), DCE/H_2_O (9:1, 0.75 M), 40 °C, 24 h; NEt_3_ (2.5 equiv), PhI(OAc)_2_ (1.5 equiv), 40 °C, 24 h.

Upon investigation of the scope of this reaction, we were interested in exploring the derivatization of these compounds. The *N*-arylation of 5-aryl-3-trifluoromethylpyrazole gives access to the synthesis of different drugs and pharmaceutical candidates (Figure 1) and has been previously reported.^8^ Therefore, we focused our attention on other functionalizations in the pyrazole. Bromination at the 4-position could be achieved with NBS as reagent in good yield (**6**, Scheme 3). Some 5-aryl-4-bromo-3-trifluoromethylpyrazole derivatives have recently showed considerable postemergent activity on weeds.^15^ In addition, *N*-alkylation with ethyl 2-bromoacetate in basic medium was carried out to obtain **7** in moderate yield, a substructure present in Rovazolac (Figure 1).

**Scheme 3:**
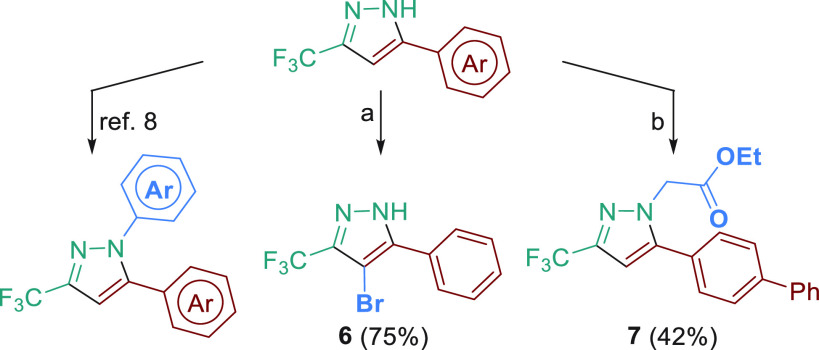
Derivatization of 5-Aryl-3-trifluoromethylpyrazolea Reaction conditions: (a) **5a**, NBS (1 equiv), CH_2_Cl_2_, 40 °C, 12 h; (b) **5i**, K_2_CO_3_ (3 equiv), BrCH_2_CO_2_Et (1 equiv), CH_3_COCH_3_, reflux, 12 h (isomer **7′**, 37%).

To gain insight into the undescribed cycloaddition of trifluorodiazoethane with styrene derivatives, we performed DFT calculations to compare the transition state energies of the 1,3-dipolar cycloadditions between unsaturated systems and diazo compounds (2,2,2,-trifluorodiazoethane and ethyl diazoacetate). These results are in agreement with the experimental findings, with an increasing trend from the reaction of trifluorodiazoethane with ethyl acrylate, styrene, or hexene (Figure 2a–c). Moreover, the different energy values allowed us to justify the complete regioselectivity found in these reactions, as well as the lack of stereoselectivity.^13^ The transition state energy for the reaction of trifluorodiazoethane with phenylacetylene shows a difference of ∼3 kcal/mol compared with styrene, which explains the low reactivity under these reaction conditions (Figure 2d). Finally, a similar comparison was performed using ethyl diazoacetate. A significant energy difference can be found between the reactions with ethyl acrylate or styrene (Figure 2e–f).

**Figure 2:**
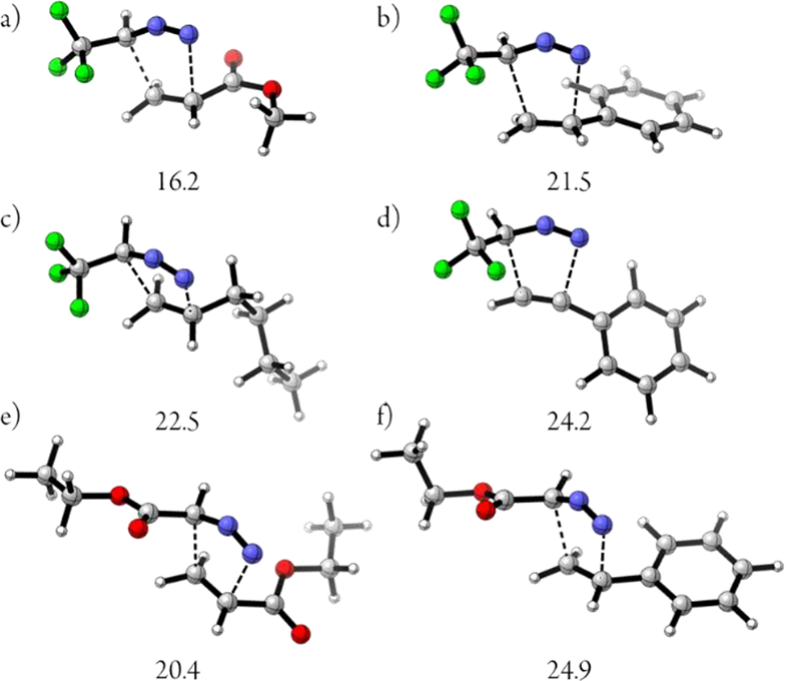
Energy of the transition states (*exo* adducts) calculated with M062X/6-311++g(d,p). Activation free energies are given in kcal/mol.

In summary, a one-pot three-step (3 + 2) cycloaddition–isomerization–oxidation sequence has been developed for the coupling of 2,2,2-trifluorodiazoethane and styrene derivatives to access 5-aryl-3-trifluoromethylpyrazoles. This protocol is metal-free, operationally simple, and scalable; features mild conditions; and has a broad substrate scope. Subsequent functionalization of 3-trifluoromethylpyrazoles, including bromination at position 4 or *N*-alkylation has been explored. Computational data of the transition state energies of the (3 + 2) cycloaddition justify the accessibility to the pyrazolines in the first step of our one-pot procedure.

## Data Availability

The data underlying this study are available in the published article and its Supporting Information.

## Supporting Information Available

Experimental procedures, characterization data, ^1^H, ^13^C and ^19^F NMR spectral data, mass spectrometry data and computational information (PDF)
