# Supplementary material for: One-Pot (3 + 2) Cycloaddition–Isomerization–Oxidation of 2,2,2-Trifluorodiazoethane and Styryl Derivatives
Source: J Org Chem. 2023 Jul 21;88(15):11258–62. doi: 10.1021/acs.joc.3c00396 (PMC10407847; doi:10.1021/acs.joc.3c00396)
Supplement: Supplementary file 1 — jo3c00396_si_001.pdf [file jo3c00396_si_001.pdf]

Supporting Information

One-Pot (3+2) Cycloaddition-Isomerization-Oxidation of 2,2,2-Trifluorodiazoethane and Styryl Derivatives

Julia Altarejos, Estíbaliz Merino, David Sucunza, Juan J. Vaquero, Javier Carreras*

Universidad de Alcalá, Departamento de Química Orgánica y Química Inorgánica, Instituto de Investigación Química "Andrés M. del Río" (IQAR), 28805, Alcalá de Henares, Madrid, Spain; Instituto Ramón y Cajal de Investigación Sanitaria (IRYCIS) 28034, Madrid, Spain.

E-mail: javier.carreras@uah.es

Table of Contents

Experimental procedures S-3

Optimization details S-4

General procedure pyrazole synthesis S-6

Unsuccessful reactions S-6

Characterization data S-7

NMR spectra S-19

DFT studies S-87

Experimental procedures

All commercially available compounds were used as received. Reactions were carried out under nitrogen atmosphere with standard schlenck techniques. Solvents were purchased from commercial sources. Analytical thin layer chromatography was carried out using TLC-aluminium sheets with 0.2 mm of silica gel (Merck 60 F254) and UV light as visualizing agent or phosphomolybdic acid solution as developing agent. Chromatography purifications were carried out using silica gel (40-63 µm, 60 Å).

NMR spectra were recorded at 298 K using either a Varian Mercury VX-300, Bruker Avance NEO 400, or Varian Unity 500 MHz spectrometer. Chemical shift values for ^{1}H and ^{13}C are reported as δ values (ppm) relative to the deuterated solvent ($CDCl_3$: 7.26 ppm, 77.16 ppm) and coupling constants (*J*) in Hz. The following abbreviations are used in reporting NMR data: s, singlet; bs, broad singlet; d, doublet; t, triplet; q, quartet; m, multiplet.

Melting points were determined in open capillary tubes using a Stuart Scientific SMP3 melting point apparatus. High-resolution analysis (HRMS) were performed using an Agilent 6210 TOF LC/MS system.

Caution: 2,2,2-Trifluorodiazoethane should be stored and handled with care due to its toxicity, explosion risk, and low boiling point (gaseous at room temperature).

Optimization details

Additional experiments in the optimization of the synthesis of pyrazoles.

Table S1. Optimization of the (3+2) cyclization reaction using trifluorodiazoethane (**1**) and styrene (**2a**).

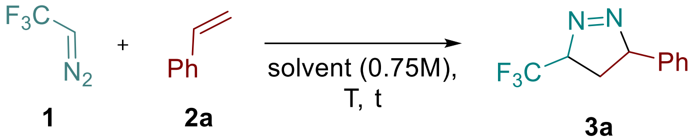

1 **2a** **3a**

	temp (ºC)	1 (mmol)	2a (eq.)	solvent	t (h)	yield[a]
**1**	25	0.85	**2**	DCE	24	10
**2**	**25**	0.85	**5**	DCE	24	64
**3**	25	0.85	**10**	DCE	24	72
**4**	**40**	0.85	5	DCE	**24**	76
**5**	40	**2 eq.**	(0.5 mmol)	DCE	24	52
**6**	40	0.85	5	DCE	**6h slow addition**	0
**7**	40	0.85	5	DCE	**5**	14
**8**	40	0.85	5	DCE	**16**	45
**9**	40	0.85	5	**THF**/DCE	24	30
**10**	40	0.85	5	**CH_3CN**/DCE	24	24
**11**	40	0.85	5	**Toluene**/DCE	24	34
**12**	40	0.85	5	**Decane**/DCE	24	28

[a] NMR yields were calculated by ^{19}F NMR integration with trifluorotoluene as an internal standard

Table S2. Optimization of the isomerization-oxidation of the pyrazoline **3a**.

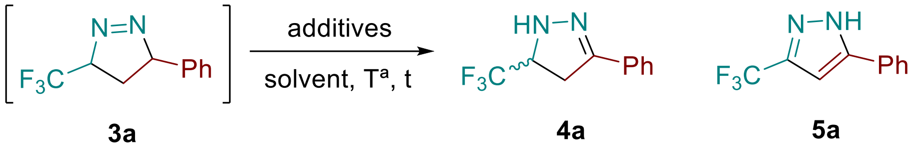

	additive	solvent	t (h)	temp (ºC)	product	yield[a]
**1**	TFA	DCM	16	rt	**4a**	15
**2**	DABCO	DCM	48	rt	Decomposition	-
**3**	AcOH	DCM	12	rt	s.m.	0
**4**	AcOH, O_2	Dioxane	32	70	**4a**	10
**5**	MnO_2	Toluene	16	rt	s.m.	0
**6**	^{t}BuONO, MnO_2	toluene	77	rt to reflux	Decomposition	-
**7**	DDQ	Toluene	16	60	s.m.	0
**8**	DDQ	DCM	16	40	s.m.	0
**9**	Et_3N	DCE	16	40	**4a**	86
**10**	NBS, Et_3N	DCE	72	60	**5a**	70
**11**	I_2, Et_3N	DCE	4	100	**5a**	51
**12**	I_2, Et_3N	DCE	24	80	**5a**	55
**13**	Br_2, Et_3N	DCE	4	rt	Decomposition	-
**14**	$PhIOAc_2$, Et_3N	DCE	24	40	**5a**	79
**15**	$PhIOAc_2$	DCE	24	40	**3a**+**4a** (1:0.3)	31

[a] NMR yields were calculated by ^{19}F NMR integration with trifluorotoluene as an internal standard

General procedure pyrazole synthesis

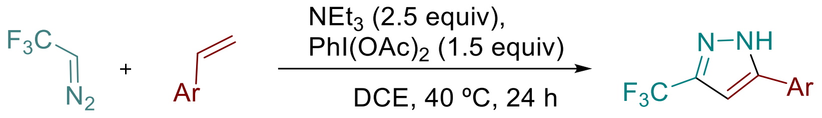

1 **2** (5 equiv) **5**

Trifluorodiazoethane (0.75-1.0 M in DCE, 0.85 mmol) was added to a solution of styrene (5 equiv.), Et_3N (2.5 equiv.) and iodobenzene diacetate (1.5 equiv.) in dry DCE (0.75 M, final concentration). The reaction was stirred at 40 °C in a sand bath for 24 hours. The crude was evaporated and purified by column chromatography (hexane/diethyl ether 6:4) to get the corresponding pyrazole.

Unsuccessful reactions

Following scheme outline the reactions that gave little or no product:

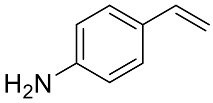

no reaction

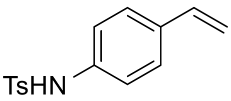

no reaction

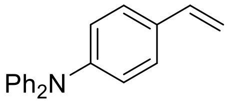

complex mixture

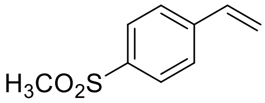

complex mixture

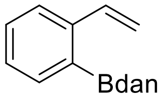

complex mixture

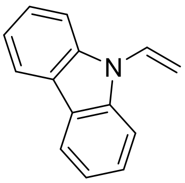

no reaction

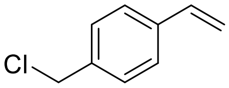

complex mixture

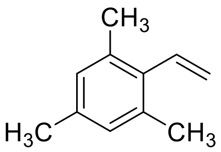

no reaction

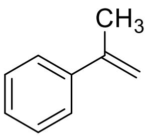

no reaction

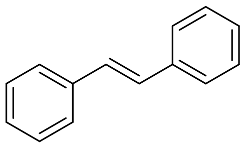

complex mixture

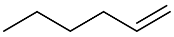

complex mixture

Characterization data

2,2,2-Trifluorodiazoethane (1)

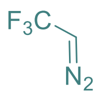

2,2,2-Trifluorodiazoethane was prepared following a described methodology.[S1] DCE solution was stored in the fridged under inert atmosphere. NMR data were in agreement with those reported.
^{1}H NMR (400 MHz, $CDCl_3$) δ 4.45 (q, *J* = 4.1 Hz, 1H).
^{19}F NMR (376 MHz, $CDCl_3$) δ -55.1 (d, *J* = 4.2 Hz).

Methyl(4-vinylphenyl)sulfane (2n)

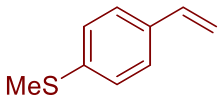

Alkene **2n** was prepared following a described methodology. [S2] NMR data were in agreement with those reported.
^{1}H NMR (300 MHz, $CDCl_3$) δ 7.40 (d, *J* = 8.3 Hz, 1H), 7.27 (d, *J* = 8.3 Hz, 2H), 6.75 (dd, *J* = 17.6, 10.9 Hz, 1H), 5.80 (dd, *J* = 17.6, 0.9 Hz, 1H), 5.30 (dd, *J* = 10.9, 0.9 Hz, 1H), 2.52 (s, 3H).

Methyl 4-vinylbenzoate (2r)

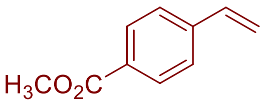

Alkene **2r** was prepared following a described methodology. [S3] NMR data were in agreement with those reported.
^{1}H NMR (300 MHz, $CDCl_3$) δ 7.88 (d, *J* = 8.3 Hz, 2H), 7.31 (d, *J* = 8.3 Hz, 2H), 6.60 (dd, *J* = 17.6, 10.9 Hz, 1H), 5.73 (d, *J* = 17.6 Hz, 1H), 5.24 (d, *J* = 10.9 Hz, 1H), 3.77 (s, 3H) ppm.

4,4,5,5-Tetramethyl-2-(4-vinylphenyl)-1,3,2-dioxaborolane (2s)

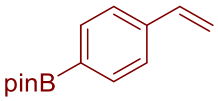

Alkene **2s** was prepared following a described methodology. [S4]
^{1}H NMR (300 MHz, $CDCl_3$) δ 7.79 (d, *J* = 8.1 Hz, 2H), 7.42 (d, *J* = 8.0 Hz, 2H), 6.74 (dd, *J* = 17.6, 10.9 Hz, 1H), 5.83 (d, *J* = 17.6 Hz, 1H), 5.30 (d, *J* = 10.9 Hz, 1H), 1.36 (s, 12H) ppm.

4-Methyl-*N*-(4-vinylphenyl)benzenesulfonamide (S2a)

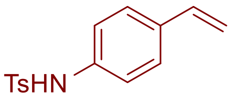

Alkene **S2a** was prepared following a described methodology. [S5] NMR data were in agreement with those reported.
^{1}H NMR (300 MHz, $CDCl_3$) δ 7.67 (d, *J* = 8.2 Hz, 2H), 7.27 (d, *J* = 8.1 Hz, 2H), 7.22 (d, *J* = 8.2 Hz, 2H), 7.03 (d, *J* = 8.1 Hz, 2H), 6.83 (s, 1H), 6.62 (dd, *J* = 17.6, 10.9 Hz, 1H), 5.65 (d, *J* = 17.6 Hz, 1H), 5.20 (d, *J* = 10.9 Hz, 1H), 2.37 (s, 3H).

[S1] Wang, S.; Yang, L.-J.; Zeng, J.-L.; Zheng, Y.; Ma, J.-A. *Org. Chem. Front.* **2015**, *2*, 1468–1474.
[S2] Peng, W.; Sun, Z.-Y.; Zhang, Q.; Cheng, S.-Q.; Wang, S.-K.; Wang, X.-N.; Kuang, G.-T.; Su, X.-X.; Tan, J.-H.; Huang, Z.-S.; Ou, T.-M. *J. Med. Chem.* **2018**, *61*, 6629–6646.
[S3] Movahhed, S.; Westphal, J.; Dindaroğlu, M.; Falk, A.; Schmalz, H. *Chem. Eur. J.* **2016**, *22*, 7381–7384.
[S4] Aukland, M. H.; Talbot, F. J. T.; Fernández-Salas, J. A.; Ball, M.; Pulis, A. P.; Procter, D. J. *Angew. Chem. Int. Ed.* **2018**, *57*, 9785–9789.
[S5] Molle, E.; Mutlu, H.; Theato, P. *Macromol. Rapid Commun.* **2021**, *42*, 2100063.

***N,N*-Diphenyl-4-vinylaniline (S2b)**

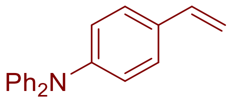

Alkene **S2b** was prepared following a described methodology.[S6] NMR data were in agreement with those reported.

^{1}H NMR (300 MHz, $CDCl_3$) δ 7.41 – 7.24 (m, 6H), 7.18 – 6.97 (m, 8H), 6.69 (dd, *J* = 17.6, 10.9 Hz, 1H), 5.67 (d, *J* = 17.6 Hz, 1H), 5.19 (d, *J* = 10.9 Hz, 1H).

1-(Methylsulfonyl)-4-vinylbenzene (S2c)

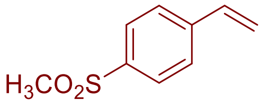

Alkene **S2c** was prepared following a described methodology.[S7] NMR data were in agreement with those reported.

^{1}H NMR (300 MHz, $CDCl_3$) δ 7.89 (d, *J* = 8.4 Hz, 2H), 7.57 (d, *J* = 8.3 Hz, 2H), 6.76 (dd, *J* = 17.6, 10.9 Hz, 1H), 5.91 (d, *J* = 17.6 Hz, 1H), 5.46 (d, *J* = 10.9 Hz, 1H), 3.05 (s, 3H).

2-(2-Vinylphenyl)-2,3-dihydro-1H-naphtho[1,8-de][1,3,2]diazaborinine (S2d)

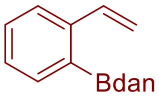

Alkene **S2d** was prepared following a described procedure.[S8]
(2-Vinylphenyl)boronic acid (1,07 g, 7.21 mmol, 1.2 equiv) and naphthalene-1,8-diamine (0.95g, 6.00 mmol) were suspended in water (15 mL) at 60 ºC in a sand bath for 18 hours. The crude was extracted with AcOEt (3x10 mL). The organic layer was dried over anhydrous Na_2SO_4, filtered, and evaporated. The crude was purified by flash columnchromatography (hexane/AcOEt, 15:1) to obtain the alkene as an off-white solid (1.39 g, 86%).

^{1}H NMR (400 MHz, $CDCl_3$) δ 7.62 (d, *J* = 7.8 Hz, 1H), 7.53 (d, *J* = 7.3 Hz, 1H), 7.44 (t, *J* = 7.6 Hz, 1H), 7.34 (t, *J* = 7.3 Hz, 1H), 7.22 – 7.12 (m, 2H), 7.13 – 7.02 (m, 3H), 6.36 (d, *J* = 7.2 Hz, 2H), 5.86 (s, 2H), 5.77 (d, *J* = 17.4 Hz, 1H), 5.34 (d, *J* = 10.9 Hz, 1H).

^{13}C NMR (101 MHz, $CDCl_3$) δ 141.4, 141.1, 137.8, 136.5, 132.6, 129.7, 127.7, 127.5, 125.4, 119.9, 118.0, 115.8, 106.1 ppm.

^{11}B NMR (128 MHz, $CDCl_3$) δ 30.4 ppm.

M.p. 95 – 97 °C; **HRMS-ESI** m/z calcd for $C_{18}H_{15}BN_2$ $[M]^+$ 270.1323, found 270.1329.

[S6] Wang, G.-Z.; Shang, R.; Fu, Y. *Org. Lett.* **2018**, *20*, 888–891.
[S7] Scheidt, F.; Neufeld, J.; Schäfer, M.; Thiehoff, C.; Gilmour, R. *Org. Lett.* **2018**, *20*, 8073–8076.
[S8] Liao, S.; Hu, X.; Li, Y.; Wang, X.; Li, D.; Wang, Q.; Wang, Y.; Huang, X.; Xu, P.; Wu, H.; Li, X.; Yuan, J. *Tetrahedron* **2021**, *90*, 132205.

5-Phenyl-3-(trifluoromethyl)-4,5-dihydro-3H-pyrazole (3a)

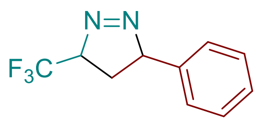

A mixture of trifluorodiazoethane (0.85 ml, 1.0 M) and styrene (0.50 mL, 4.25 mmol) in DCE (1.1 mL, total volume) was stirred at 40 °C in a sand bath for 24 hours. Solvent was evaporated for characterization. The compound partially isomerizes to 2-pyrazoline **4a** in chloroform solution. The NMR characterization was done with the mixture *cis*-*trans* of pyrazoline **3a**. Signals were assigned with bidimensional NMR experiments (COSY, HSQC) and coupling constants values.

^{1}H NMR (500 MHz, $CDCl_3$) δ 7.46 – 7.33 (m, 6H), 7.31 – 7.24 (m, 2H), 7.23 – 7.10 (m, 2H), 5.87 (ddd, *J* = 9.3, 6.2, 2.4 Hz, 1H, *trans*), 5.43 (ddd, *J* = 10.0, 8.7, 2.9 Hz, 1H, *cis*), 5.40 – 5.33 (m, 1H, *trans*), 5.00 – 4.82 (m, 1H, *cis*), 2.56 (dt, *J* = 13.2, 8.7 Hz, 1H, *cis*), 2.28 (ddd, *J* = 13.7, 9.3, 5.9 Hz, 1H, *trans*), 1.89 (ddd, *J* = 13.7, 9.9, 6.2 Hz, 1H, *trans*), 1.57 (dt, *J* = 13.2, 10.0 Hz, 1H, *cis*) ppm.
^{13}C{^{1}H} NMR (126 MHz, $CDCl_3$) δ 137.5, 137.2, 129.3, 129.2, 128.60, 128.56, 127.4, 127.0, 124.1 (q, *J* = 278.4 Hz), 124.0 (q, *J* = 279.4 Hz), 93.3, 93.0, 89.0 (q, *J* = 27.2 Hz), 88.9 (q, *J* = 27.8 Hz), 27.0 (q, *J* = 1.6 Hz), 26.0 (q, *J* = 1.5 Hz) ppm.
^{19}F NMR (376 MHz, $CDCl_3$) δ -70.0 (d, *J* = 8.0 Hz, *cis*), -70.2 (d, *J* = 8.2 Hz, *trans*) ppm.

5-Phenyl-3-(trifluoromethyl)-4,5-dihydro-1H-pyrazole (4a)

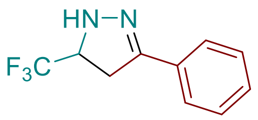

A mixture of trifluorodiazoethane (0.85 ml, 1.0 M), styrene (0.50 mL, 4.25 mmol) and Et_3N (0.30 mL, 2.13 mmol) in DCE (1.1 mL, total volume) was stirred at 40 °C in a sand bath for 24 hours. Solvent was evaporated for characterization. The compound decomposed upon standing or in solution.

^{1}H NMR (400 MHz, $CDCl_3$) δ 7.72 – 7.58 (m, 2H), 7.48 – 7.34 (m, 3H), 6.00 (bs, 1H), 4.43 – 4.27 (m, 1H), 3.36 (dd, *J* = 17.0, 11.5 Hz, 1H), 3.23 (dd, *J* = 17.0, 8.2 Hz, 1H) ppm.
^{13}C{^{1}H} NMR (101 MHz, $CDCl_3$) δ 151.6, 131.7, 129.6, 128.8, 126.3, 125.4 (q, *J* = 279.0 Hz), 60.4 (q, *J* = 30.6 Hz), 33.7 (q, *J* = 2.1 Hz) ppm.
^{19}F NMR (376 MHz, $CDCl_3$) δ -76.7 (d, *J* = 7.3 Hz) ppm.

5-Phenyl-3-(trifluoromethyl)-1*H*-pyrazole (5a)

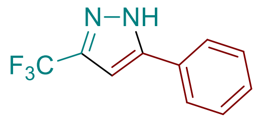

Following the general procedure a mixture of trifluorodiazoethane (0.71 mL, 1.2 M), styrene (0.50 mL, 4.25 mmol), Et_3N (0.30 mL, 2.13 mmol) and iodobenzene diacetate (412 mg, 1.28 mmol) in DCE (1.1 mL, total volume) was stirred at 40 °C in a sand bath for 24 hours. Yellow solid (114 mg, 63%).
The same procedure was followed to scale-up the reaction to 10 mmol of trifluorodiazoethane to get 1.25 g (59%).
In-situ generation of trifluorodiazoethane: To a solution of 2,2,2-trifluoroethan-1-amine hydrochloride (86 mg, 0.85 mmol) in DCE/H_2O (0.95 mL / 0.15 mL) was added $NaNO_2$ (70 mg, 1.02 mmol) and styrene (0.50 mL, 4.25 mmol). The reaction was stirred at 40 °C in a sand bath for 24 hours. Na_2SO_4 (50 mg), Et_3N (0.30 mL, 2.13 mmol) and iodobenzene diacetate (412 mg, 1.28 mmol) were added to the reaction mixture. The temperature was maintained at 40 °C in a sand bath for 24 hours. Yellow solid (100 mg, 55%).

^{1}H NMR (400 MHz, $CDCl_3$) δ 10.39 (s, 1H), 7.58 – 7.56 (m, 2H), 7.49 – 7.35 (m, 3H), 6.72 (s, 1H) ppm.
^{13}C{^{1}H} NMR (101 MHz, $CDCl_3$) δ 145.3, 143.7 (q, *J* = 38.3 Hz), 129.6, 129.4, 128.0, 125.8, 121.2 (q, *J* = 268.8 Hz), 101.2 (q, *J* = 2.0 Hz) ppm.
^{19}F NMR (376 MHz, $CDCl_3$) δ -62.2 ppm.
NMR data were in agreement with those reported.[S9]
M.p. 104 – 106 °C; **HRMS-ESI** m/z calcd for $C_{10}H_7F_3N_2$ $[M+H]^+$ 213.0634, found 213.0639.

[S9] Li, F.; Nie, J.; Sun, L.; Zheng, Y.; Ma, J.-A. *Angew. Chem. Int. Ed.* **2013**, *52*, 6255–6258.

5-(*o*-Tolyl)-3-(trifluoromethyl)-1*H*-pyrazole (5b)

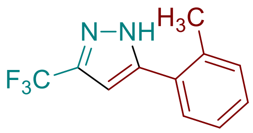

Following the general procedure a mixture of trifluorodiazoethane (0.85 ml, 1.0 M), 1-methyl-2-vinylbenzene (0.56 mL, 4.25 mmol), Et_3N (0.30 mL, 2.13 mmol) and iodobenzene diacetate (412 mg, 1.28 mmol) in DCE (1.1 mL, total volume) was stirred at 40 °C in a sand bath for 24 hours. Yellow solid (105 mg, 54%).

^{1}H NMR (400 MHz, $CDCl_3$) δ 11.64 (bs, 1H), 7.29 – 7.22 (m, 2H), 7.22 – 7.15 (m, 2H), 6.55 (s, 1H), 2.30 (s, 3H) ppm.
^{13}C{^{1}H} NMR (101 MHz, $CDCl_3$) δ 144.2, 143.3 (q, *J* = 38.3 Hz), 136.1, 131.2, 129.5, 128.9, 127.9, 126.4, 121.2 (q, *J* = 268.7 Hz), 103.9 (q, *J* = 2.0 Hz), 20.5 ppm.
^{19}F NMR (376 MHz, $CDCl_3$) δ -62.1 ppm.
NMR data were in agreement with those reported.[S10]
M.p. 83 – 85 °C; **HRMS-APCI** m/z calcd for $C_{12}H_{11}F_3N_2$ $[M+H]^+$ 241.0947, found 241.0950.

5-(*o*-Chlorophenyl)-3-(trifluoromethyl)-1*H*-pyrazole (5c)

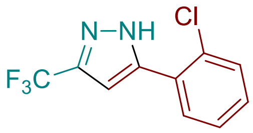

Following the general procedure a mixture of trifluorodiazoethane (1.06 mL, 0.75 M), 1-chloro-2-vinylbenzene (0.54 mL, 4.25 mmol), Et_3N (0.30 mL, 2.13 mmol) and iodobenzene diacetate (412 mg, 1.28 mmol) in DCE (1.1 mL, total volume) was stirred at 40 °C in a sand bath for 24 hours. Yellow solid (137 mg, 65%).

^{1}H NMR (400 MHz, $CDCl_3$) δ 7.60 – 7.56 (m, 1H), 7.54 – 7.50 (m, 1H), 7.40 – 7.36 (m, 2H), 6.88 (s, 1H) ppm.
^{13}C{^{1}H} NMR (101 MHz, $CDCl_3$) δ 143.6 (q, *J* = 38.1 Hz), 141.9, 131.7, 131.1, 130.7, 130.4, 127.8, 127.0, 121.3 (q, *J* = 268.6 Hz), 104.4 (q, *J* = 2.2 Hz) ppm.
^{19}F NMR (376 MHz, $CDCl_3$) δ -62.2 ppm.
NMR data were in agreement with those reported.[S10]
M.p. 101 – 103 °C; **HRMS-ESI** m/z calcd for $C_{10}H_6ClF_3N_2$ $[M+H]^+$ 247.0244, found 247.0250.

5-(*m*-Tolyl)-3-(trifluoromethyl)-1*H*-pyrazole (5d)

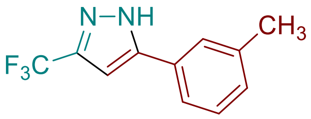

Following the general procedure a mixture of trifluorodiazoethane (0.85 ml, 1.0 M), 1-methyl-3-vinylbenzene (0.56 mL, 4.25 mmol), Et_3N (0.30 mL, 2.13 mmol) and iodobenzene diacetate (412 mg, 1.28 mmol) in DCE (1.1 mL, total volume) was stirred at 40 °C in a sand bath for 24 hours. Brown solid (142 mg, 73%).

^{1}H NMR (400 MHz, $CDCl_3$) δ 7.41 – 7.35 (m, 2H), 7.32 (t, *J* = 7.5 Hz, 1H), 7.22 (d, *J* = 7.4 Hz, 1H), 6.68 (s, 1H), 2.38 (s, 3H) ppm.
^{13}C{^{1}H} NMR (101 MHz, $CDCl_3$) δ 145.4, 143.6 (q, *J* = 38.2 Hz), 139.2, 130.3, 129.2, 127.9, 126.3, 122.8, 121.3 (q, *J* = 268.8 Hz), 101.0 (q, *J* = 1.8 Hz), 21.4 ppm.
^{19}F NMR (376 MHz, $CDCl_3$) δ -62.1 ppm.
NMR data were in agreement with those reported.[S10]
M.p. 68 – 70 °C; **HRMS-ESI** m/z calcd for $C_{11}H_9F_3N_2$ $[M+H]^+$ 227.0791, found 227.0791.

[S10] Wang, H.; Ning, Y.; Sun, Y.; Sivaguru, P.; Bi, X. *Org. Lett.* **2020**, *22*, 2012–2016.

5-(*m*-Bromophenyl)-3-(trifluoromethyl)-1*H*-pyrazole (5e)

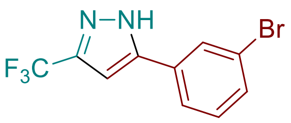

Following the general procedure a mixture of trifluorodiazoethane (0.85 ml, 1.0 M), 1-bromo-3-vinylbenzene (0.56 mL, 4.25 mmol), Et_3N (0.30 mL, 2.13 mmol) and iodobenzene diacetate (412 mg, 1.28 mmol) in DCE (1.1 mL, total volume) was stirred at 40 °C in a sand bath for 24 hours. Yellow solid (176 mg, 71%).

^{1}H NMR (400 MHz, $CDCl_3$) δ 7.59 (t, *J* = 1.8 Hz, 1H), 7.46 – 7.40 (m, 1H), 7.40 – 7.35 (m, 1H), 7.18 (t, *J* = 7.9 Hz, 1H), 6.61 (s, 1H) ppm.
^{13}C{^{1}H} NMR (101 MHz, $CDCl_3$) δ 144.2, 143.5 (q, *J* = 38.6 Hz), 132.5, 130.9, 129.9, 128.9, 124.1, 123.4, 121.0 (q, *J* = 268.9 Hz), 101.7 (q, *J* = 2.1 Hz) ppm.
^{19}F NMR (376 MHz, $CDCl_3$) δ -62.1 ppm.
NMR data were in agreement with those reported.[S11]
M.p. 78 – 80 °C; **HRMS-ESI** m/z calcd for $C_{10}H_6BrF_3N_2$ $[M+H]^+$ 290.9739, found 290.9741.

5-(*m*-Nitrophenyl)-3-(trifluoromethyl)-1*H*-pyrazole (5f)

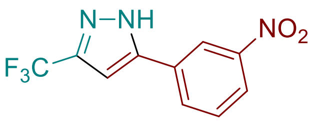

A mixture of trifluorodiazoethane (0.85 ml, 1.0 M), 1-nitro-3-vinylbenzene (0.59 mL, 4.25 mmol), Et_3N (0.30 mL, 2.13 mmol) and iodobenzene diacetate (1.09 g, 3.40 mmol) in DCE (1.1 mL, total volume) was stirred at 60 °C in a sand bath for 24 hours. Yellow solid (116 mg, 53%).

^{1}H NMR (400 MHz, $CDCl_3$) δ 8.50 (t, *J* = 1.9 Hz, 1H), 8.28 (ddd, *J* = 8.3, 2.2, 1.0 Hz, 1H), 7.97 (dt, *J* = 7.8, 1.1, 1H), 7.68 (t, *J* = 8.0 Hz, 1H), 6.95 (s, 1H) ppm.
^{13}C{^{1}H} NMR (101 MHz, $CDCl_3$) δ 149.0, 142.9 (q, *J* = 39.2 Hz), 131.5, 130.6, 130.4, 124.1, 120.77, 120.75 (q, *J* = 268.9 Hz), 102.8 (q, *J* = 2.1 Hz) ppm.
^{19}F NMR (376 MHz, $CDCl_3$) δ -62.1 ppm.
M.p. 160 – 162 °C; **HRMS-ESI** m/z calcd for $C_{10}H_7F_3N_3O_2$ $[M+H]^+$ 258.0490, found 258.0487.

5-(*p*-Tolyl)-3-(trifluoromethyl)-1*H*-pyrazole (5g)

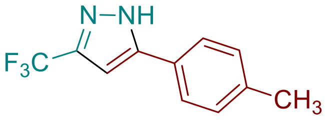

Following the general procedure a mixture of trifluorodiazoethane (1.06 mL, 0.75 M), 1-methyl-4-vinylbenzene (0.56 mL, 4.25 mmol), Et_3N (0.30 mL, 2.13 mmol) and iodobenzene diacetate (412 mg, 1.28 mmol) in DCE (1.1 mL, total volume) was stirred at 40 °C in a sand bath for 24 hours. Yellow solid (110 mg, 57%).
^{1}H NMR (400 MHz, $CDCl_3$) δ 7.46 (d, *J* = 8.2 Hz, 2H), 7.26 (d, *J* = 8.0 Hz, 2H), 6.72 (s, 1H), 2.40 (s, 3H) ppm.
^{13}C{^{1}H} NMR (101 MHz, $CDCl_3$) δ 145.2, 144.0 (q, *J* = 37.6 Hz), 139.8, 130.1, 125.7, 125.3, 121.3 (q, *J* = 268.5 Hz), 100.9, 21.5 ppm.
^{19}F NMR (376 MHz, $CDCl_3$) δ -62.2 ppm.
NMR data were in agreement with those reported.[S11]
M.p. 120 – 122 °C; **HRMS-ESI** m/z calcd for $C_{11}H_{10}F_3N_2$ $[M+H]^+$ 227.0796, found 227.0792.

5-(*p*-(*Tert*butyl)phenyl)-3-(trifluoromethyl)-1*H*-pyrazole (5h)

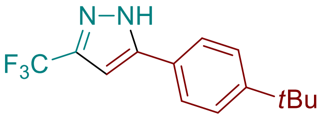

Following the general procedure a mixture of trifluorodiazoethane (0.85 ml, 1.0 M), 1-tertbutyl-4-vinylbenzene (0.77 mL, 4.25 mmol), Et_3N (0.30 mL, 2.13 mmol) and iodobenzene diacetate (412 mg, 1.28 mmol) in DCE (1.1 mL, total volume) was stirred at 40 °C for 24 hours. Pale yellow solid (137 mg, 60%).

[S11] Mei, H.; Wang, N.; Li, Z.; Han, J. *Org. Lett.* **2022**, *24*, 2258–2263

^{1}H NMR (400 MHz, $CDCl_3$) δ 7.56 – 7.42 (m, 4H), 6.71 (s, 1H), 1.36 (s, 9H) ppm.
^{13}C{^{1}H} NMR (101 MHz, $CDCl_3$) δ 152.8, 145.0, 143.8 (q, *J* = 38.3 Hz), 126.2, 125.4, 125.1, 121.2 (q, *J* = 268.7 Hz), 100.8 (q, *J* = 2.1 Hz), 34.8, 31.2 ppm.
^{19}F NMR (376 MHz, $CDCl_3$) δ -62.2 ppm.
NMR data were in agreement with those reported.[S10]
M.p. 99 – 101 °C; **HRMS-ESI** m/z calcd for $C_{14}H_{15}F_3N_2$ $[M+H]^+$ 269.1260, found 269.1264.

5-([1,1'-Biphenyl]-4-yl)-3-(trifluoromethyl)-1*H*-pyrazole (5i)

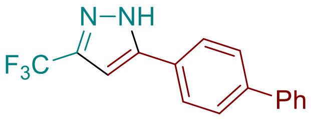

Following the general procedure a mixture of trifluorodiazoethane (0.85 ml, 1.0 M), 4-vinyl-1, 1'-biphenyl (766 mg, 4.25 mmol), Et_3N (0.30 mL, 2.13 mmol) and iodobenzene diacetate (412 mg, 1.28 mmol) in DCE (1.1 mL, total volume) was stirred at 40 °C in a sand bath for 24 hours. White solid (123 mg, 50%).

^{1}H NMR (400 MHz, $CDCl_3$) δ 11.20 (bs, 1H), 7.73 – 7.68 (m, 2H), 7.67 – 7.60 (m, 2H), 7.52 – 7.44 (m, 3H), 7.43 – 7.37 (m, 2H), 6.83 (s, 1H) ppm.
^{13}C{^{1}H} NMR (101 MHz, $CDCl_3$) δ 144.9, 142.5, 140.1, 129.1, 128.12, 128.07, 127.2, 126.2, 121.3 (q, *J* = 268.7 Hz), 114.0, 101.4 (q, *J* = 2.2 Hz) ppm.
^{19}F NMR (376 MHz, $CDCl_3$) δ -62.2 ppm.
NMR data were in agreement with those reported.[S12]
M.p. 184 – 186 °C; **HRMS-ESI** m/z calcd for $C_{16}H_{11}F_3N_2$ $[M+H]^+$ 289.0947, found 289.0950.

5-(4-Fluorophenyl)-3-(trifluoromethyl)-1*H*-pyrazole (5j)

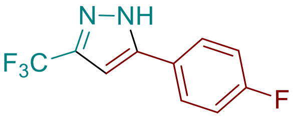

Following the general procedure a mixture of trifluorodiazoethane (1.03 ml, 0.8 M), 1-fluor-4-vinylbenzene (0.50 mL, 4.25 mmol), Et_3N (0.30 mL, 2.13 mmol) and iodobenzene diacetate (412 mg, 1.28 mmol) in DCE (1.1 mL, total volume) was stirred at 40 °C in a sand bath for 24 hours. Yellow solid (117 mg, 59%).

^{1}H NMR (400 MHz, $CDCl_3$) δ 7.59 – 7.44 (m, 2H), 7.17 – 7.02 (m, 2H), 6.66 (s, 1H) ppm.
^{13}C{^{1}H} NMR (101 MHz, $CDCl_3$) δ 163.5 (d, *J* = 250.0 Hz), 144.6, 143.7 (q, *J* = 38.2 Hz), 127.7 (d, *J* = 8.5 Hz), 124.4 (d, *J* = 4.0 Hz), 121.1 (q, *J* = 269.0 Hz), 116.5 (d, *J* = 22.7 Hz), 101.2 ppm.
^{19}F NMR (376 MHz, $CDCl_3$) δ -62.2 (CF_3), -111.0 (bs, F) ppm.
NMR data were in agreement with those reported.[S9]
M.p. 106 – 108 °C; **HRMS-ESI** m/z calcd for $C_{10}H_6F_4N_2$ $[M+H]^+$ 231.0540, found 231.0548.

5-(4-Chlorophenyl)-3-(trifluoromethyl)-1H-pyrazole (5k)

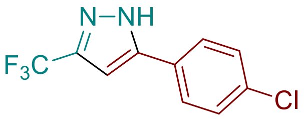

Following the general procedure a mixture of trifluorodiazoethane (0.85 ml, 1.0 M), 1-chloro-4-vinylbenzene (0.54 mL, 4.25 mmol), Et_3N (0.30 mL, 2.13 mmol) and iodobenzene diacetate (412 mg, 1.28 mmol) in DCE (1.1 mL, total volume) was stirred at 40 °C in a sand bath for 24 hours. Yellow solid (135 mg, 64%).

^{1}H NMR (400 MHz, $CDCl_3$) δ 13.06 (bs, 1H), 7.47 (d, *J* = 8.6 Hz, 2H), 7.39 (d, *J* = 8.6 Hz, 2H), 6.67 (s, 1H) ppm.
^{13}C{^{1}H} NMR (101 MHz, $CDCl_3$) δ 144.5, 143.6 (q, *J* = 38.3 Hz), 135.7, 129.6, 127.0, 126.4, 121.0 (q, *J* = 268.9 Hz), 101.4 (q, *J* = 2.2 Hz) ppm.
^{19}F NMR (376 MHz, $CDCl_3$) δ -62.1 ppm.
NMR data were in agreement with those reported.[S9]
M.p. 131 – 133 °C**; HRMS-ESI** m/z calcd for $C_{10}H_6ClF_3N_2$ $[M+H]^+$ 247.0244, found 247.0249.

[S12] Zhu, C.; Zeng, H.; Liu, C.; Cai, Y.; Fang, X.; Jiang, H. *Org. Lett.* **2020**, *22*, 809–813

5-(4-Bromophenyl)-3-(trifluoromethyl)-1*H*-pyrazole (5l)

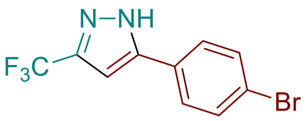

Following the general procedure a mixture of trifluorodiazoethane (0.85 ml, 1.0 M), 1-bromo-4-vinylbenzene (0.56 mL, 4.25 mmol), Et_3N (0.30 mL, 2.13 mmol) and iodobenzene diacetate (412 mg, 1.28 mmol) in DCE (1.1 mL, total volume) was stirred at 40 °C in a sand bath for 24 hours. Yellow solid (157 mg, 63%).

^{1}H NMR (400 MHz, $CDCl_3$) δ 7.57 (d, *J* = 8.5 Hz, 2H), 7.42 (d, *J* = 8.5 Hz, 2H), 6.72 (s, 1H) ppm.
^{13}C{^{1}H} NMR (101 MHz, $CDCl_3$) δ 144.5, 143.7 (q, *J* = 38.3 Hz), 132.6, 127.2, 126.9, 123.8, 121.0 (q, *J* = 268.8 Hz), 101.5 (q, *J* = 2.2 Hz) ppm.
^{19}F NMR (376 MHz, $CDCl_3$) δ -62.2 ppm.
NMR data were in agreement with those reported.[S9]
M.p. 140 – 142 °C; **HRMS-ESI** m/z calcd for $C_{10}H_6BrF_3N_2$ $[M+H]^+$ 290.9739, found 290.9736.

5-(*p*-(Anisol)-3-(trifluoromethyl)-1*H*-pyrazole (5m)

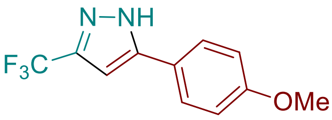

Following the general procedure a mixture of trifluorodiazoethane (0.85 ml, 1.0 M), 1-methoxy-4-vinylbenzene (0.57 mL, 4.25 mmol), Et_3N (0.30 mL, 2.13 mmol) and iodobenzene diacetate (412 mg, 1.28 mmol) in DCE (1.1 mL, total volume) was stirred at 40 °C in a sand bath for 24 hours. Yellow solid (157 mg, 76%).

^{1}H NMR (400 MHz, $CDCl_3$) δ 7.48 (d, *J* = 8.7 Hz, 2H), 6.96 (d, *J* = 8.6 Hz, 2H), 6.63 (s, 1H), 3.85 (s, 3H) ppm.
^{13}C{^{1}H} NMR (101 MHz, $CDCl_3$) δ 160.6, 145.1, 143.8 (q, *J* = 36.3 Hz), 127.2, 121.3 (q, *J* = 268.7 Hz), 120.7, 114.8, 100.4, 55.5 ppm.
^{19}F NMR (376 MHz, $CDCl_3$) δ -62.2 ppm.
NMR data were in agreement with those reported.[S9]
M.p. 125 – 127 °C; **HRMS-ESI** m/z calcd for $C_{11}H_9F_3N_2O$ $[M+H]^+$ 243.0740, found 243.0732.

5-(*p*-(Methyltio)phenyl-3-(trifluoromethyl)-1*H*-pyrazole (5n)

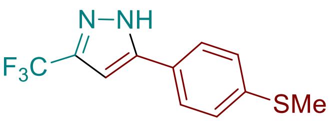

Following the general procedure a mixture of trifluorodiazoethane (0.53 mL, 1.6 M), methyl(4-vinylphenyl)sulfane (639 mg, 4.25 mmol), Et_3N (0.30 mL, 2.13 mmol) and iodobenzene diacetate (412 mg, 1.28 mmol) in DCE (1.1 mL, total volume) was stirred at 40 °C in a sand bath for 24 hours. Yellow solid (112 mg, 51%).

^{1}H NMR (400 MHz, $CDCl_3$) δ 7.48 (d, *J* = 8.4 Hz, 2H), 7.30 (d, *J* = 8.5 Hz, 2H), 6.72 (s, 1H), 2.52 (s, 3H) ppm.
^{13}C{^{1}H} NMR (101 MHz, $CDCl_3$) δ 144.8, 143.9 (q, *J* = 38.1 Hz), 140.9, 126.8, 126.1, 124.6, 121.2 (q, *J* = 268.7 Hz), 101.3 (q, *J* = 2.2 Hz), 15.5 ppm.
^{19}F NMR (376 MHz, $CDCl_3$) δ -62.2 ppm.
NMR data were in agreement with those reported.[S12]
M.p. 120 – 122 °C; **HRMS-ESI** m/z calcd for $C_{11}H_{10}F_3N_2S$ $[M+H]^+$ 259.0511, found 259.0513.

4-(3-(Trifluoromethyl)-1*H*-pyrazol-5-yl)phenyl acetate (5o)

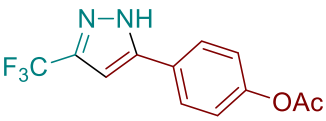

Following the general procedure a mixture of trifluorodiazoethane (0.85 ml, 1.0 M), 4-vinylphenyl acetate (0.65 mL, 4.25 mmol), Et_3N (0.30 mL, 2.13 mmol) and iodobenzene diacetate (412 mg, 1.28 mmol) in DCE (1.1 mL, total volume) was stirred at 40 °C in a sand bath for 24 hours. Pale yellow solid (146 mg, 64%).

^{1}H NMR (400 MHz, $CDCl_3$) δ 11.79 (bs, 1H), 7.50 (d, *J* = 8.7 Hz, 2H), 7.14 (d, *J* = 8.6 Hz, 2H), 6.70 (s, 1H), 2.34 (s, 3H) ppm.
$^{13}C\{^1H\}$ NMR (101 MHz, $CDCl_3$) δ 169.9, 151.4, 144.3, 143.9 (q, *J* = 38.0 Hz), 127.0, 126.0, 122.7, 121.2 (q, *J* = 268.8 Hz), 101.4 (q, *J* = 2.2 Hz), 21.3 ppm.
^{19}F NMR (376 MHz, $CDCl_3$) δ -62.2 ppm.
M.p. 111 – 113 °C; **HRMS-ESI** m/z calcd for $C_{12}H_9F_3N_2O_2$ $[M+H]^+$ 271.0689, found 271.0684.

5-(*p*-Benzonitrile)-3-(trifluoromethyl)-1*H*-pyrazole (5p)

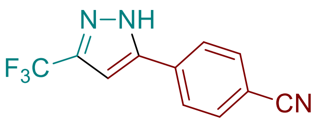

Following the general procedure a mixture of trifluorodiazoethane (0.53 mL, 1.6 M), 4-vinylbenzonitrile (0.55 mL, 4.25 mmol), Et_3N (0.30 mL, 2.13 mmol) and iodobenzene diacetate (412 mg, 1.28 mmol) in DCE (1.1 mL, total volume) was stirred at 40 °C in a sand bath for 24 hours. Yellow solid (103 mg, 51%).

^{1}H NMR (400 MHz, $CDCl_3$) δ 11.47 (bs, 1H), 7.84 – 7.68 (m, 4H), 6.91 (s, 1H) ppm.
$^{13}C\{^1H\}$ NMR (101 MHz, $CDCl_3$) δ 144.2, 133.3, 132.7, 126.3, 120.8 (q, *J* = 268.9 Hz), 118.3, 113.1, 102.9 (q, *J* = 2.2 Hz) ppm.
^{19}F NMR (376 MHz, $CDCl_3$) δ -61.3 ppm.
NMR data were in agreement with those reported.[S9]
M.p. 178 – 180 °C; **HRMS-ESI** m/z calcd for $C_{11}H_7F_3N_3$ $[M+H]^+$ 238.0592, found 238.0589.

5-(*p*-(Trifluoromethyl)phenyl)-3-(trifluoromethyl)-1*H*-pyrazole (5q)

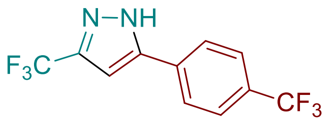

Following the general procedure a mixture of trifluorodiazoethane (1.06 mL, 0.75 M), 1-trifluoromethyl-4-vinylbenzene (0.63 mL, 4.25 mmol), Et_3N (0.30 mL, 2.13 mmol) and iodobenzene diacetate (412 mg, 1.28 mmol) in DCE (1.1 mL, total volume) was stirred at 40 °C in a sand bath for 24 hours. White solid (156 mg, 65%).

^{1}H NMR (400 MHz, $CDCl_3$) δ 7.71-7.66 (m, 4H), 6.78 (s, 1H) ppm.
$^{13}C\{^1H\}$ NMR (126 MHz, $CDCl_3$) δ 144.3, 143.8 (q, *J* = 38.6 Hz), 131.6 (q, *J* = 33.0 Hz), 131.3, 126.5 (q, *J* = 3.8 Hz), 126.0, 123.9 (q, *J* = 272.3 Hz), 120.9 (q, *J* = 268.9 Hz), 102.3 (q, *J* = 2.2 Hz) ppm.
^{19}F NMR (376 MHz, $CDCl_3$) δ -62.2, -63.0 ppm.
NMR data were in agreement with those reported.[S11]
M.p. 141 – 143 °C; **HRMS-ESI** m/z calcd for $C_{11}H_6F_6N_2$ $[M+H]^+$ 281.0508, found 281.0517.

Methyl 4-(3-(trifluoromethyl)-1*H*-pyrazol-5-yl)benzoate (5r)

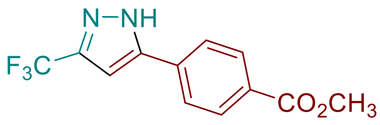

Following the general procedure a mixture of trifluorodiazoethane (1.06 mL, 0.75 M), methyl 4-vinylbenzoate (689 mg, 4.25 mmol), Et_3N (0.30 mL, 2.13 mmol) and iodobenzene diacetate (412 mg, 1.28 mmol) in DCE (1.1 mL, total volume) was stirred at 40 °C in a sand bath for 24 hours. White solid (119 mg, 52%).

^{1}H NMR (500 MHz, $CDCl_3$) δ 11.51 (bs, 1H), 8.13 (d, *J* = 8.2 Hz, 2H), 7.67 (d, *J* = 8.1 Hz, 2H), 6.87 (s, 1H), 3.95 (s, 3H) ppm.
$^{13}C\{^1H\}$ NMR (101 MHz, $CDCl_3$) δ 166.5, 144.4, 143.9 (q, *J* = 39.1 Hz), 132.3, 131.0, 130.8, 125.6, 121.1 (q, *J* = 268.5 Hz), 102.4 (q, *J* = 2.1 Hz), 52.6 ppm.
^{19}F NMR (376 MHz, $CDCl_3$) δ -62.3 ppm.
NMR data were in agreement with those reported.[S12]
M.p. 178 – 180 °C; **HRMS-APCI** m/z calcd for $C_{13}H_{11}F_3N_2O_2$ $[M+H]^+$ 285.0845, found 285.0844.

5-(4-(4,4,5,5-Tetramethyl-1,3,2-dioxaborolan-2-yl)phenyl)-3-(trifluoromethyl)-1*H*-pyrazole (5s)

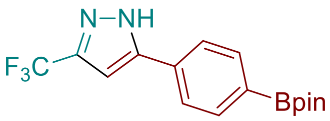

Following the general procedure a mixture of trifluorodiazoethane (1.06 mL, 0.75 M), 1-(4,4,5,5-tetramethyl-1,3,2-dioxaborolan-2-yl)-4-vinylbenzene (977 mg, 4.25 mmol), Et_3N (0.30 mL, 2.13 mmol) and iodobenzene diacetate (412 mg, 1.28 mmol) in DCE (1.1 mL, total volume) was stirred at 40 °C in a sand bath for 24 hours. Yellow solid (215 mg, 75%).

^{1}H NMR (400 MHz, $CDCl_3$) δ 7.87 (d, *J* = 8.2 Hz, 2H), 7.57 (d, *J* = 8.2 Hz, 2H), 6.77 (s, 1H), 1.36 (s, 12H) ppm.
^{13}C{^{1}H} NMR (126 MHz, $CDCl_3$) δ 145.0, 143.8 (q, *J* = 38.4 Hz), 135.7, 135.1, 130.4, 124.8, 121.2 (q, *J* = 268.6 Hz), 101.5, 84.2, 24.9 ppm.
^{19}F NMR (376 MHz, $CDCl_3$) δ -62.1 ppm.
^{11}B NMR (128 MHz, $CDCl_3$) δ 33.5 ppm.
M.p. 151 – 153 °C; **HRMS-ESI** m/z calcd for $C_{16}H_{18}BF_3N_2O_2$ $[M+H]^+$ 338.1523, found 338.1530.

5-(2,5-Dimethylphenyl)-3-(trifluoromethyl)-1*H*-pyrazole (5t)

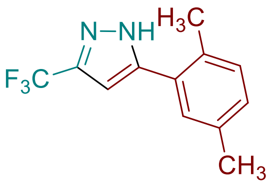

Following the general procedure a mixture of trifluorodiazoethane (1.06 mL, 0.75 M), 1,4-dimethyl-2-vinylbenzene (0.62 mL, 4.25 mmol), Et_3N (0.30 mL, 2.13 mmol) and iodobenzene diacetate (412 mg, 1.28 mmol) in DCE (1.1 mL, total volume) was stirred at 40 °C in a sand bath for 24 hours. Yellow solid (137 mg, 67%).

^{1}H NMR (400 MHz, $CDCl_3$) δ 10.99 (bs, 1H), 7.23 – 7.19 (m, 2H), 7.19 – 7.15 (m, 1H), 6.65 (s, 1H), 2.37 (s, 3H), 2.36 (s, 3H) ppm.
^{13}C{^{1}H} NMR (101 MHz, $CDCl_3$) δ 144.2, 143.5 (q, *J* = 38.0 Hz), 136.1, 133.0, 131.2, 130.3, 129.4, 127.7, 121.3 (q, *J* = 268.6 Hz), 103.7 (q, *J* = 1.8 Hz), 20.8, 20.1 ppm.
^{19}F NMR (376 MHz, $CDCl_3$) δ -62.1 ppm.
M.p. 103 – 105 °C; **HRMS-ESI** m/z calcd for $C_{12}H_{11}F_3N_2$ $[M+H]^+$ 241.0947, found 241.0947.

5-(3,5-Bis(trifluoromethyl)phenyl)-3-(trifluoromethyl)-1H-pyrazole (5v)

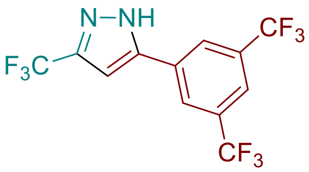

A mixture of trifluorodiazoethane (0.85 ml, 1.0 M), 1,3-bis(trifluoromethyl)-5-vinylbenzene (0.62 mL, 4.25 mmol), Et_3N (0.30 mL, 2.13 mmol) and iodobenzene diacetate (1.09 g, 3.40 mmol) in DCE (1.1 mL, total volume) was stirred at 60 °C in a sand bath for 24 hours. Yellow solid (104 mg, 35%).

^{1}H NMR (400 MHz, $CDCl_3$) δ 12.63 (s, 1H), 8.05 (s, 2H), 7.91 (s, 1H), 6.95 (s, 1H) ppm.
^{13}C{^{1}H} NMR (101 MHz, $CDCl_3$) δ 144.1, 133.1 (q, *J* = 33.9 Hz), 130.7, 125.9 (q, *J* = 4.0 Hz), 124.35, 123.2 – 123.0 (m), 123.0 (q, *J* = 273.0 Hz), 120.5 (q, *J* = 269.0 Hz), 103.0 (q, *J* = 2.0 Hz) ppm.
^{19}F NMR (376 MHz, $CDCl_3$) δ -62.3 (bs, 3F, CF_3), -63.3 (s, 6F, 2xCF_3) ppm.
M.p. 118 – 120 °C; **HRMS-ESI** m/z calcd for $C_{12}H_6F_9N_2$ $[M+H]^+$ 349.0387, found 349.0386.

5-(Perfluorophenyl)-3-(trifluoromethyl)-1*H*-pyrazole (5w)

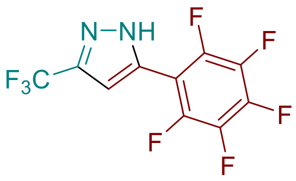

A mixture of trifluorodiazoethane (0.55 mL, 1.5 M), 1,2,3,4,5-pentafluoro-6-vinylbenzene (0.59 mL, 4.25 mmol), Et_3N (0.30 mL, 2.13 mmol) and iodobenzene diacetate (412 mg, 1.28 mmol) in DCE (1.1 mL, total volume) was stirred at 40 °C in a sand bath for 24 hours. White solid (135 mg, 53%).

^{1}H NMR (400 MHz, $CDCl_3$) δ 11.90 (bs, 1H), 7.09 (s, 1H) ppm.
^{13}C{^{1}H} NMR (101 MHz, $CDCl_3$) δ 146.1 – 144.7 (m), 143.9 (q, *J* = 37.6 Hz), 143.8 – 142.0 (m), 140.7 – 139.0 (m), 138.0 – 136.4 (m), 131.2, 120.9 (q, *J* = 268.8 Hz), 106.4 ppm.

^{19}F NMR (376 MHz, $CDCl_3$) δ -62.5 (s), -138.66 – -143.84 (m), -151.62 (t, *J* = 20.8 Hz), -160.02 (t, *J* = 17.9 Hz) ppm.
M.p. 118 – 120 °C; **HRMS-ESI-** m/z calcd for $C_{10}H_1F_8N_2$ $[M-H]^-$ 301.0017, found 301.002.

2-(3-(Trifluoromethyl)-1H-pyrazol-5-yl)pyridine (5x)

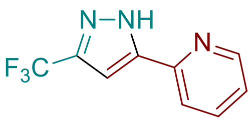

Following the general procedure a mixture of trifluorodiazoethane (1.06 mL, 0.75 M), methyl 2-vinylpyridine (0.46 mL, 4.25 mmol), Et_3N (0.30 mL, 2.13 mmol) and iodobenzene diacetate (412 mg, 1.28 mmol) in DCE (1.1 mL, total volume) was stirred at 40 °C in a sand bath for 24 hours. Yellow solid (109 mg, 60%).

^{1}H NMR (400 MHz, $CDCl_3$) δ 13.01 (bs, 1H), 8.73 (d, *J* = 5.0 Hz, 1H), 7.83 (td, *J* = 7.8, 1.7 Hz, 1H), 7.68 (d, *J* = 7.9 Hz, 1H), 7.35 (dd, *J* = 7.6, 4.9 Hz, 1H), 6.97 (s, 1H) ppm.
^{13}C{^{1}H} NMR (126 MHz, $CDCl_3$) δ 149.8, 146.9, 144.4 (q, *J* = 37.9 Hz), 143.1, 137.9, 124.1, 121.3 (q, *J* = 269.1 Hz), 120.7, 101.7 ppm.
^{19}F NMR (376 MHz, $CDCl_3$) δ -62.2 ppm.
M.p. 138 – 140 °C; **HRMS-ESI** m/z calcd for $C_9H_6F_3N_3$ $[M+H]^+$ 214.0587, found 214.0591.

5-(Tiophen-2-yl)-3-(trifluoromethyl)-1*H*-pyrazole (5y)

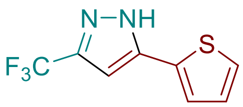

A mixture of trifluorodiazoethane (0.55 mL, 1.5 M), 2-vinyltiophene (0.43 mL, 4.25 mmol), Et_3N (0.30 mL, 2.13 mmol) and iodobenzene diacetate (412 mg, 1.28 mmol) in DCE (1.1 mL, total volume) was stirred at 40 °C in a sand bath for 24 hours. Yellow solid (30 mg, 16%).

^{1}H NMR (400 MHz, $CDCl_3$) δ 12.07 (bs, 1H), 7.37 (dd, *J* = 5.1, 0.9 Hz, 1H), 7.29 (dd, *J* = 3.7, 0.9 Hz, 1H), 7.08 (dd, *J* = 5.1, 3.7 Hz, 1H), 6.65 (s, 1H) ppm.
^{13}C{^{1}H} NMR (101 MHz, $CDCl_3$) δ 143.2 (q, *J* = 33.6 Hz), 139.7, 129.8, 128.1, 126.6, 125.5, 120.9 (q, *J* = 268.8 Hz), 101.5 ppm.
^{19}F NMR (376 MHz, $CDCl_3$) δ -62.2 ppm.
NMR data were in agreement with those reported.[S12]
M.p. 90 – 92 °C; **HRMS-ESI+** m/z calcd for $C_8H_6F_3N_2S$ $[M+H]^+$ 219.0198, found 219.0199.

***Tert*-*B*utyl-3-(trifluoromethyl)-1*H*-pyrazole-5-carboxylate (5z)**

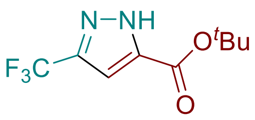

A mixture of trifluorodiazoethane (0.55 mL, 1.5 M), ^{t}butyl acrylate (0.62 mL, 4.25 mmol), Et_3N (0.30 mL, 2.13 mmol) and iodobenzene diacetate (412 mg, 1.28 mmol) in DCE (1.1 mL, total volume) was stirred at 40 °C in a sand bath for 24 hours. Yellow solid (95 mg, 47%).

^{1}H NMR (400 MHz, $CDCl_3$) δ 11.82 (bs, 1H), 7.01 (s, 1H), 1.60 (s, 9H) ppm.
^{13}C{^{1}H} NMR (101 MHz, $CDCl_3$) δ 158.0, 144.0 (q, *J* = 39.1, 37.8 Hz), 136.8, 120.8 (q, *J* = 268.8 Hz), 107.1 (q, *J* = 2.2 Hz), 84.2, 28.3 ppm.
^{19}F NMR (376 MHz, $CDCl_3$) δ -62.4 ppm.
NMR data were in agreement with those reported.[S13]
M.p. 87 – 89 °C; **HRMS-ESI** m/z calcd for $C_9H_{11}F_3N_2O_2$ $[M+H]^+$ 237.0845, found 237.0835.

[S13] Mertens, L.; Hock, K. J.; Koenigs, R. M. *Chem. Eur. J.* **2016**, *22*, 9542–9545

3-(Perfluoroethyl)-5-phenyl-1*H*-pyrazole (5aa)

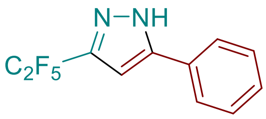

To a solution of 2,2,3,3,3-pentafluoropropan-1-amine-hydrochloride (157 mg, 0.85 mmol) in DCE/H_2O (0.95 mL / 0.15 mL) was added $NaNO_2$ (70 mg, 1.02 mmol) and styrene (0.50 mL, 4.25 mmol). The reaction was stirred at 40 °C in a sand bath for 24 hours. Na_2SO_4 (50 mg), Et_3N (0.30 mL, 2.13 mmol) and iodobenzene diacetate (412 mg, 1.28 mmol) were added to the reaction mixture. The temperature was maintained at 40 °C in a sand bath for 24 hours. Pale yellow solid (113 mg, 51%).

^{1}H NMR (400 MHz, $CDCl_3$) δ 12.31 (bs, 1H), 7.59 – 7.56 (m, 2H), 7.48 – 7.40 (m, 3H), 6.78 (s, 1H) ppm.
^{13}C{^{1}H} NMR (101 MHz, $CDCl_3$) δ 145.5, 142.3 (t, *J* = 28.4 Hz), 129.6, 129.4, 128.0, 125.7, 119.0 (qt, *J* = 285.9, 37.8 Hz), 110.8 (tq, *J* = 251.0, 39.3 Hz), 102.5 ppm.
^{19}F NMR (376 MHz, $CDCl_3$) δ -84.6 (t, *J* = 2.3 Hz), -113.2 (q, *J* = 2.3 Hz) ppm.
NMR data were in agreement with those reported.[S11]
M.p. 110 – 112 °C; **HRMS-ESI** m/z calcd for $C_{11}H_7F_5N_2$ $[M+H]^+$ 263.0602, found 263.0604.

4-Bromo-5-phenyl-3-(trifluoromethyl)-1*H*-pyrazole (6)

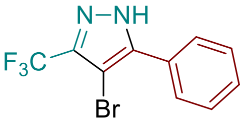

N-bromosuccinimide (34 mg, 0.19 mmol) was added to a solution of pyrazole **5a** (40 mg, 0.19 mmol) in CH_2Cl_2 (4 mL). The reaction was stirred at 40 °C in a sand bath for 12 hours. The crude was evaporated and purified by column chromatography (hexane/ethyl acetate 8:2) to afford 4-bromopyrazole **6**. White solid (41 mg, 75%).

^{1}H NMR (400 MHz, $CDCl_3$) δ 7.69 – 7.64 (m, 2H), 7.59 – 7.47 (m, 3H) ppm.
^{13}C{^{1}H} NMR (101 MHz, $CDCl_3$) δ 143.3, 142.4 (q, *J* = 37.0 Hz), 130.2, 129.4, 127.7, 127.0, 120.7 (q, *J* = 269.7 Hz), 90.5 ppm.
^{19}F NMR (376 MHz, $CDCl_3$) δ -62.7 ppm.
NMR data were in agreement with those reported.[S14]
M.p. 184 – 186 °C; **HRMS-ESI** m/z calcd for $C_{10}H_7BrF_3N_2$ $[M+H]^+$ 290.9745, found 290.9741.

Ethyl 2-(5-([1,1'-biphenyl]-4-yl)-3-(trifluoromethyl)-1H-pyrazol-1-yl)acetate (7)

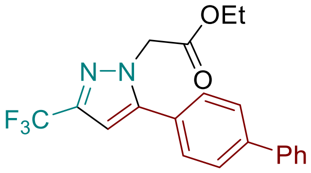

K_2CO_3 (112 mg, 0.96 mmol) was added in one portion to a solution of pyrazole **5i** (93 mg, 0.32 mmol) in acetone (5 mL). Then, ethyl 2-bromoacetate (36 µL, 0.32 mmol) was slowly added to the stirred mixture. The reaction was stirred under reflux in a sand bath for 12 h. Upon completion of the reaction, the crude was evaporated and ethyl acetate and water were added. The aqueous phase was extracted with ethyl acetate. The extracts were dried, filtered, and evaporated. The residue was purified by column chromatography (hexane/ethyl acetate 9:1) to afford the pyrazole **7** (white solid, 51 mg, 42%).

^{1}H NMR (500 MHz, $CDCl_3$) δ 7.70 (d, *J* = 8.2 Hz, 2H), 7.63 (d, *J* = 7.1 Hz, 2H), 7.52 – 7.46 (m, 4H), 7.44 – 7.36 (m, 1H), 6.64 (s, 1H), 4.95 (s, 2H), 4.23 (q, *J* = 7.1 Hz, 2H), 1.26 (t, *J* = 7.1 Hz, 3H).
^{13}C{^{1}H} NMR (126 MHz, $CDCl_3$) δ 167.5, 146.1, 142.9 (q, *J* = 38.4 Hz), 142.7, 140.0, 129.4, 129.1, 128.1, 127.83, 127.79, 127.2, 121.3 (q, *J* = 268.7 Hz), 105.0 (q, *J* = 2.2 Hz), 62.2, 51.8, 14.2 ppm.
^{19}F NMR (282 MHz, $CDCl_3$) δ -61.4 ppm.
M.p. 120 – 123 °C; **HRMS-ESI** m/z calcd for $C_{20}H_{17}F_3N_2O_2Na$ $[M+Na]^+$ 397.1134, found 397.1112.

[S14] Samanta, R. C.; Yamamoto, H. *Chem. Eur. J.* **2015**, *21*, 11976–11979

Ethyl 2-(3-([1,1'-biphenyl]-4-yl)-5-(trifluoromethyl)-1H-pyrazol-1-yl)acetate (7´)

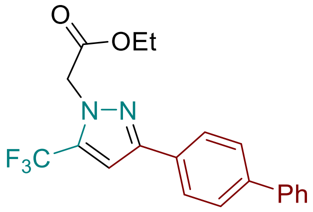

Isomer **7´** was also purified from the reaction mixture (white solid, 45 mg, 37%).

^{1}H NMR (500 MHz, $CDCl_3$) δ 7.87 (d, *J* = 8.2 Hz, 2H), 7.66 (d, *J* = 8.2 Hz, 2H), 7.65 – 7.63 (m, 2H), 7.48 – 7.44 (m, 2H), 7.39 – 7.34 (m, 1H), 7.01 (s, 1H), 5.08 (s, 2H), 4.26 (q, *J* = 7.1 Hz, 2H), 1.29 (t, *J* = 7.1 Hz, 3H) ppm.
^{13}C{^{1}H} NMR (126 MHz, $CDCl_3$) δ 166.9, 151.3, 141.5, 140.7, 133.9 (q, *J* = 39.4 Hz), 129.0, 127.64, 127.58, 127.2, 126.3, 120.0 (q, *J* = 269.0 Hz), 105.6 (q, *J* = 2.3 Hz), 62.3, 52.6, 14.2 ppm.
^{19}F NMR (282 MHz, $CDCl_3$) δ -59.1 ppm.
M.p. 105 – 107 °C; **HRMS-ESI** m/z calcd for $C_{20}H_{17}F_3N_2O_2Na$ $[M+Na]^+$ 397.1134, found 397.1132.

NMR spectra

2,2,2-Trifluorodiazoethane, DCE solution (1)

^1H NMR (400 MHz, $CDCl_3$)

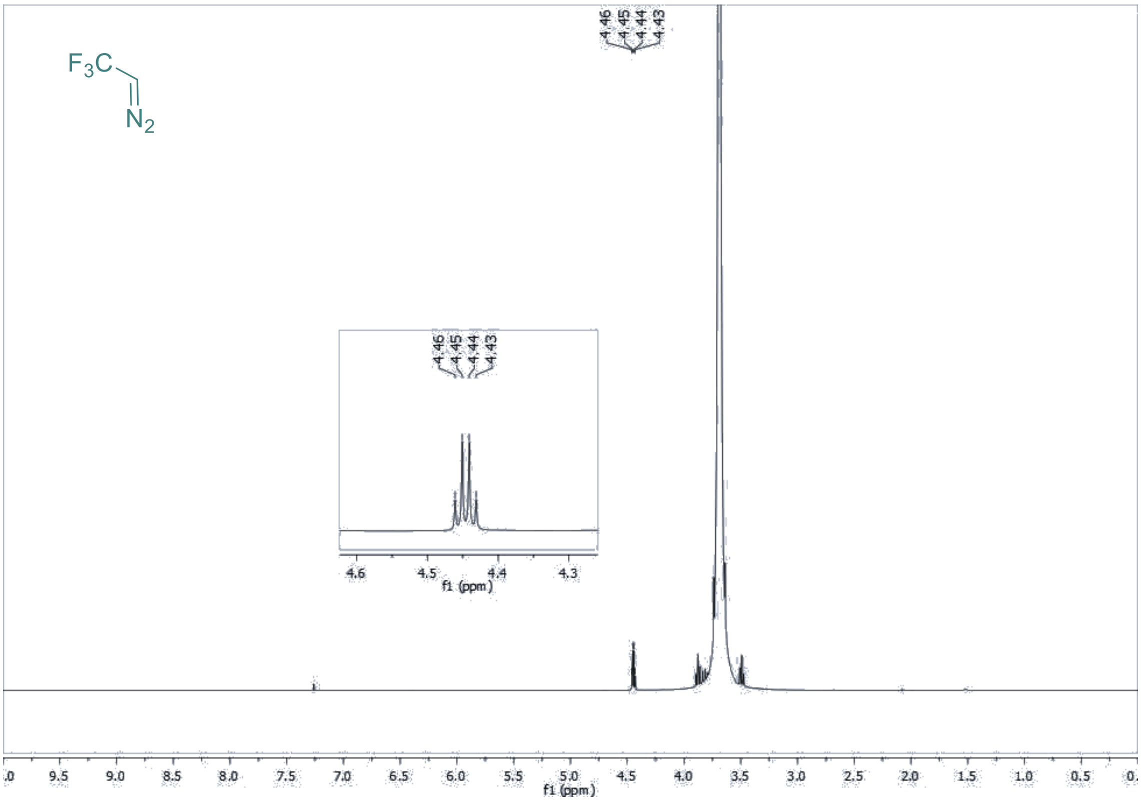

^{19}F NMR (376 MHz, $CDCl_3$)

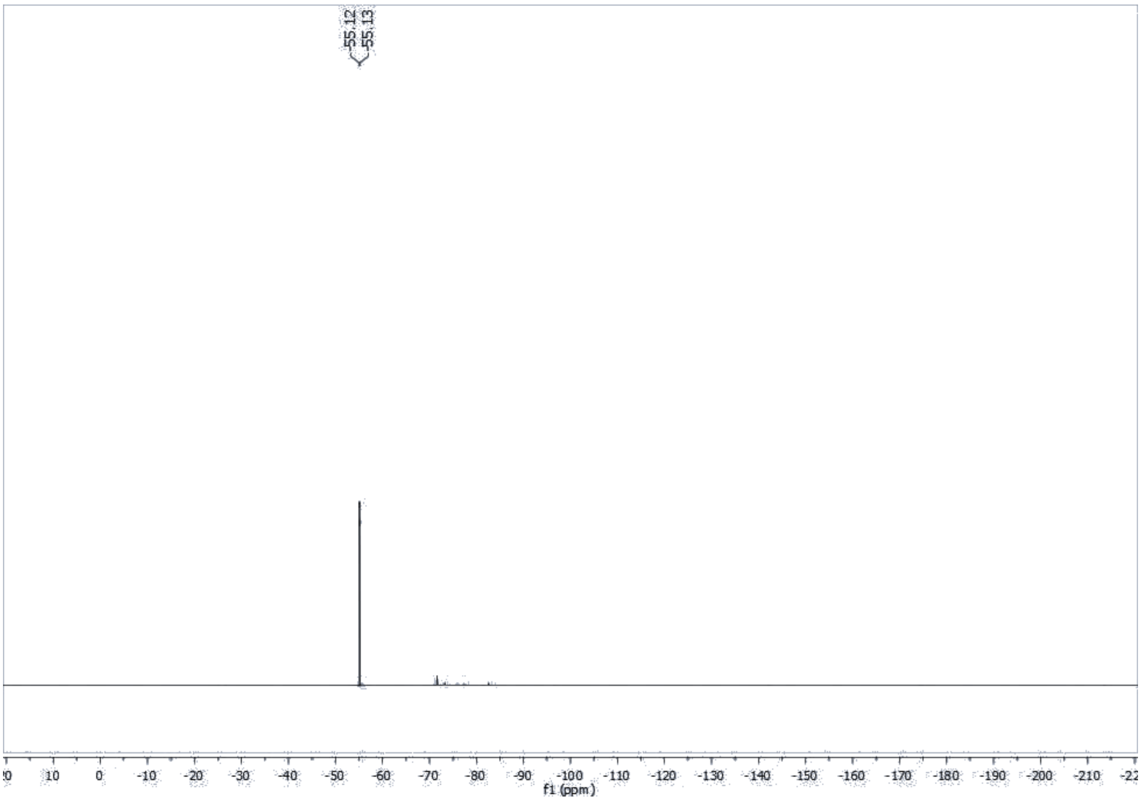

Methyl(4-vinylphenyl)sulfane (2n)

^{1}H NMR (300 MHz, $CDCl_3$)

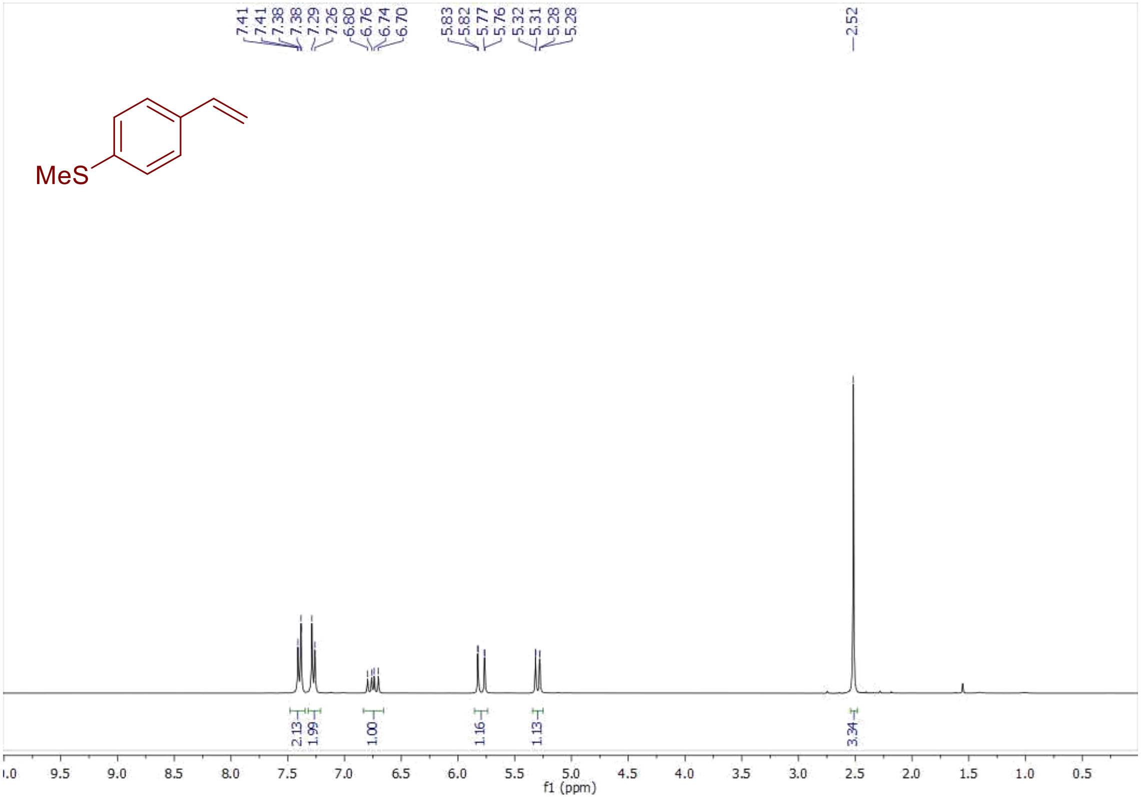

Methyl 4-vinylbenzoate (2r)

^{1}H NMR (300 MHz, $CDCl_3$)

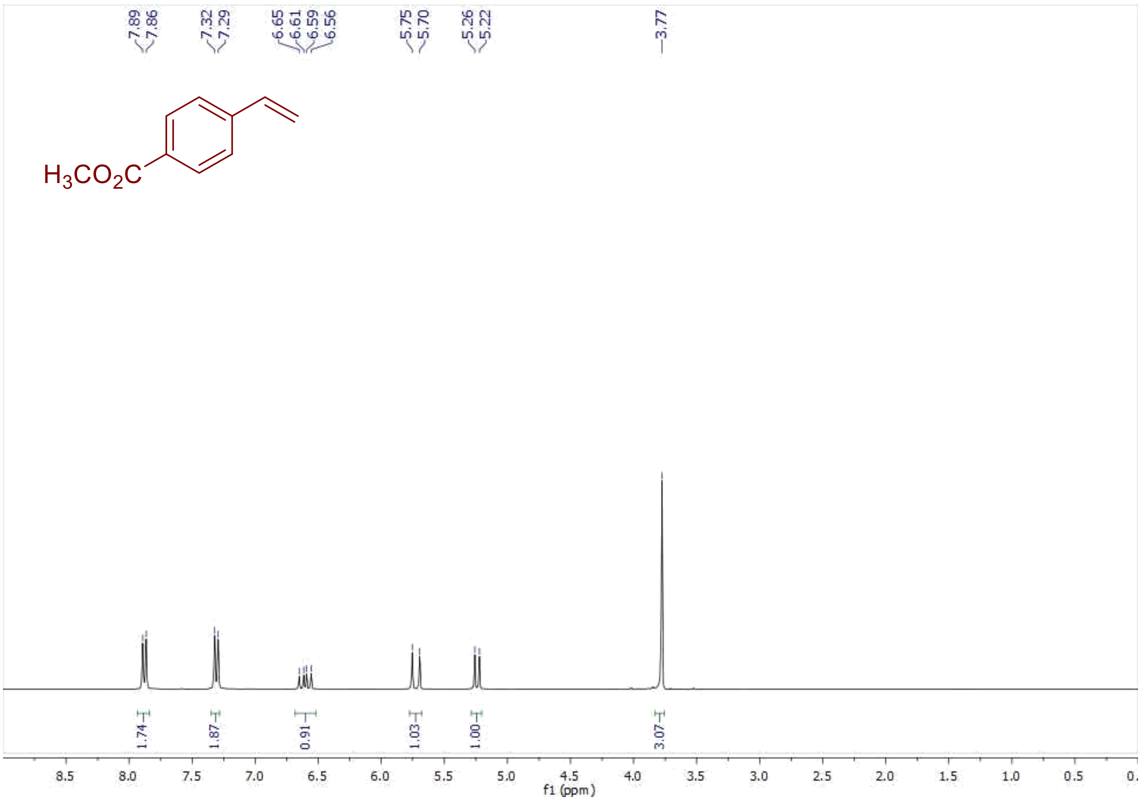

4,4,5,5-Tetramethyl-2-(4-vinylphenyl)-1,3,2-dioxaborolane (2s)

^{1}H NMR (300 MHz, $CDCl_3$)

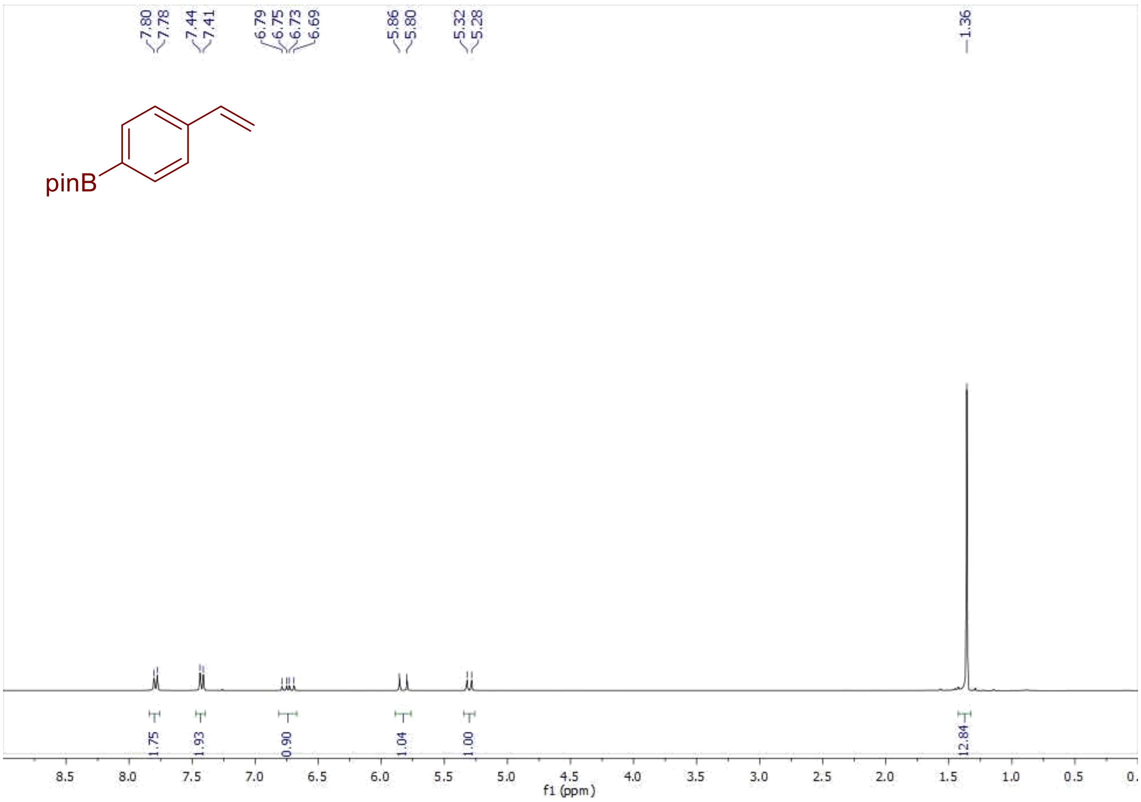

4-Methyl-*N*-(4-vinylphenyl)benzenesulfonamide (S2a)

^{1}H NMR (300 MHz, $CDCl_3$)

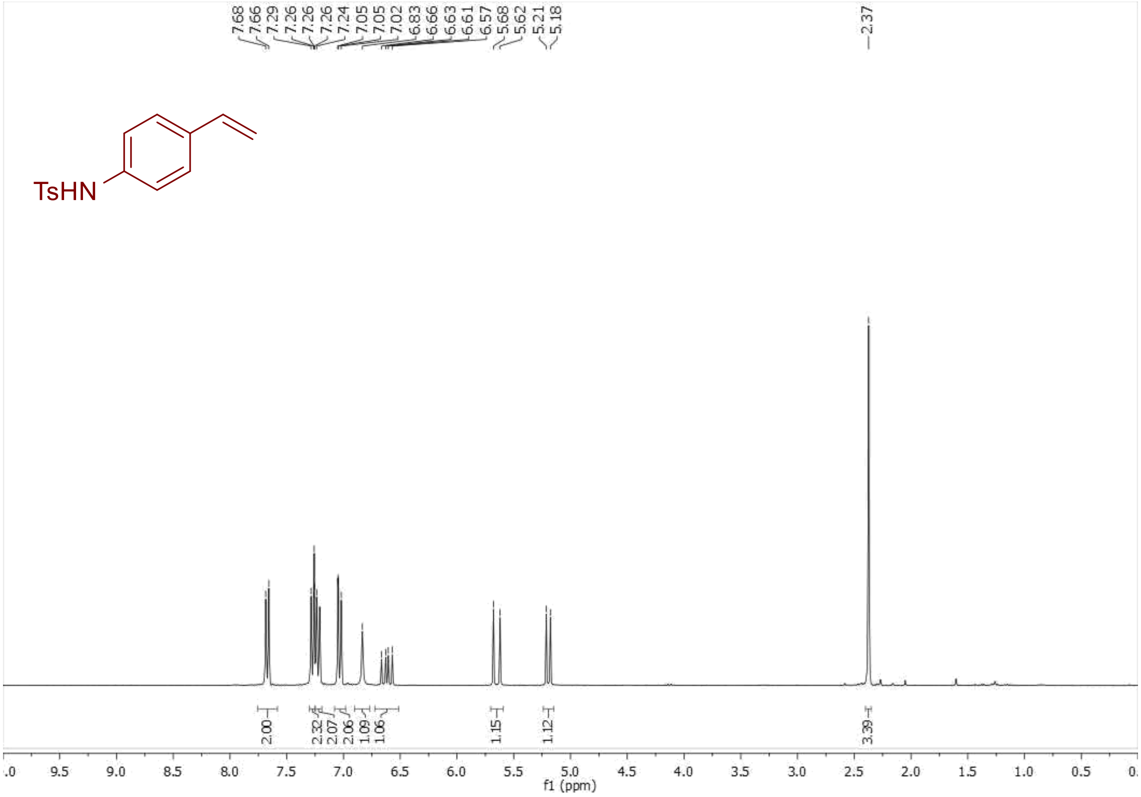

_N_,_N_-Diphenyl-4-vinylaniline (S2b)

^1H NMR (300 MHz, $CDCl_3$)

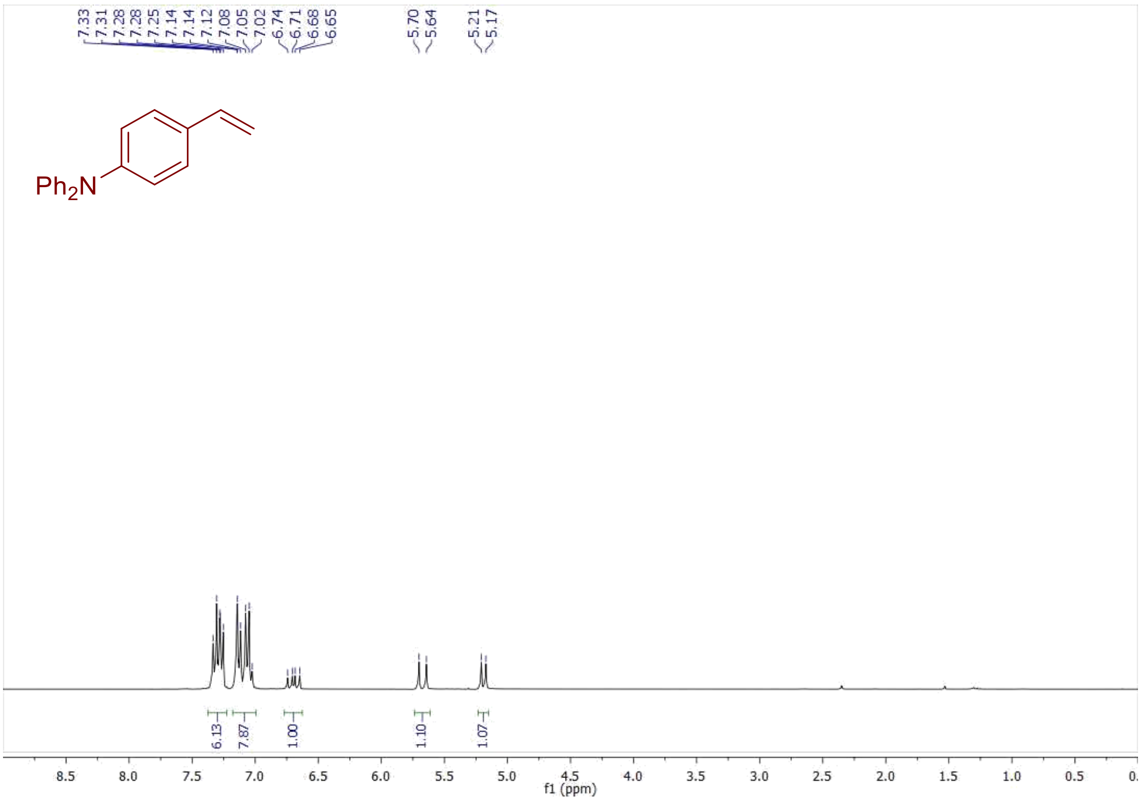

1-(Methylsulfonyl)-4-vinylbenzene (S2c)

^1H NMR (300 MHz, $CDCl_3$)

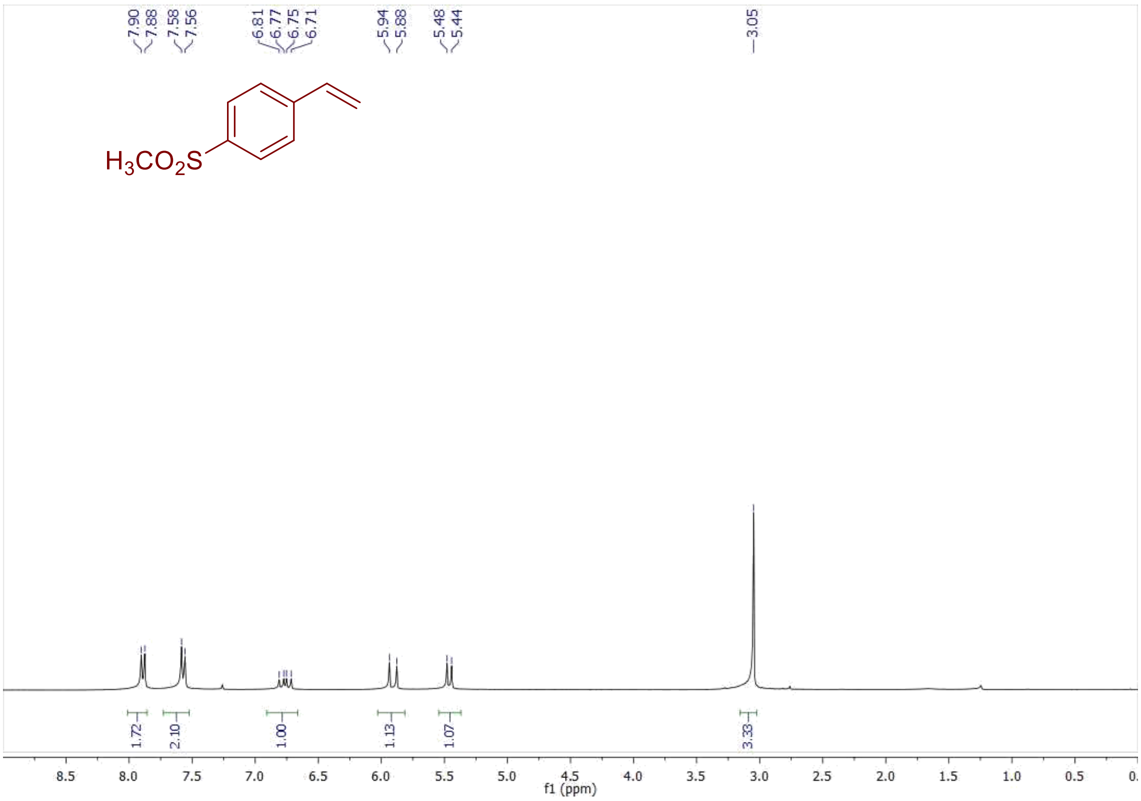

2-(2-Vinylphenyl)-2,3-dihydro-1H-naphtho[1,8-de][1,3,2]diazaborinine (S2d)

^{1}H NMR (400 MHz, $CDCl_3$)

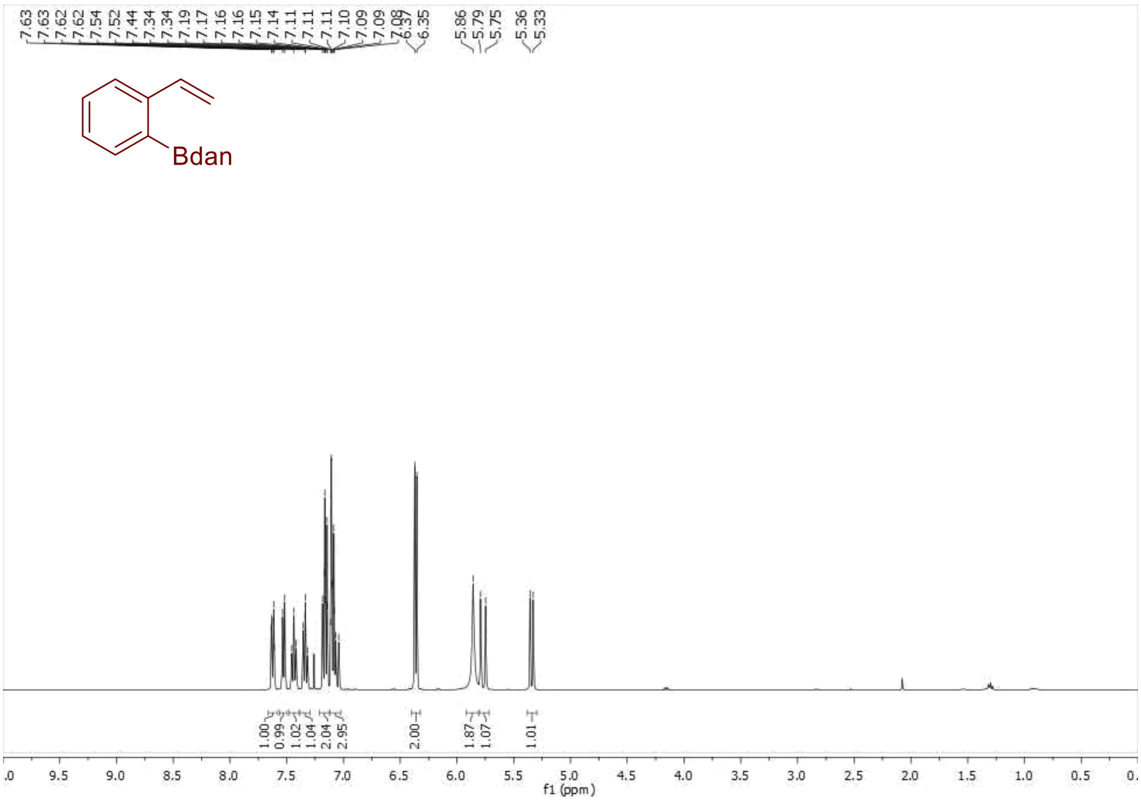

$^{13}C\{^{1}H\}$ NMR (101 MHz, $CDCl_3$)

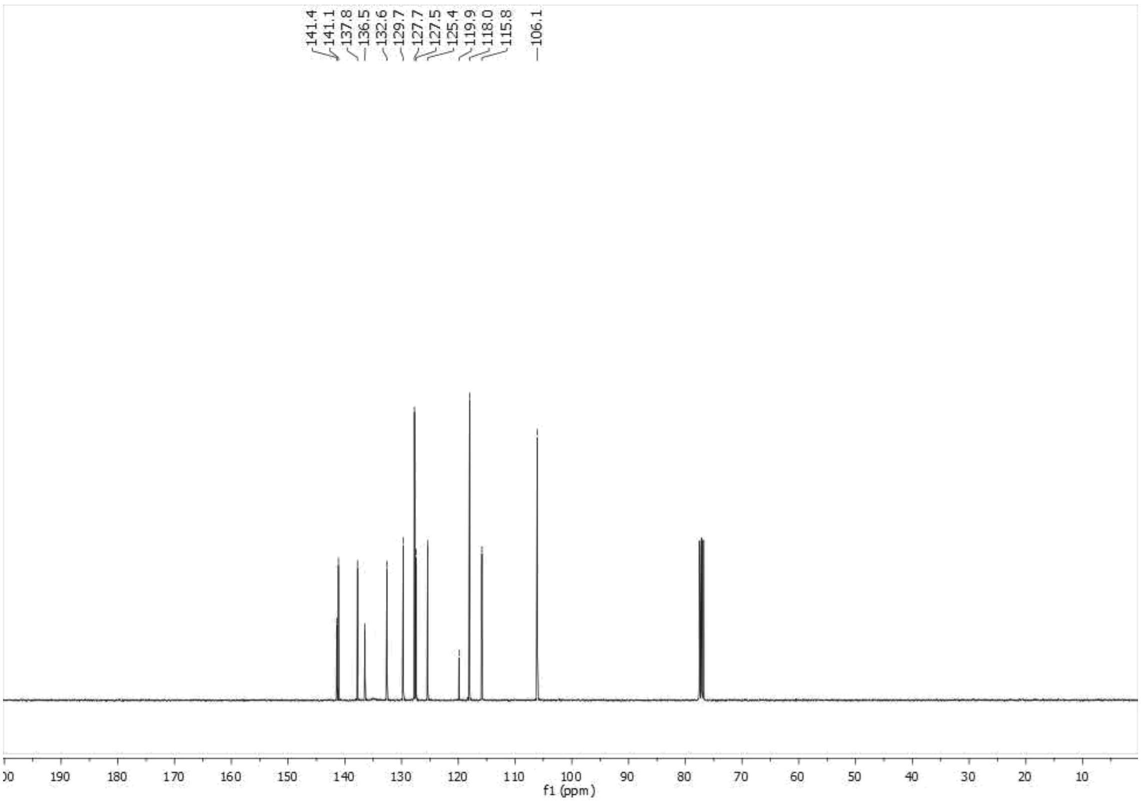

5-Phenyl-3-(trifluoromethyl)-4,5-dihydro-3H-pyrazole (3a)

^{1}H NMR (500 MHz, $CDCl_3$)

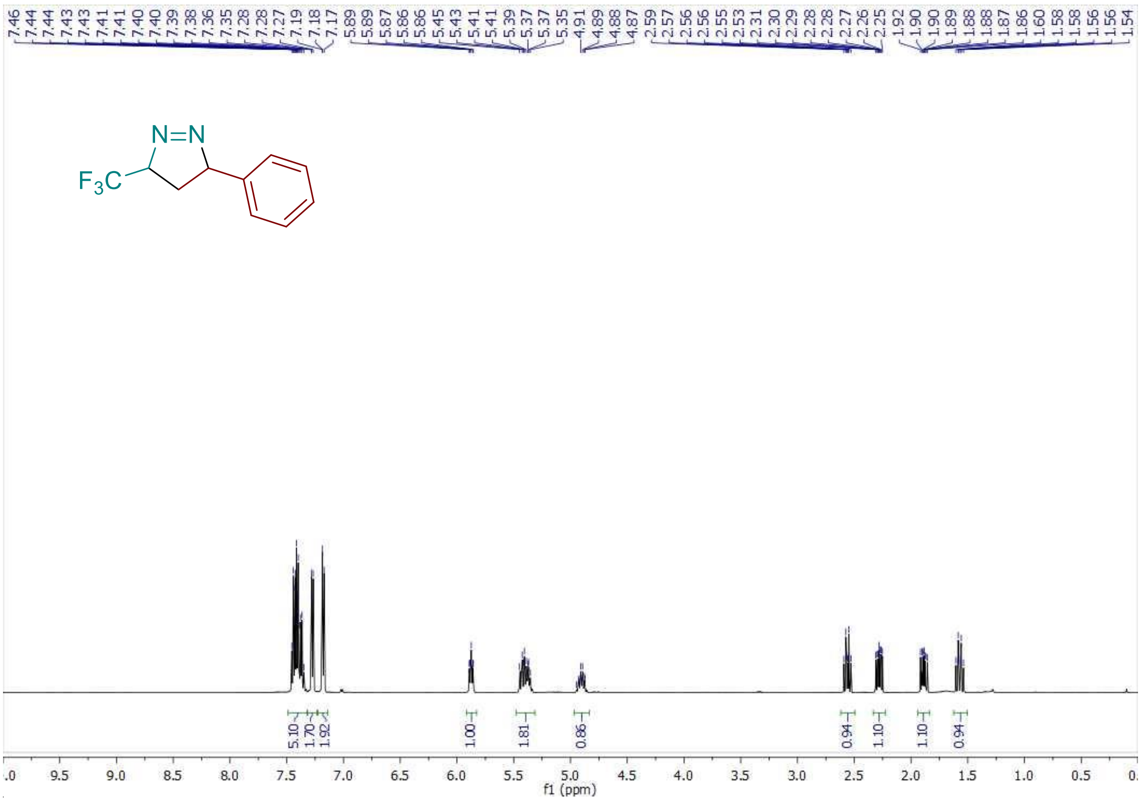

^{13}C{^{1}H} NMR (101 MHz, $CDCl_3$)

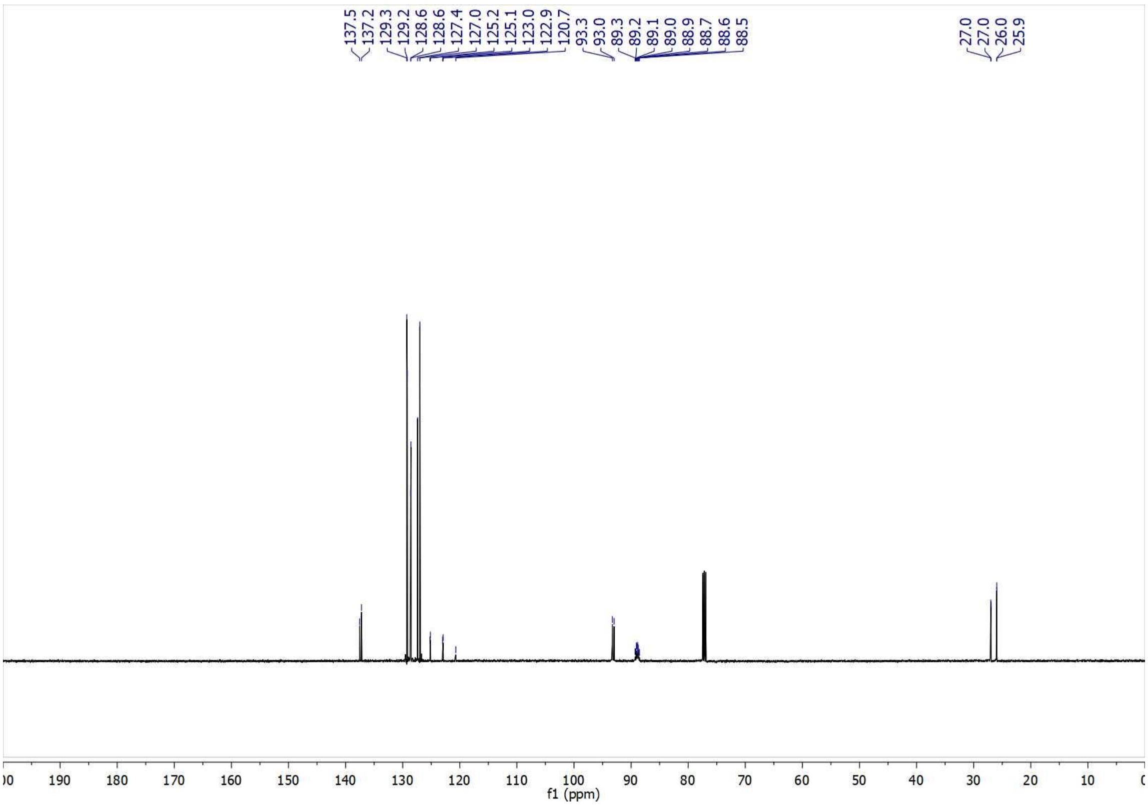

^{19}F NMR (376 MHz, $CDCl_3$)

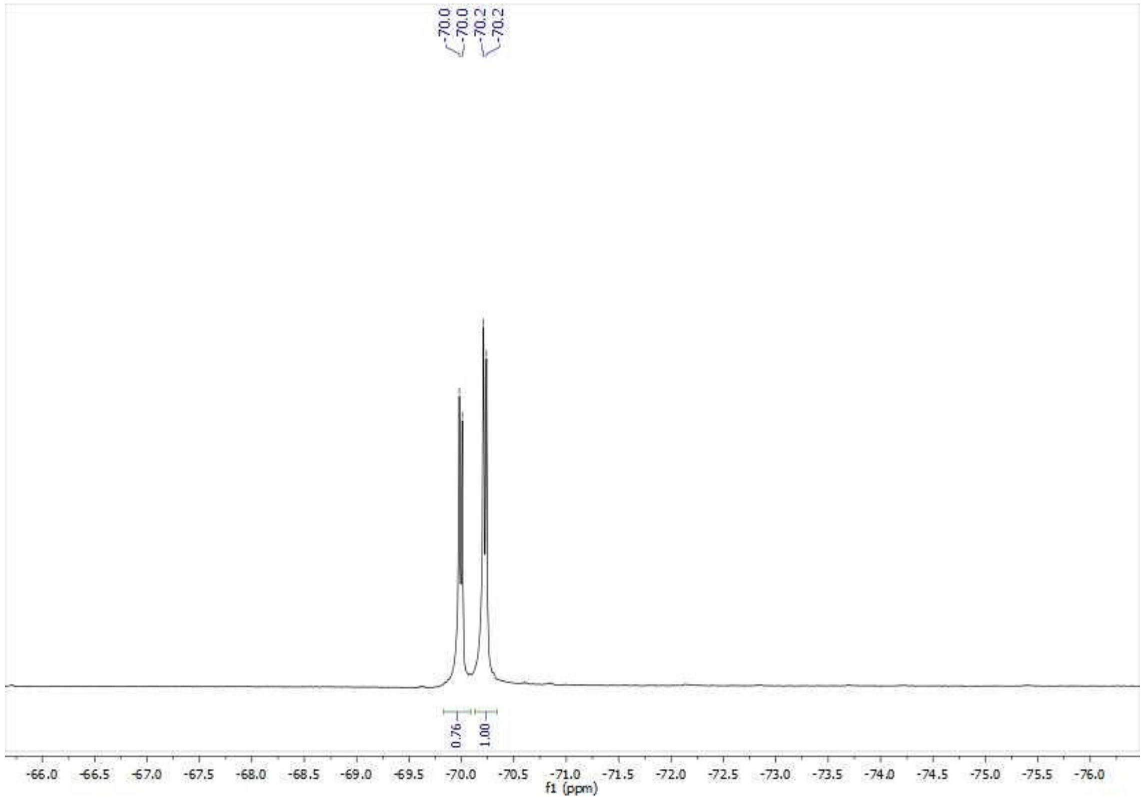

COSY ($CDCl_3$)

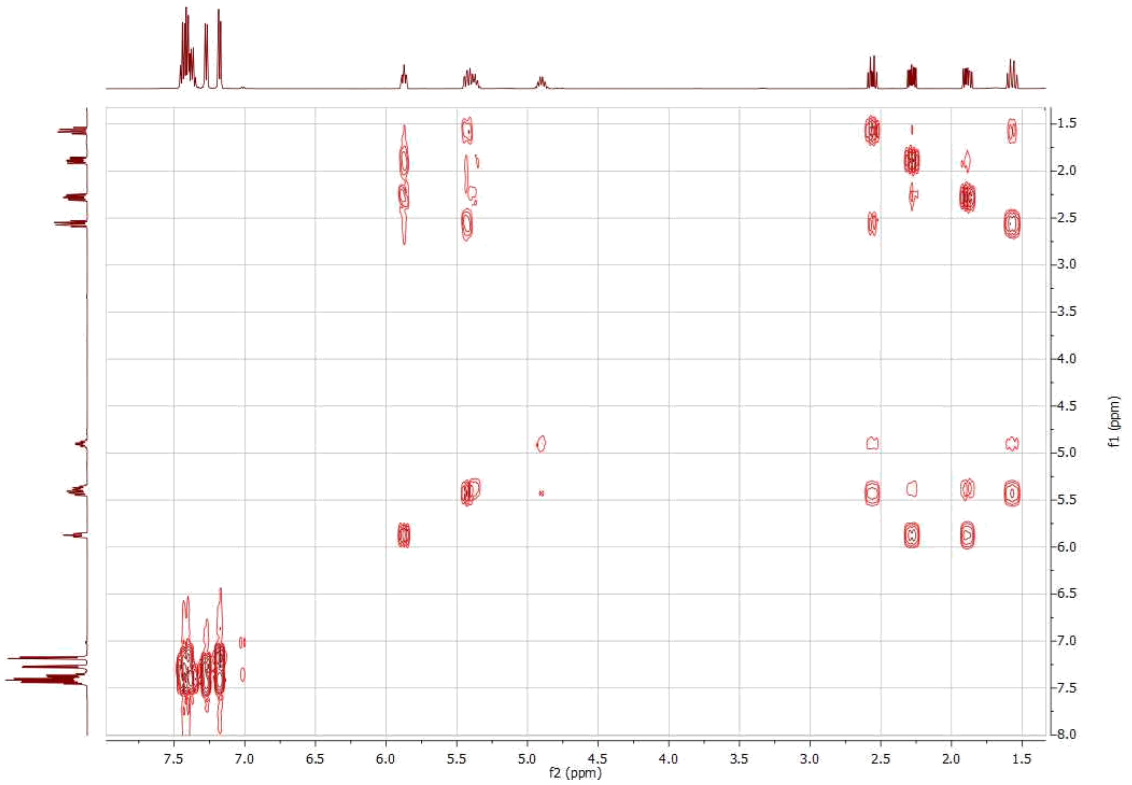

HSQC ($CDCl_3$)

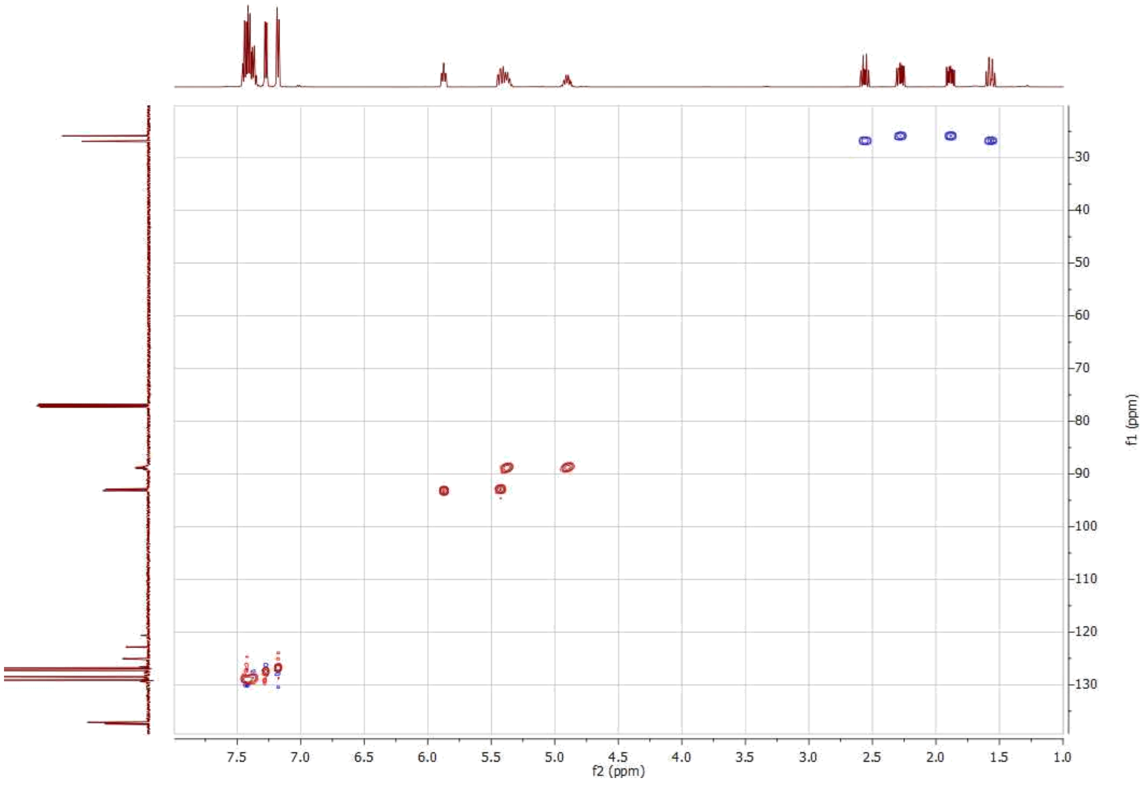

5-Phenyl-3-(trifluoromethyl)-4,5-dihydro-1H-pyrazole (4a)

^{1}H NMR (400 MHz, $CDCl_3$)

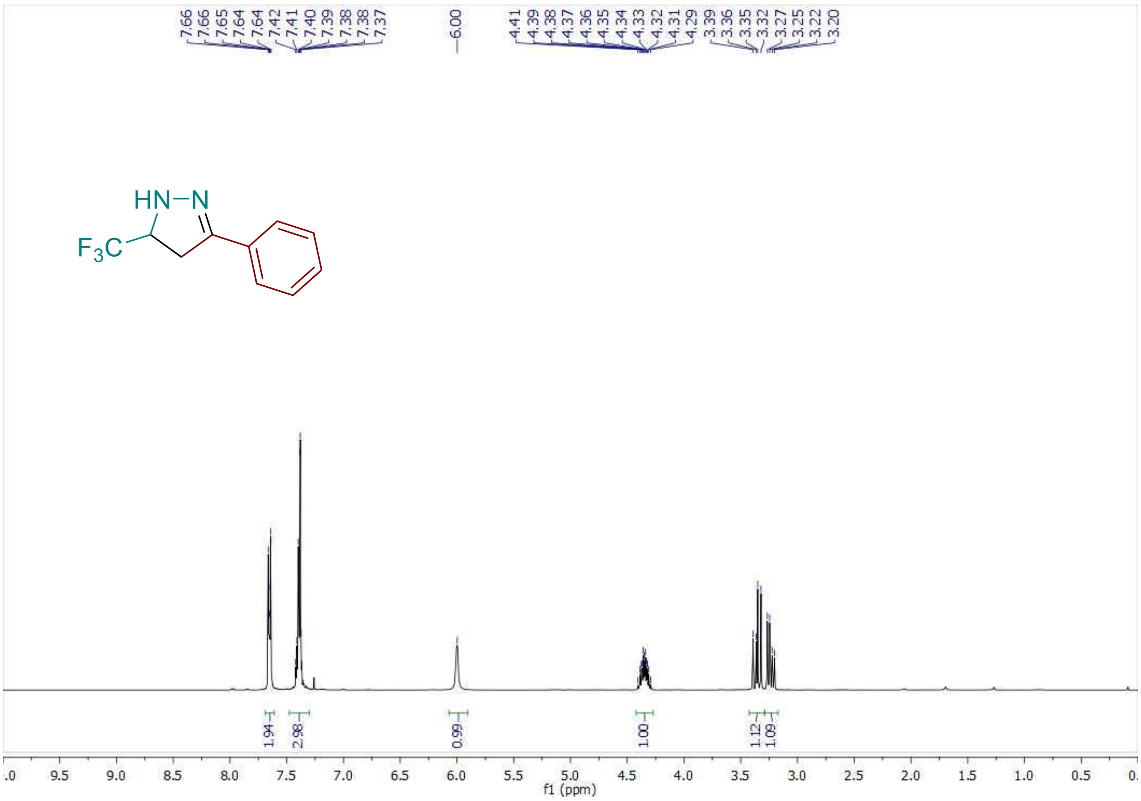

^{13}C{^{1}H} NMR (101 MHz, $CDCl_3$)

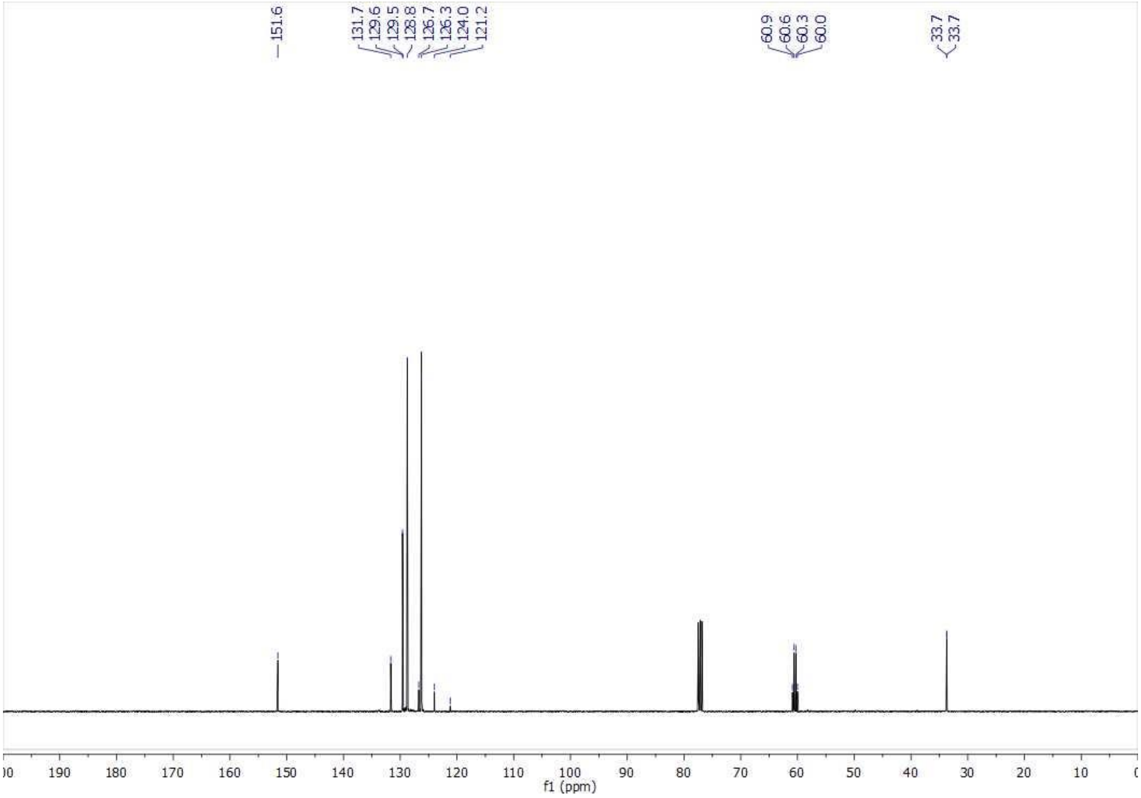

^{19}F NMR (376 MHz, $CDCl_3$)

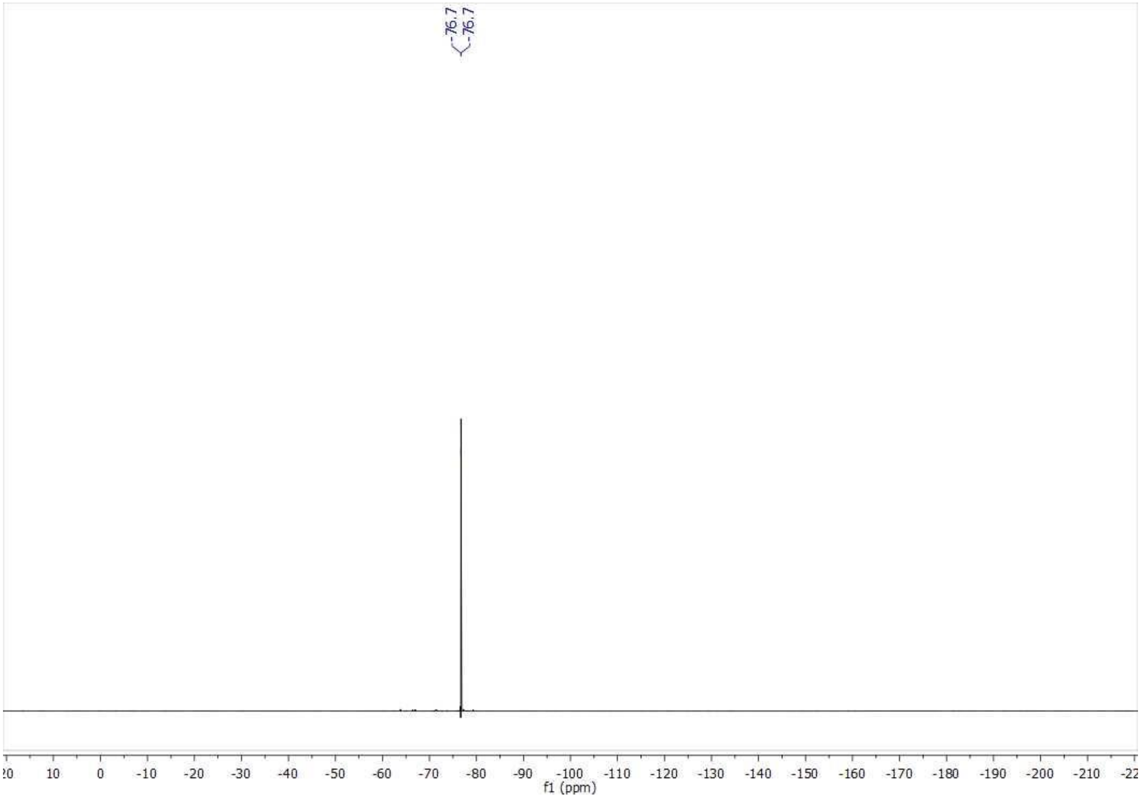

5-Phenyl-3-(trifluoromethyl)-1*H*-pyrazole (5a)

^{1}H NMR (400 MHz, $CDCl_3$)

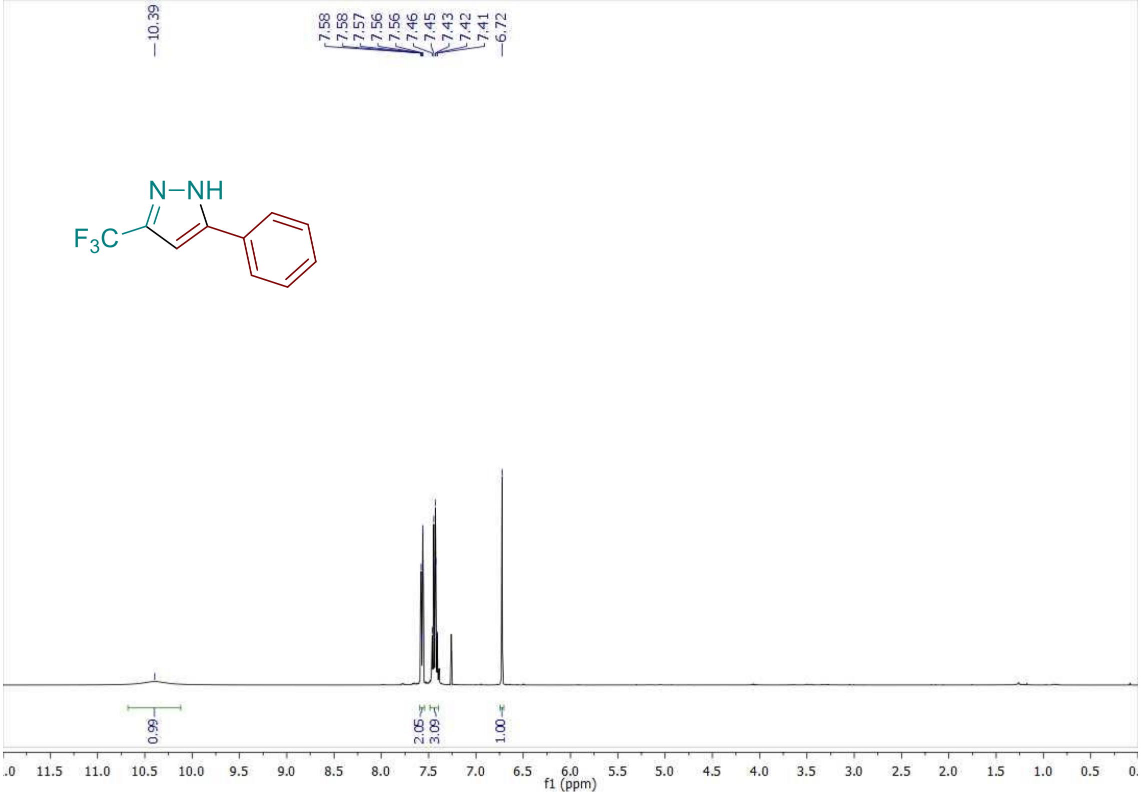

$^{13}C\{^{1}H\}$ NMR (101 MHz, $CDCl_3$)

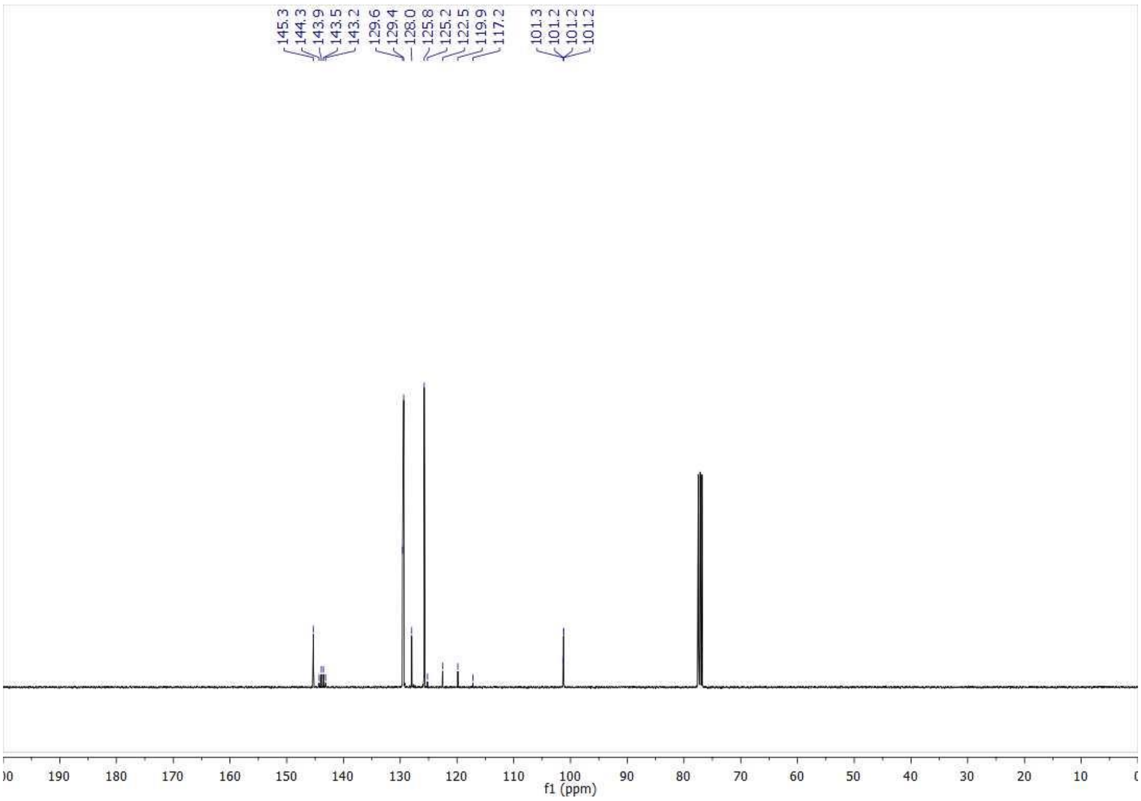

^{19}F NMR (376 MHz, $CDCl_3$)

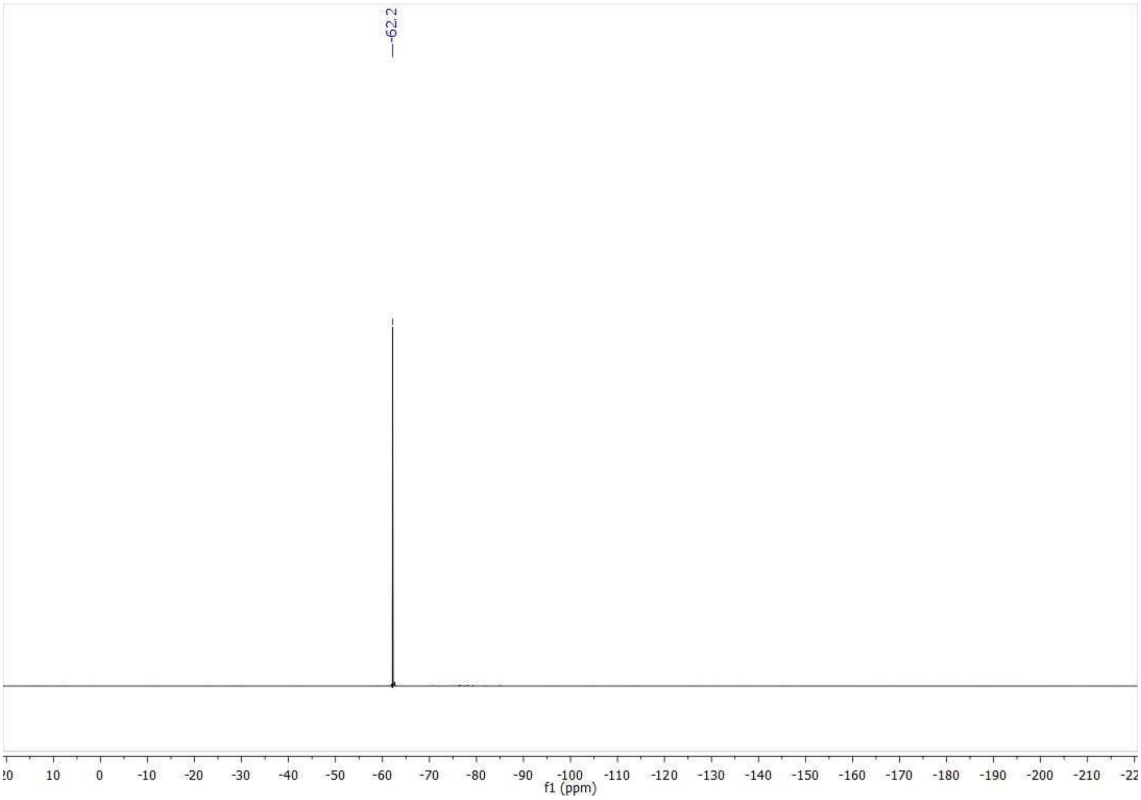

5-(*o*-Tolyl)-3-(trifluoromethyl)-1*H*-pyrazole (5b)

^{1}H NMR (400 MHz, $CDCl_3$)

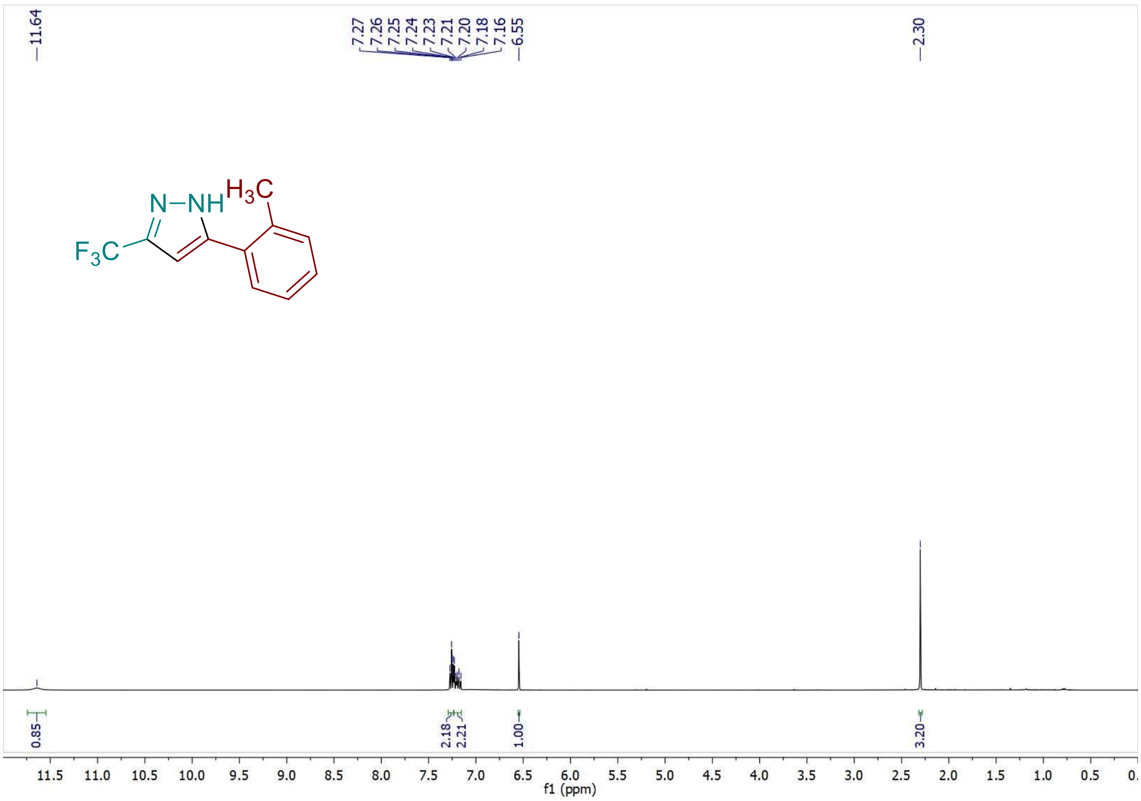

$^{13}C\{^{1}H\}$ NMR (101 MHz, $CDCl_3$)

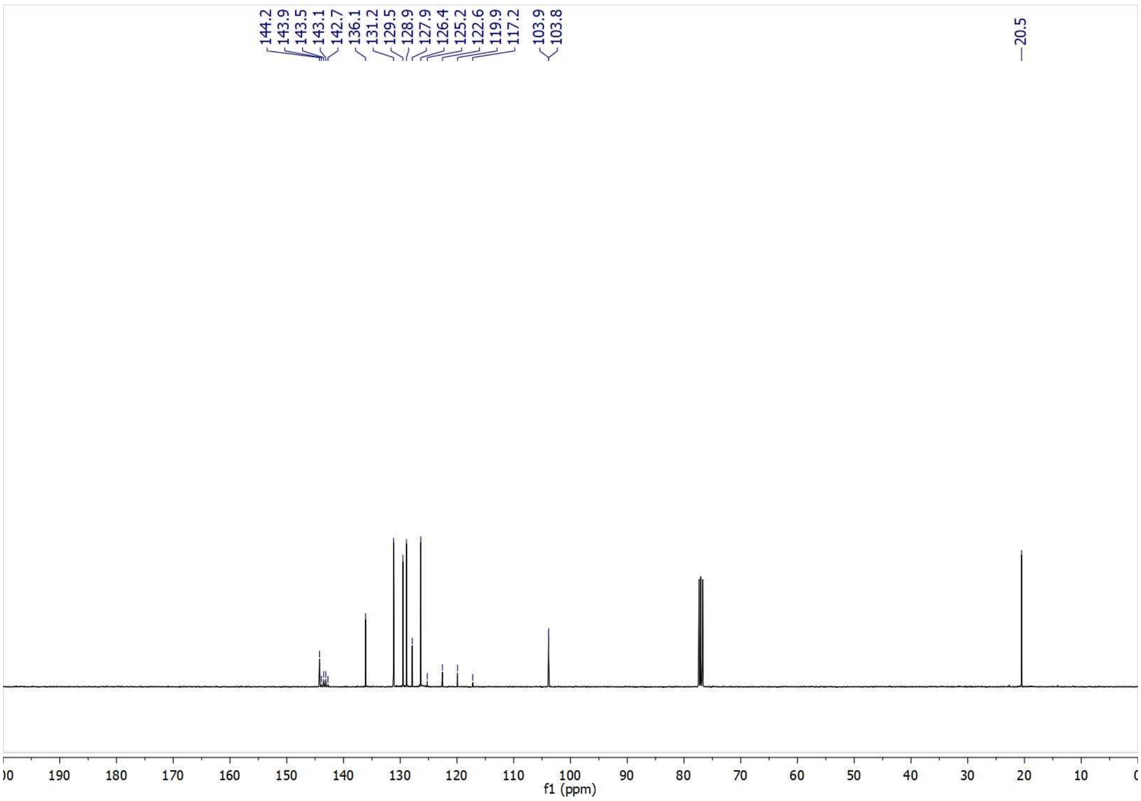

^{19}F NMR (376 MHz, $CDCl_3$)

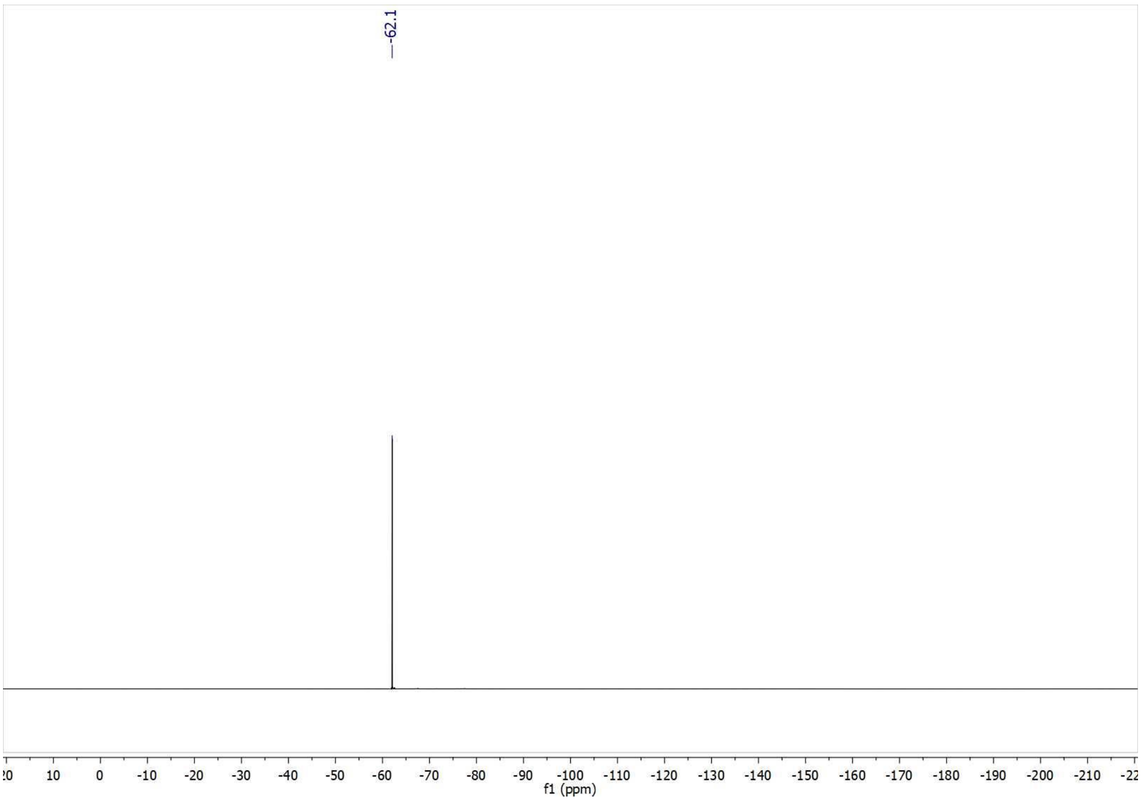

5-(*o*-Chlorophenyl)-3-(trifluoromethyl)-1*H*-pyrazole (5c)

^{1}H NMR (400 MHz, $CDCl_3$)

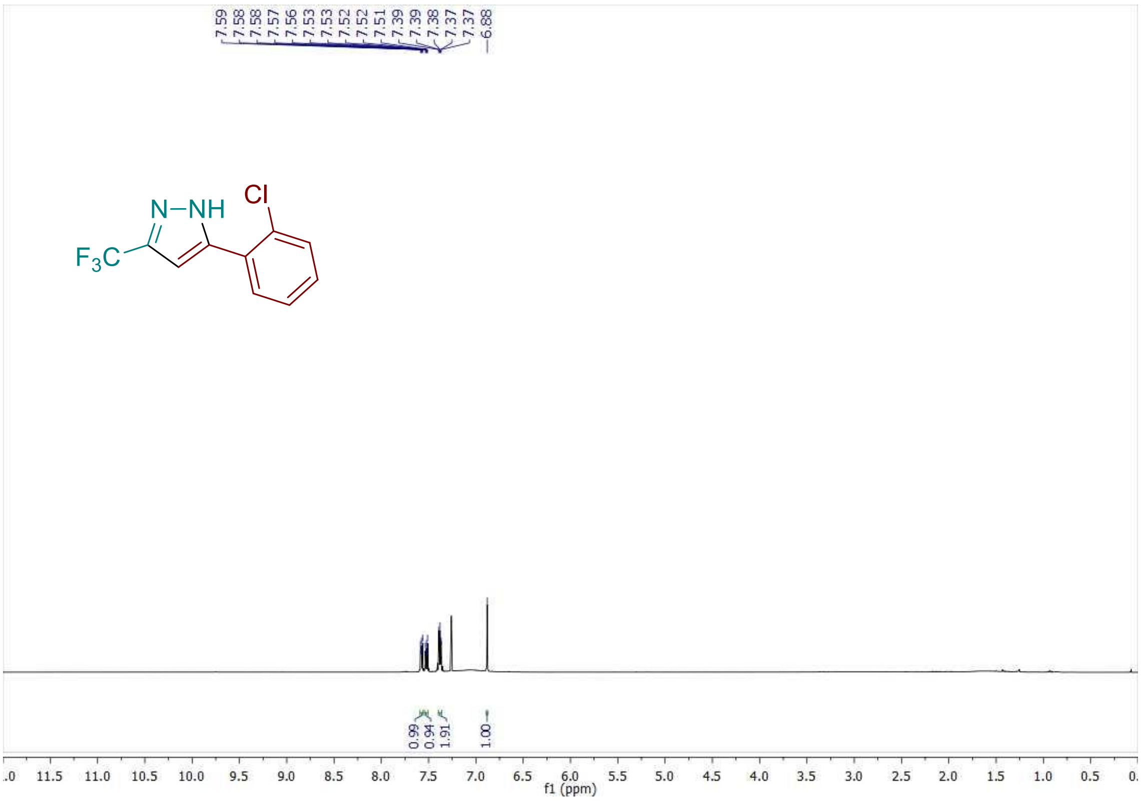

^{13}C{^{1}H} NMR (101 MHz, $CDCl_3$)

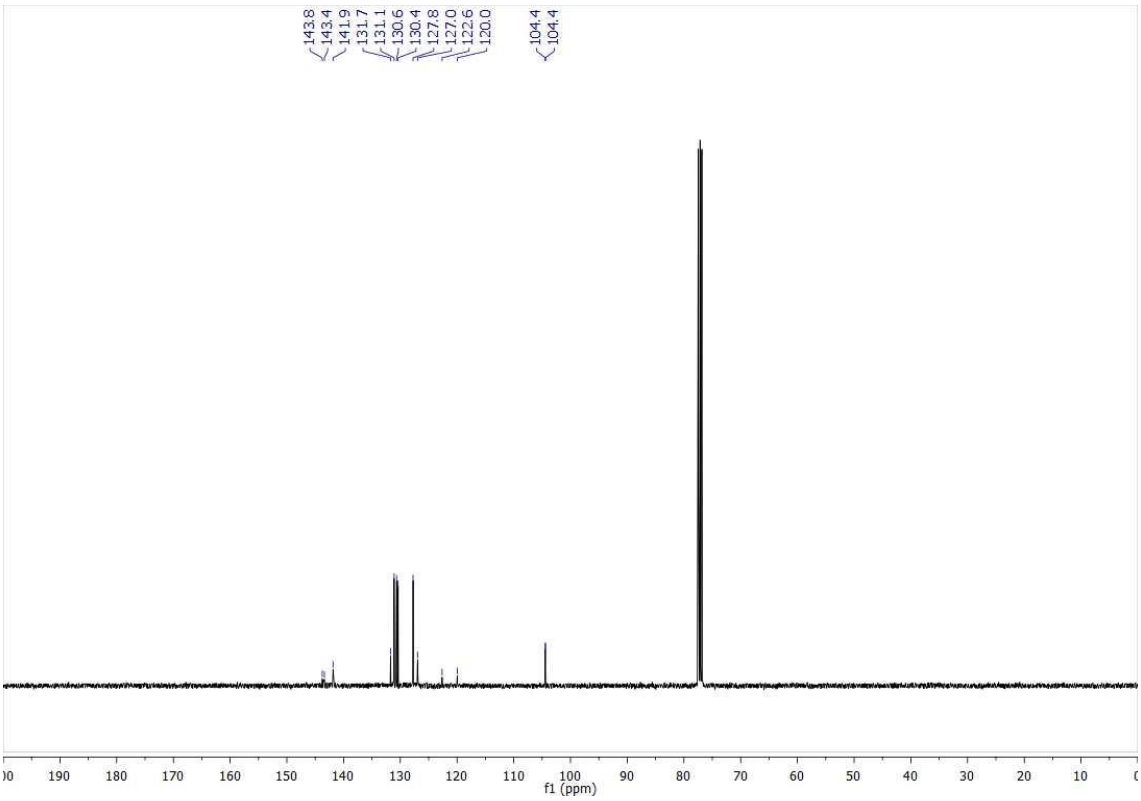

^{19}F NMR (376 MHz, $CDCl_3$)

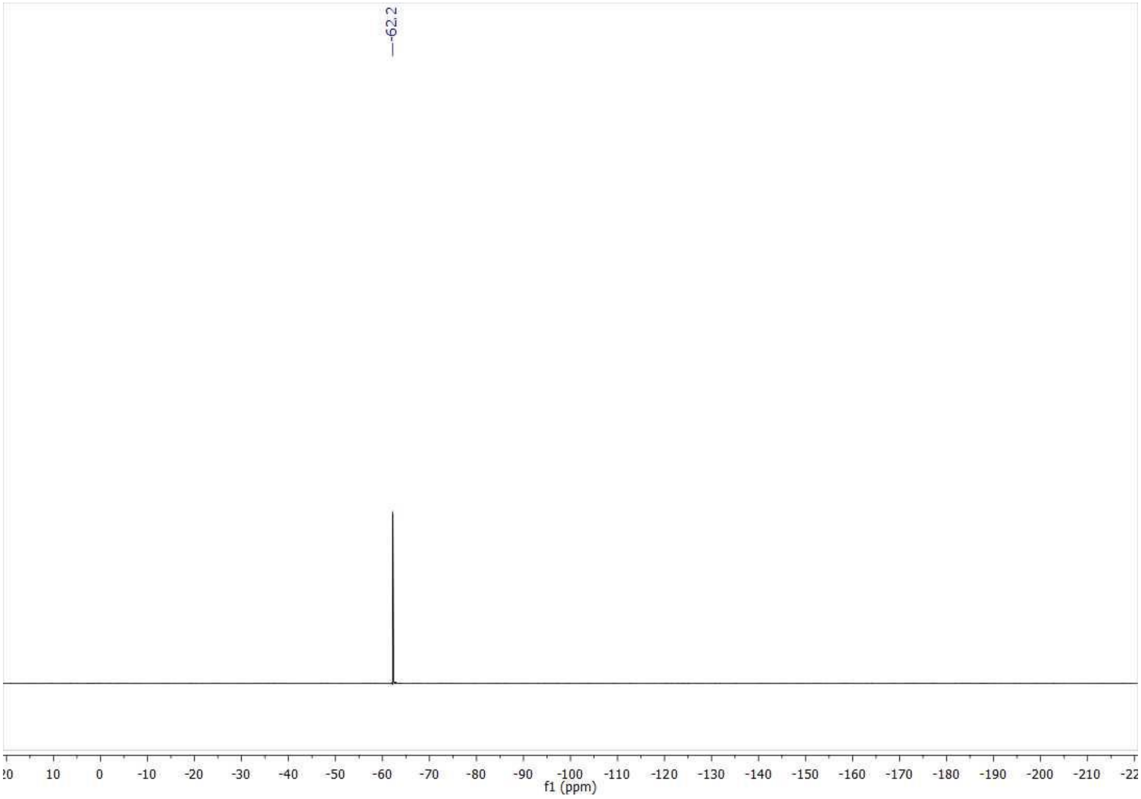

5-(*m*-Tolyl)-3-(trifluoromethyl)-1*H*-pyrazole (5d)

^{1}H NMR (400 MHz, $CDCl_3$)

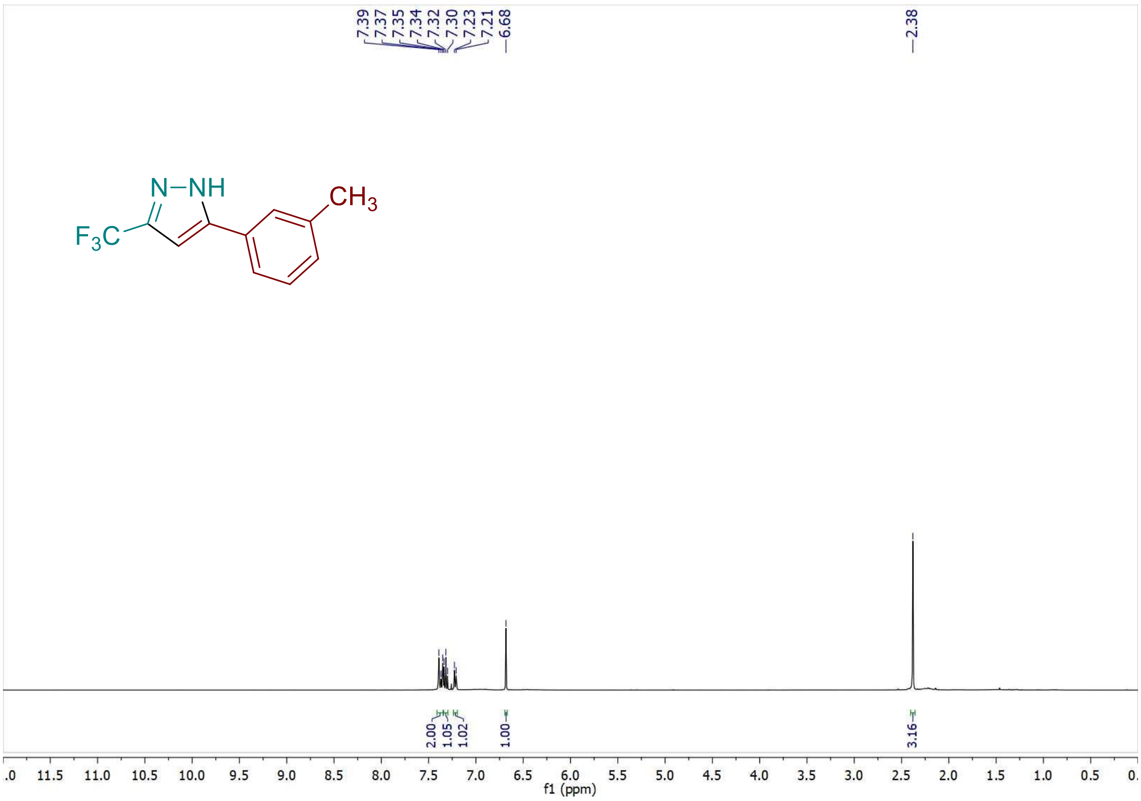

^{13}C{^{1}H} NMR (101 MHz, $CDCl_3$)

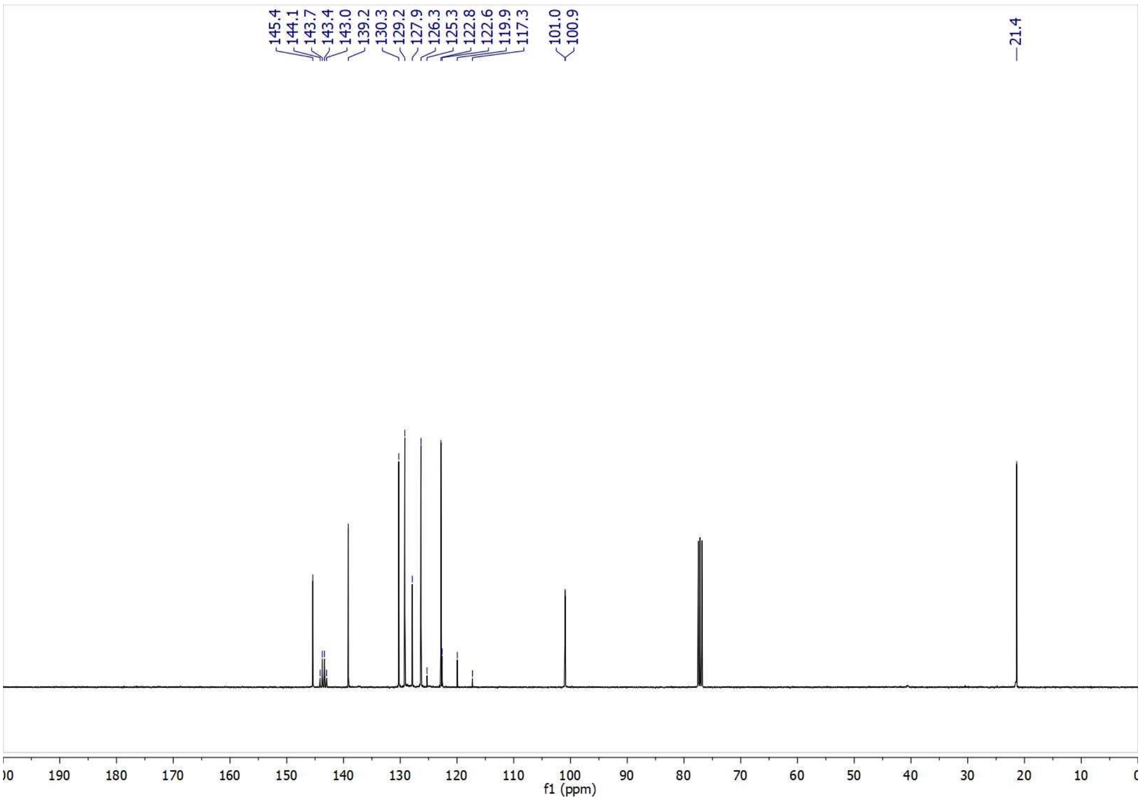

^{19}F NMR (376 MHz, $CDCl_3$)

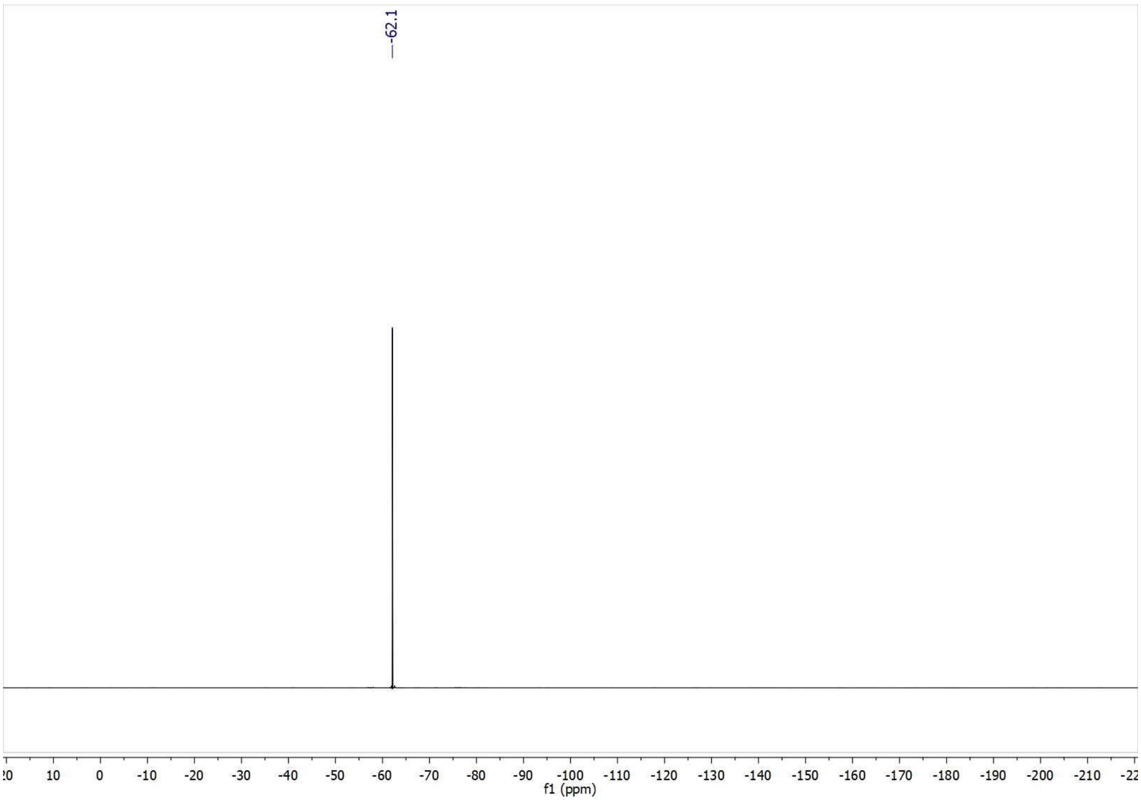

5-(*m*-Bromophenyl)-3-(trifluoromethyl)-1*H*-pyrazole (5e)

^{1}H NMR (400 MHz, $CDCl_3$)

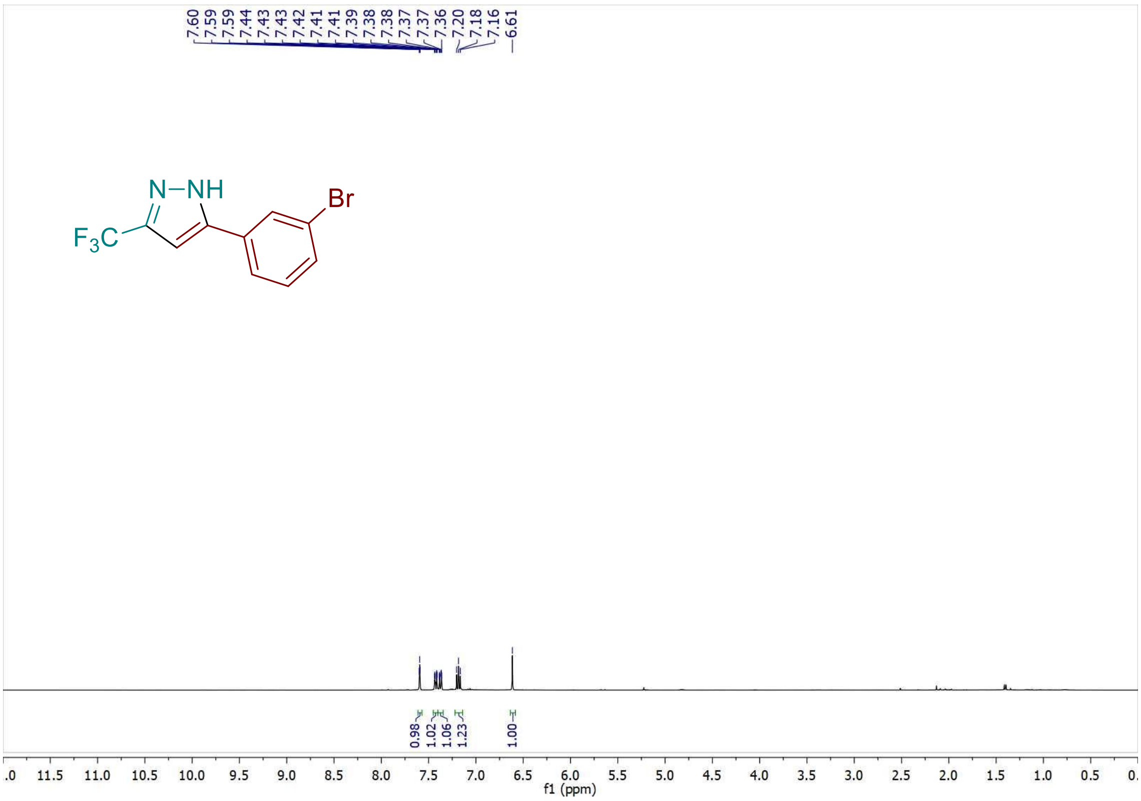

^{13}C{^{1}H} NMR (101 MHz, $CDCl_3$)

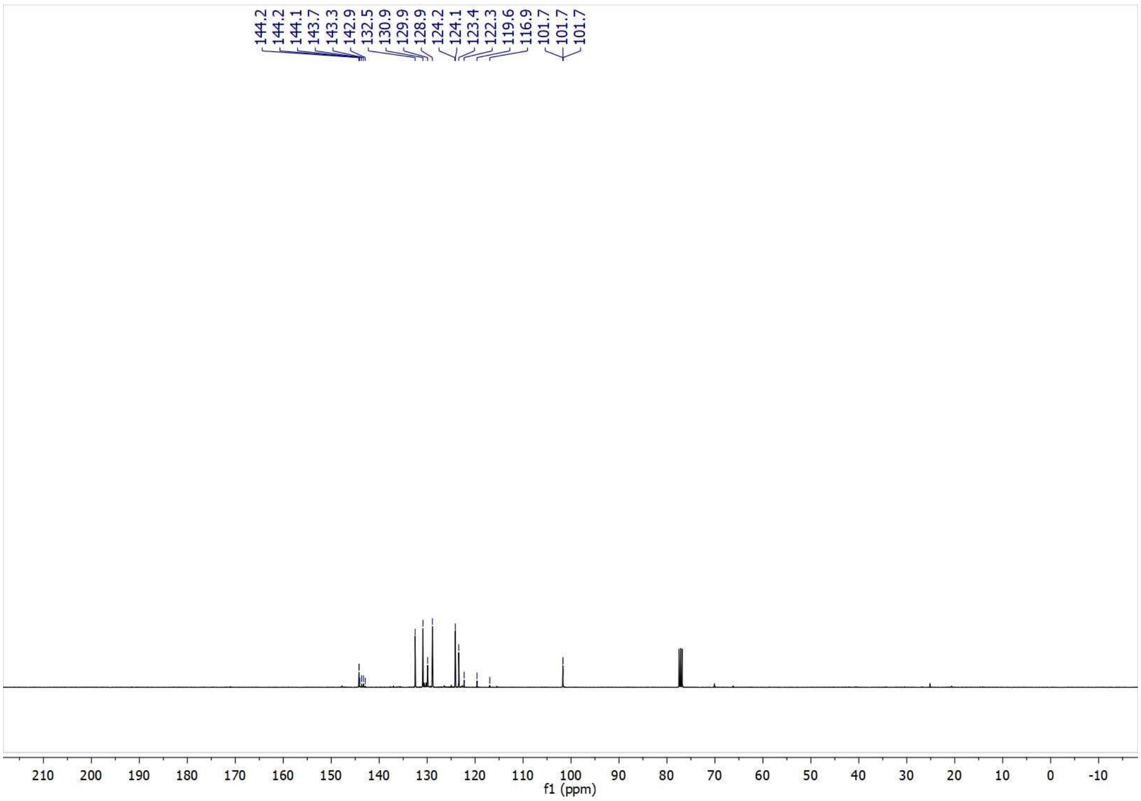

^{19}F NMR (376 MHz, $CDCl_3$)

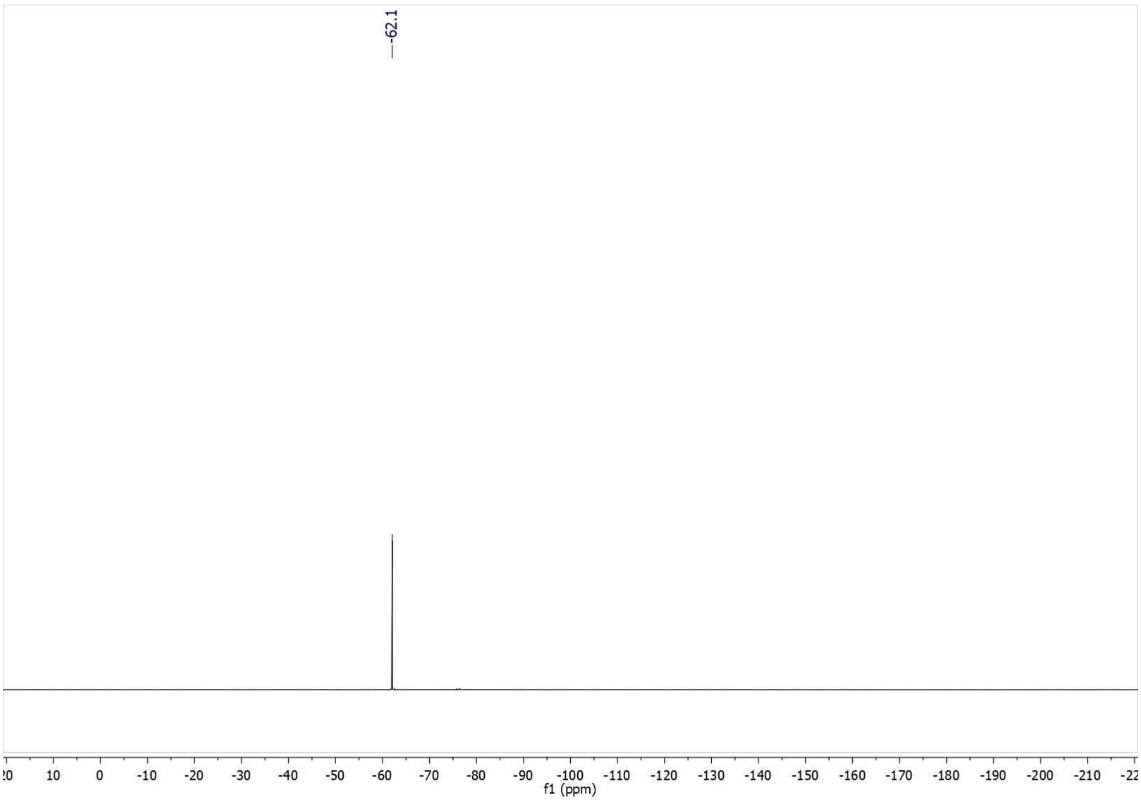

5-(*m*-Nitrophenyl)-3-(trifluoromethyl)-1*H*-pyrazole (5f)

^{1}H NMR (400 MHz, $CDCl_3$)

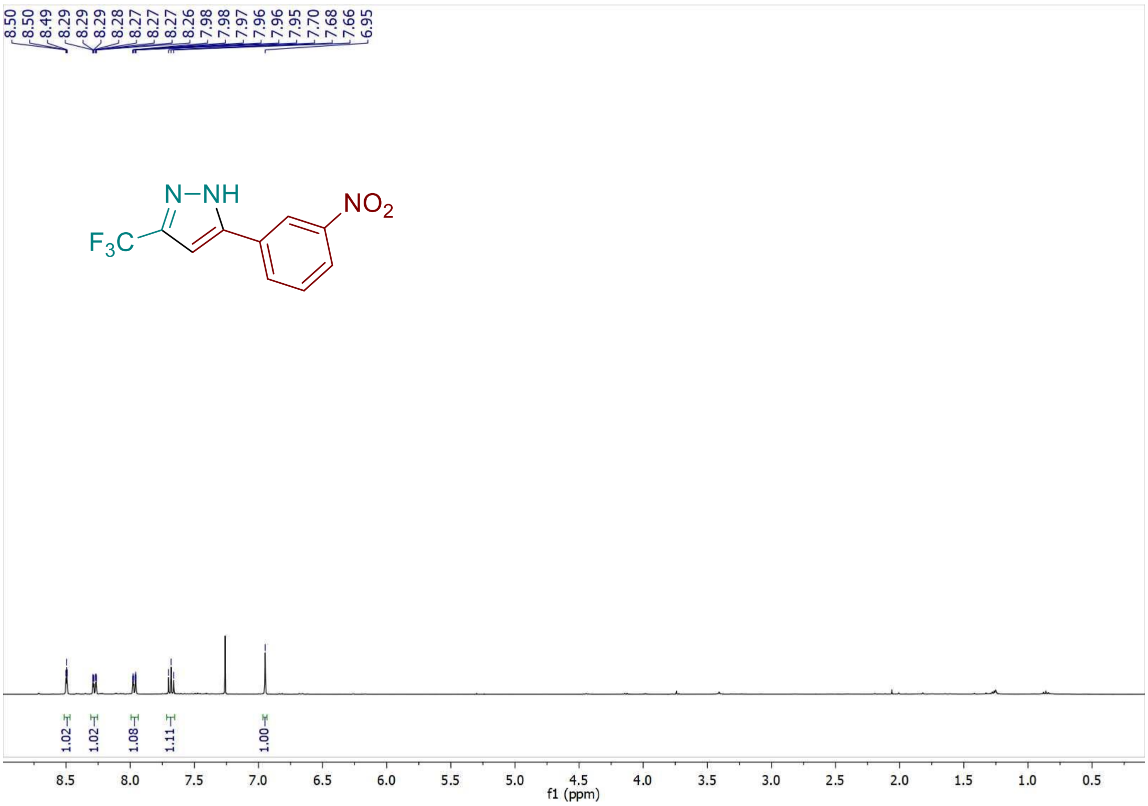

^{13}C{^{1}H} NMR (101 MHz, $CDCl_3$)

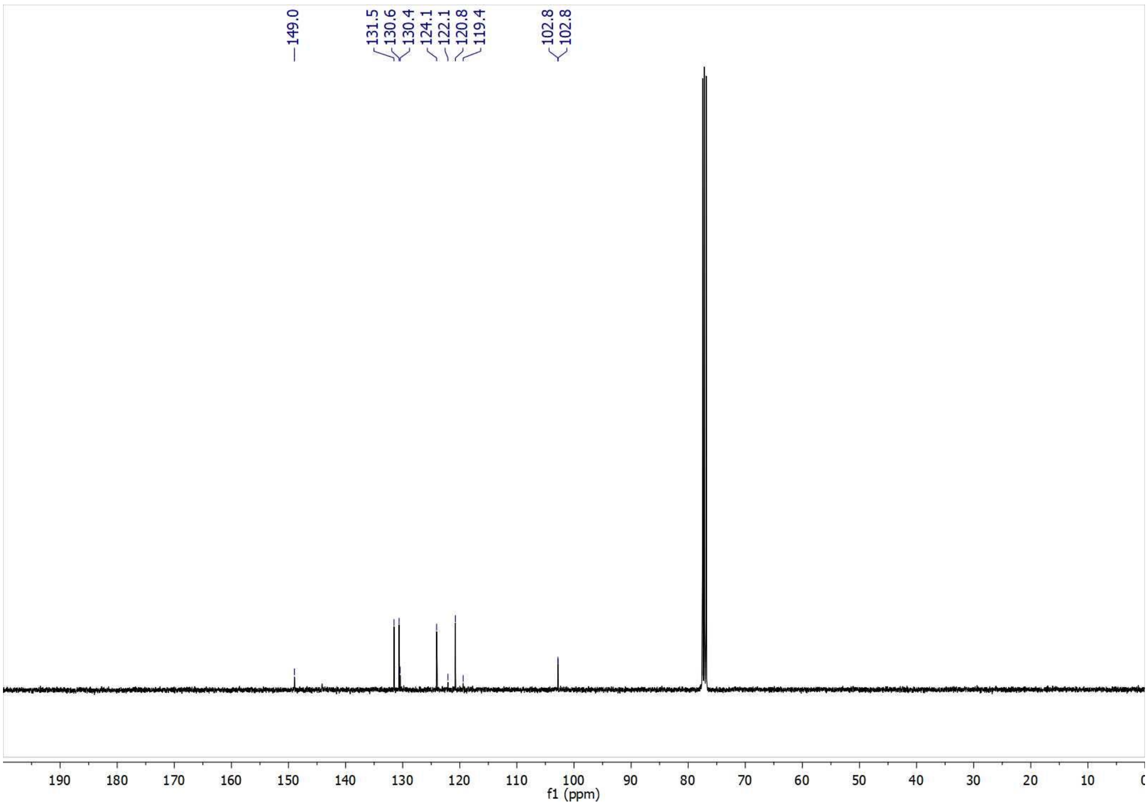

^{19}F NMR (376 MHz, $CDCl_3$)

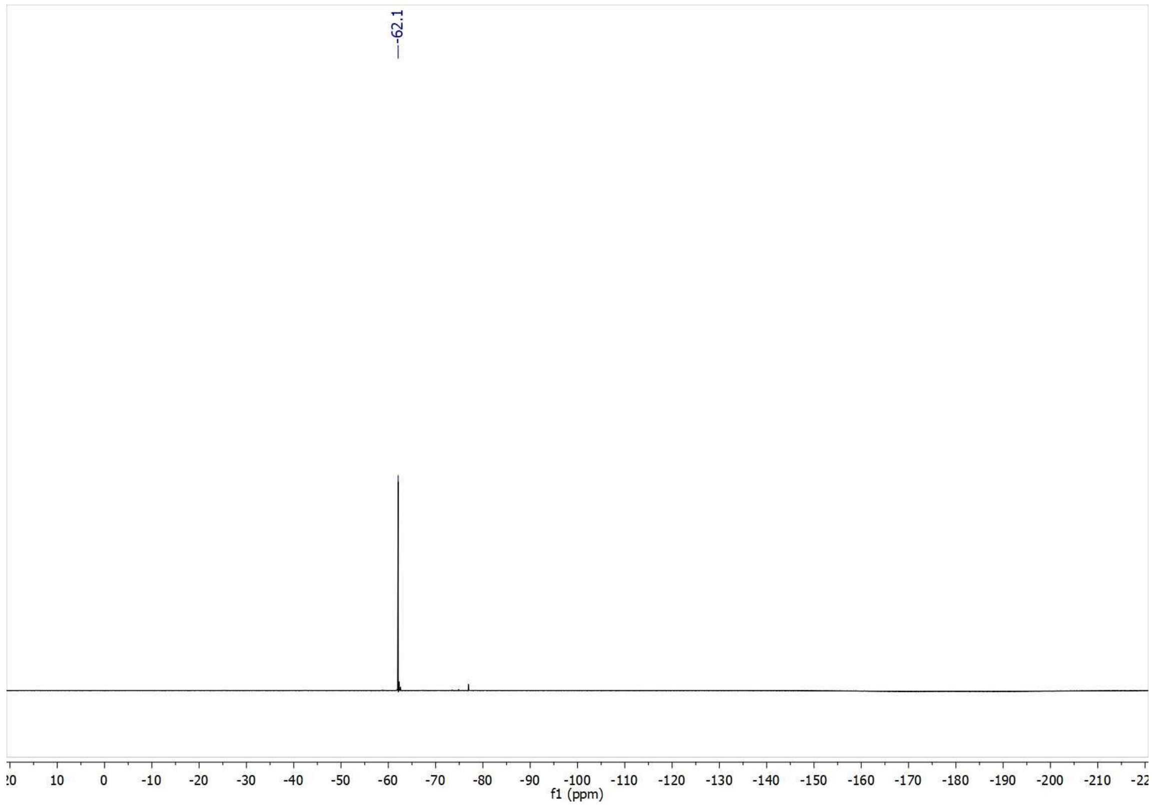

5-(*p*-Tolyl)-3-(trifluoromethyl)-1*H*-pyrazole (5g)

^{1}H NMR (400 MHz, $CDCl_3$)

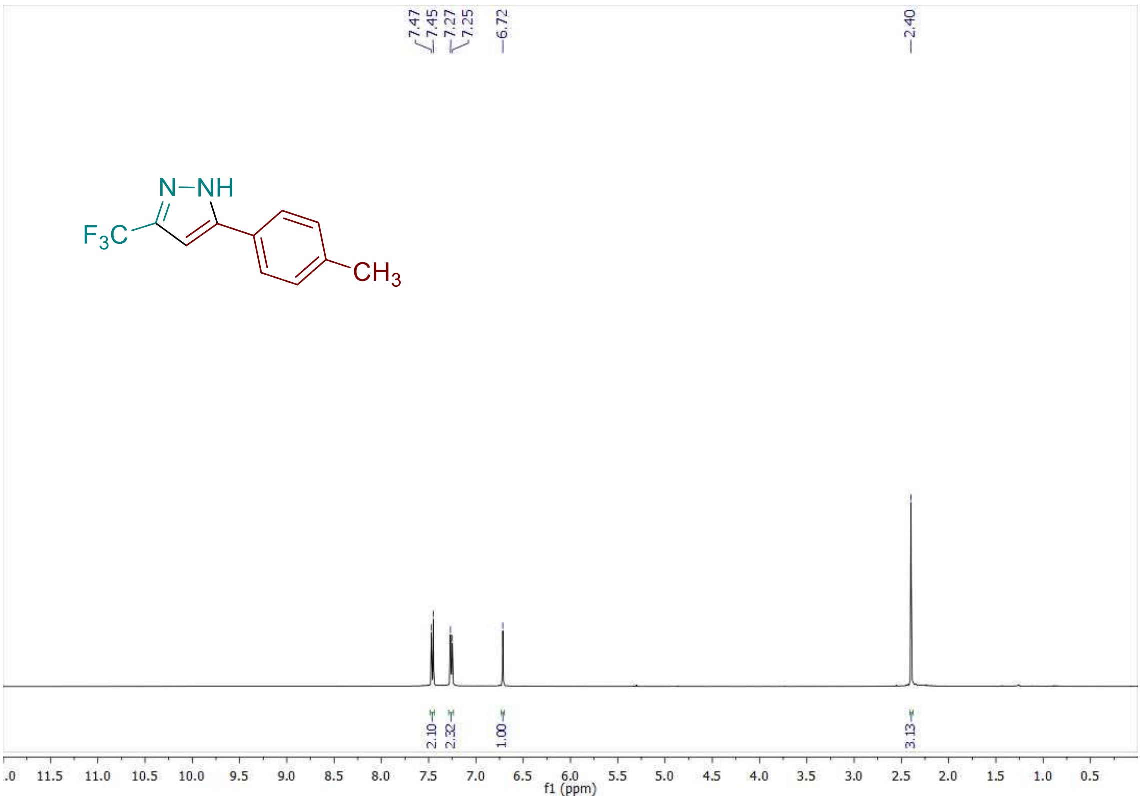

$^{13}C\{^{1}H\}$ NMR (101 MHz, $CDCl_3$)

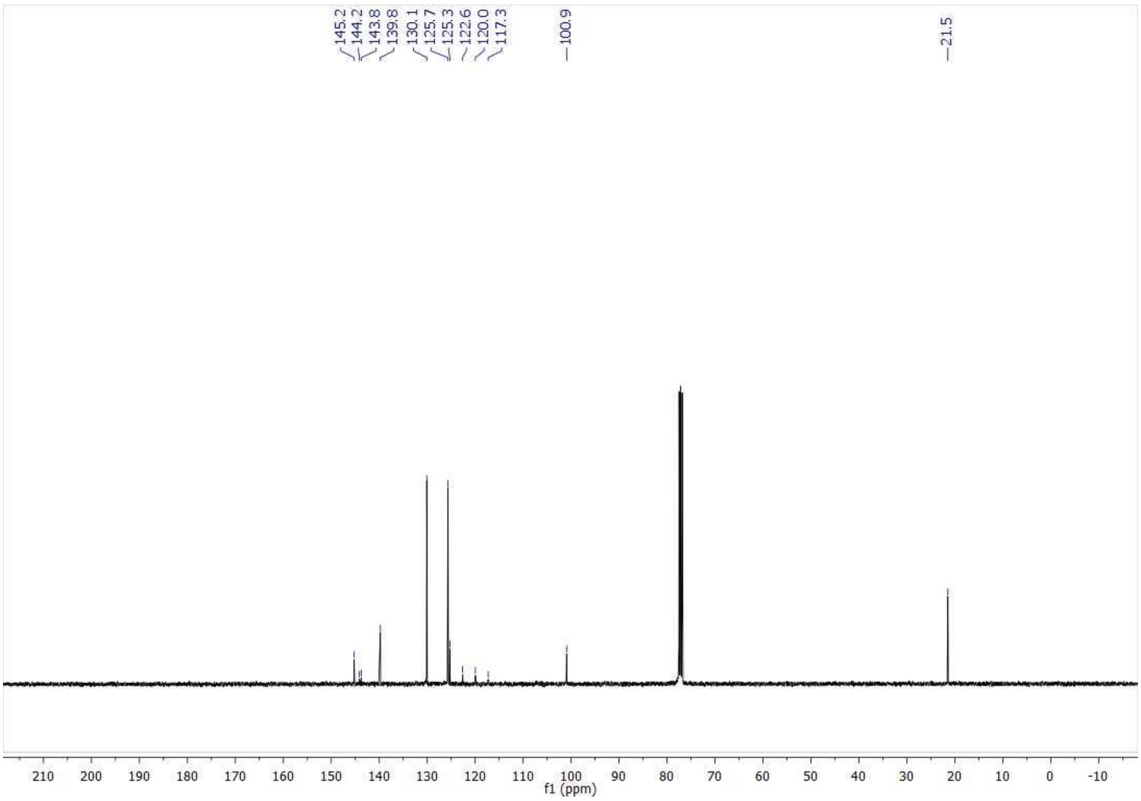

^{19}F NMR (376 MHz, $CDCl_3$)

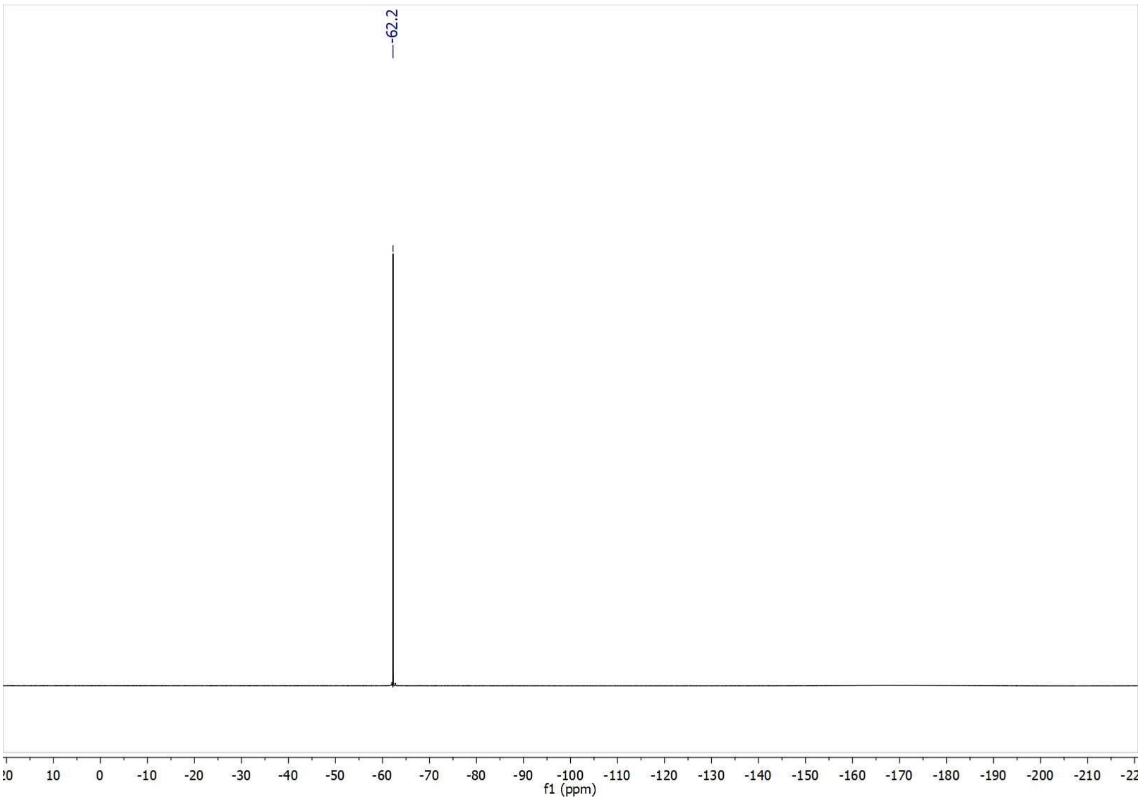

5-(*p*-(*Tert*butyl)phenyl)-3-(trifluoromethyl)-1*H*-pyrazole (5h)

^{1}H NMR (400 MHz, $CDCl_3$)

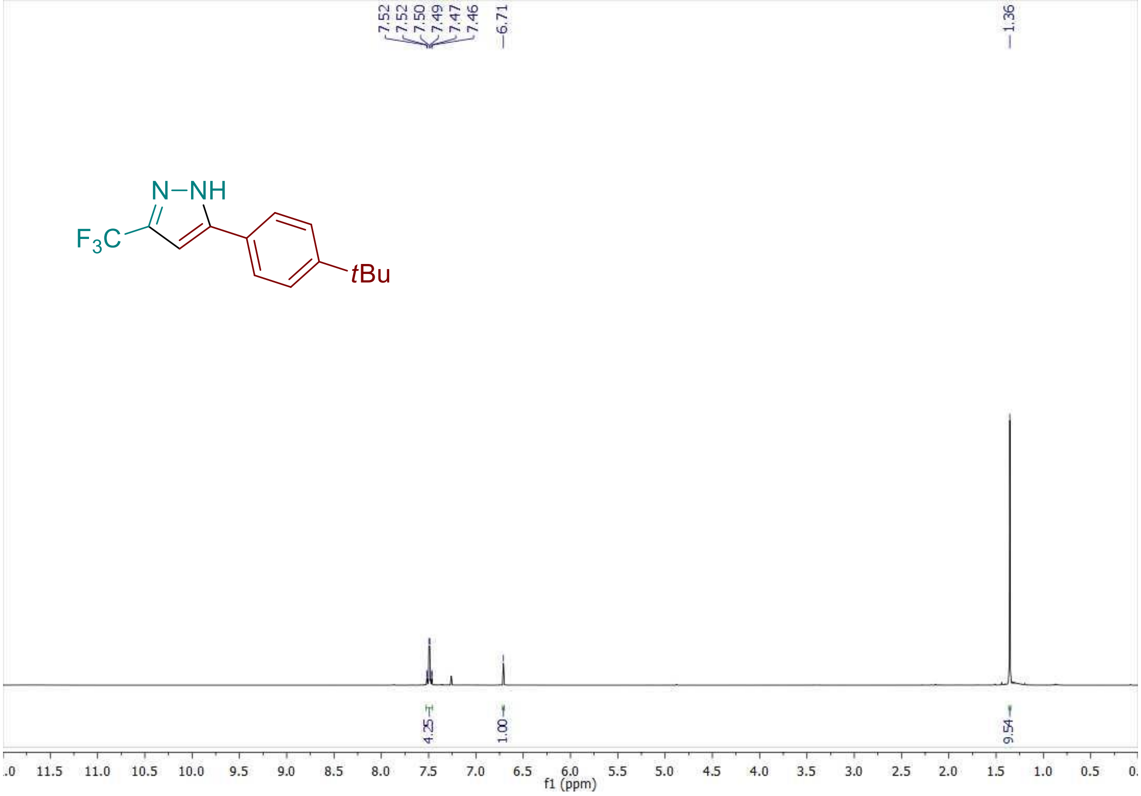

$^{13}C\{^{1}H\}$ NMR (101 MHz, $CDCl_3$)

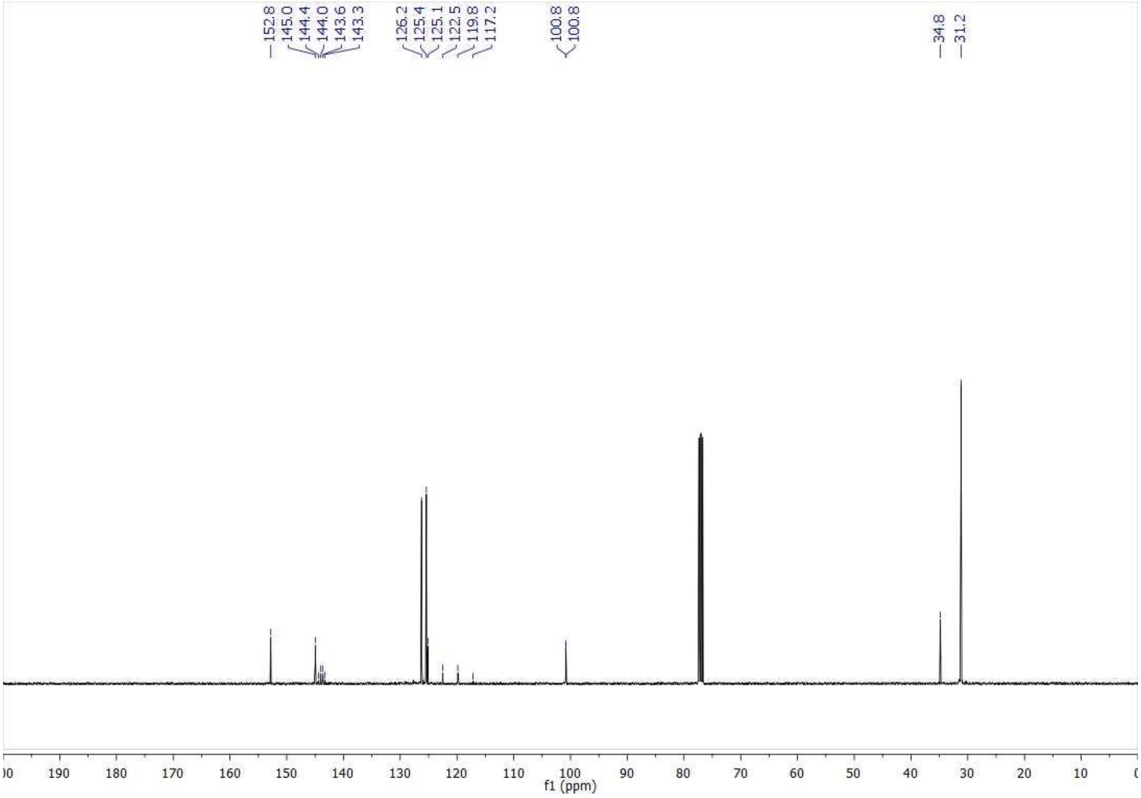

^{19}F NMR (376 MHz, $CDCl_3$)

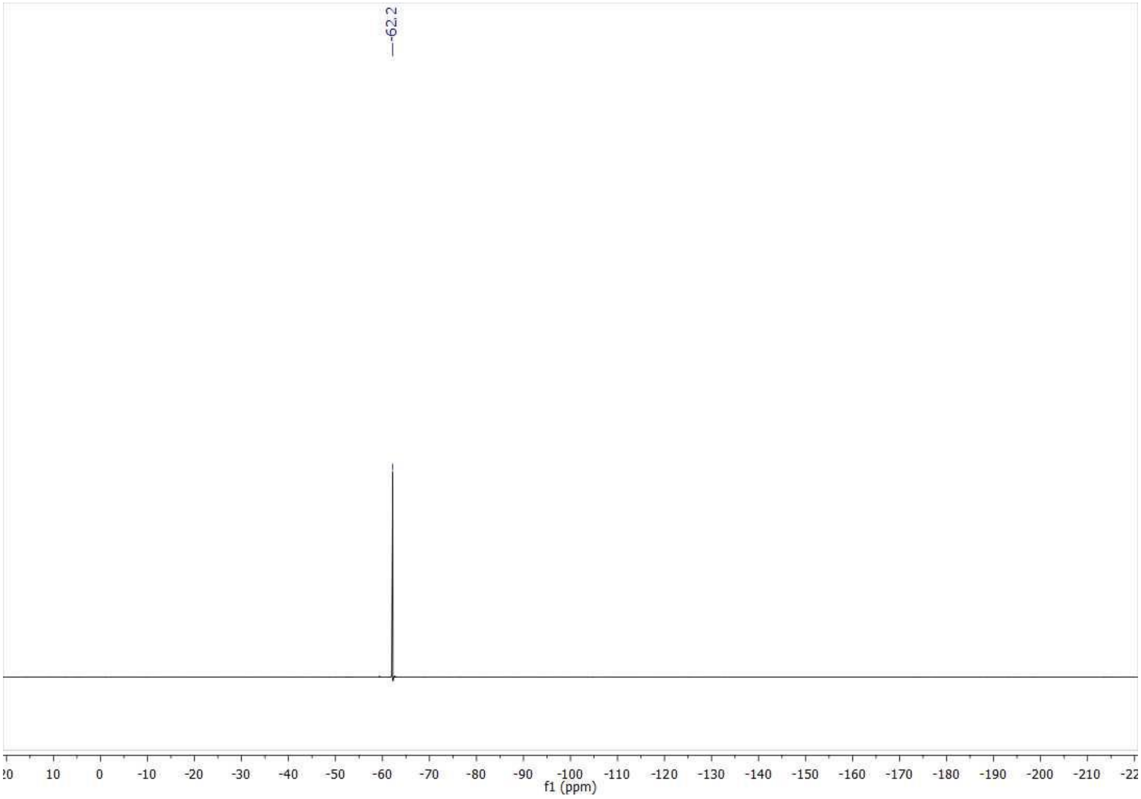

5-([1,1'-biphenyl]-4-yl)-3-(trifluoromethyl)-1*H*-pyrazole (5i)

^{1}H NMR (400 MHz, $CDCl_3$)

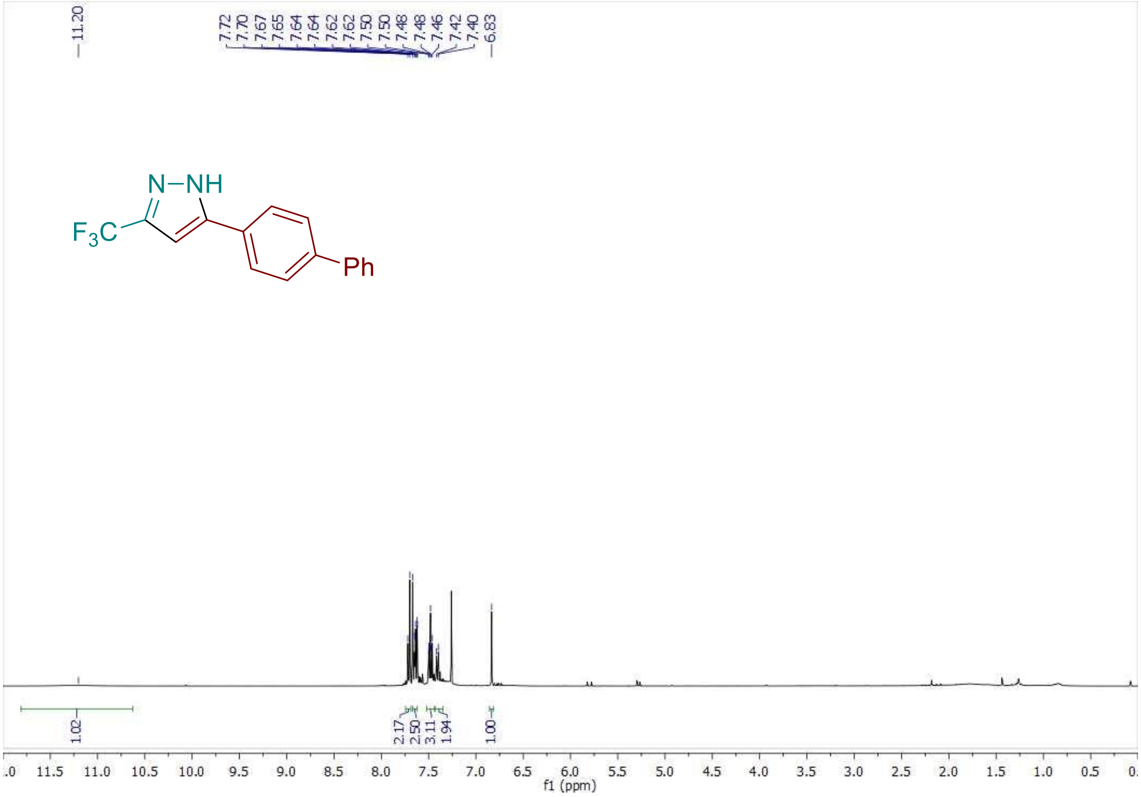

$^{13}C\{^{1}H\}$ NMR (101 MHz, $CDCl_3$)

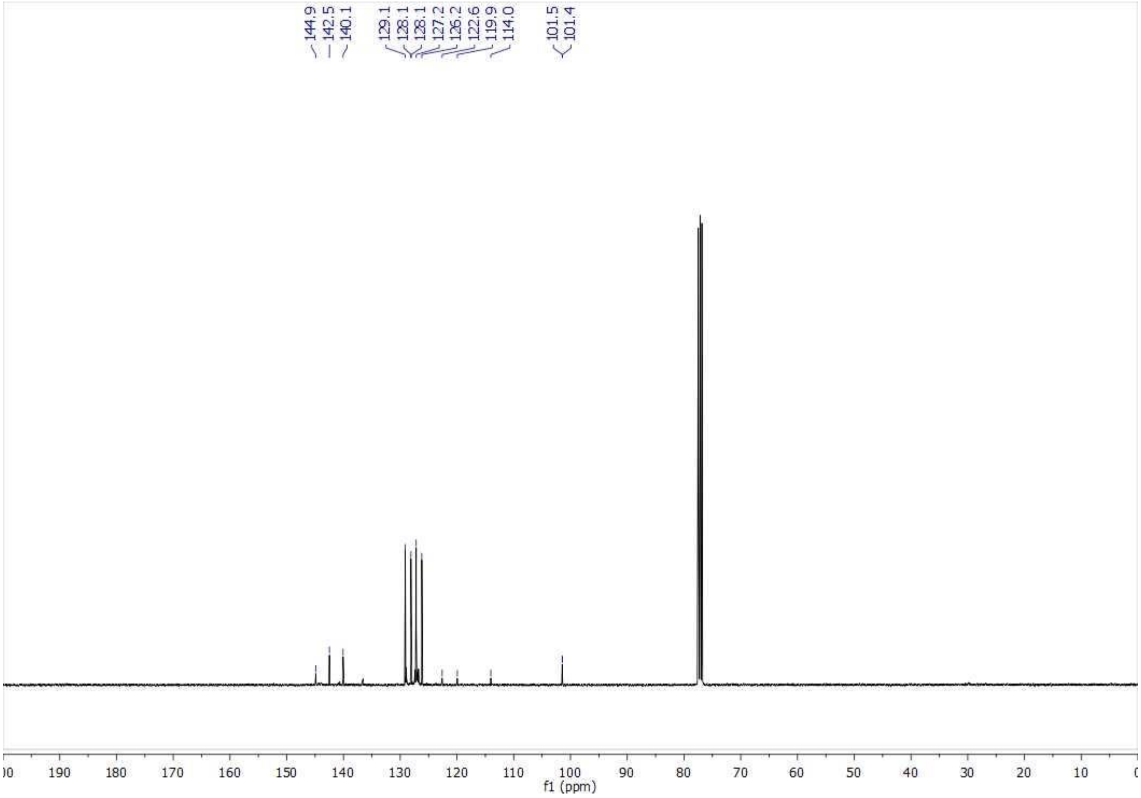

^{19}F NMR (376 MHz, $CDCl_3$)

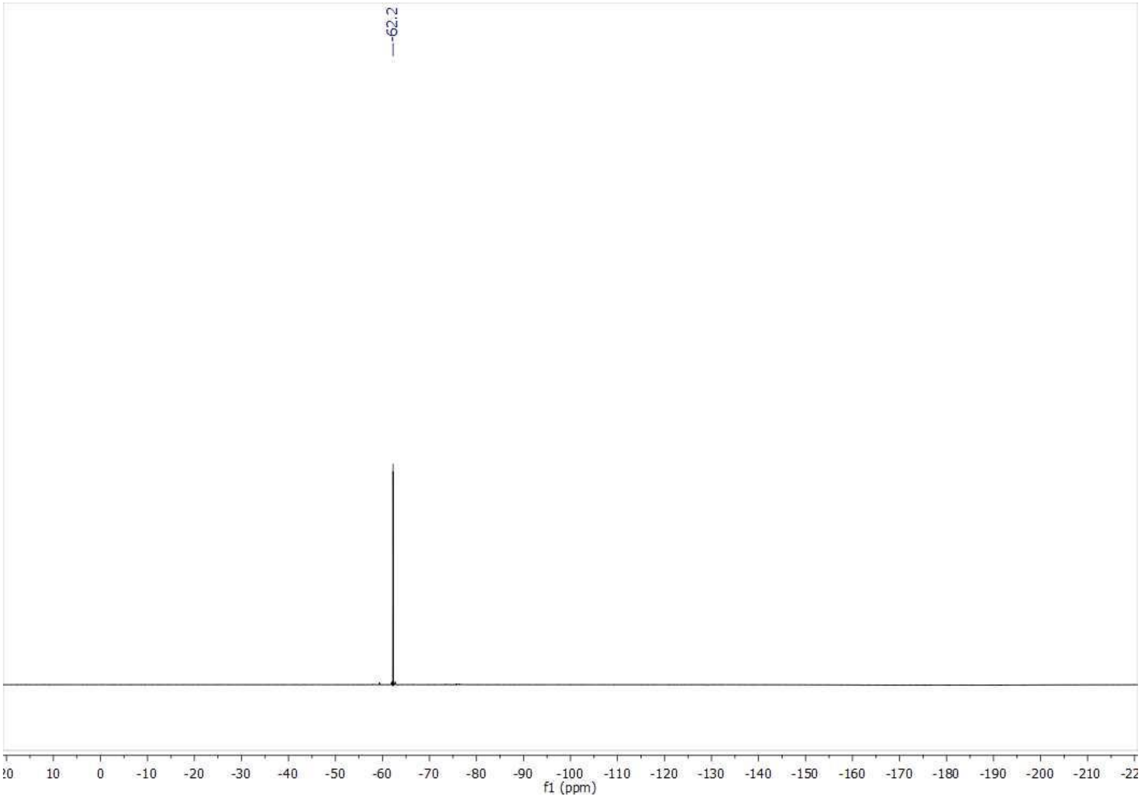

5-(4-Fluorophenyl)-3-(trifluoromethyl)-1*H*-pyrazole (5j)

^{1}H NMR (400 MHz, $CDCl_3$)

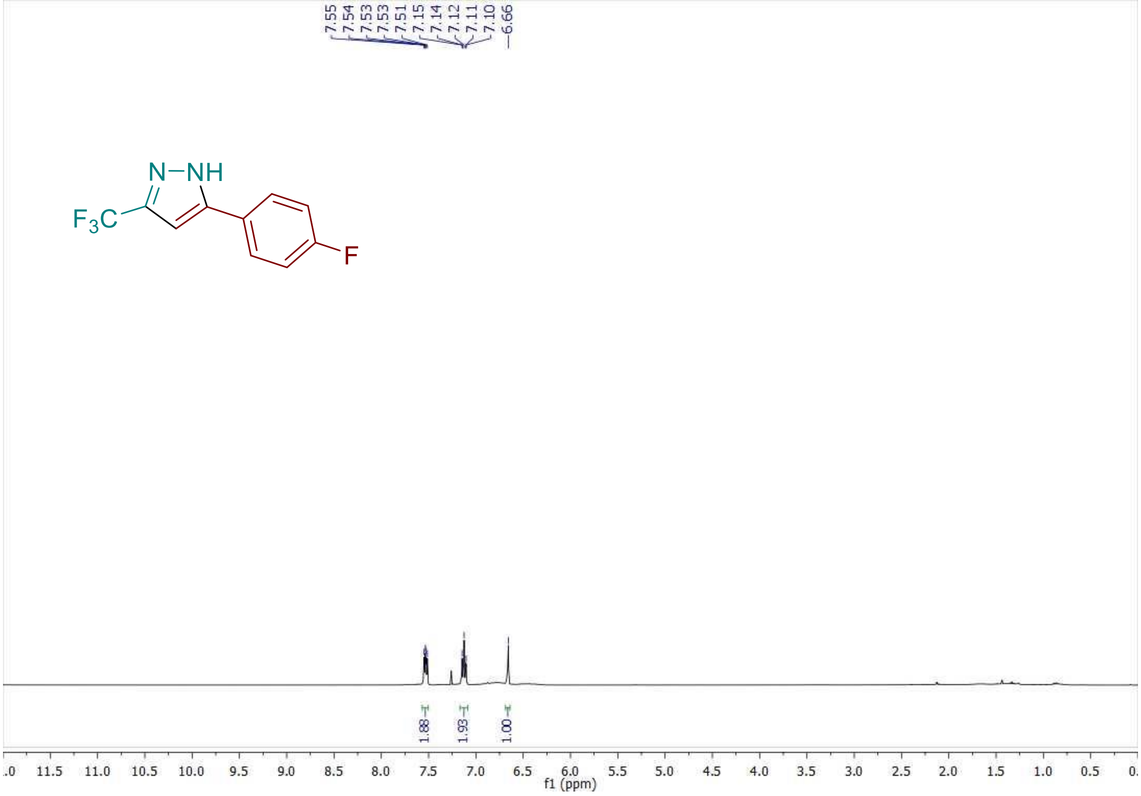

$^{13}C\{^{1}H\}$ NMR (101 MHz, $CDCl_3$)

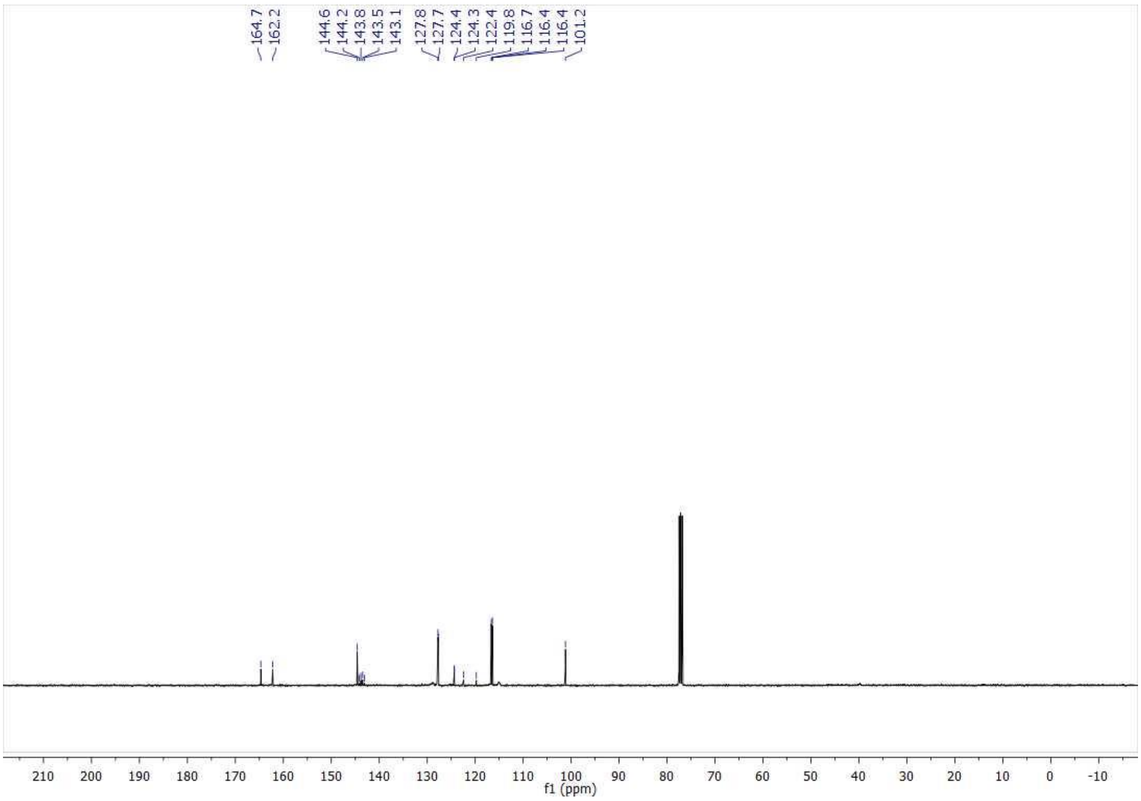

^{19}F NMR (376 MHz, $CDCl_3$)

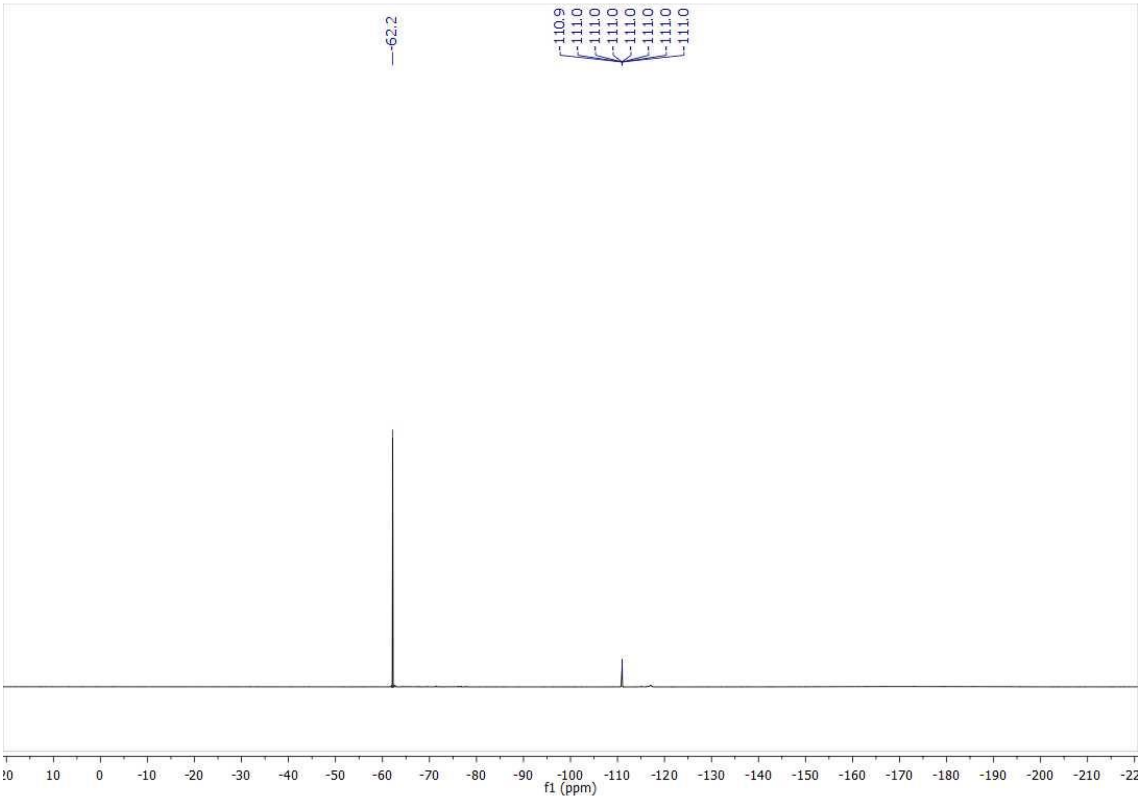

5-(4-Chlorophenyl)-3-(trifluoromethyl)-1H-pyrazole (5k)

^{1}H NMR (400 MHz, $CDCl_3$)

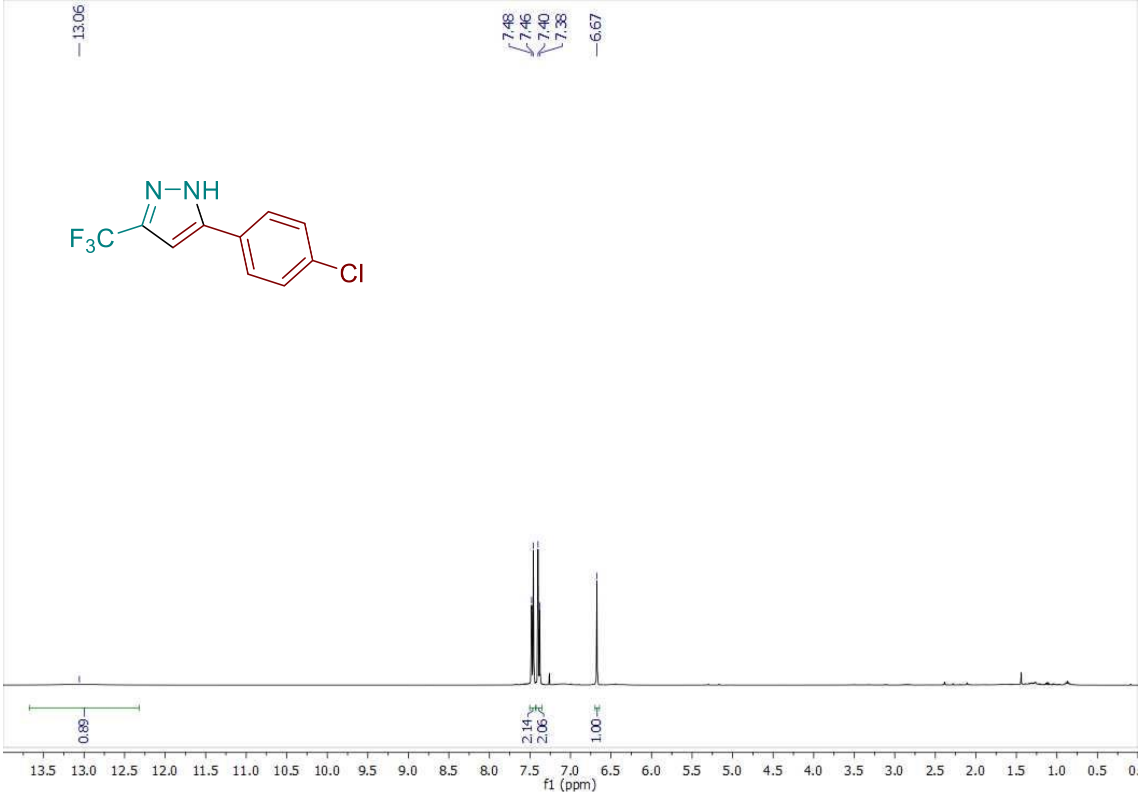

$^{13}C\{^{1}H\}$ NMR (101 MHz, $CDCl_3$)

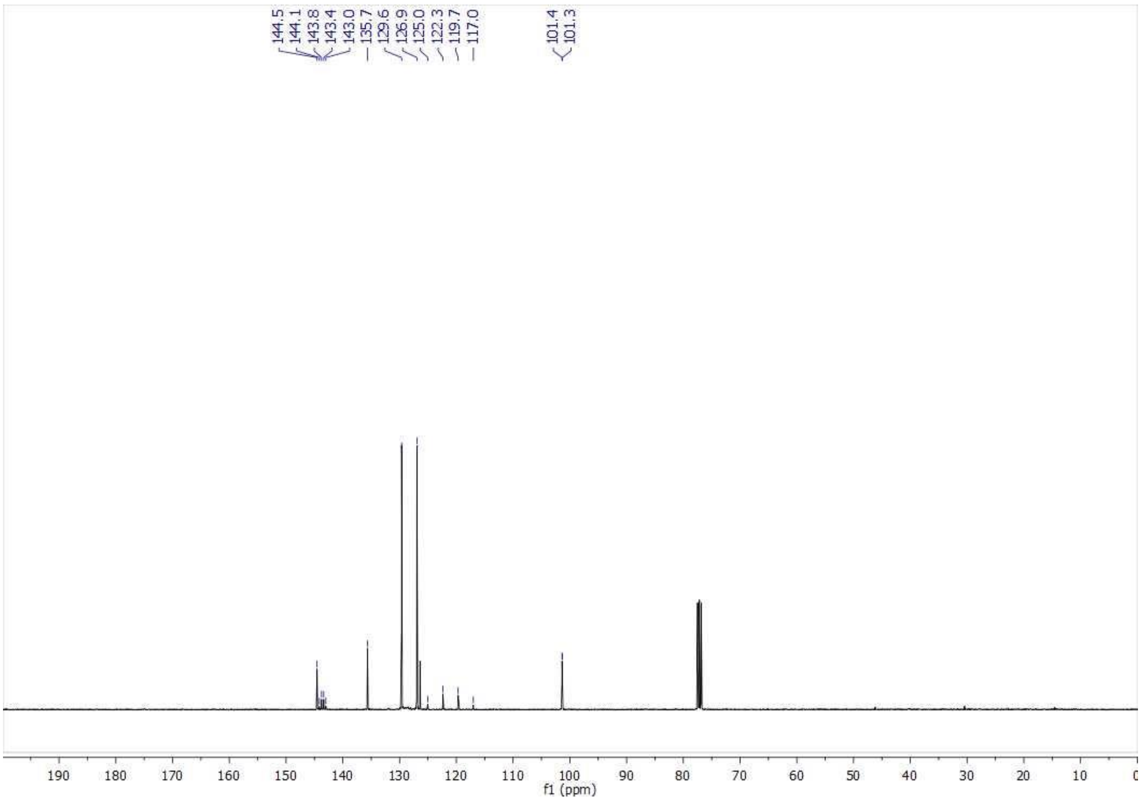

^{19}F NMR (376 MHz, $CDCl_3$)

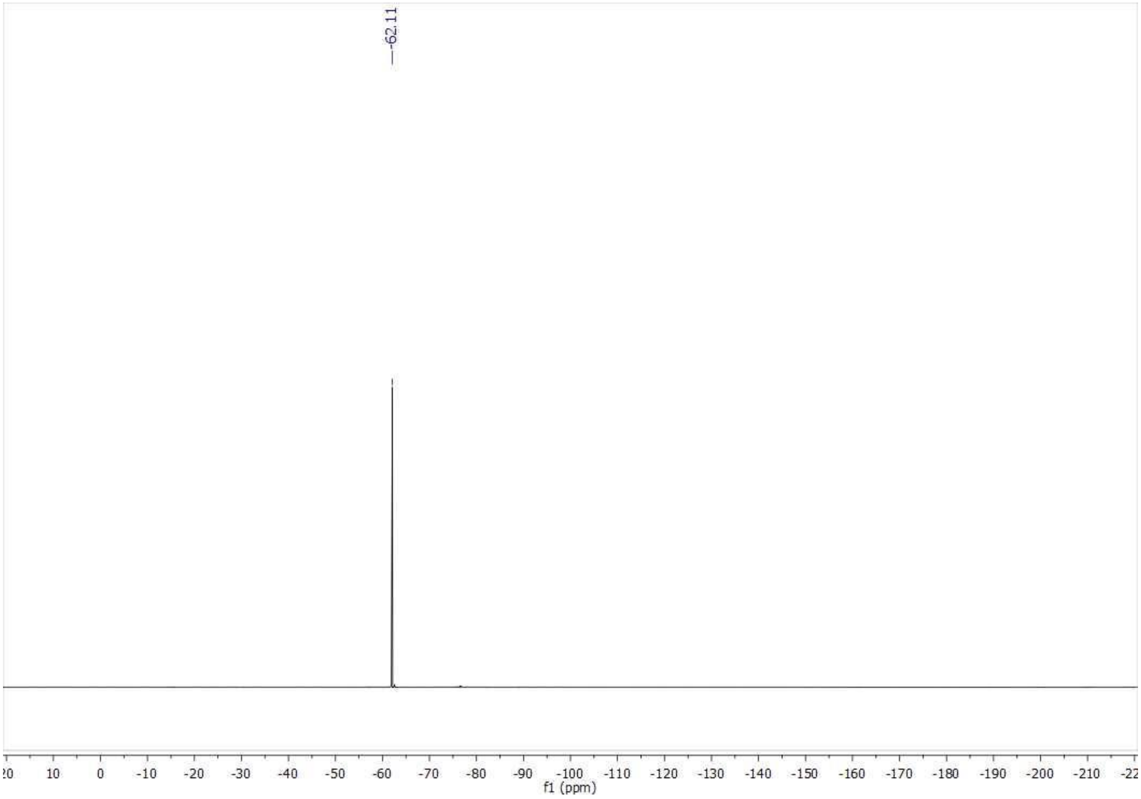

5-(4-Bromophenyl)-3-(trifluoromethyl)-1*H*-pyrazole (5l)

^{1}H NMR (400 MHz, $CDCl_3$)

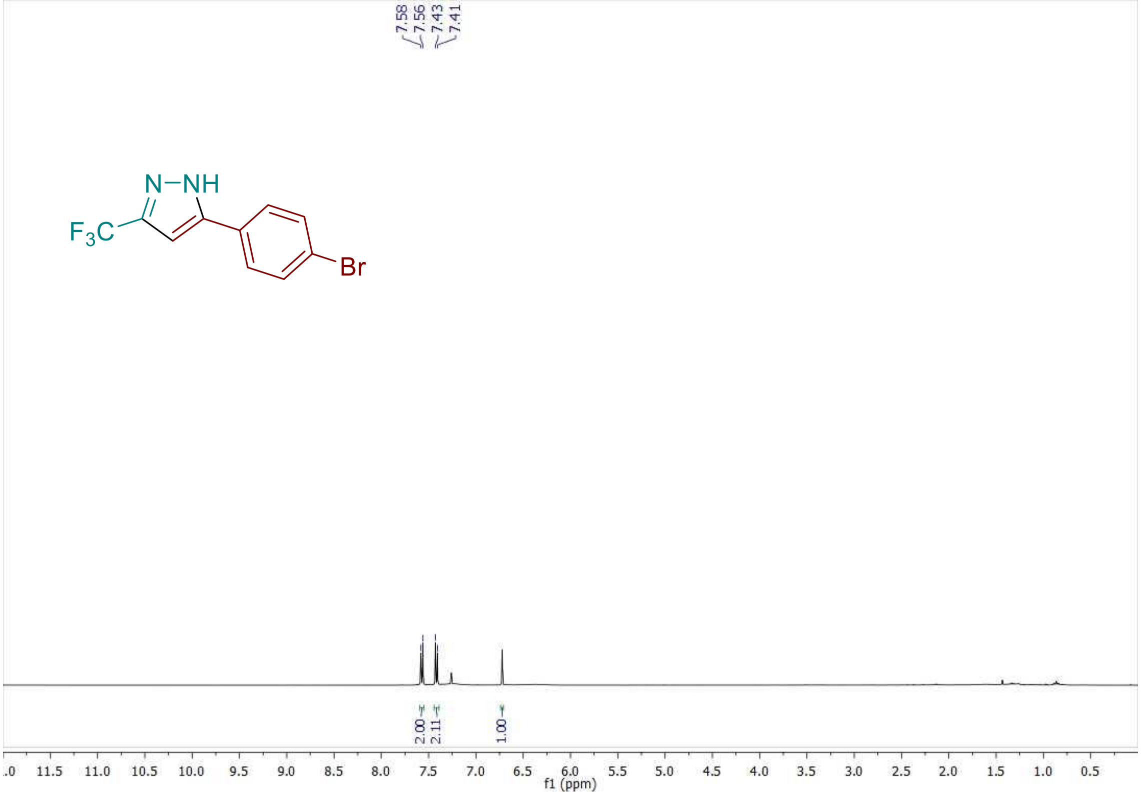

^{13}C{^{1}H} NMR (101 MHz, $CDCl_3$)

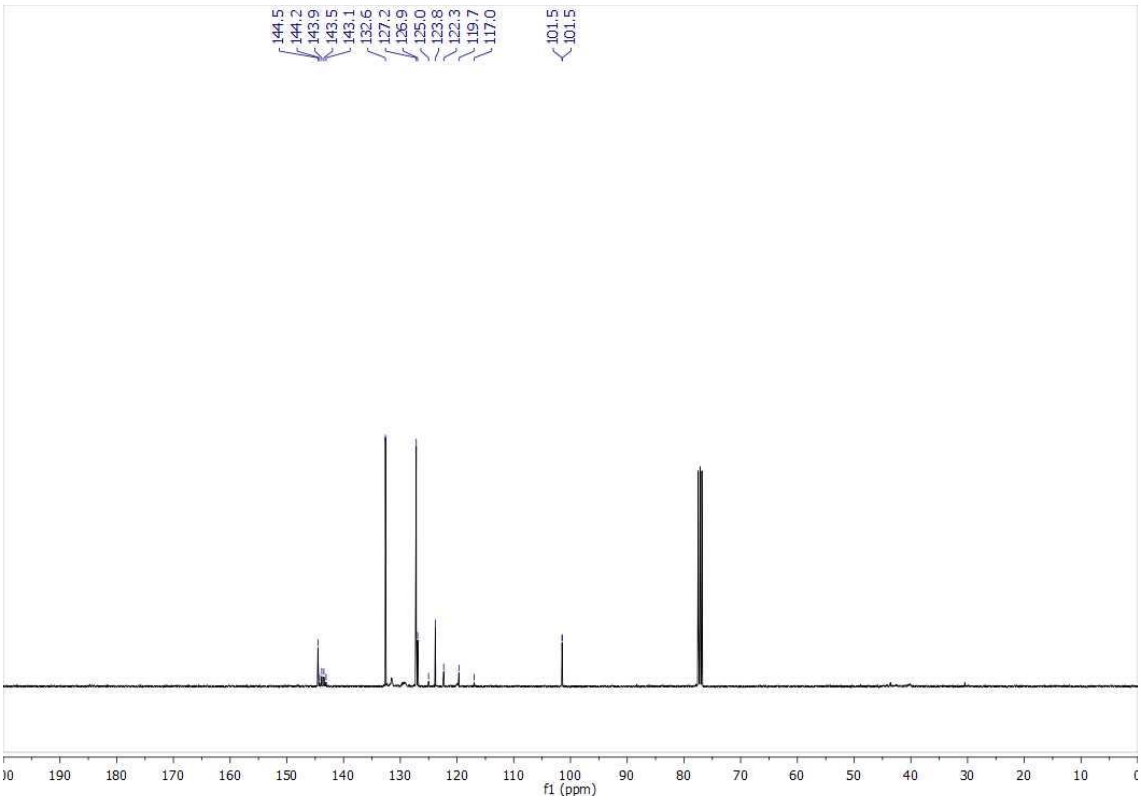

^{19}F NMR (376 MHz, $CDCl_3$)

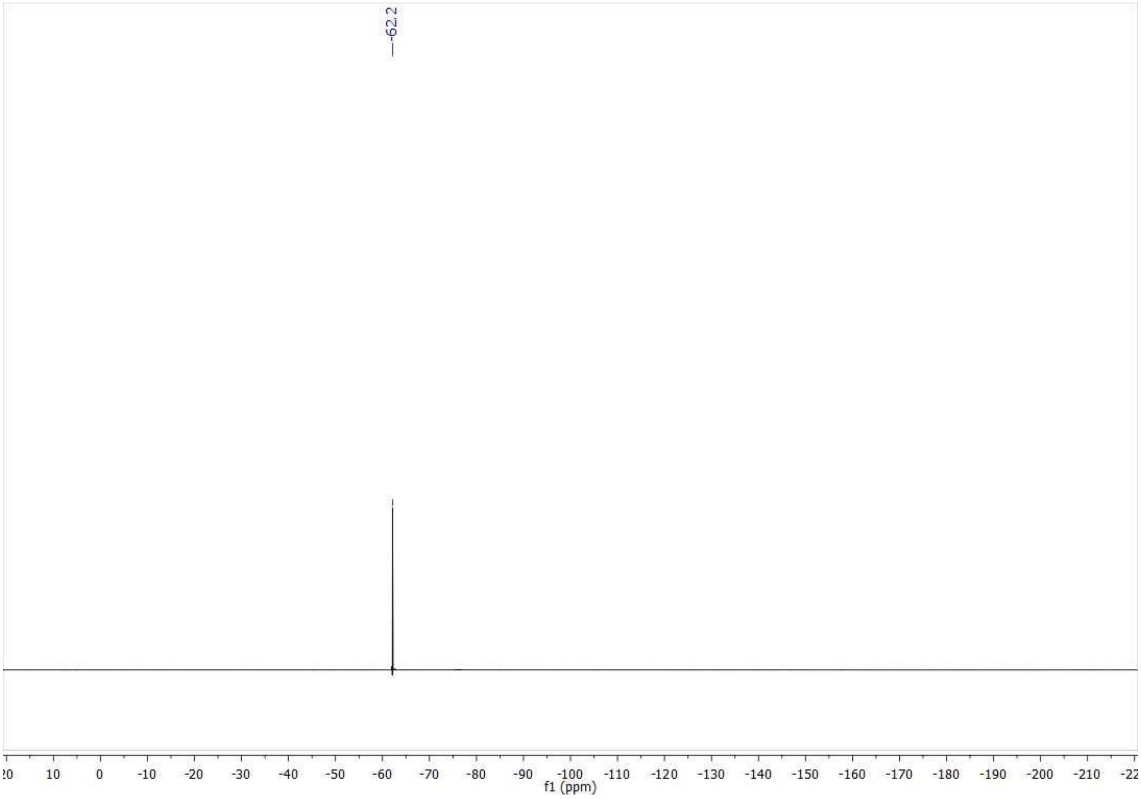

5-(*p*-(Anisol)-3-(trifluoromethyl)-1*H*-pyrazole (5m)

^{1}H NMR (400 MHz, $CDCl_3$)

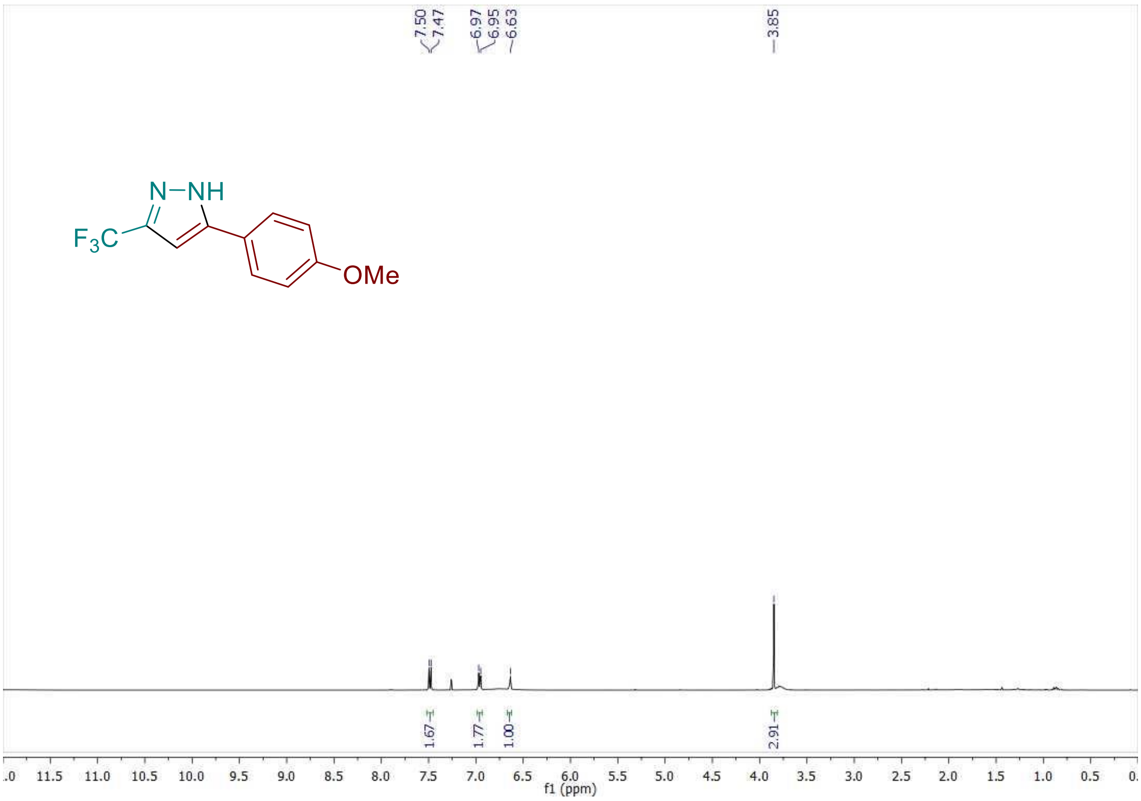

$^{13}C\{^{1}H\}$ NMR (101 MHz, $CDCl_3$)

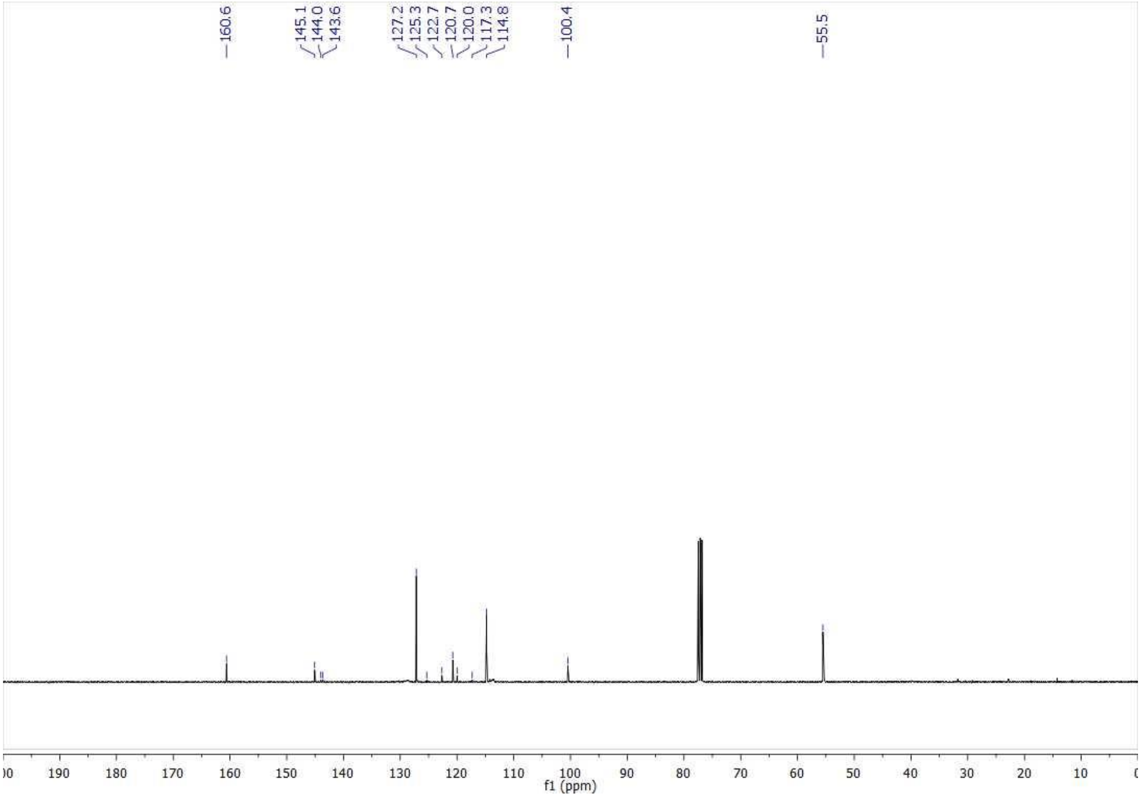

^{19}F NMR (376 MHz, $CDCl_3$)

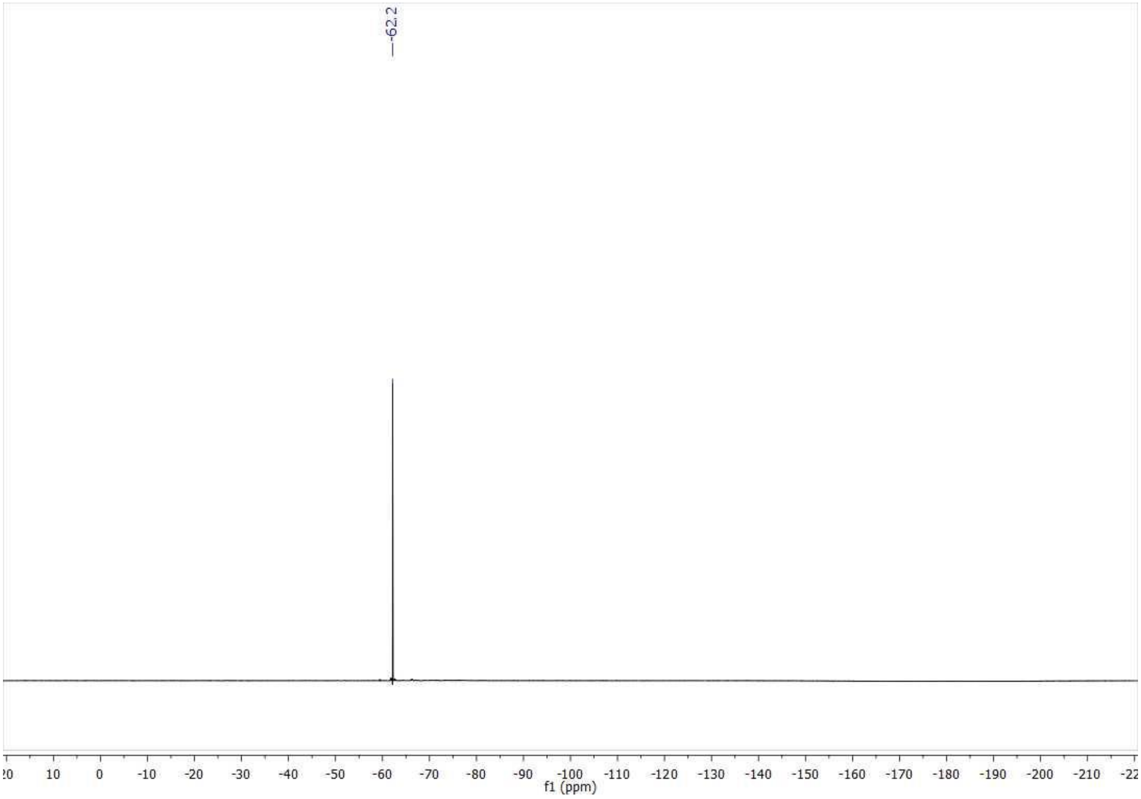

5-(*p*-(Methyltio)phenyl-3-(trifluoromethyl)-1*H*-pyrazole (5n)

^{1}H NMR (400 MHz, $CDCl_3$)

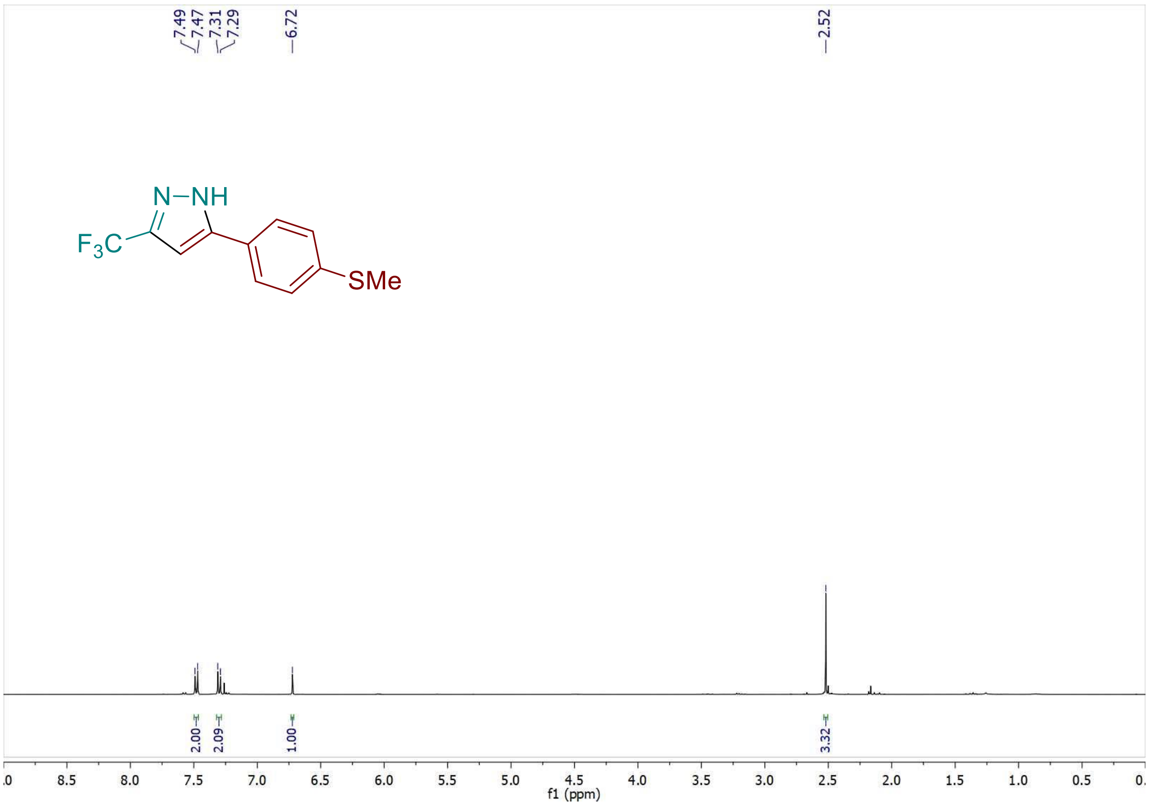

$^{13}C\{^{1}H\}$ NMR (101 MHz, $CDCl_3$)

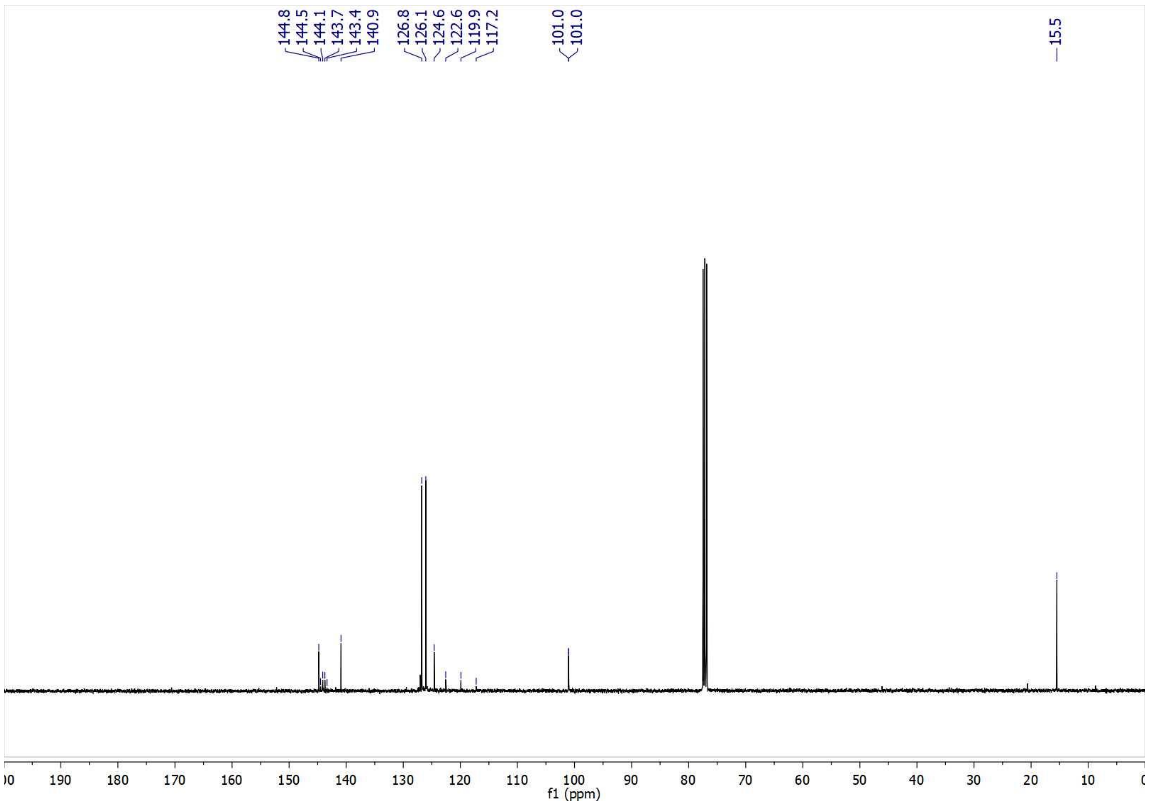

^{19}F NMR (376 MHz, $CDCl_3$)

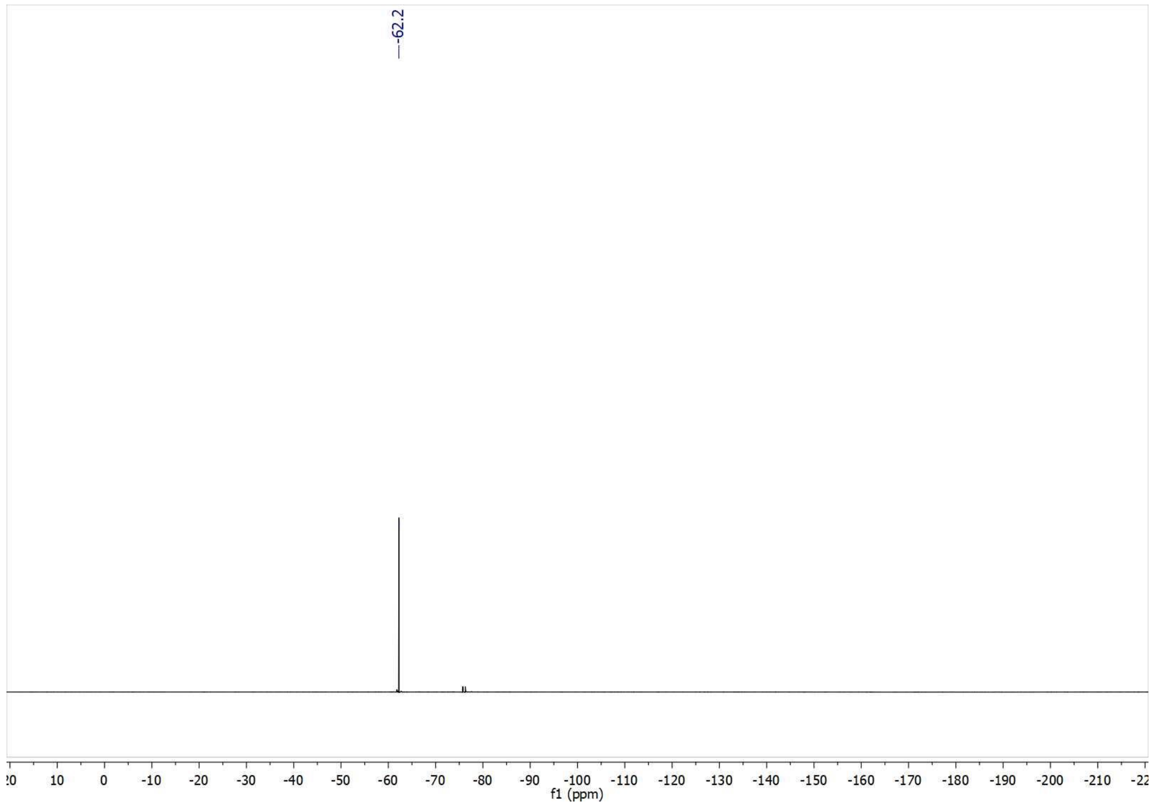

4-(3-(Trifluoromethyl)-1*H*-pyrazol-5-yl)phenyl acetate (5o)

^{1}H NMR (400 MHz, $CDCl_3$)

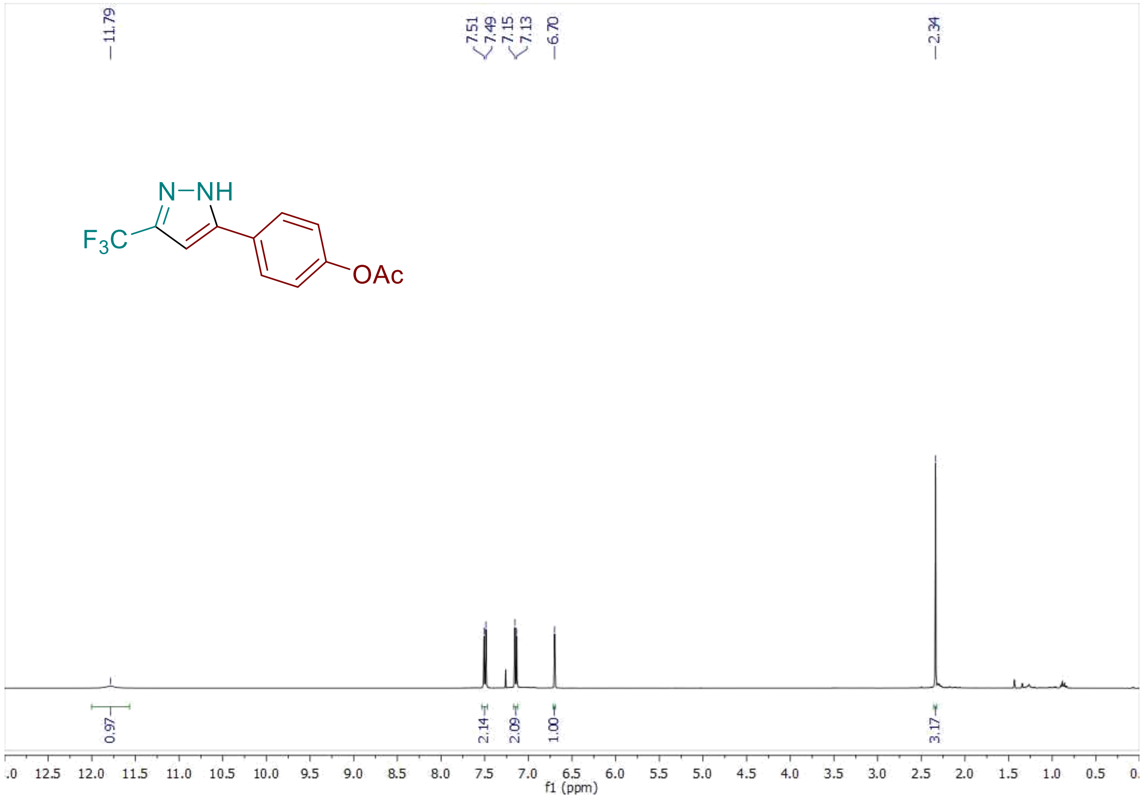

$^{13}C\{^{1}H\}$ NMR (101 MHz, $CDCl_3$)

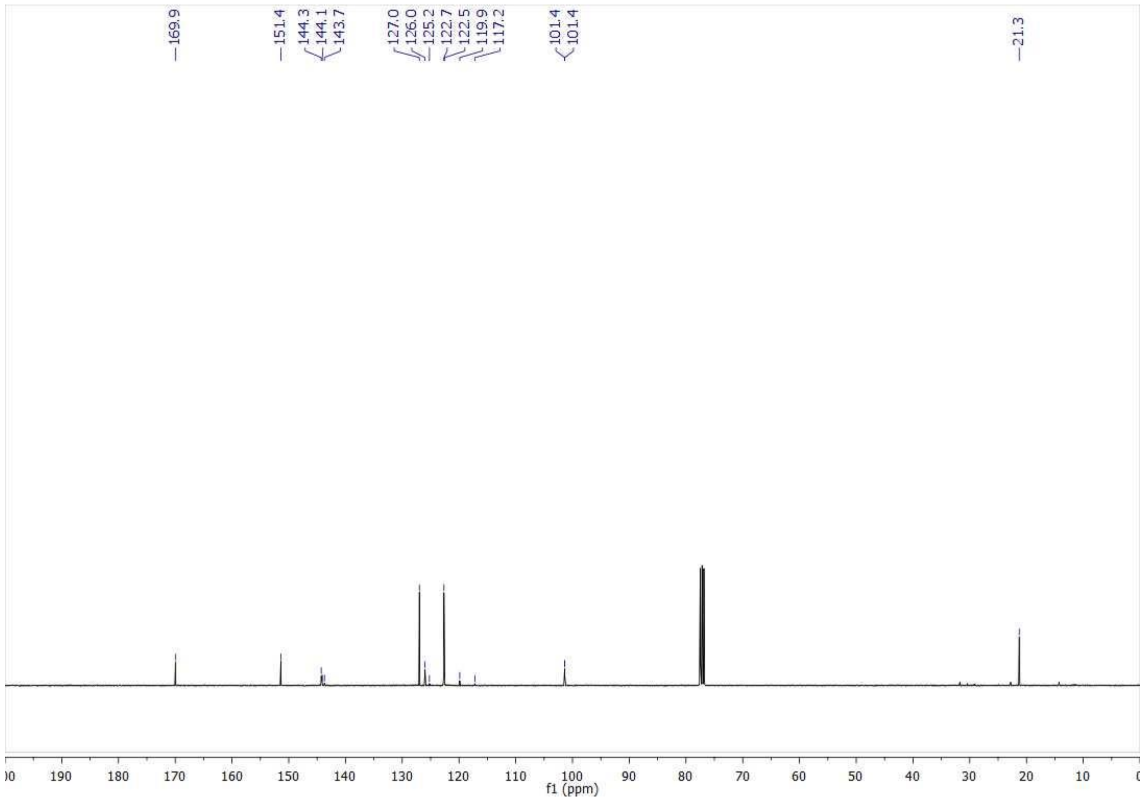

^{19}F NMR (376 MHz, $CDCl_3$)

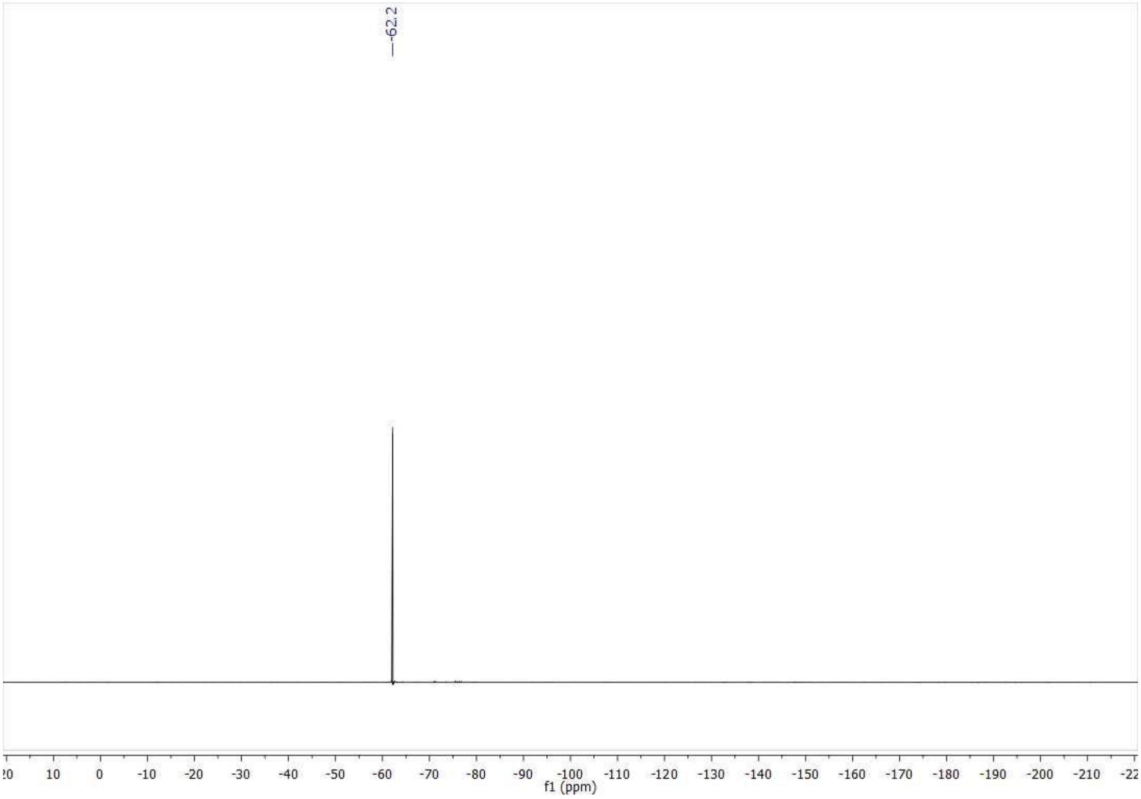

5-(*p*-Benzonitrile)-3-(trifluoromethyl)-1*H*-pyrazole (5p)

^{1}H NMR (400 MHz, $CDCl_3$)

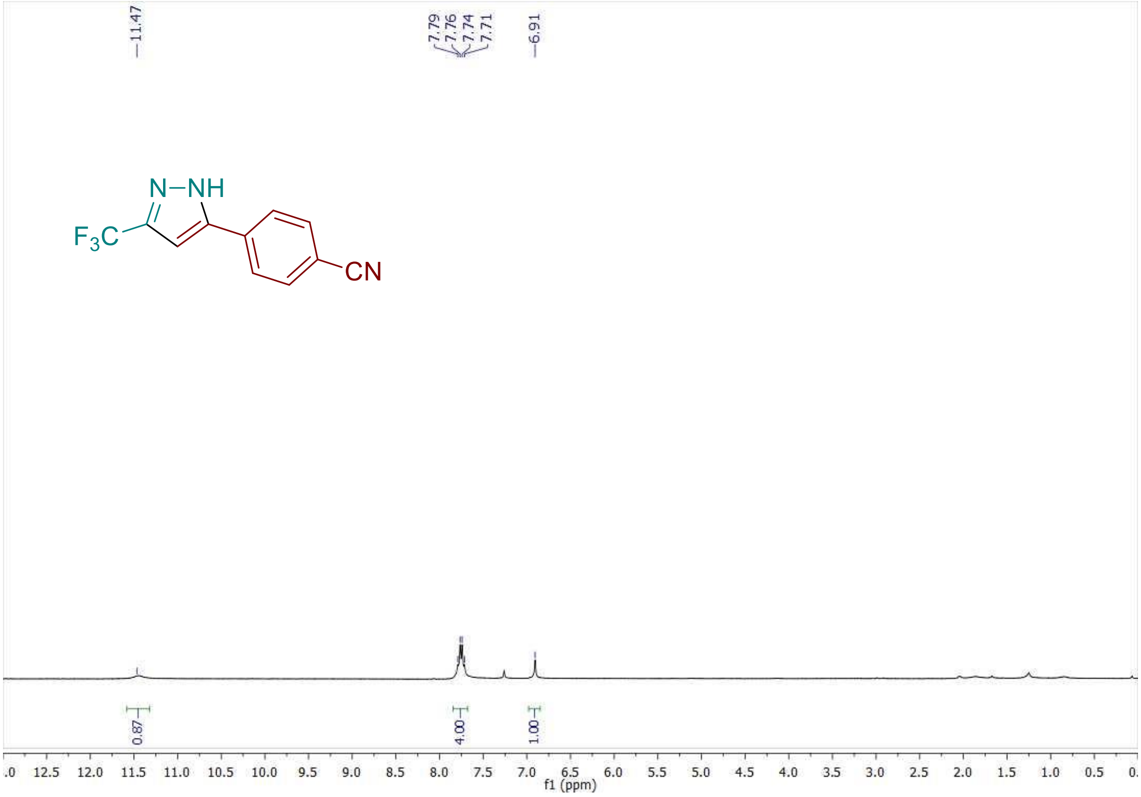

$^{13}C\{^{1}H\}$ NMR (126 MHz, $CDCl_3$)

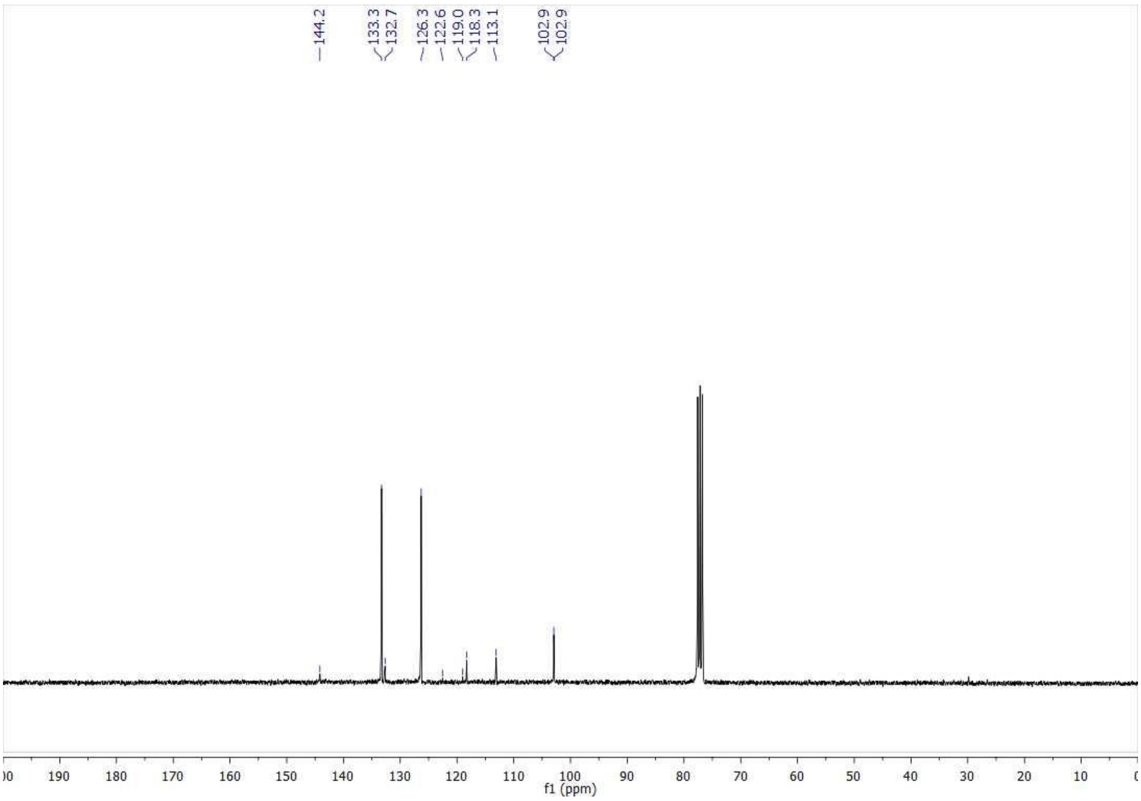

^{19}F NMR (376 MHz, $CDCl_3$)

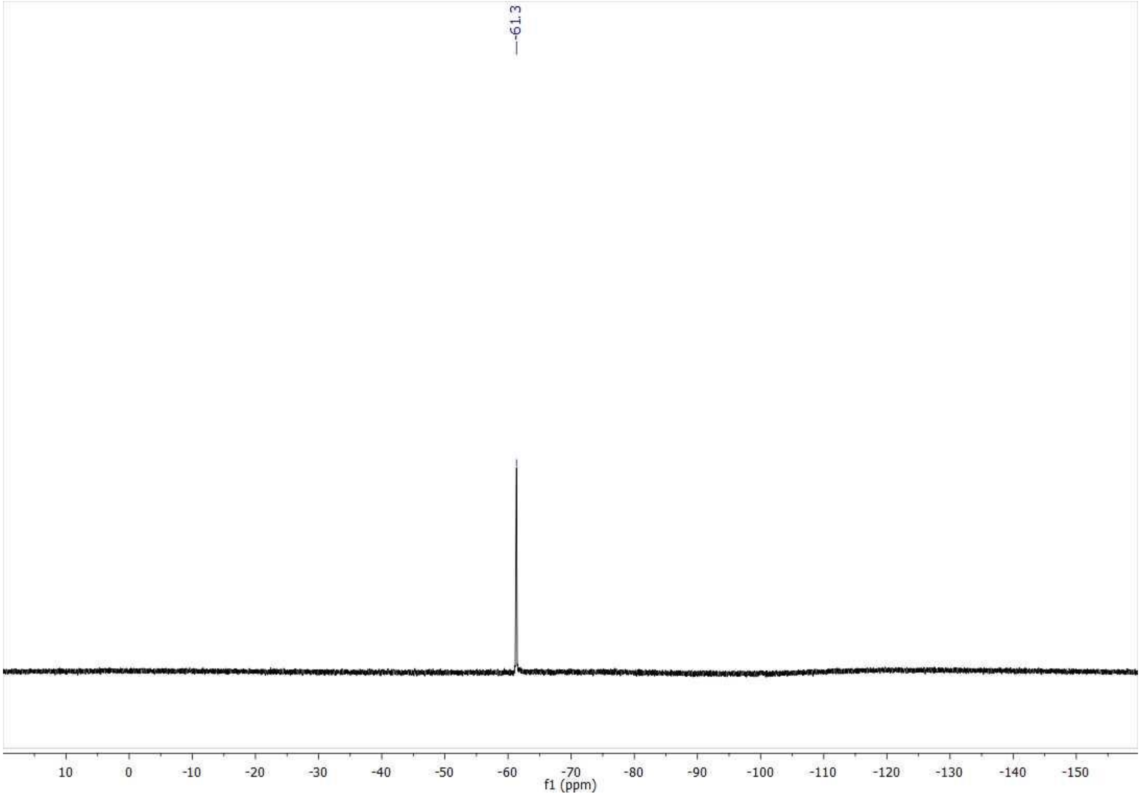

5-(*p*-(Trifluoromethyl)phenyl)-3-(trifluoromethyl)-1*H*-pyrazole (5q)

^{1}H NMR (400 MHz, $CDCl_3$)

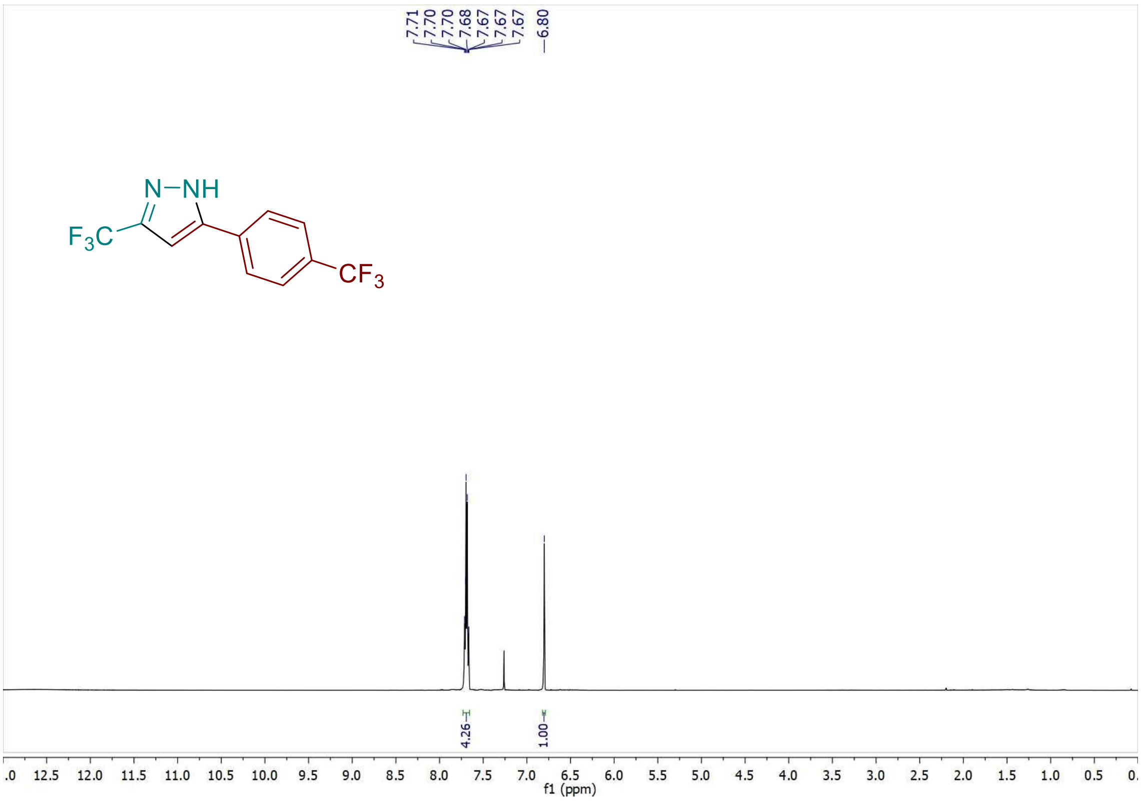

^{13}C{^{1}H} NMR (126 MHz, $CDCl_3$)

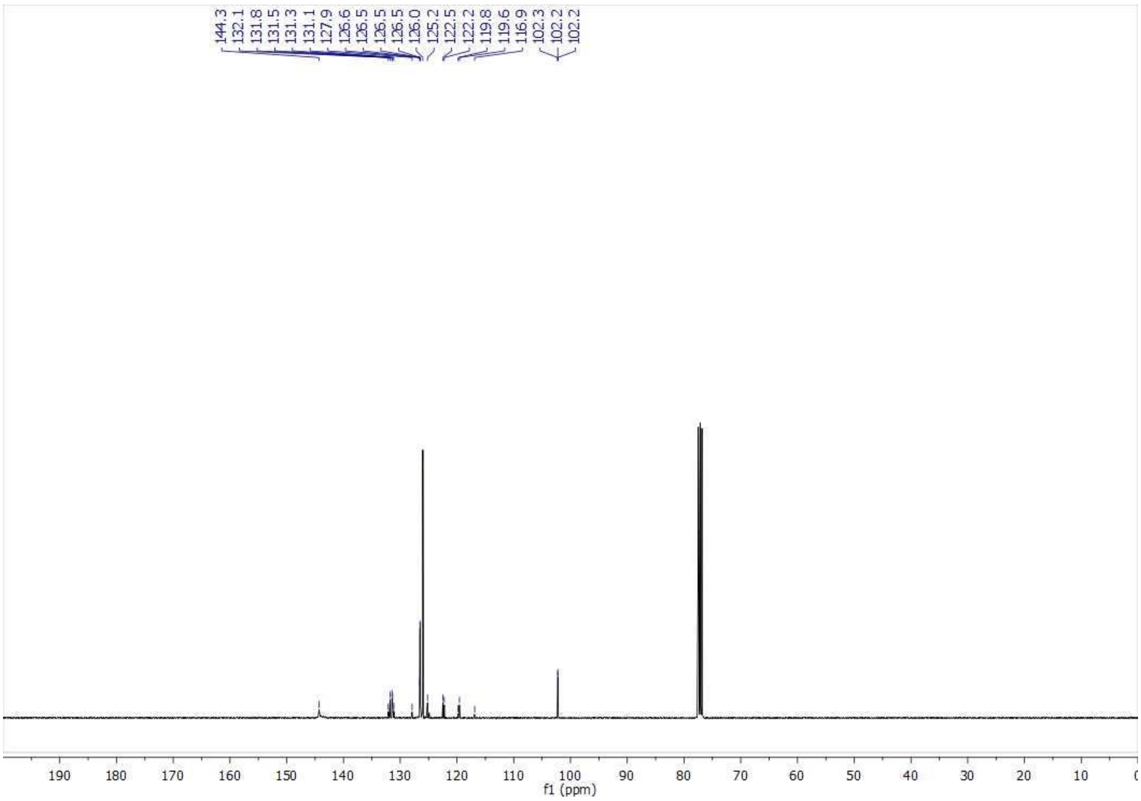

^{19}F NMR (376 MHz, $CDCl_3$)

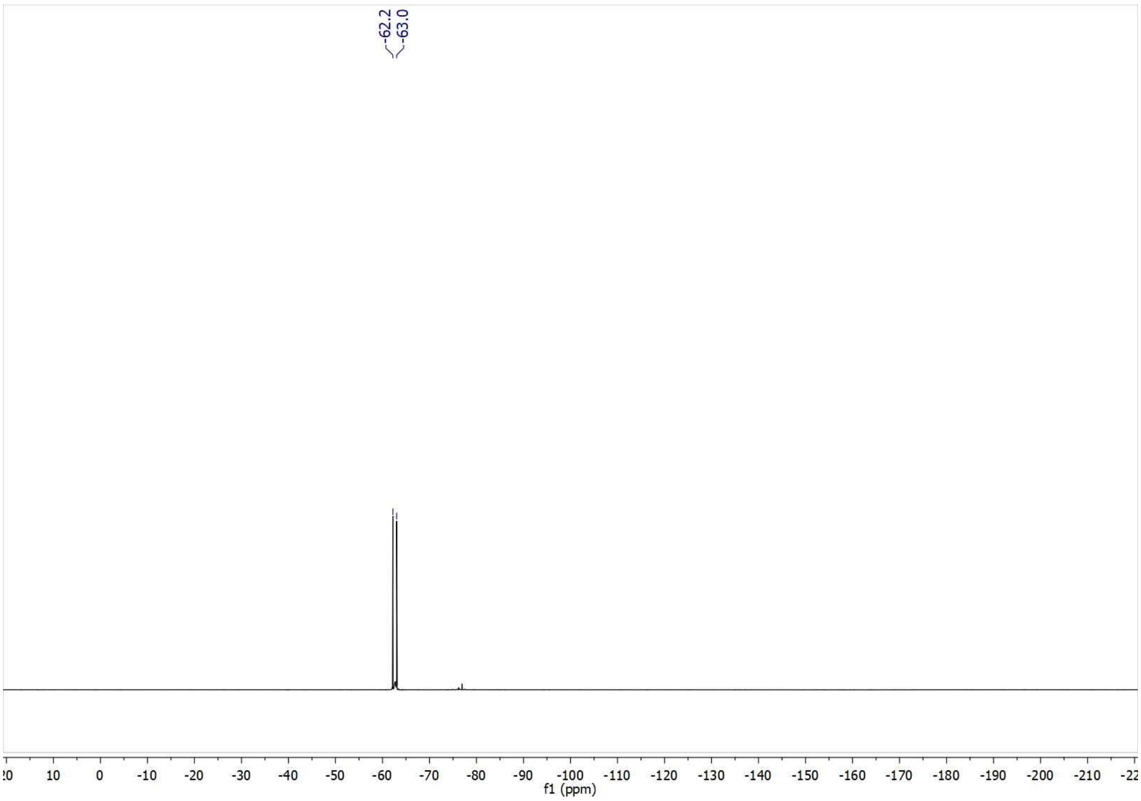

Methyl 4-(3-(trifluoromethyl)-1*H*-pyrazol-5-yl)benzoate (5r)

^{1}H NMR (500 MHz, $CDCl_3$)

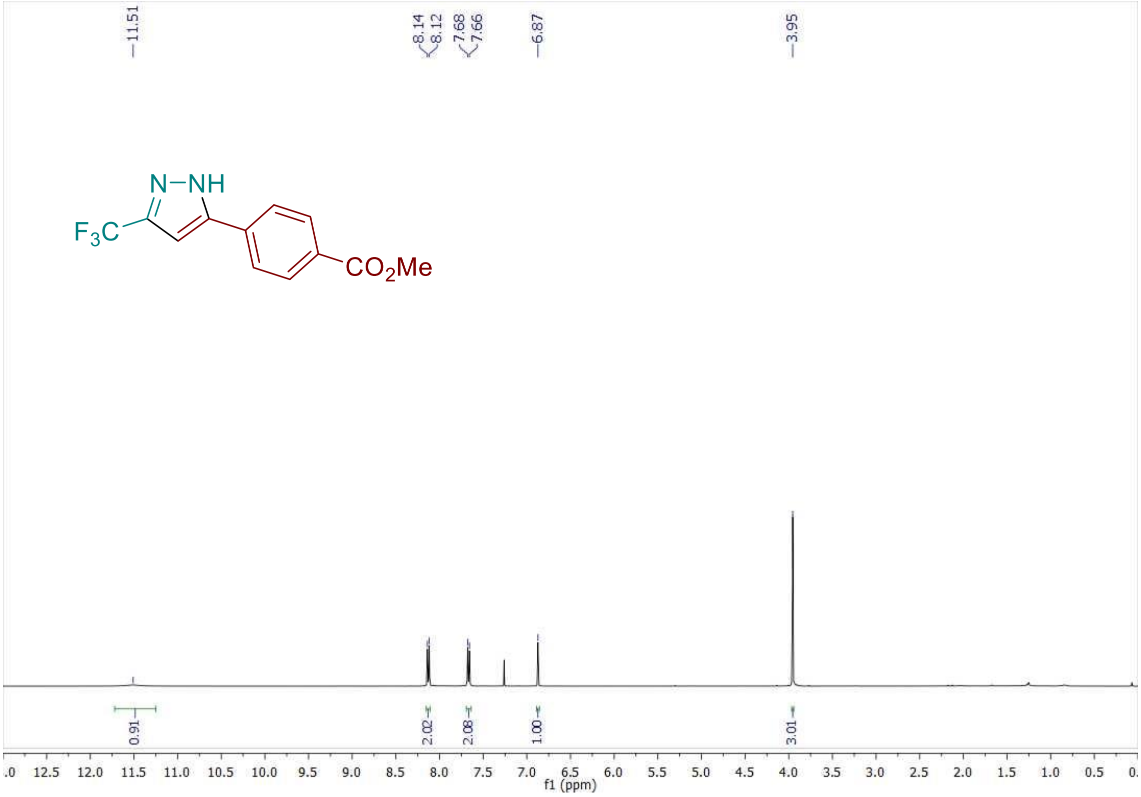

^{13}C{^{1}H} NMR (101 MHz, $CDCl_3$)

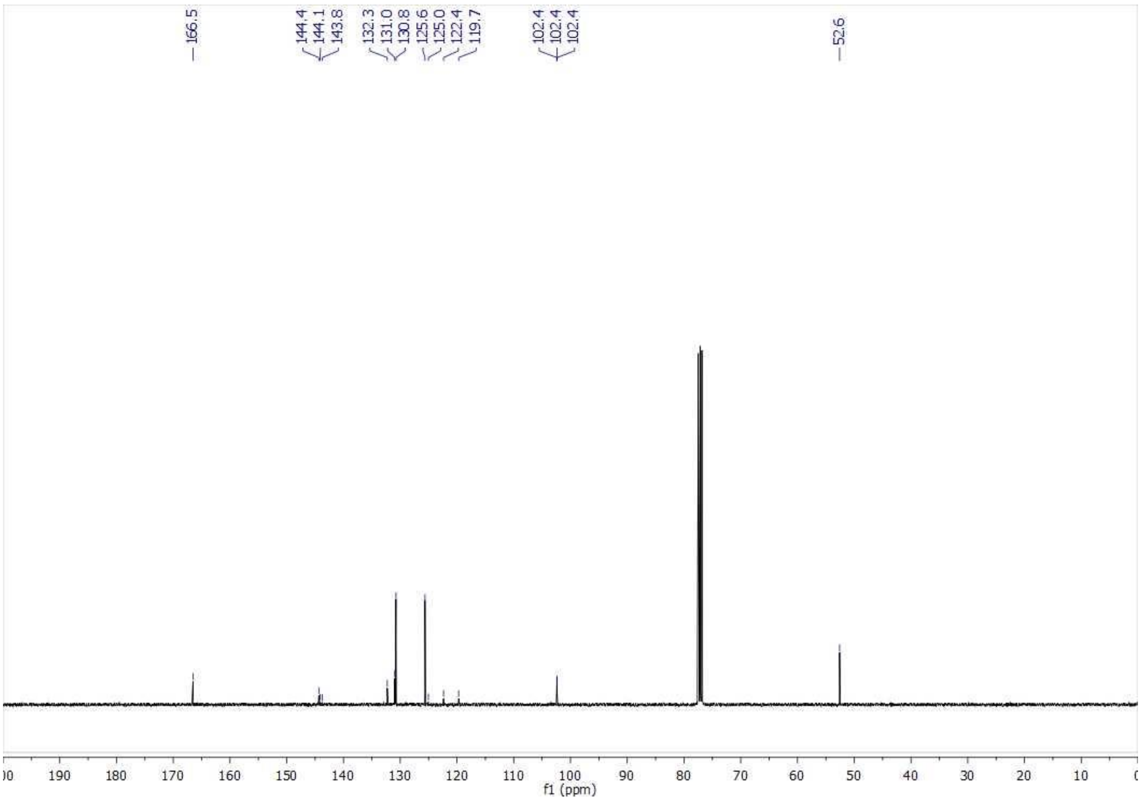

^{19}F NMR (376 MHz, $CDCl_3$)

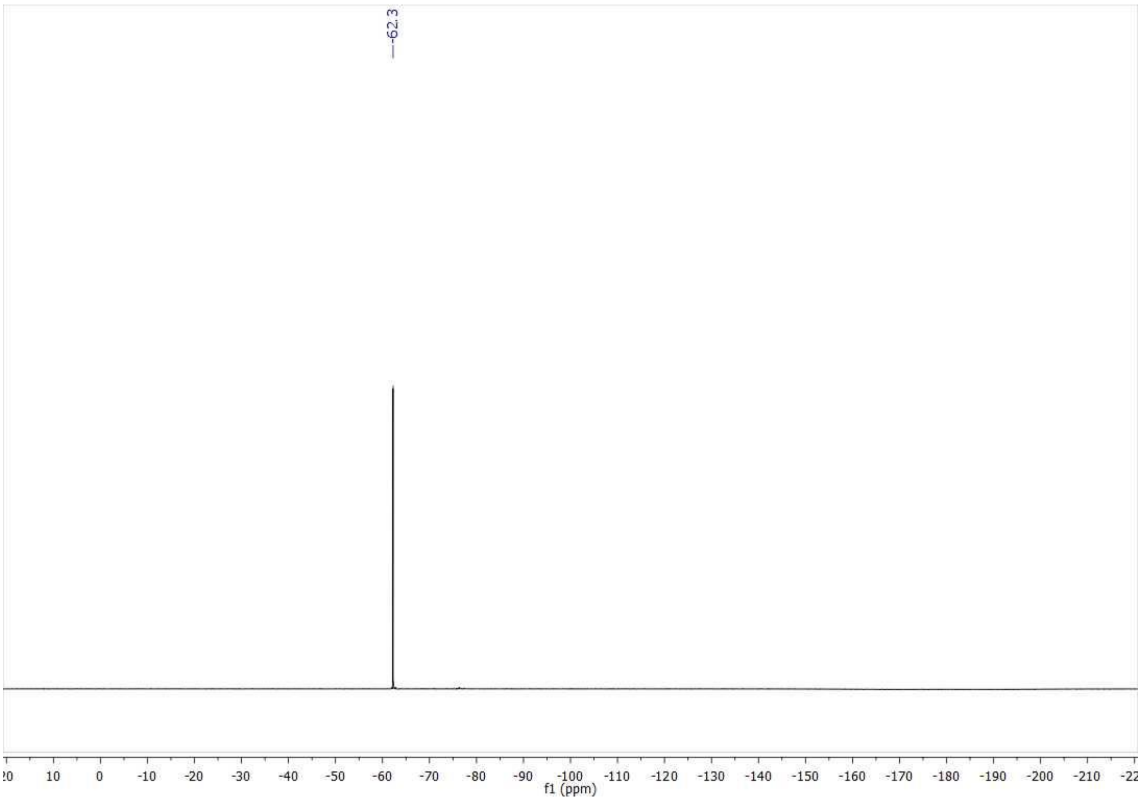

5-(4-(4,4,5,5-Tetramethyl-1,3,2-dioxaborolan-2-yl)phenyl)-3-(trifluoromethyl)-1*H*-pyrazole (5s)

^{1}H NMR (400 MHz, $CDCl_3$)

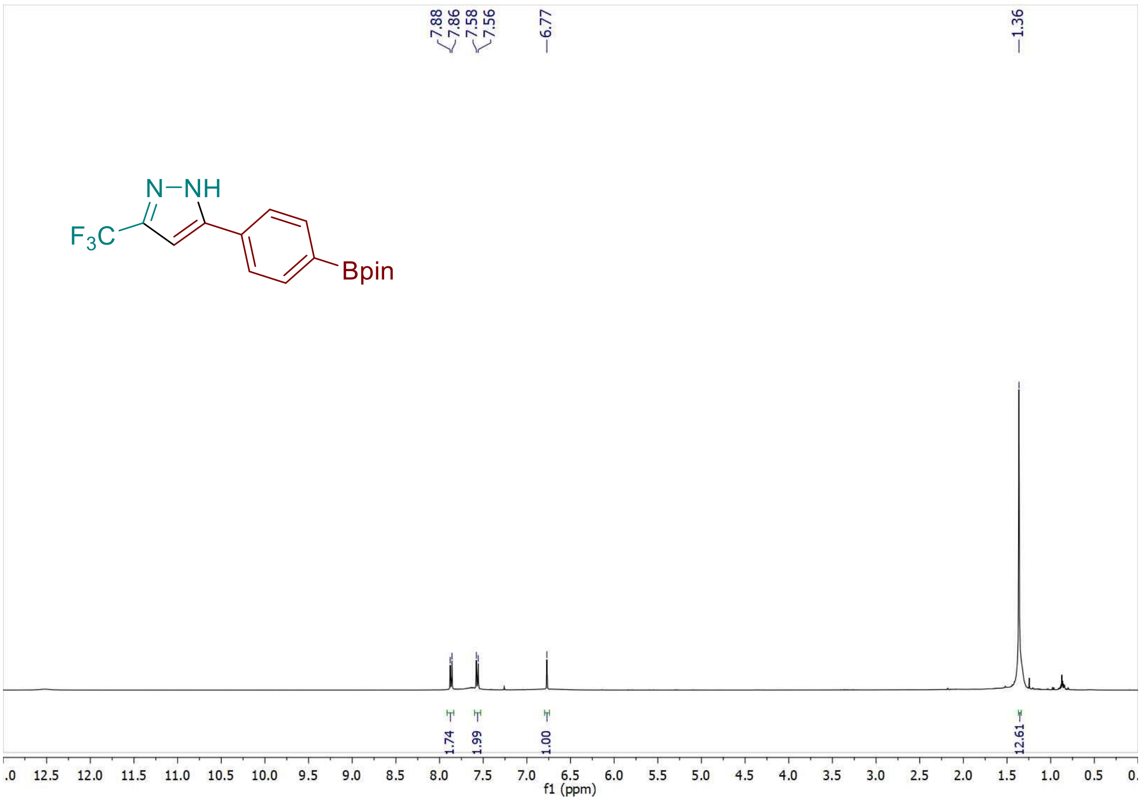

$^{13}C\{^1H\}$ NMR (126 MHz, $CDCl_3$)

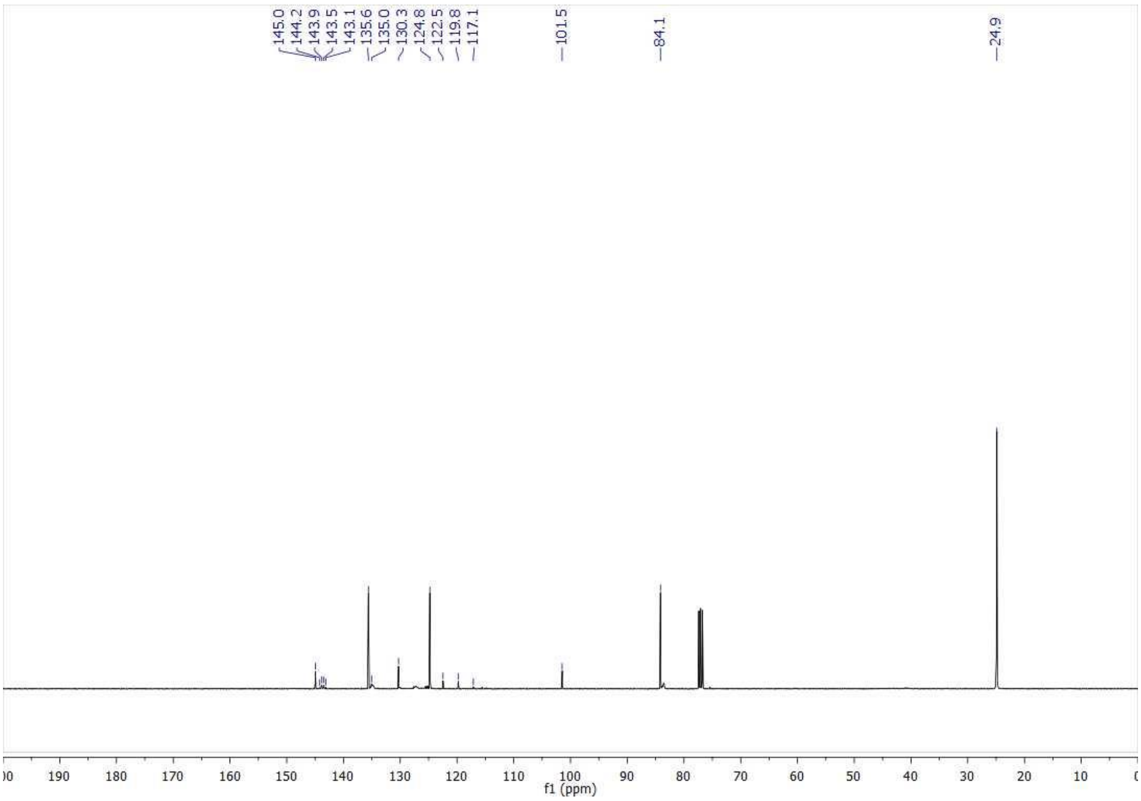

^{19}F NMR (376 MHz, $CDCl_3$)

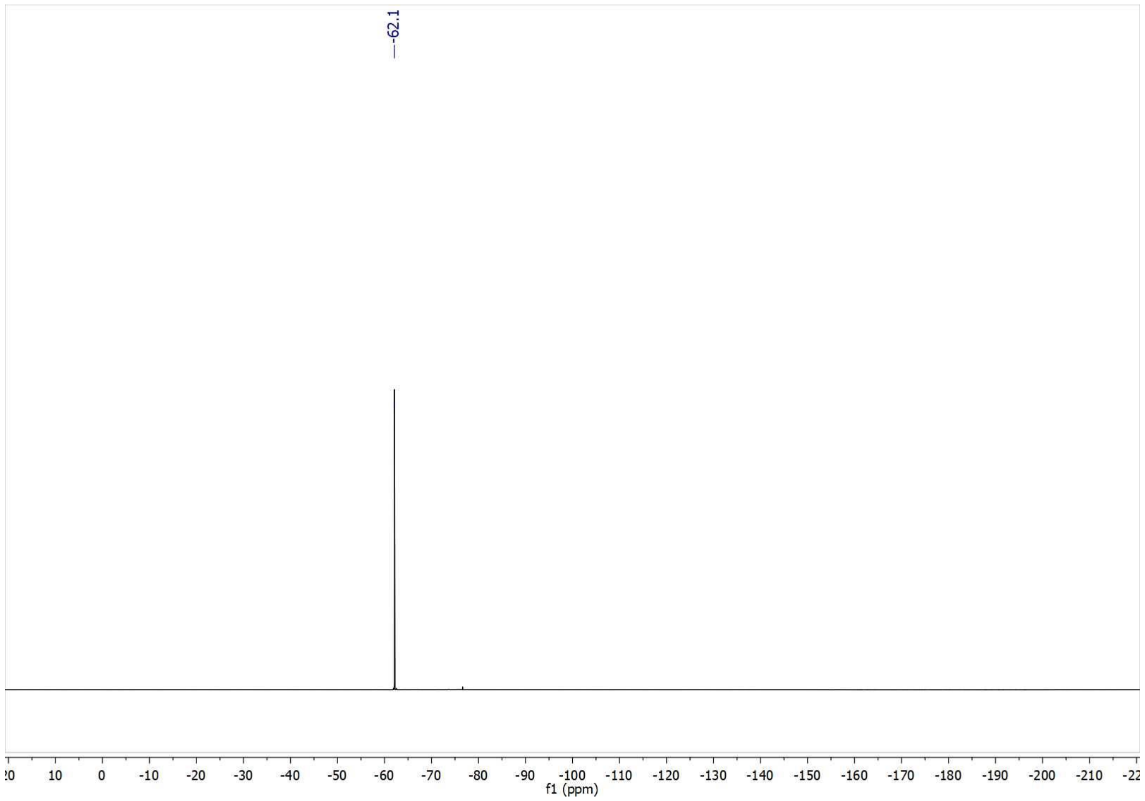

5-(2,5-Dimethylphenyl)-3-(trifluoromethyl)-1*H*-pyrazole (5t)

^{1}H NMR (400 MHz, $CDCl_3$)

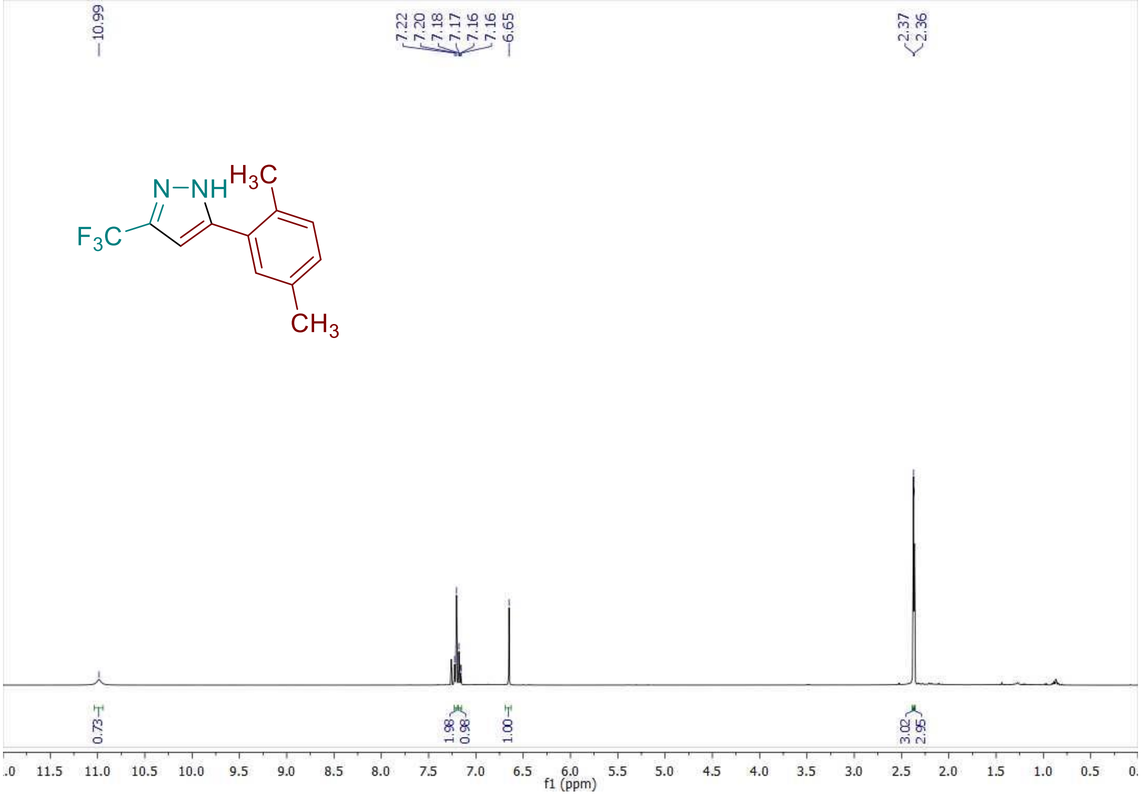

$^{13}C\{^{1}H\}$ NMR (101 MHz, $CDCl_3$)

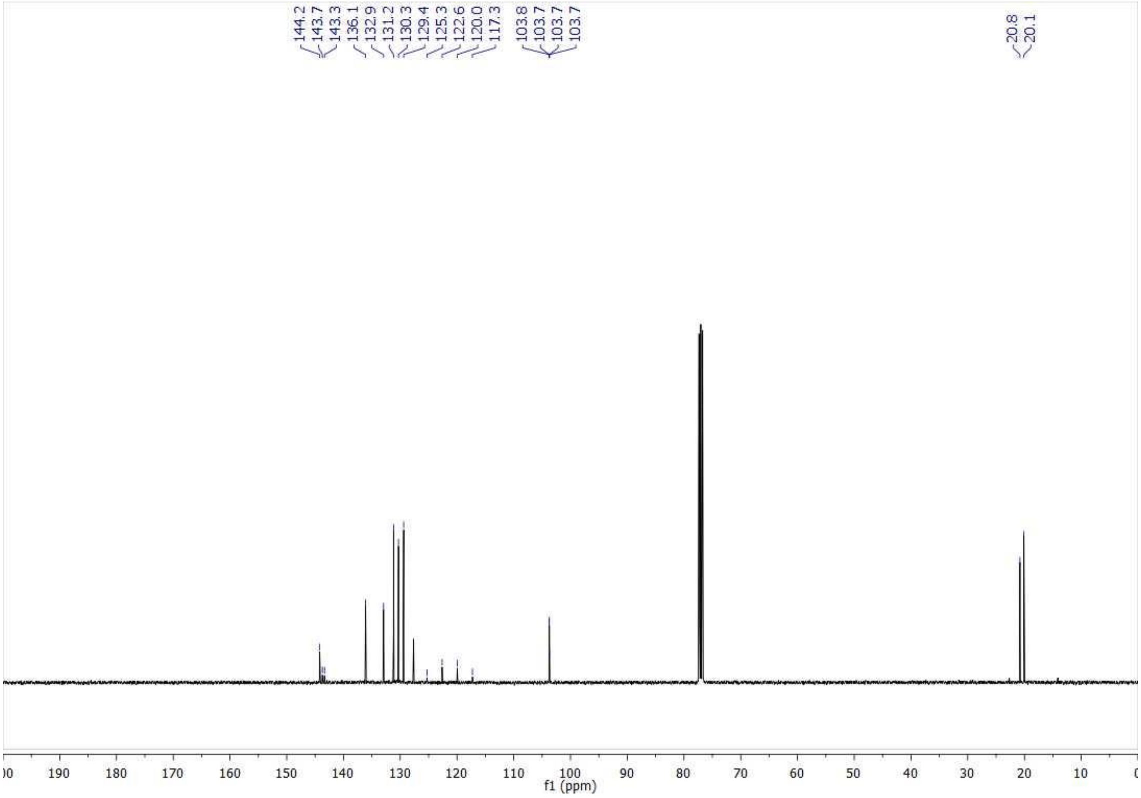

^{19}F NMR (376 MHz, $CDCl_3$)

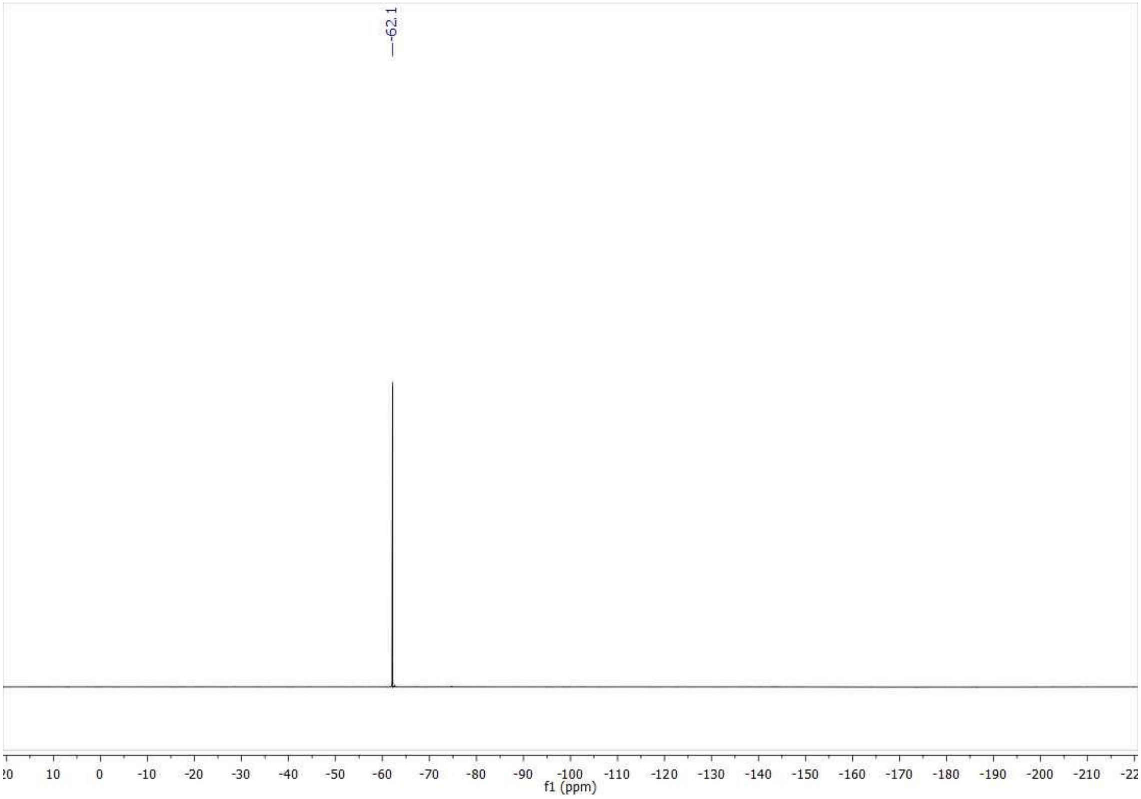

5-(3,5-Bis(trifluoromethyl)phenyl)-3-(trifluoromethyl)-1H-pyrazole (5v)

^{1}H NMR (400 MHz, $CDCl_3$)

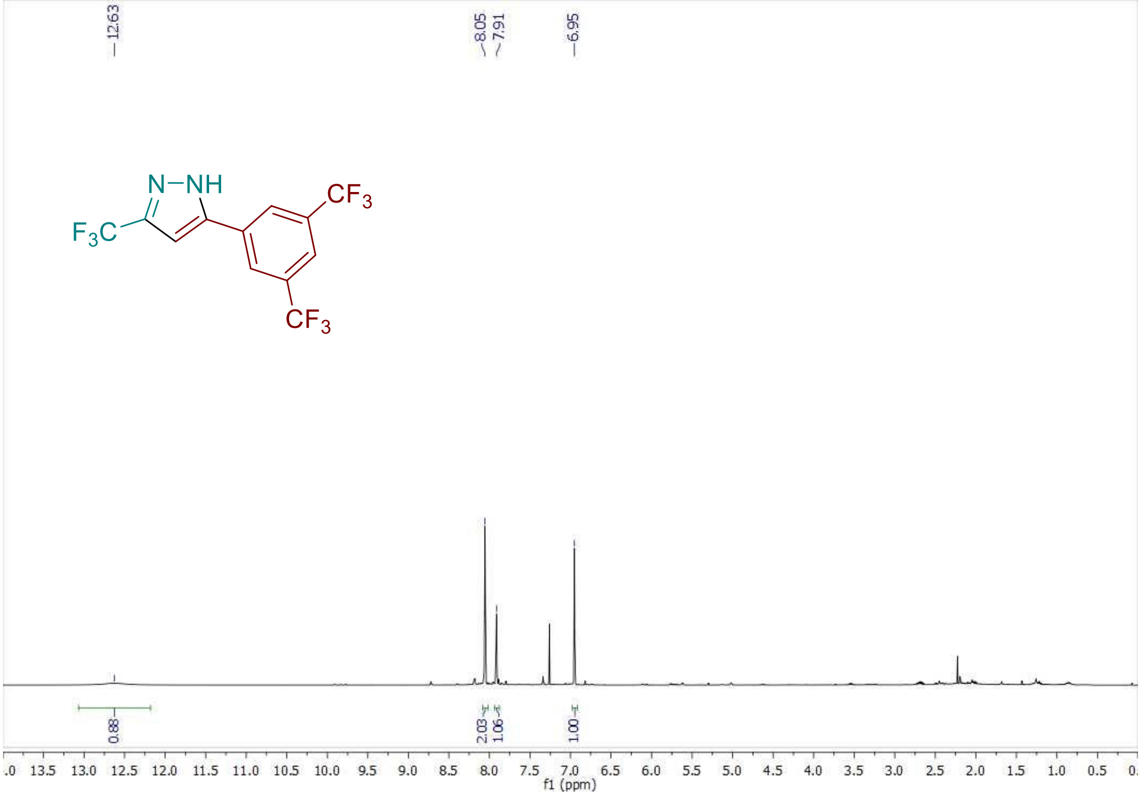

$^{13}C\{^{1}H\}$ NMR (101 MHz, $CDCl_3$)

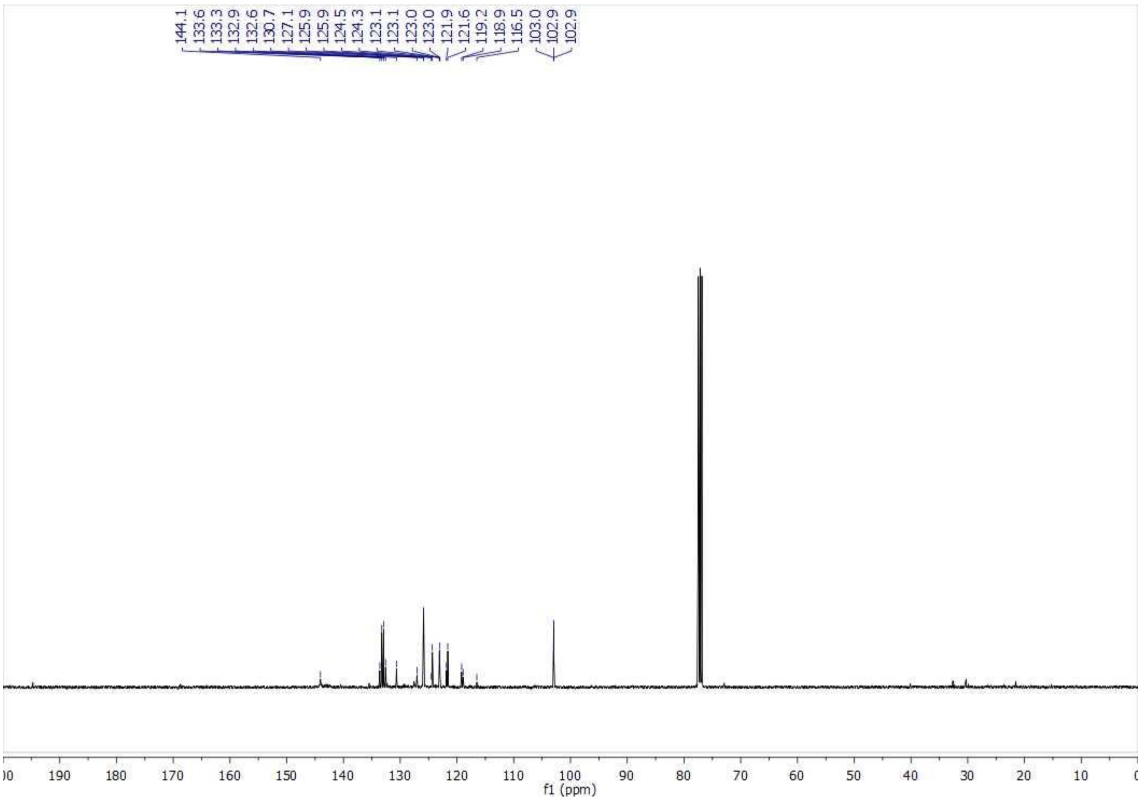

^{19}F NMR (376 MHz, $CDCl_3$)

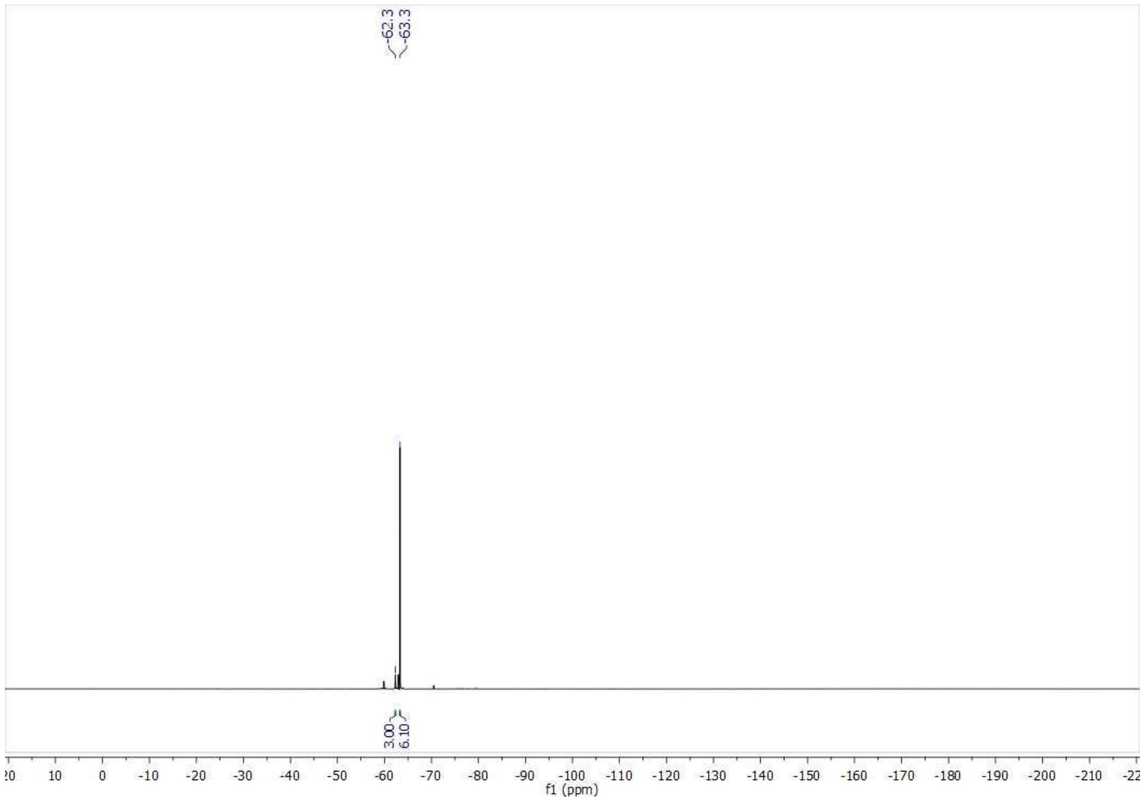

5-(Perfluorophenyl)-3-(trifluoromethyl)-1*H*-pyrazole (5w)

^{1}H NMR (400 MHz, $CDCl_3$)

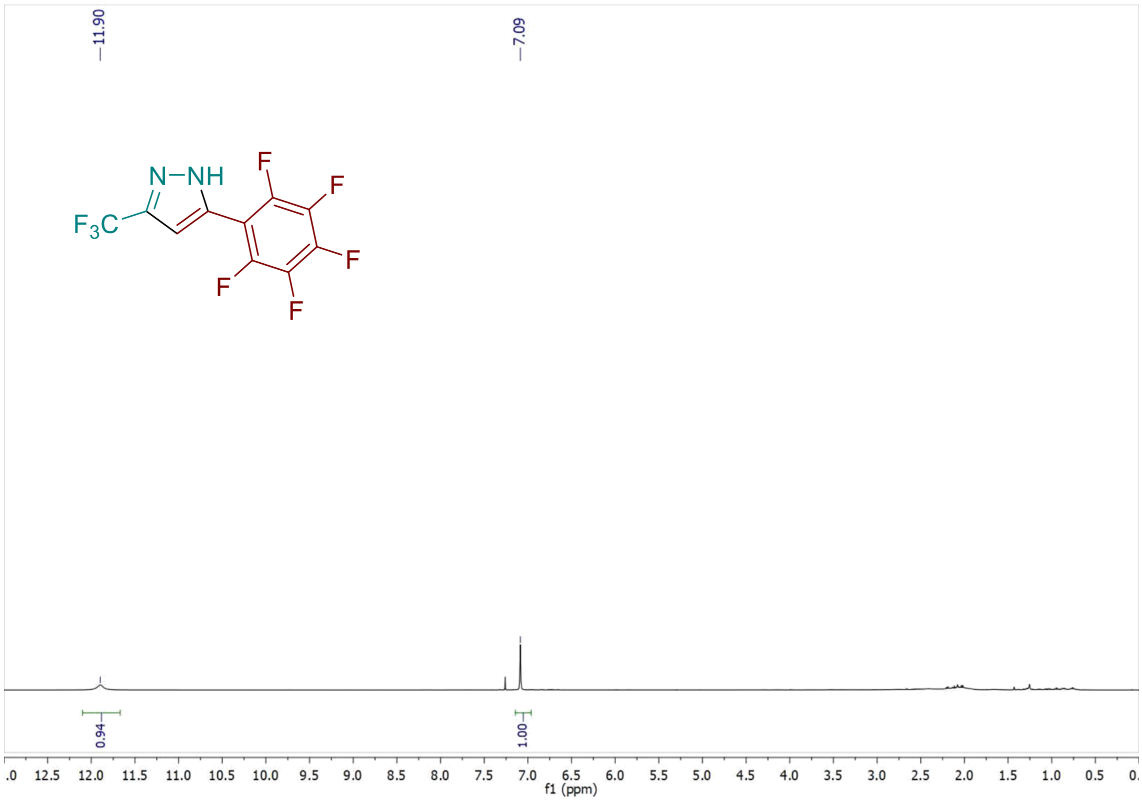

$^{13}C\{^{1}H\}$ NMR (101 MHz, $CDCl_3$)

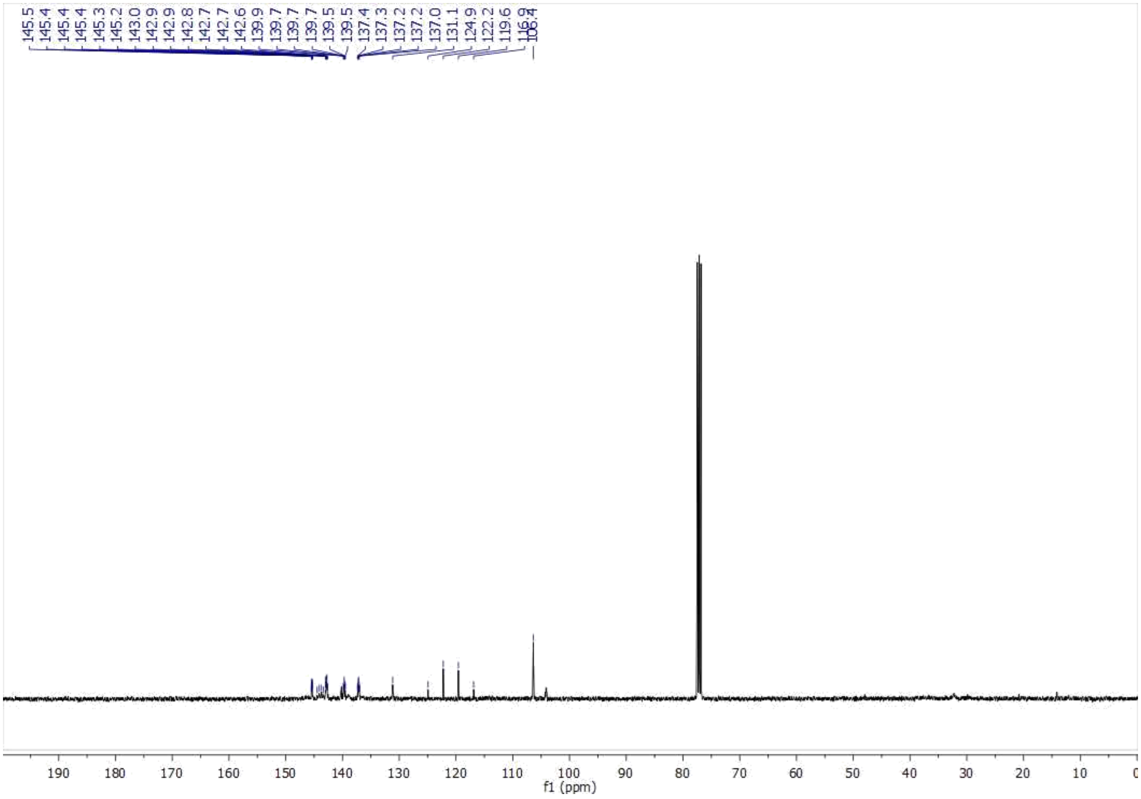

^{19}F NMR (376 MHz, $CDCl_3$)

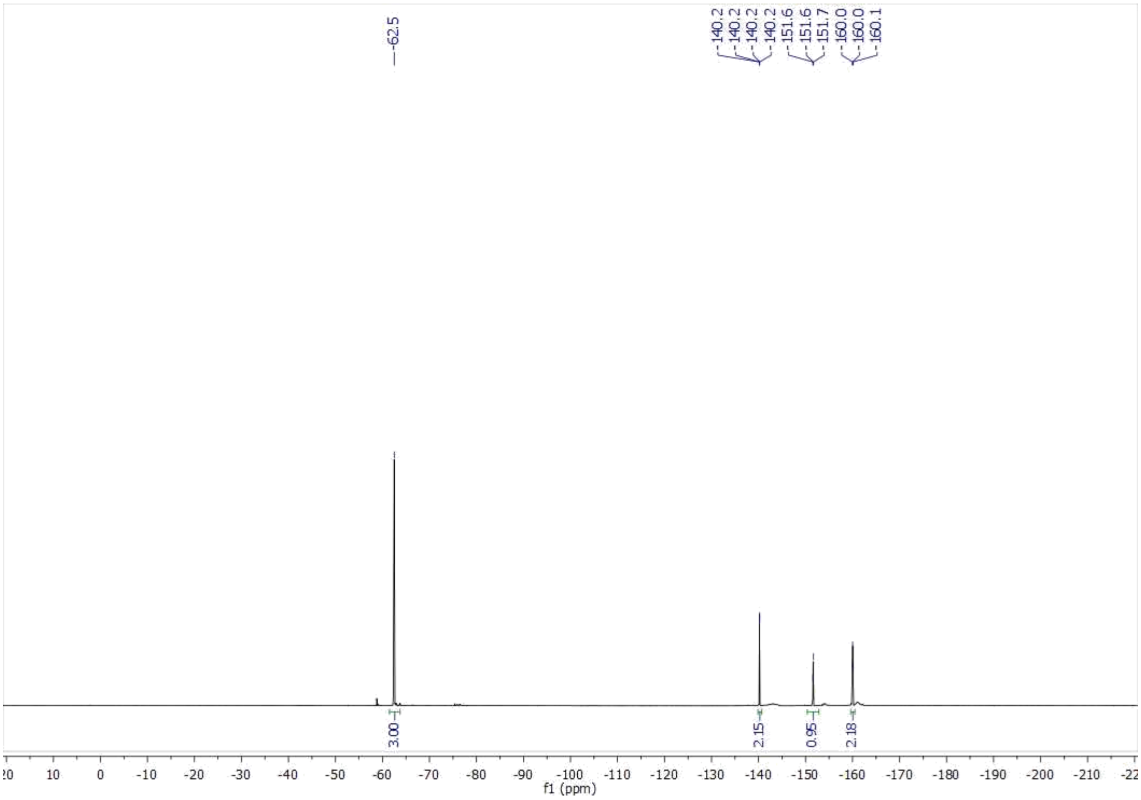

2-(3-(Trifluoromethyl)-1H-pyrazol-5-yl)pyridine (5x)

^{1}H NMR (400 MHz, $CDCl_3$)

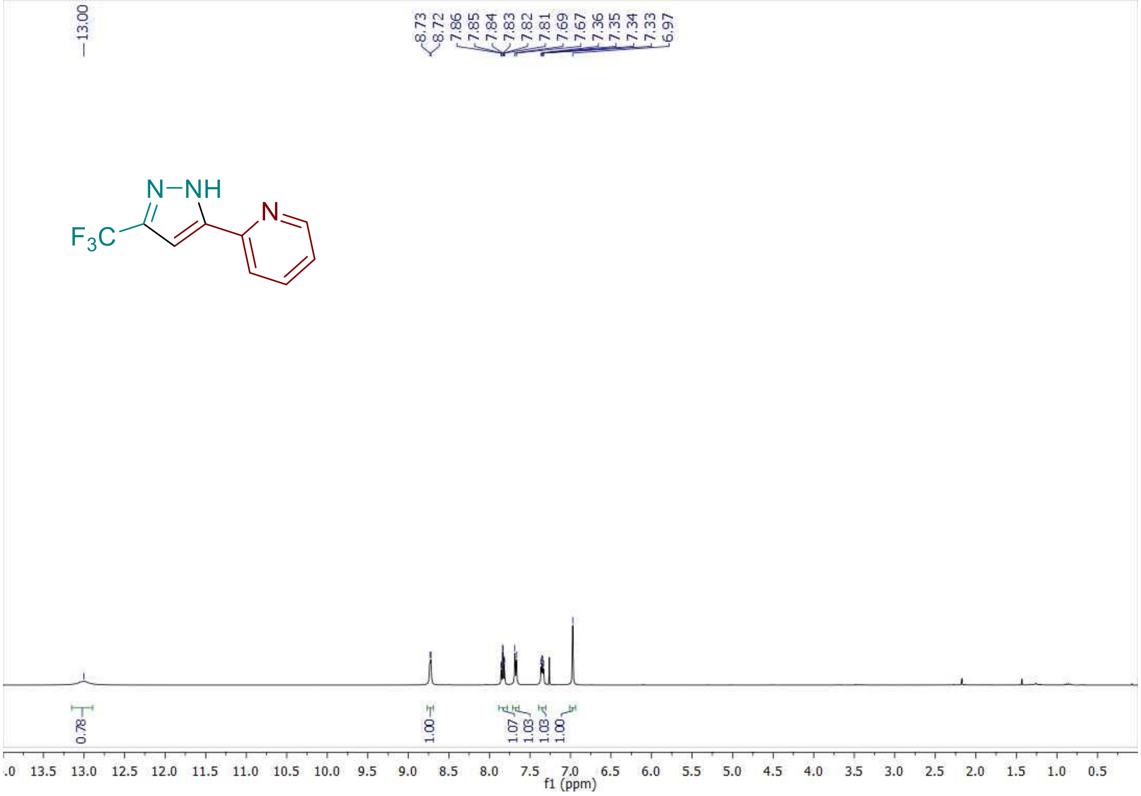

$^{13}C\{^{1}H\}$ NMR (126 MHz, $CDCl_3$)

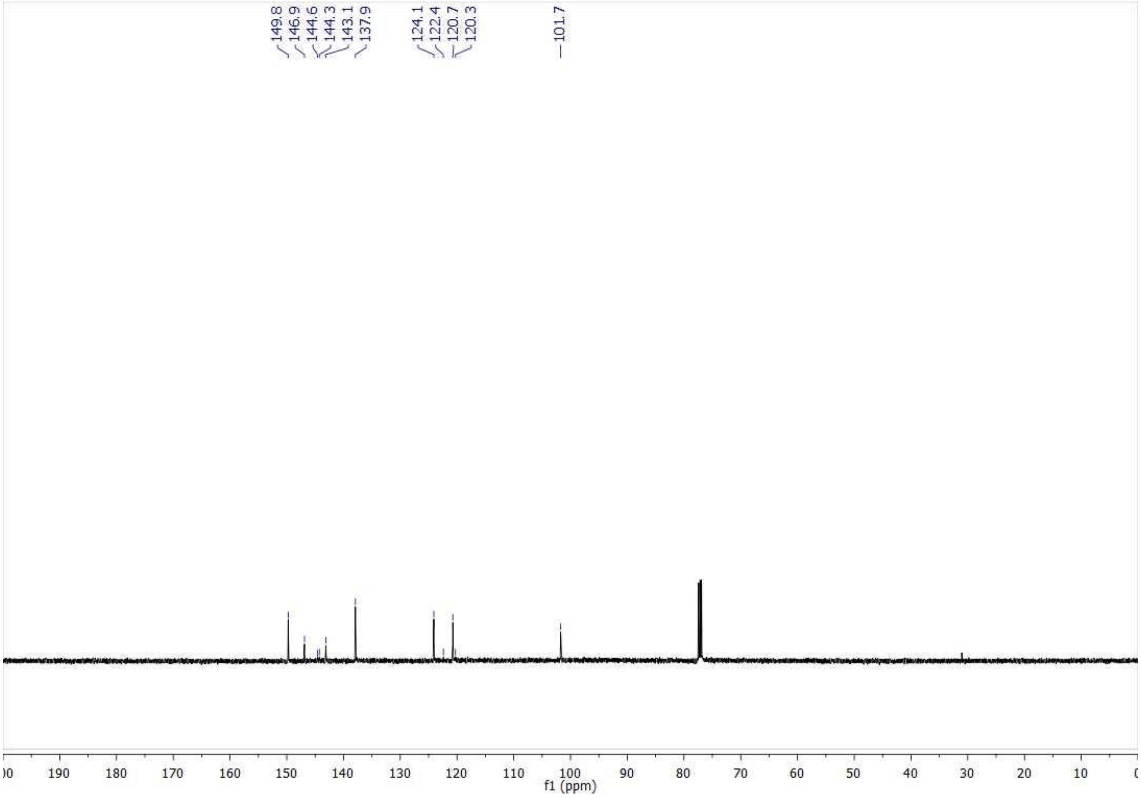

^{19}F NMR (376 MHz, $CDCl_3$)

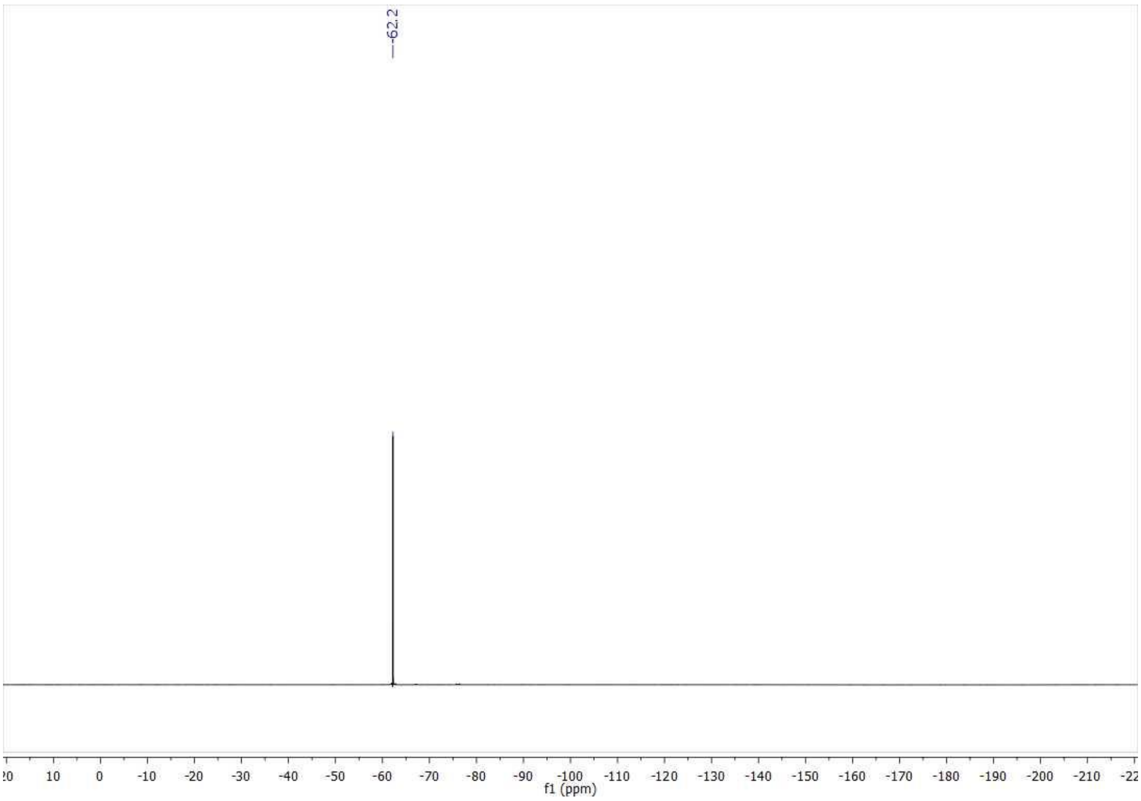

5-(Tiophen-2-yl)-3-(trifluoromethyl)-1*H*-pyrazole (5y)

^{1}H NMR (400 MHz, $CDCl_3$)

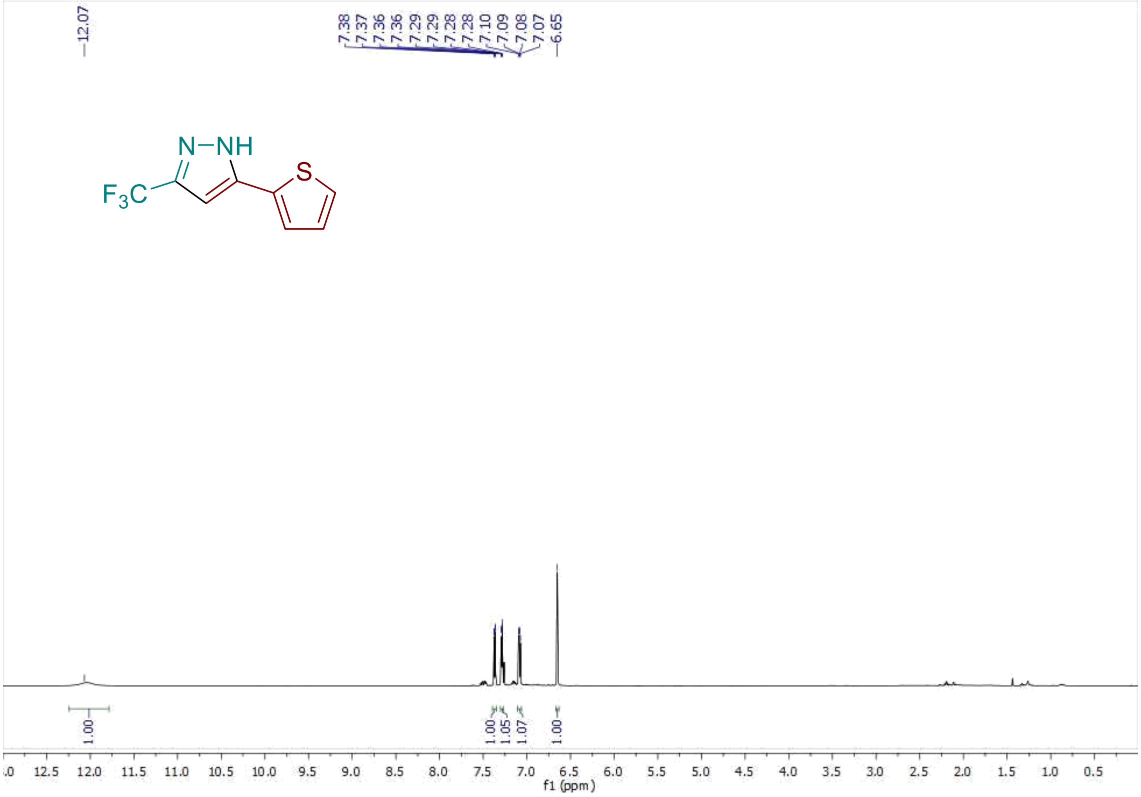

^{13}C{^{1}H} NMR (101 MHz, $CDCl_3$)

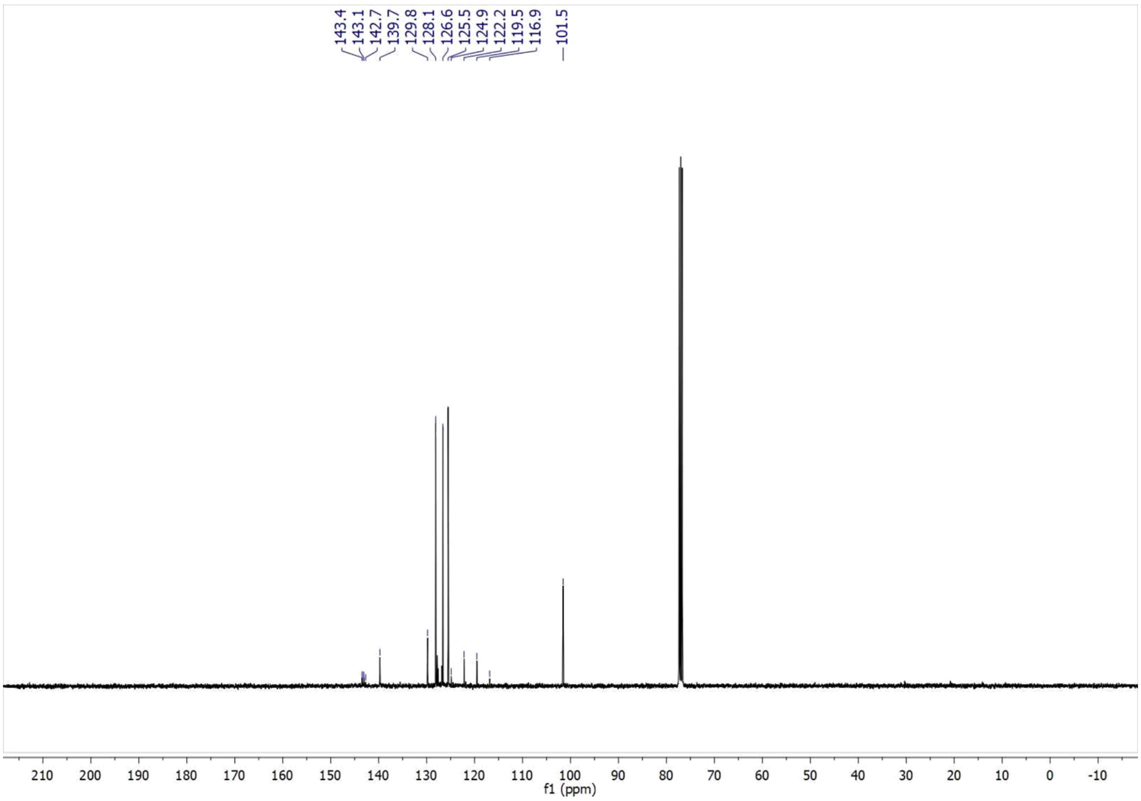

^{19}F NMR (376 MHz, $CDCl_3$)

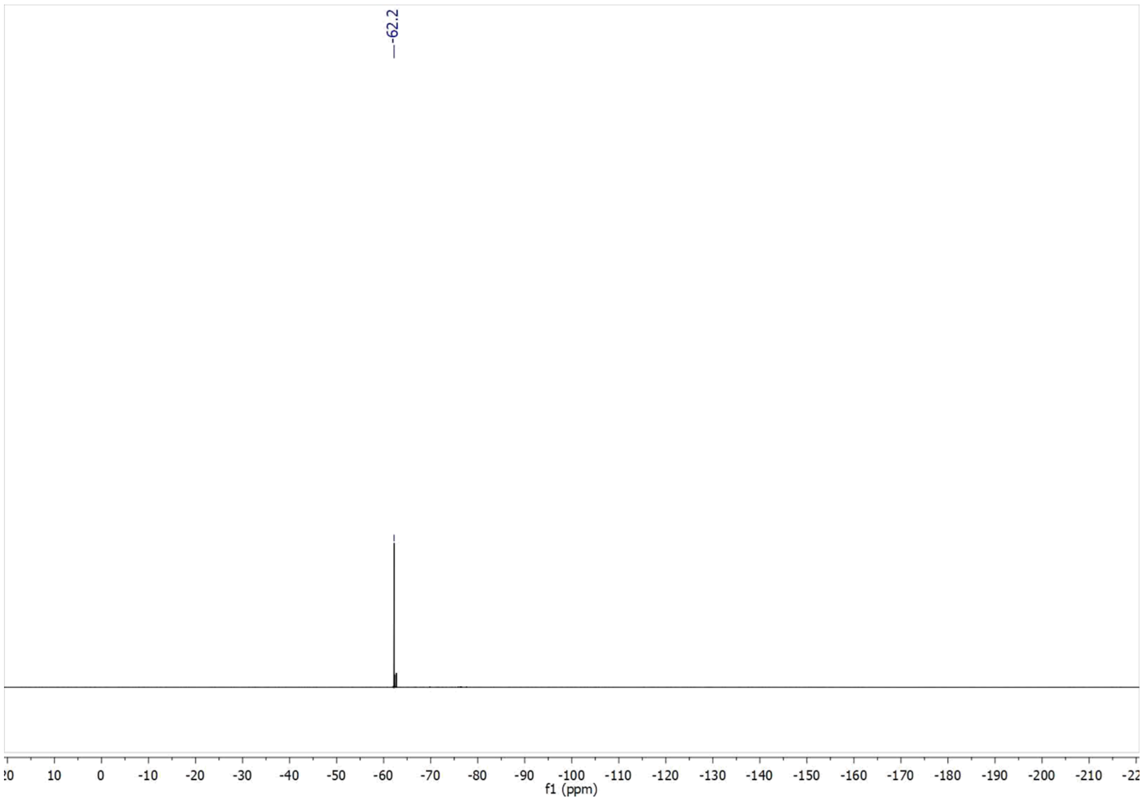

_Tert-B_utyl-3-(trifluoromethyl)-1_H_-pyrazole-5-carboxylate (5z)

^{1}H NMR (400 MHz, $CDCl_3$)

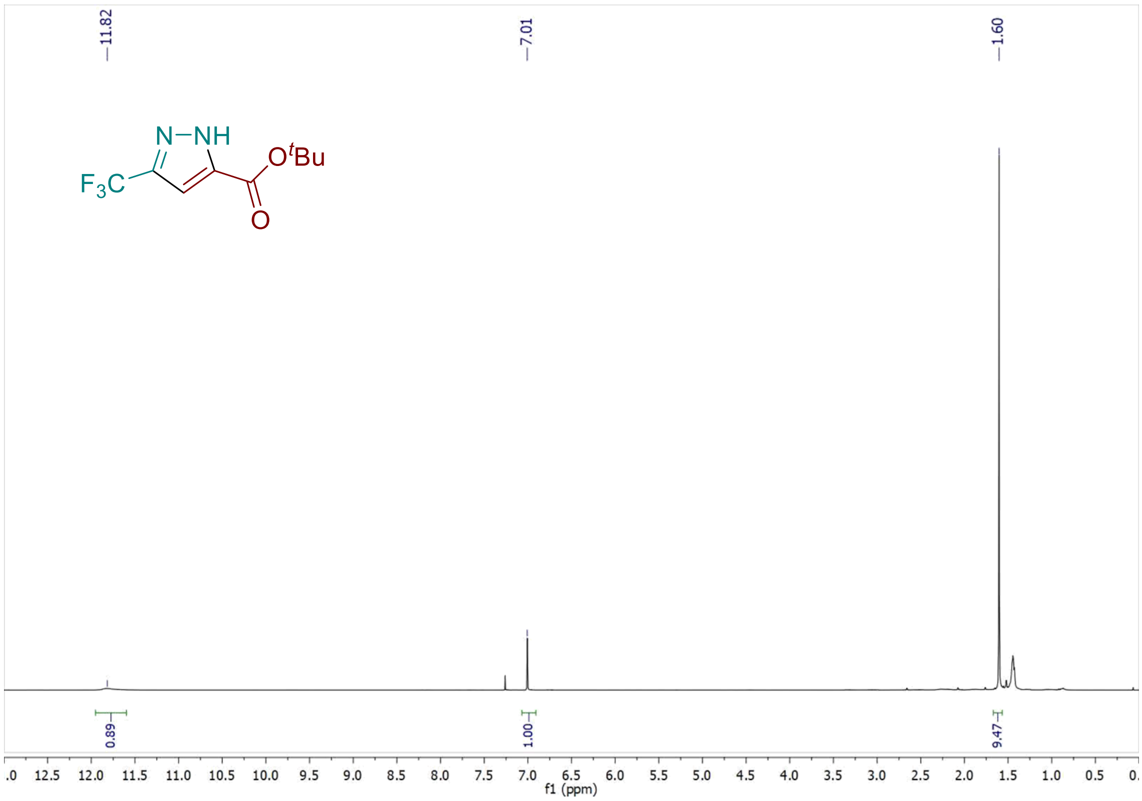

$^{13}C\{^{1}H\}$ NMR (101 MHz, $CDCl_3$)

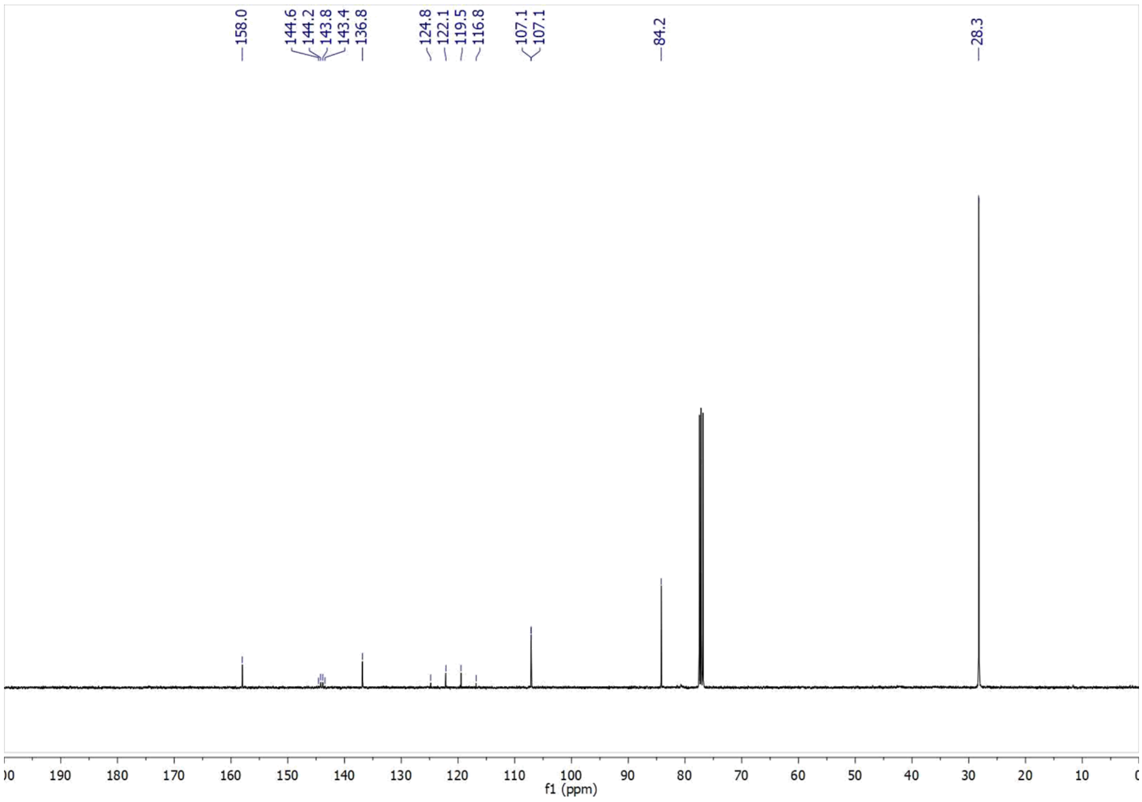

^{19}F NMR (376 MHz, $CDCl_3$)

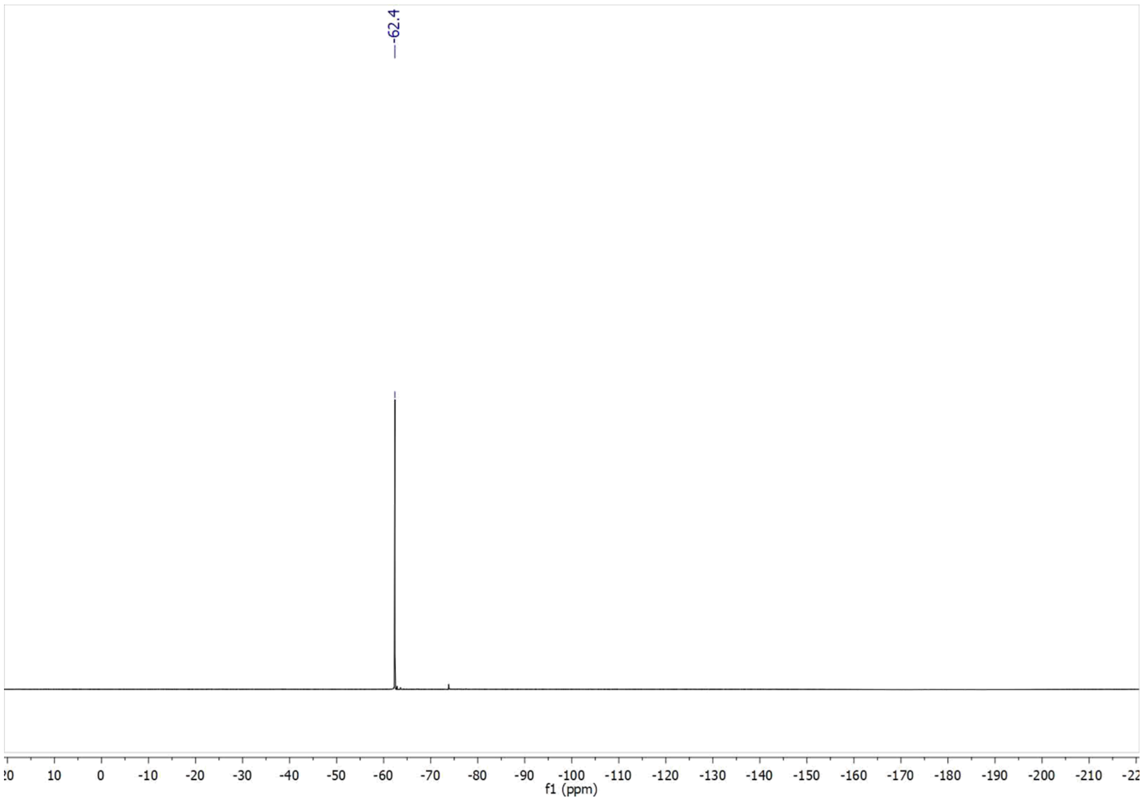

3-(Perfluoroethyl)-5-phenyl-1*H*-pyrazole (5aa)

^{1}H NMR (400 MHz, $CDCl_3$)

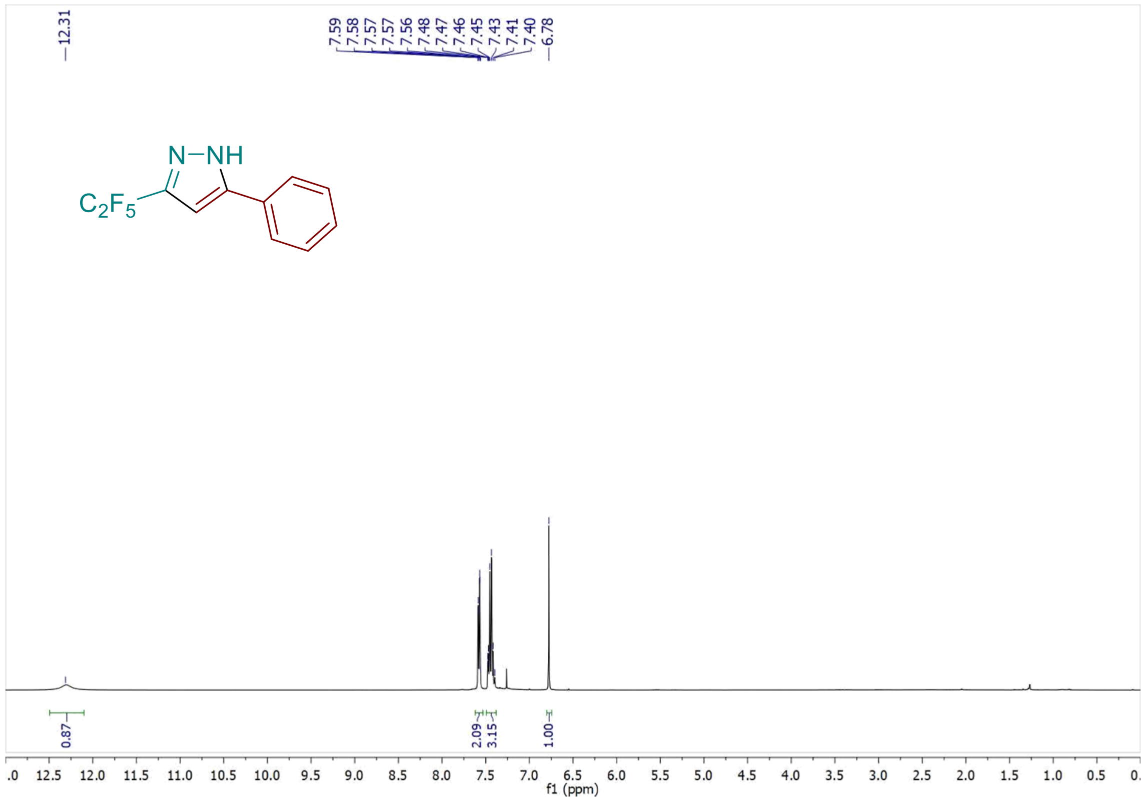

^{13}C{^{1}H} NMR (101 MHz, $CDCl_3$)

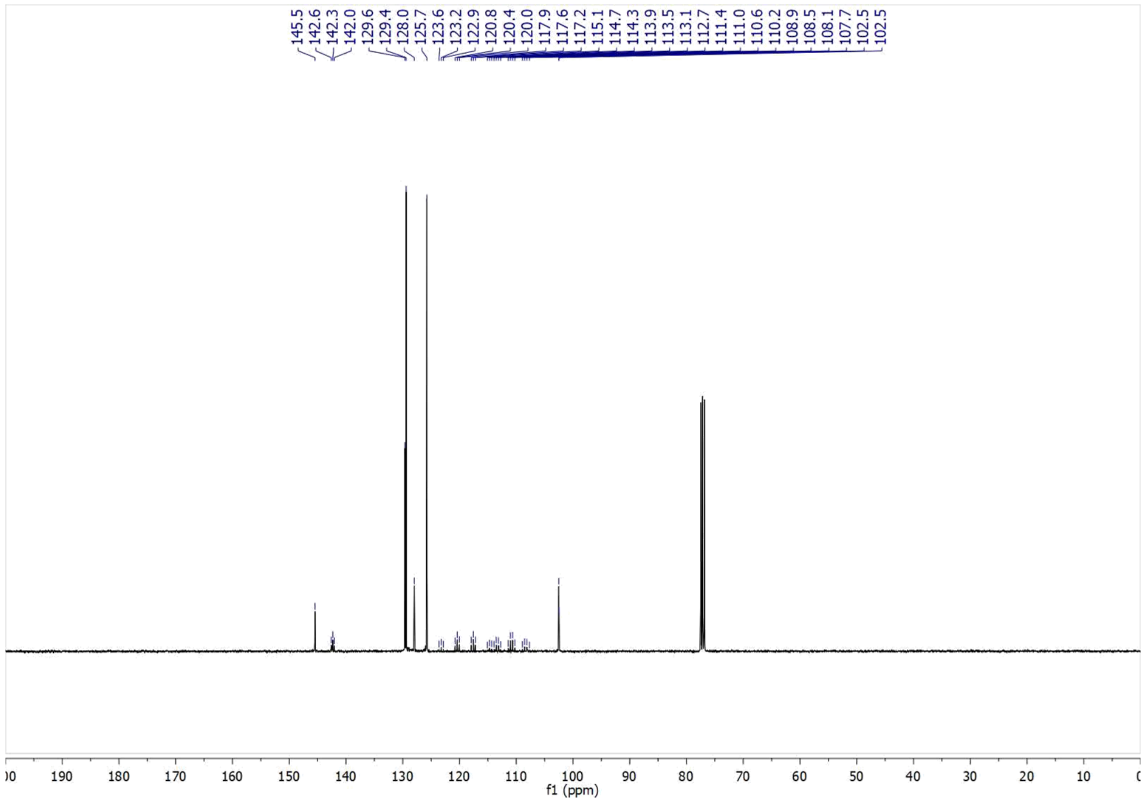

^{19}F NMR (376 MHz, $CDCl_3$)

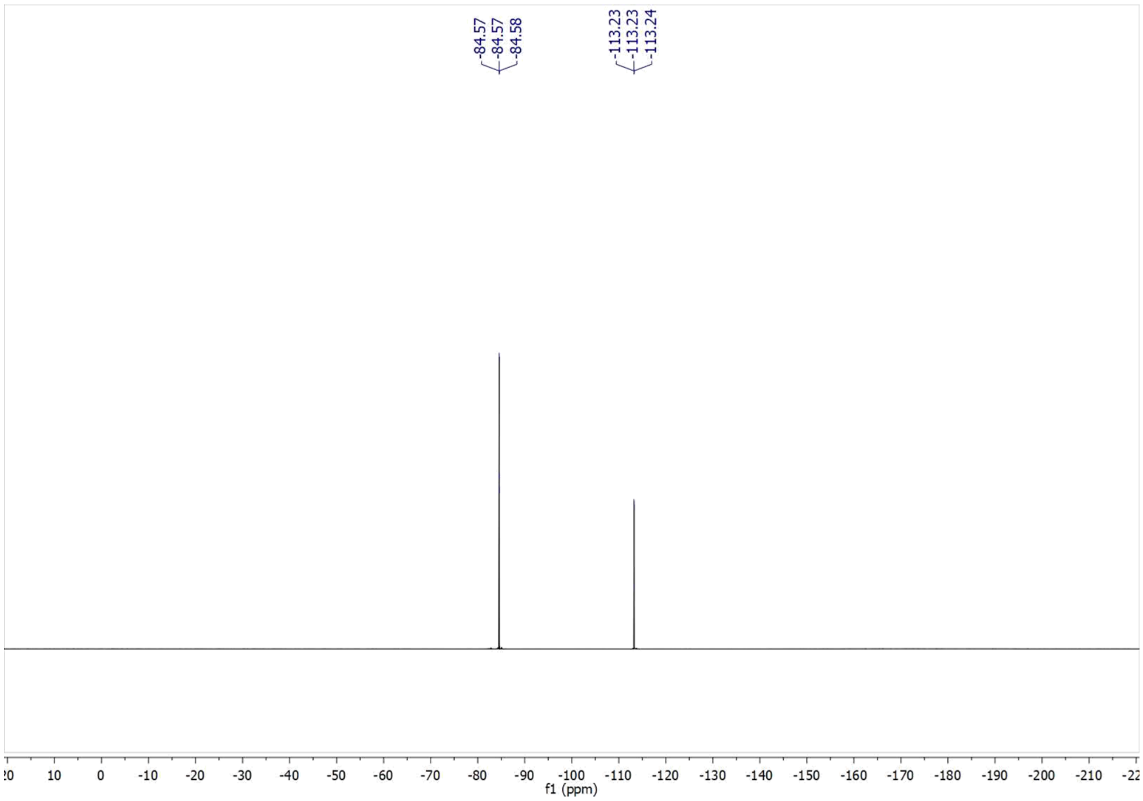

4-Bromo-5-phenyl-3-(trifluoromethyl)-1*H*-pyrazole (6)

^{1}H NMR (400 MHz, $CDCl_3$)

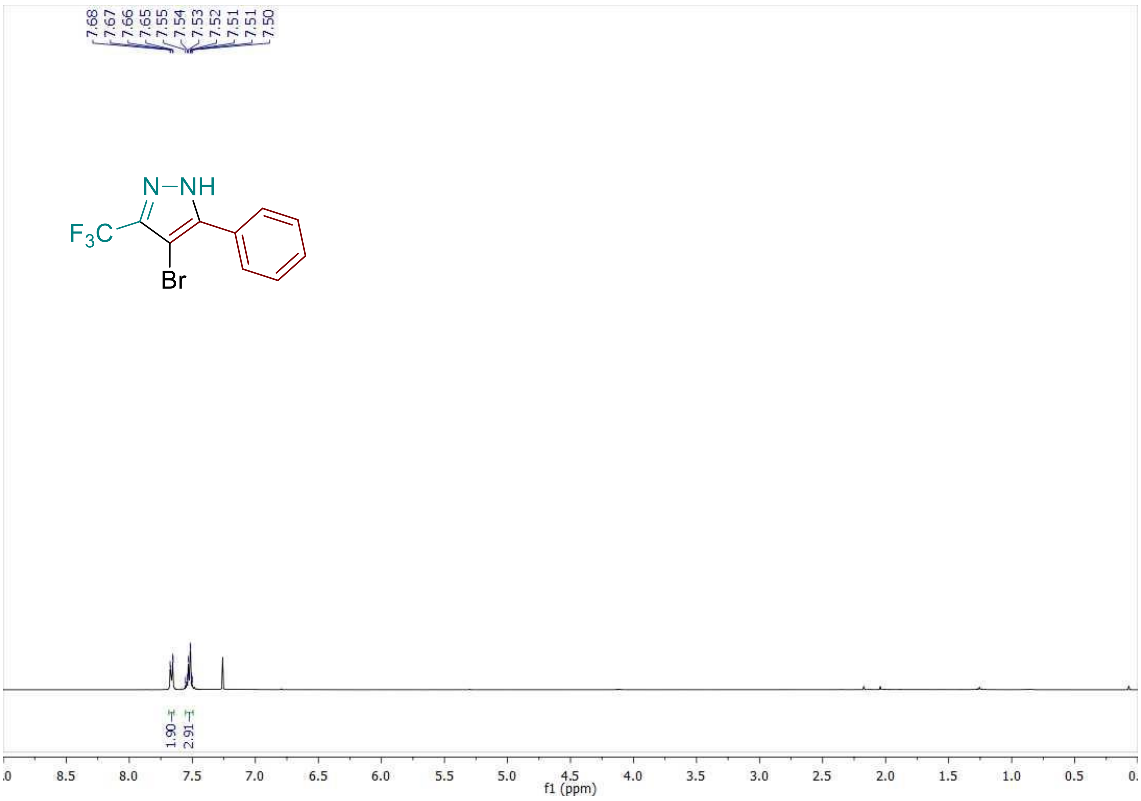

^{13}C{^{1}H} NMR (101 MHz, $CDCl_3$)

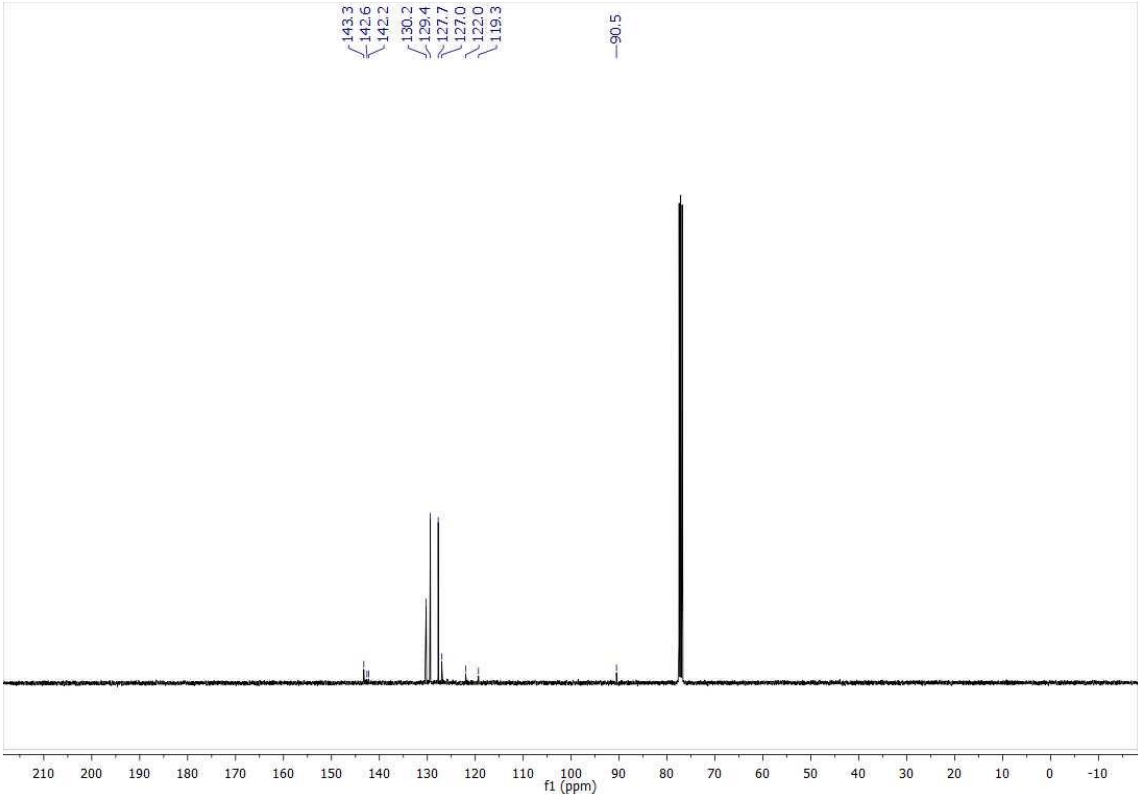

^{19}F NMR (376 MHz, $CDCl_3$)

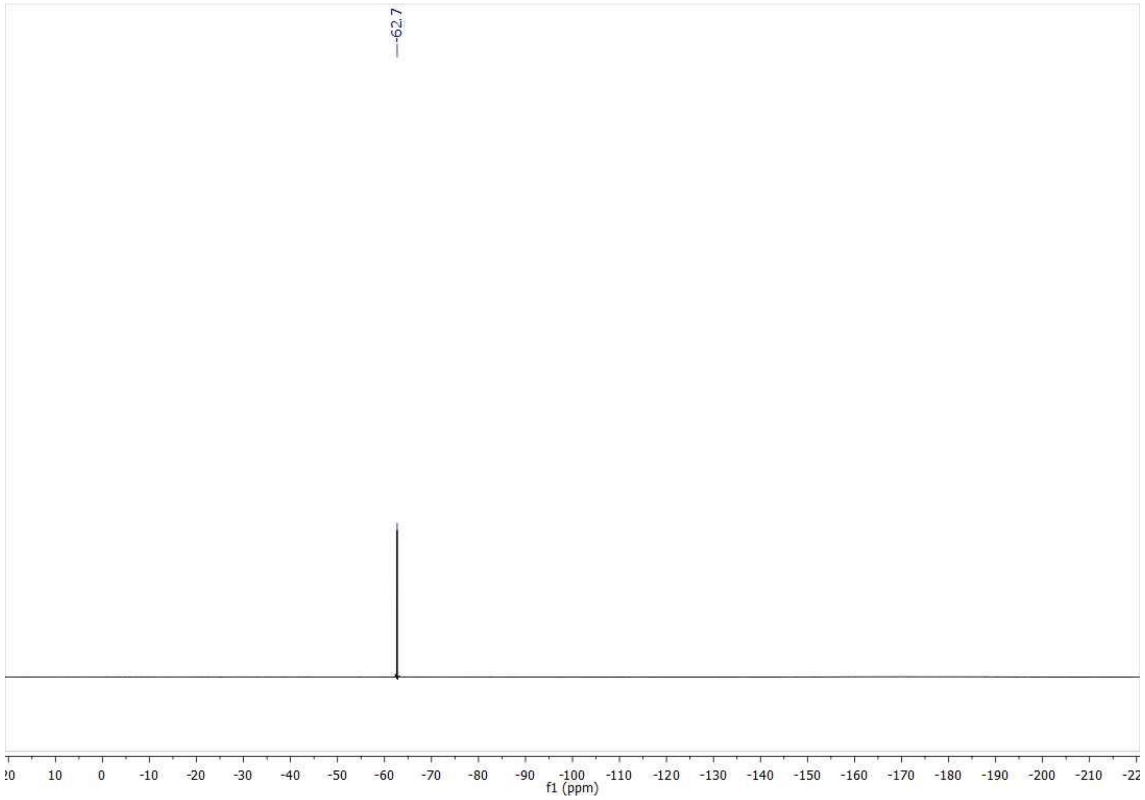

Ethyl 2-(5-([1,1'-biphenyl]-4-yl)-3-(trifluoromethyl)-1H-pyrazol-1-yl)acetate (7)

^{1}H NMR (500 MHz, $CDCl_3$)

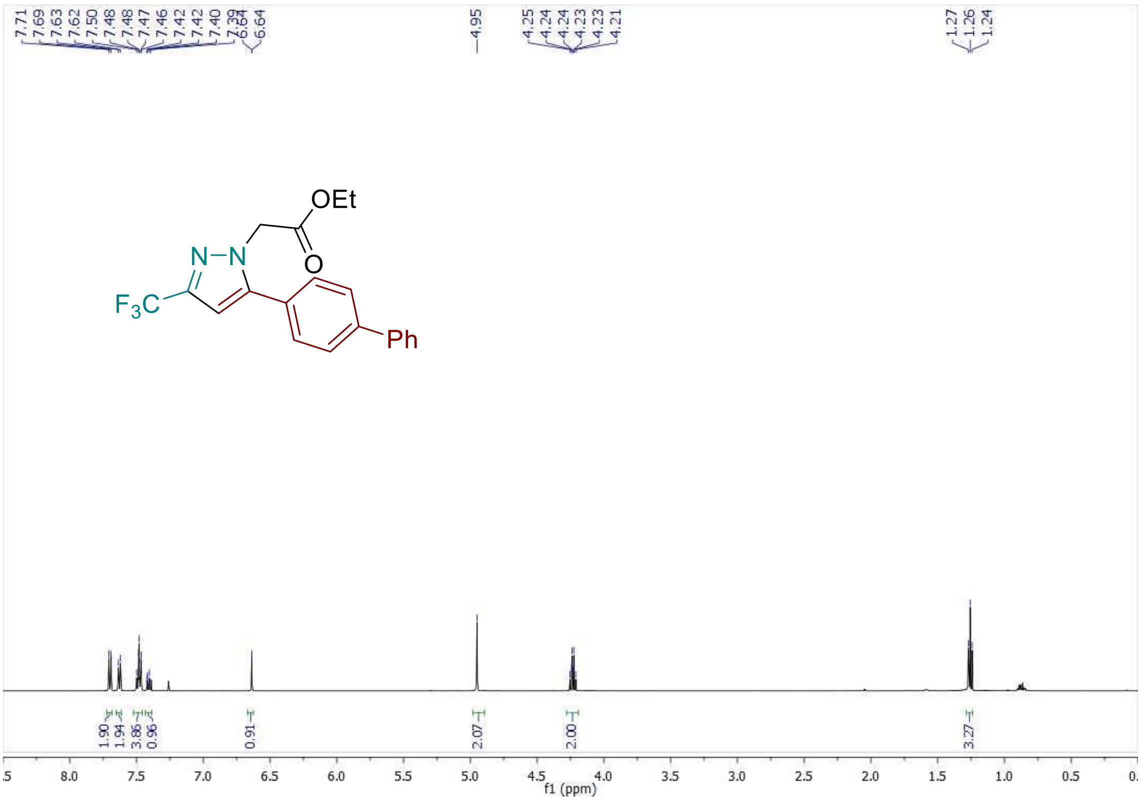

$^{13}C\{^{1}H\}$ NMR (126 MHz, $CDCl_3$)

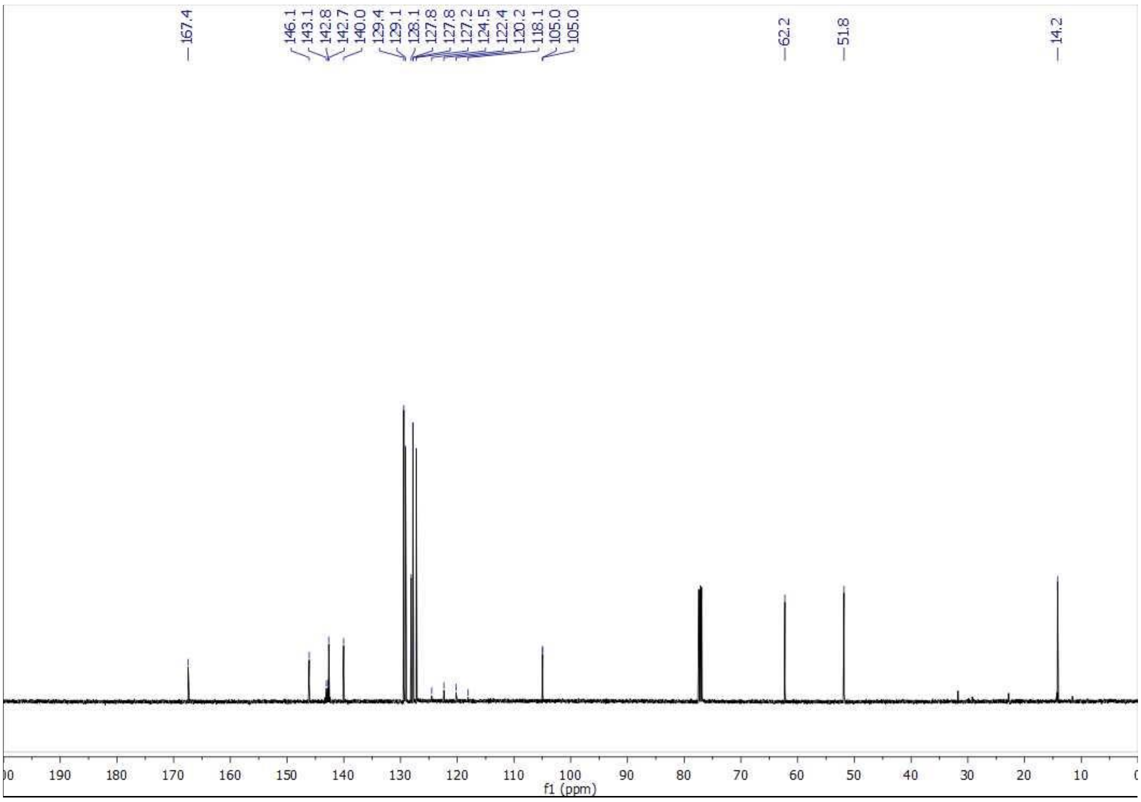

^{19}F NMR (282 MHz, $CDCl_3$)

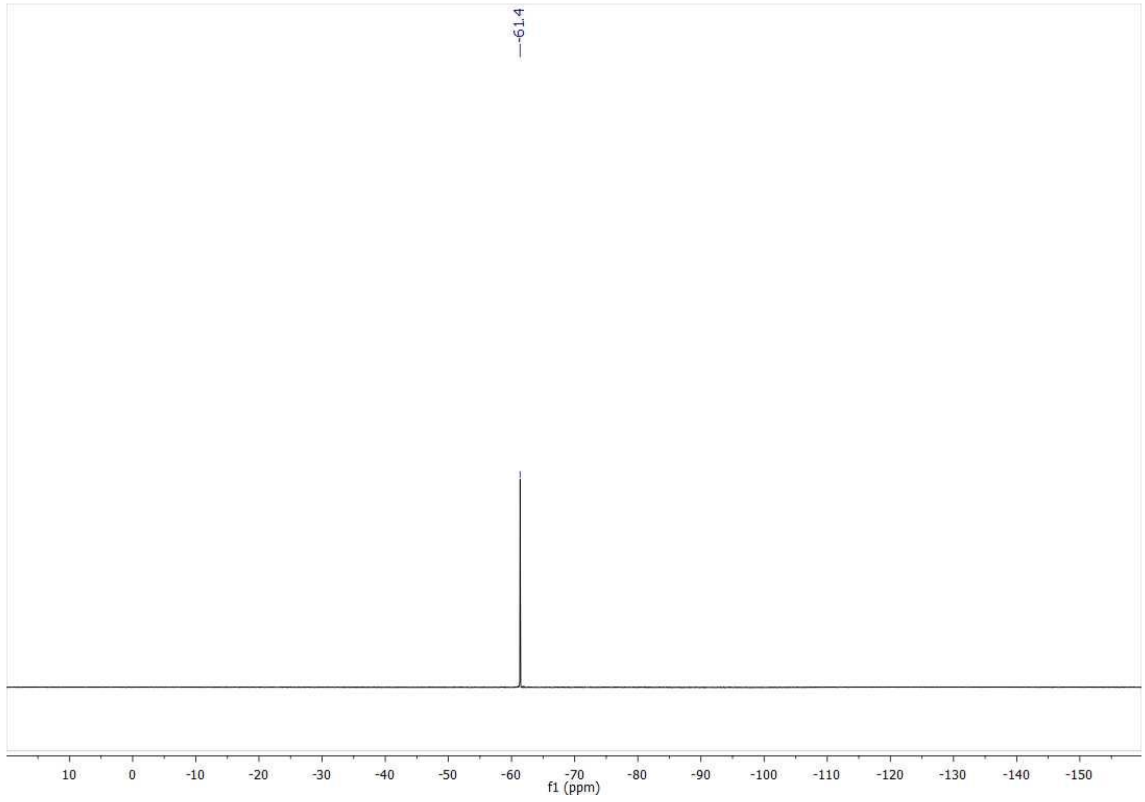

Ethyl 2-(3-([1,1'-biphenyl]-4-yl)-5-(trifluoromethyl)-1H-pyrazol-1-yl)acetate (7´)

^{1}H NMR (500 MHz, $CDCl_3$)

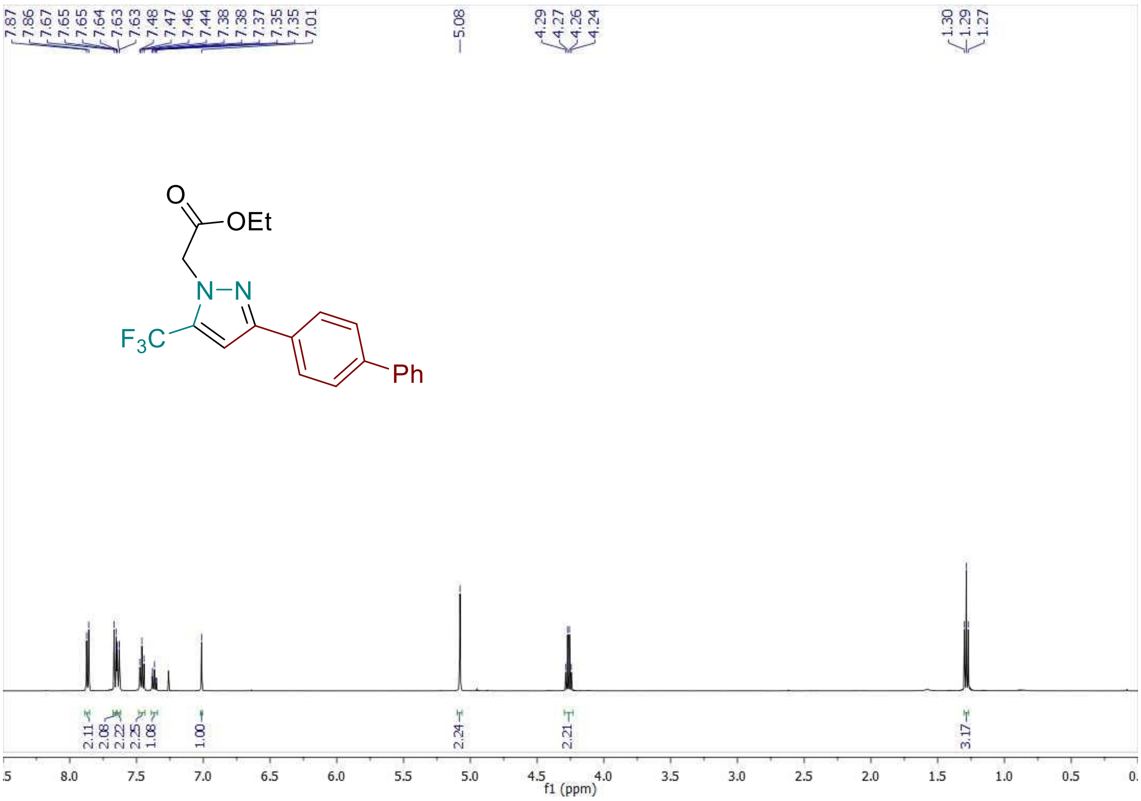

$^{13}C\{^{1}H\}$ NMR (126 MHz, $CDCl_3$)

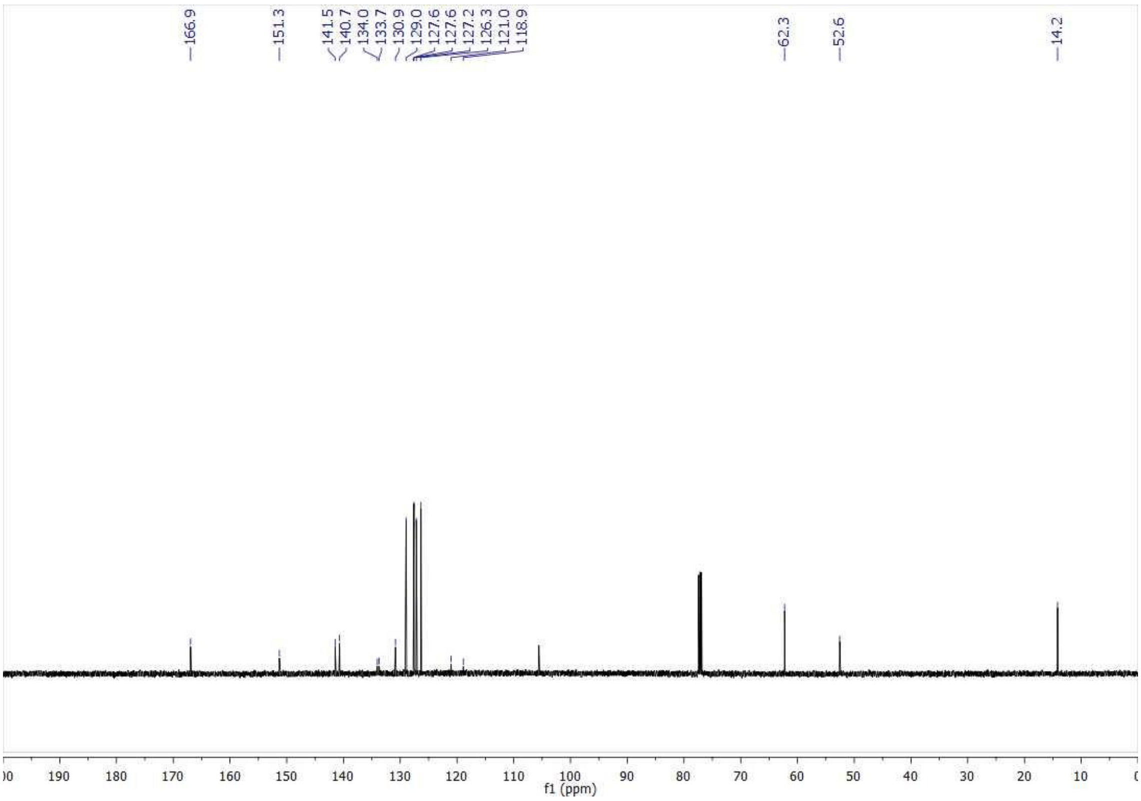

^{19}F NMR (282 MHz, $CDCl_3$)

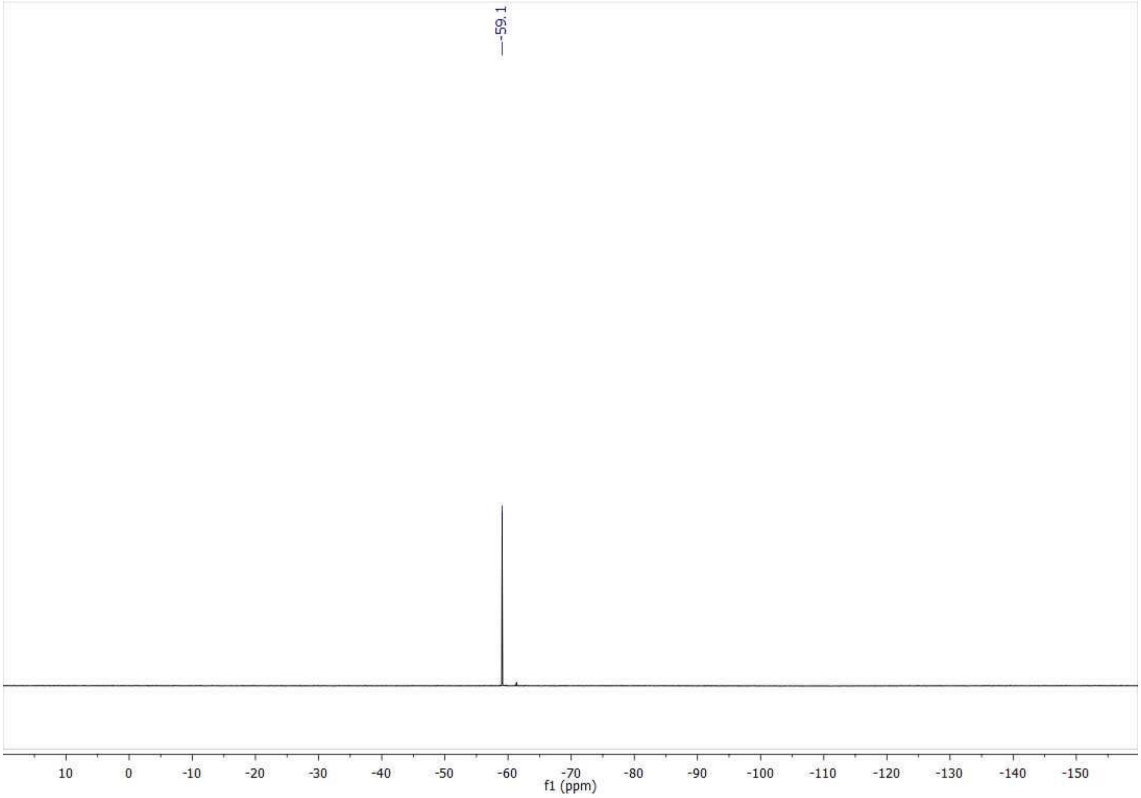

DFT studies

All reported structures were optimized at Density Functional Theory level as implemented in Gaussian 16.[S15] The geometry optimizations were performed using M062X functional[S16] with 6-311++G(d,p) basis set for all the atoms. Solvent effects were considered in all the calculations applying the solvation model based on density (SMD)[S17] using 1,2-dichloroethane as solvent at 298.15 K. Reported energy values correspond to Gibbs Free (G) energies in kcal·mol^{-1}. All structures were optimized without geometrical constraint. Stationary points were characterized by frequency calculations (no negative frequency for minima and one negative frequency for transition states).

[S15] Gaussian 16, Revision C.01; Frisch, M. J. et al. Gaussian, Inc., Wallingford CT, 2016.
[S16] Zhao, Y. & Truhlar, D. G. *Theor. Chem. Acc.* **2008**, *120*, 215-241.
[S17] Marenich, A. V.; Cramer, C. J.; Truhlar, D. G. *J. Phys. Chem. B* **2009**, *113*, 6378-6396.

CARTESIAN COORDINATES OF THE COMPUTED STRUCTURES

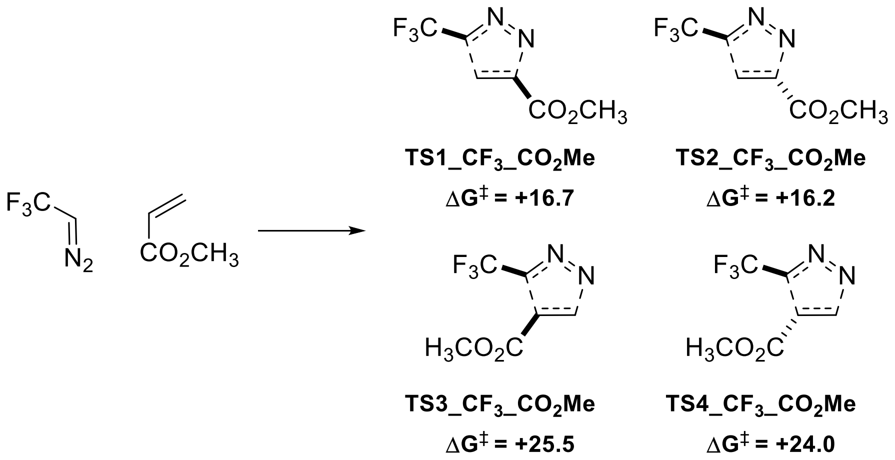

Precursors TS1_CF_3_CO_2Me

C	-0.33705700	-2.01060800	0.39470100
C	1.76713500	0.54487600	1.06273700
N	0.60780900	1.10929000	0.93117800
N	-0.40572500	1.57900300	0.81821500
C	-1.46445700	-1.40720500	0.75776500
H	0.06464600	-1.87729900	-0.60511000
H	0.20279700	-2.65673700	1.07749400
H	2.19180000	0.44832600	2.04936900
H	-1.89617600	-1.52024900	1.74552300
C	2.47262600	0.11608100	-0.16058600
F	1.74217500	0.33950600	-1.26077000
F	3.65068800	0.75127600	-0.33235200
F	2.78087600	-1.19668300	-0.14293100
C	-3.97952800	0.96694500	-0.43282500
H	-4.80714200	1.31144500	0.18210400
H	-3.33532700	1.80392000	-0.70596300
C	-2.17605800	-0.51340400	-0.19088400
O	-1.83686200	-0.28676100	-1.32787600
O	-3.25583200	0.03236100	0.37590500
H	-4.35324700	0.48075100	-1.33437900

E(RM062X) = -792.208254510
Zero-point correction= 0.135820 (Hartree/Particle)
Thermal correction to Energy= 0.150391
Thermal correction to Enthalpy= 0.151335
Thermal correction to Gibbs Free Energy= 0.091768
Sum of electronic and zero-point Energies= -792.072434
Sum of electronic and thermal Energies= -792.057864
Sum of electronic and thermal Enthalpies= -792.056920
Sum of electronic and thermal Free Energies= -792.116486

TS1_CF_3_CO_2Me

C	0.01485000	0.44935700	1.58216900
C	1.50612600	0.92929800	0.16692500
N	0.66767100	1.26554300	-0.83187800
N	-0.40246000	1.44375300	-1.15009300
C	-1.20262700	0.80814800	1.04214000
H	0.26327000	-0.60718600	1.62366000
H	0.46152800	1.07476800	2.34604300
H	2.06838700	1.77226400	0.56015700
H	-1.65032300	1.77481500	1.23374300
C	2.33138600	-0.29227800	-0.11051700
F	1.61042300	-1.26078100	-0.68131600
F	3.38177100	-0.06024900	-0.91718100
F	2.82479600	-0.76056900	1.04112900
C	-4.11293100	-0.61425200	-0.63613500
H	-4.30435300	-1.50313200	-0.03362500
H	-5.03807300	-0.06626300	-0.79974300
C	-2.02485300	-0.19243200	0.36197700
O	-1.68088200	-1.32274800	0.08275900
O	-3.24474400	0.28599100	0.05623400
H	-3.67651500	-0.90893200	-1.59142700

E(RM062X) = -792.185890292
Zero-point correction= 0.136906 (Hartree/Particle)
Thermal correction to Energy= 0.149758
Thermal correction to Enthalpy= 0.150703
Thermal correction to Gibbs Free Energy= 0.095949
Sum of electronic and zero-point Energies= -792.048985
Sum of electronic and thermal Energies= -792.036132
Sum of electronic and thermal Enthalpies= -792.035188
Sum of electronic and thermal Free Energies= -792.089941

Precursors TS2_CF_3_CO_2Me

C	0.44907400	1.89044800	-0.53615800
C	-1.37726200	-0.57918500	0.88130000
N	-0.41187700	-1.26415200	0.35732500
N	0.43775000	-1.83488800	-0.10630300
C	1.48644200	1.06956600	-0.68276800
H	-0.04625300	2.32426100	-1.39762300
H	0.08200400	2.15325600	0.45016500
H	-1.39783800	-0.43224400	1.94952700
C	-2.41821100	-0.05797100	-0.02700600
F	-2.09026000	-0.24162100	-1.31459700
F	-3.62235400	-0.64050900	0.15415500
F	-2.63171800	1.25906300	0.15161000
C	3.67009700	-0.68174000	-0.92204300
H	2.89535300	-1.19601500	-1.49265800
H	4.03208200	0.19254000	-1.46476800
H	1.85825700	0.81482000	-1.66625300
C	2.12843600	0.48453400	0.53014300
O	3.19033100	-0.31806100	0.37782400
O	1.73020400	0.69973200	1.65032400
H	4.49853000	-1.36301700	-0.74204400

E(RM062X) = -792.200527852
Zero-point correction= 0.136024 (Hartree/Particle)
Thermal correction to Energy= 0.150510
Thermal correction to Enthalpy= 0.151455
Thermal correction to Gibbs Free Energy= 0.091826
Sum of electronic and zero-point Energies= -792.064504
Sum of electronic and thermal Energies= -792.050017
Sum of electronic and thermal Enthalpies= -792.049073
Sum of electronic and thermal Free Energies= -792.108702
Frequency: -460.8996

TS2_CF3_CO_2Me

C	0.12077400	1.03529100	-0.89268100
C	-1.22050900	0.41608100	0.59472400
N	-0.47101700	-0.58644200	1.09333000
N	0.48973500	-1.17639900	1.05712600
C	1.24972400	0.24390300	-0.79632800
H	-0.54547200	0.88246500	-1.73522900
H	0.15527200	2.04098600	-0.48898600
H	-1.35712800	1.24377500	1.28536200
C	-2.47854800	-0.06129100	-0.07211400
F	-2.24154500	-1.10431400	-0.87742800
F	-3.43419700	-0.44513700	0.78921900
F	-2.99077700	0.92777500	-0.80945200
C	3.63385900	-1.20529700	-0.66952300
H	2.88636600	-1.96993800	-0.44867900
H	3.59727000	-0.93327100	-1.72596200
H	1.37595400	-0.60800400	-1.44776500
C	2.31434100	0.65566500	0.12036500
O	3.45745900	-0.05924800	0.16453900
O	2.21931300	1.60460600	0.87443900
H	4.62336400	-1.59001900	-0.43146800

SCF Done: E(RM062X) = -792.179885854
Zero-point correction= 0.137334 (Hartree/Particle)
Thermal correction to Energy= 0.149969
Thermal correction to Enthalpy= 0.150913
Thermal correction to Gibbs Free Energy= 0.096954
Sum of electronic and zero-point Energies= -792.042552
Sum of electronic and thermal Energies= -792.029917
Sum of electronic and thermal Enthalpies= -792.028973
Sum of electronic and thermal Free Energies= -792.082932
Frequency: -466.5239

Precursors TS3_CF_3_CO_2Me

C	-0.53151400	2.56319800	-0.16126700
C	1.36511100	-0.32783000	0.86880000
N	2.34510600	0.51304900	0.75554800
N	3.18067000	1.25646100	0.65925900
C	0.70183100	2.88980100	-0.53101800
H	-1.01609600	3.10249600	0.64869600
H	0.68447700	-0.22947100	1.69952000
H	1.24413100	3.69472100	-0.04680900
C	1.21463500	-1.35559100	-0.17618100
F	1.88477100	-1.03723400	-1.29573500
F	1.65407100	-2.58183000	0.18814300
F	-0.07842100	-1.52743600	-0.50743600
C	-1.34171200	1.49109600	-0.82419600
H	-0.74344500	0.93145900	-1.54984300
H	-2.17939200	1.92705700	-1.37977000
C	-3.31075700	-1.38901600	0.34824000
H	-3.94561700	-0.94727800	1.11696500
H	-3.90418200	-2.00498300	-0.32284600
H	-2.52698200	-1.98536300	0.81633600
H	1.20855500	2.36391500	-1.33661600
O	-2.73413100	-0.36505100	-0.47136200
C	-1.93076700	0.50027500	0.15036900
O	-1.72169900	0.47102400	1.33854800

E(RM062X) = -831.513675390
Zero-point correction= 0.163679 (Hartree/Particle)
Thermal correction to Energy= 0.179842
Thermal correction to Enthalpy= 0.180787
Thermal correction to Gibbs Free Energy= 0.116716
Sum of electronic and zero-point Energies= -831.349996
Sum of electronic and thermal Energies= -831.333833
Sum of electronic and thermal Enthalpies= -831.332889
Sum of electronic and thermal Free Energies= -831.396959

TS3_CF_3_CO_2Me

C	0.49306700	-1.78455900	-0.26594400
C	1.38101200	0.25982200	-0.75169400
N	2.60471800	-0.08579600	-0.29272600
N	3.17503700	-0.95962200	0.17394200
C	1.59441300	-2.44510400	0.23202900
H	0.24332000	-1.91379600	-1.31313900
H	1.32671300	0.35311200	-1.83331000
H	2.19598100	-3.06747000	-0.41983200
C	0.82069400	1.43679500	-0.01177500
F	0.97807200	1.30837700	1.31498200
F	1.38396600	2.61492500	-0.34672400
F	-0.48916600	1.56206600	-0.25711300
C	-0.67164000	-1.42193200	0.62679800
H	-0.38161500	-0.79608300	1.47301800
H	-1.09805200	-2.33499200	1.06063500
C	-3.80277400	0.44776000	0.11526800
H	-4.40122200	-0.28332100	-0.42925100
H	-4.37744700	0.87071800	0.93551400
H	-3.47683200	1.23577300	-0.56461000
H	1.69493100	-2.62399300	1.29801200
O	-2.66871800	-0.18510600	0.72067600
C	-1.80911300	-0.76927300	-0.11750000
O	-1.96551300	-0.79381700	-1.31304600

E(RM062X) = -831.479319252
Zero-point correction= 0.165250 (Hartree/Particle)
Thermal correction to Energy= 0.179241
Thermal correction to Enthalpy= 0.180185
Thermal correction to Gibbs Free Energy= 0.123041
Sum of electronic and zero-point Energies= -831.314070
Sum of electronic and thermal Energies= -831.300078
Sum of electronic and thermal Enthalpies= -831.299134
Sum of electronic and thermal Free Energies= -831.356278
Frequency: -504.1080

Precursors TS4_CF_3_CO_2Me

C	-0.64825000	0.81580500	-0.74721500
C	2.22413900	0.15052600	0.99538000
N	2.50121000	1.31728600	0.50235700
N	2.72489100	2.32975300	0.07336000
C	-0.15278000	1.95882800	-1.20918700
H	2.07161600	0.05429000	2.05855600
H	-0.21574200	2.87823500	-0.63386500
C	2.20263000	-1.00071700	0.07340500
F	2.33910100	-0.61995900	-1.20445300
F	3.18650200	-1.89317600	0.32114400
F	1.05900100	-1.70751000	0.16611900
H	0.33589000	2.00999000	-2.17652300
C	-1.32681000	0.68648200	0.59043500
H	-0.83700500	-0.09728900	1.17902600
H	-1.28317600	1.62297300	1.14596400
C	-4.27038000	-1.28324800	-0.44700600
H	-4.85330300	-0.52943000	-0.97690300
H	-4.19251300	-2.18889100	-1.04317500
H	-4.73702300	-1.50099500	0.51407500
H	-0.57642400	-0.09090800	-1.34171900
C	-2.77914500	0.29322100	0.45725600
O	-3.70684000	0.89844200	0.93332000
O	-2.92477200	-0.82679600	-0.25571600

E(RM062X) = -831.510824841
Zero-point correction= 0.163773 (Hartree/Particle)
Thermal correction to Energy= 0.180089
Thermal correction to Enthalpy= 0.181033
Thermal correction to Gibbs Free Energy= 0.114465
Sum of electronic and zero-point Energies= -831.347052
Sum of electronic and thermal Energies= -831.330736
Sum of electronic and thermal Enthalpies= -831.329792
Sum of electronic and thermal Free Energies= -831.396360

TS4_CF_3_CO_2Me

C	0.36196100	0.42322200	0.32912300
C	-1.60209300	0.29651300	-0.70361600
N	-1.99027500	1.52627600	-0.32772100
N	-1.66524700	2.43863400	0.27726800
C	0.30516400	1.63814000	0.97203500
H	-1.43790400	0.17170000	-1.77049600
H	0.82374500	2.50071800	0.56552100
C	-2.36866400	-0.80688900	-0.04772600
F	-2.53071900	-0.58536600	1.26410500
F	-3.59980200	-1.00812500	-0.55549800
F	-1.70207300	-1.95913100	-0.19420600
H	-0.01037700	1.70119800	2.00682900
C	1.23650100	0.24736900	-0.90166000
H	0.95838500	-0.65227400	-1.45456100
H	1.15254400	1.11360100	-1.55890600
C	4.24444500	-1.21958400	0.64510100
H	4.50346900	-0.43722400	1.35918700
H	4.23998200	-2.19074600	1.13367400
H	4.95658800	-1.21442300	-0.18066700
H	0.17217100	-0.47852500	0.90361600
C	2.68174200	0.12630000	-0.48434200
O	3.53436600	0.95882800	-0.67192500
O	2.91176800	-1.02448000	0.15516100

E(RM062X) = -831.480199228
Zero-point correction= 0.165369 (Hartree/Particle)
Thermal correction to Energy= 0.179545
Thermal correction to Enthalpy= 0.180489
Thermal correction to Gibbs Free Energy= 0.121999
Sum of electronic and zero-point Energies= -831.314830
Sum of electronic and thermal Energies= -831.300654
Sum of electronic and thermal Enthalpies= -831.299710
Sum of electronic and thermal Free Energies= -831.358200
Frequency: -508.7811

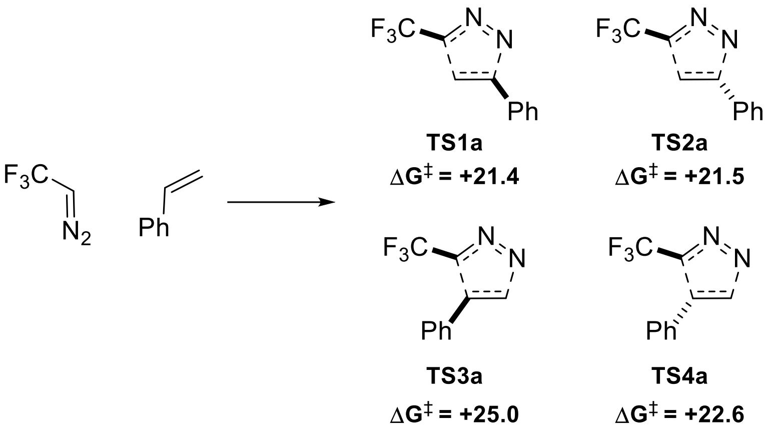

Precursors of TS1a

C	-0.29053900	0.84428200	-2.17938300
C	-2.44654900	0.89685800	0.57383700
N	-1.35247000	1.44111900	1.00826400
N	-0.40547600	1.92402000	1.36848600
C	0.74078800	1.27187100	-1.44953500
H	-0.52239900	-0.20898400	-2.29774300
H	-0.93728700	1.54897600	-2.69012400
H	-3.34808800	1.48779400	0.54775300
H	0.91237500	2.34211200	-1.35677800
C	-2.41737700	-0.53494400	0.22263600
F	-1.18090900	-1.04412100	0.30134800
F	-2.87190900	-0.76728700	-1.02530400
F	-3.20276400	-1.28781100	1.02504500
C	1.68687500	0.42323200	-0.69929300
C	2.54716500	1.02509000	0.22609200
C	1.75335600	-0.96632900	-0.86740300
C	3.44151300	0.26393000	0.97293900
H	2.50538900	2.10107900	0.36290200
C	2.64541900	-1.72697800	-0.12280000
H	1.10901900	-1.45586000	-1.58914200
C	3.49195700	-1.11592800	0.80218600
H	4.09780400	0.74893900	1.68665700
H	2.68538300	-2.80085700	-0.26667600
H	4.18802100	-1.71270700	1.38045400

E(RM062X) = -795.367156679
Zero-point correction= 0.173641 (Hartree/Particle)
Thermal correction to Energy= 0.188478
Thermal correction to Enthalpy= 0.189422
Thermal correction to Gibbs Free Energy= 0.128290
Sum of electronic and zero-point Energies= -795.193516
Sum of electronic and thermal Energies= -795.178679
Sum of electronic and thermal Enthalpies= -795.177735
Sum of electronic and thermal Free Energies= -795.238866

TS1a

C	0.50808900	1.08535900	1.47860100
C	2.06370200	0.91158200	-0.03508500
N	1.26441300	1.23696300	-1.06738200
N	0.21303900	1.57308000	-1.34642200
C	-0.64920800	1.42949000	0.80980600
H	0.64220100	0.07915900	1.86339400
H	1.05535000	1.85676100	2.00727900
H	2.82077300	1.65697500	0.19125400
H	-0.91906800	2.47939400	0.75309300
C	2.57157700	-0.49551400	-0.07125100
F	1.61210500	-1.36463000	-0.41391600
F	3.02576100	-0.83686000	1.14115400
F	3.58924100	-0.69003200	-0.93131100
C	-1.71242600	0.48456800	0.42593600
C	-2.94372300	0.98721500	-0.01914000
C	-1.54574600	-0.90731600	0.46707500
C	-3.97499500	0.13471000	-0.39663600
H	-3.08775300	2.06229400	-0.06478500
C	-2.57743300	-1.75968000	0.08938400
H	-0.60385500	-1.33201700	0.79417100
C	-3.79813300	-1.24585600	-0.34372400
H	-4.91863600	0.54933400	-0.73371300
H	-2.42554400	-2.83267100	0.13091800
H	-4.59982300	-1.91317800	-0.63812000

E(RM062X) = -795.338606740
Zero-point correction= 0.174916 (Hartree/Particle)
Thermal correction to Energy= 0.187852
Thermal correction to Enthalpy= 0.188796
Thermal correction to Gibbs Free Energy= 0.133812
Sum of electronic and zero-point Energies= -795.163691
Sum of electronic and thermal Energies= -795.150755
Sum of electronic and thermal Enthalpies= -795.149811
Sum of electronic and thermal Free Energies= -795.204795
Frequency: -506.0594

Precursors of TS2a

C	0.22330100	1.81649400	-1.83889100
C	1.10378000	-0.48606700	1.03412000
N	0.86895400	0.69870900	1.50318200
N	0.64930000	1.72140000	1.91072800
C	-0.80439300	1.86319900	-0.99071200
H	0.77238700	2.71608000	-2.09361500
H	0.55543400	0.89089400	-2.29806200
H	0.47943000	-1.30568100	1.35436100
C	2.18916900	-0.64070500	0.04843100
F	2.77705000	0.52985400	-0.23646400
F	1.75959500	-1.16826300	-1.11633800
F	3.16219300	-1.47637200	0.47128100
C	-1.60362700	0.70372900	-0.55011900
C	-2.32023600	0.78898800	0.64878700
C	-1.64214900	-0.49760900	-1.27024400
C	-3.02879700	-0.30572500	1.13608500
H	-2.30752600	1.71970600	1.20782300
C	-2.35368700	-1.58867100	-0.78763700
H	-1.11934500	-0.57561200	-2.21706300
C	-3.04352600	-1.50012700	0.42145100
H	-3.57202000	-0.22399900	2.07079900
H	-2.37586200	-2.51051000	-1.35799700
H	-3.59805600	-2.35311400	0.79562400
H	-1.07078000	2.81347400	-0.53318900

E(RM062X) = -795.368771915
Zero-point correction= 0.173387 (Hartree/Particle)
Thermal correction to Energy= 0.188299
Thermal correction to Enthalpy= 0.189243
Thermal correction to Gibbs Free Energy= 0.128466
Sum of electronic and zero-point Energies= -795.195385
Sum of electronic and thermal Energies= -795.180473
Sum of electronic and thermal Enthalpies= -795.179529
Sum of electronic and thermal Free Energies= -795.240306

TS2a

C	0.41934800	-0.09216800	1.36891100
C	1.61225200	0.54920500	-0.32673600
N	1.01420100	-0.26399200	-1.21651500
N	0.18321300	-1.03057700	-1.34959000
C	-0.60263000	-0.85770300	0.84573900
H	1.22545300	-0.59522200	1.89221100
H	0.24188900	0.93434200	1.67068100
H	1.43708400	1.60733200	-0.50082000
C	3.03525300	0.19026700	-0.03288800
F	3.19408100	-1.13110600	0.11913700
F	3.41923500	0.78758100	1.10189600
F	3.90687700	0.57417800	-0.98420000
C	-1.90896000	-0.31210800	0.44272200
C	-2.99207000	-1.17948700	0.24237100
C	-2.10977900	1.05793800	0.21621400
C	-4.23525900	-0.69648700	-0.15034700
H	-2.85052600	-2.24381200	0.40230900
C	-3.35312200	1.54040200	-0.17708100
H	-1.28802300	1.75455500	0.34529900
C	-4.42374400	0.66758900	-0.36092600
H	-5.05892100	-1.38715000	-0.29352400
H	-3.48599600	2.60342300	-0.34581700
H	-5.39158800	1.04597900	-0.66877000
H	-0.54074100	-1.93802000	0.92179800

E(RM062X) = -795.338910902
Zero-point correction= 0.174617 (Hartree/Particle)
Thermal correction to Energy= 0.187671
Thermal correction to Enthalpy= 0.188616
Thermal correction to Gibbs Free Energy= 0.132880
Sum of electronic and zero-point Energies= -795.164294
Sum of electronic and thermal Energies= -795.151240
Sum of electronic and thermal Enthalpies= -795.150295
Sum of electronic and thermal Free Energies= -795.206031
Frequency: -510.8147

Precursors of TS3a

C	-1.52154500	1.56187700	1.20391600
C	1.72055300	1.10166000	0.22709400
N	1.05067800	1.62254400	-0.75368000
N	0.45197200	2.06321500	-1.59405100
C	-1.77565000	2.75676000	0.66839600
H	-1.19354400	1.51305800	2.24010500
H	2.05279400	1.74016000	1.03012200
C	2.08745300	-0.32344600	0.12722400
F	1.38496100	-0.95381200	-0.82405200
F	1.88967900	-0.97167700	1.28830300
F	3.39383500	-0.52327400	-0.16931900
H	-2.11037600	2.88164000	-0.35611500
H	-1.65644400	3.65957300	1.25686300
C	-1.63324800	0.25286100	0.53123300
C	-1.35049500	-0.90539700	1.26491400
C	-2.00490500	0.11583900	-0.81388200
C	-1.43808700	-2.16479000	0.68014700
H	-1.05785500	-0.81175100	2.30607700
C	-2.08906100	-1.14116800	-1.39960700
H	-2.23266400	0.99354800	-1.40800800
C	-1.80684700	-2.28664700	-0.65579300
H	-1.21390700	-3.04850200	1.26685600
H	-2.37768900	-1.22911800	-2.44102700
H	-1.87413900	-3.26534600	-1.11698600

E(RM062X) = -795.368868368
Zero-point correction= 0.173303 (Hartree/Particle)
Thermal correction to Energy= 0.188185
Thermal correction to Enthalpy= 0.189130
Thermal correction to Gibbs Free Energy= 0.129212
Sum of electronic and zero-point Energies= -795.195566
Sum of electronic and thermal Energies= -795.180683
Sum of electronic and thermal Enthalpies= -795.179739
Sum of electronic and thermal Free Energies= -795.239656

TS3a

C	-0.07358300	1.50939300	-0.94894100
C	-1.78228200	0.03775600	-0.57958200
N	-2.37266100	0.85254200	0.31562000
N	-2.30034300	1.91433900	0.74056500
C	-0.58683200	2.63588300	-0.33317700
H	-0.24318400	1.37869700	-2.01254900
H	-2.30344800	-0.00720200	-1.53253200
C	-1.38787700	-1.29812000	-0.03456700
F	-0.89479600	-1.21245900	1.20632300
F	-0.45817700	-1.85595900	-0.81817700
F	-2.41046800	-2.17950500	0.02445300
H	-0.12783900	3.03556000	0.56324700
H	-1.20104400	3.32450600	-0.90265100
C	1.10194700	0.78627500	-0.40682800
C	1.95597700	0.10345400	-1.27899100
C	1.39100600	0.76634900	0.96259000
C	3.07425800	-0.57156700	-0.80089000
H	1.73659300	0.10216100	-2.34216700
C	2.50750100	0.09121500	1.44208300
H	0.72665100	1.26549400	1.66077700
C	3.35513600	-0.58024800	0.56287600
H	3.72575800	-1.09240600	-1.49369000
H	2.71315600	0.08246200	2.50669600
H	4.22384700	-1.10847200	0.93874700

E(RM062X) = -795.334006603
Zero-point correction= 0.174807 (Hartree/Particle)
Thermal correction to Energy= 0.187668
Thermal correction to Enthalpy= 0.188612
Thermal correction to Gibbs Free Energy= 0.134156
Sum of electronic and zero-point Energies= -795.159200
Sum of electronic and thermal Energies= -795.146339
Sum of electronic and thermal Enthalpies= -795.145395
Sum of electronic and thermal Free Energies= -795.199850
Frequency: -500.6984

Precursors TS4a

C	-0.45629800	2.02187000	-0.90719900
C	1.76790400	-0.29795800	0.97706900
N	2.53915300	0.69162900	0.64405100
N	3.18901000	1.56101000	0.35897600
C	0.33661300	2.83385200	-0.20757800
H	1.54138800	-0.45313000	2.01994600
C	1.40570500	-1.27067900	-0.07114600
F	1.35344200	-0.71145400	-1.29110600
F	0.21678800	-1.83021900	0.18664600
F	2.27650500	-2.30558800	-0.18254400
H	0.99490000	3.53199200	-0.71181700
H	0.36447600	2.83172800	0.87793200
C	-1.39687600	1.02504300	-0.35977900
C	-1.67376600	0.91402300	1.00989900
C	-2.03454600	0.14674200	-1.24204700
C	-2.55099800	-0.05519200	1.47764300
H	-1.20556300	1.59223400	1.71484800
C	-2.91555000	-0.82440200	-0.77482300
H	-1.82932900	0.22545400	-2.30509500
C	-3.17430900	-0.93075900	0.58761500
H	-2.75513200	-0.12765000	2.54011000
H	-3.39656200	-1.49803200	-1.47499000
H	-3.85920800	-1.68568000	0.95651200
H	-0.42000200	2.06471900	-1.99352600

E(RM062X) = -795.366567996
Zero-point correction= 0.173310 (Hartree/Particle)
Thermal correction to Energy= 0.188299
Thermal correction to Enthalpy= 0.189243
Thermal correction to Gibbs Free Energy= 0.128131
Sum of electronic and zero-point Energies= -795.193258
Sum of electronic and thermal Energies= -795.178269
Sum of electronic and thermal Enthalpies= -795.177325
Sum of electronic and thermal Free Energies= -795.238437

TS4a

C	0.15428400	0.57068900	1.09648500
C	-1.13784800	0.08073300	-0.67637600
N	-1.50845700	1.35784600	-0.85657600
N	-1.40515900	2.40359300	-0.40205800
C	-0.16319900	1.89530900	1.32263400
H	-0.50326500	-0.33724200	-1.45243300
C	-2.23792500	-0.80998500	-0.19683400
F	-2.95522300	-0.23125000	0.77689900
F	-1.71405800	-1.94108900	0.29194600
F	-3.11558200	-1.17375800	-1.15178400
H	-0.95357100	2.14868500	2.01978700
H	0.54263500	2.68392500	1.08865700
C	1.45228900	0.16139900	0.50511800
C	2.13235900	0.97691800	-0.40781100
C	2.01221900	-1.07439300	0.84337600
C	3.34780300	0.57405600	-0.94942600
H	1.70162200	1.92808200	-0.70522700
C	3.22944000	-1.47775100	0.30167200
H	1.48851000	-1.72006100	1.54154200
C	3.90370300	-0.65440400	-0.59565100
H	3.86045800	1.21794200	-1.65545500
H	3.65068200	-2.43695500	0.58140700
H	4.85055400	-0.96777600	-1.01996900
H	-0.33729000	-0.18723200	1.69946400

E(RM062X) = -795.335733448
Zero-point correction= 0.174625 (Hartree/Particle)
Thermal correction to Energy= 0.187590
Thermal correction to Enthalpy= 0.188534
Thermal correction to Gibbs Free Energy= 0.133264
Sum of electronic and zero-point Energies= -795.161108
Sum of electronic and thermal Energies= -795.148143
Sum of electronic and thermal Enthalpies= -795.147199
Sum of electronic and thermal Free Energies= -795.202469
Frequency: -512.0040

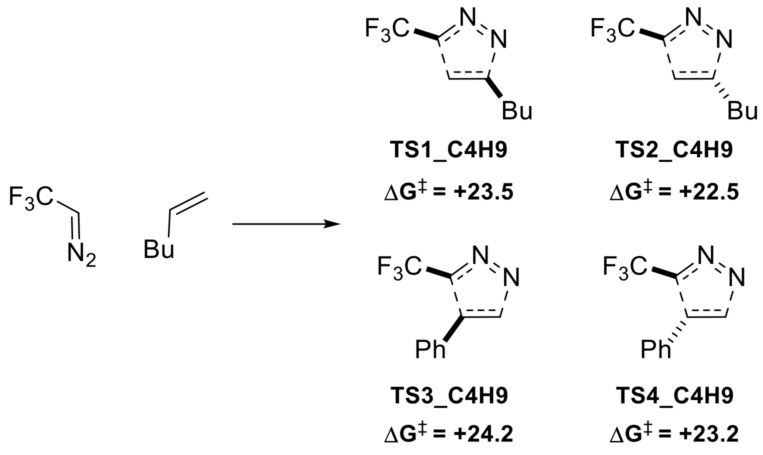

Precursors TS1_C4H9

C	0.15418100	-1.30440600	1.66994300
C	-1.63825700	0.91732200	-0.75009900
N	-0.98185100	1.49947700	0.20450600
N	-0.41084900	1.99613600	1.03302900
C	0.71578500	-1.23263900	0.46612100
H	0.45679300	-0.63798000	2.47393900
H	-0.62430100	-2.02724100	1.89119100
H	-1.38596600	1.14248500	-1.77391800
C	-2.69719800	-0.03758500	-0.37196300
F	-2.87693200	-0.08172500	0.95543800
F	-3.88700100	0.27602900	-0.92588800
F	-2.44499200	-1.29942300	-0.77843200
C	1.80145500	-0.27166800	0.08029100
H	1.43976500	0.37296200	-0.73364000
H	2.03086800	0.37651500	0.93063200
C	3.06485400	-0.99433400	-0.40437800
H	3.48882200	-1.56585000	0.42949100
H	2.78242300	-1.72204000	-1.17286700
C	4.12746800	-0.05063200	-0.97060300
H	4.96619700	-0.64810200	-1.34073500
H	3.71343300	0.47653500	-1.83764200
C	4.64417600	0.96528600	0.04660900
H	5.00883600	0.46137800	0.94712100
H	5.46926200	1.55026000	-0.36684200
H	3.86322600	1.66682100	0.34985900
H	0.38508100	-1.91703500	-0.31492800

E(RM062X) = -721.560863512
Zero-point correction= 0.205167 (Hartree/Particle)
Thermal correction to Energy= 0.221301
Thermal correction to Enthalpy= 0.222245
Thermal correction to Gibbs Free Energy= 0.157627
Sum of electronic and zero-point Energies= -721.355697
Sum of electronic and thermal Energies= -721.339562
Sum of electronic and thermal Enthalpies= -721.338618
Sum of electronic and thermal Free Energies= -721.403236

TS1_C4H9

C	-0.60926000	1.76742800	-0.63486500
C	-1.98985600	0.60003500	0.67192400
N	-1.01047800	0.18853200	1.49058600
N	0.11299400	0.26762700	1.68688900
C	0.64744500	1.52872900	-0.13156700
H	-0.93169500	1.25469500	-1.53756300
H	-1.11765600	2.69983000	-0.41714300
H	-2.70364300	1.28524700	1.11871500
H	1.09525500	2.27082200	0.52468700
C	-2.58919800	-0.49003300	-0.15594000
F	-1.65250900	-1.30367400	-0.66402500
F	-3.45617100	-1.27943800	0.50937400
F	-3.27034000	0.04461700	-1.17767000
C	1.58054900	0.50215100	-0.73554400
H	1.84763400	0.81364800	-1.75346300
H	1.05526500	-0.45540200	-0.84011000
C	2.86704700	0.30073400	0.06322500
H	2.62291700	-0.01452100	1.08327100
H	3.38775600	1.26238000	0.14963200
C	3.80490500	-0.72287200	-0.57210800
H	4.04447700	-0.40745500	-1.59370700
H	3.28439400	-1.68327400	-0.65679800
C	5.09394800	-0.90628200	0.22397300
H	4.87861400	-1.24681300	1.24091800
H	5.75349700	-1.64188700	-0.24270800
H	5.64328300	0.03674100	0.29824400

E(RM062X) = -721.529469165
Zero-point correction= 0.206438 (Hartree/Particle)
Thermal correction to Energy= 0.220576
Thermal correction to Enthalpy= 0.221520
Thermal correction to Gibbs Free Energy= 0.163651
Sum of electronic and zero-point Energies= -721.323032
Sum of electronic and thermal Energies= -721.308893
Sum of electronic and thermal Enthalpies= -721.307949
Sum of electronic and thermal Free Energies= -721.365818
Frequency: -513.4370

Precursors TS2_C4H9

C	0.21909800	-1.26904900	1.30164700
C	-1.84288200	0.71368000	-0.84154400
N	-1.22744300	1.53851000	-0.05288800
N	-0.68219100	2.23964500	0.63309000
C	1.12854000	-0.37921100	0.91228500
H	-0.08571500	-1.35531500	2.33955700
H	-0.25032700	-1.94261500	0.58925300
H	-1.65588500	0.76713600	-1.90227000
C	-2.82963600	-0.20668800	-0.24588900
F	-2.80696300	-0.16659200	1.09340600
F	-4.10019800	0.06545300	-0.62021500
F	-2.62260000	-1.48424900	-0.61881300
C	1.59454700	-0.21454100	-0.50605800
H	0.98282700	-0.84594900	-1.15765500
C	3.07898200	-0.55870800	-0.70182800
H	3.31642600	-0.47848400	-1.76843300
H	3.24469100	-1.60474100	-0.41930800
C	4.03434700	0.33679100	0.08576200
H	3.81674800	1.38576000	-0.14641800
H	3.86093500	0.21131300	1.15890400
C	5.49652900	0.03196600	-0.22862000
H	5.70878400	0.18969000	-1.28997200
H	6.17171300	0.66950900	0.34708800
H	5.73589600	-1.00888700	0.00778000
H	1.43952600	0.82687800	-0.81850000
H	1.57251600	0.28167600	1.65463000

E(RM062X) = -721.560279834
Zero-point correction= 0.205545 (Hartree/Particle)
Thermal correction to Energy= 0.221540
Thermal correction to Enthalpy= 0.222484
Thermal correction to Gibbs Free Energy= 0.158614
Sum of electronic and zero-point Energies= -721.354735
Sum of electronic and thermal Energies= -721.338740
Sum of electronic and thermal Enthalpies= -721.337795
Sum of electronic and thermal Free Energies= -721.401666

TS2_C4H9

C	0.10715000	-0.77136900	-0.32985000
C	-1.78796000	0.37187300	-0.68894100
N	-1.35557100	1.45621600	-0.02849900
N	-0.45964100	1.88261800	0.53913900
C	0.94295300	0.14076600	0.26994000
H	-0.37002400	-1.54626700	0.26109600
H	0.20707300	-0.98154400	-1.39099700
H	-1.93156700	0.51037200	-1.75607000
C	-2.87826000	-0.37557900	0.00707900
F	-2.65138000	-0.47881500	1.32404600
F	-4.10182800	0.17545000	-0.12535700
F	-2.96472500	-1.61390100	-0.49593500
C	1.96486300	0.92033600	-0.52322200
H	1.53223500	1.18077700	-1.49600600
C	3.27419100	0.14984000	-0.74747900
H	3.93252700	0.75011500	-1.38619100
H	3.05785400	-0.77492000	-1.29561000
C	4.01234600	-0.19051200	0.54575000
H	4.18669200	0.73174500	1.11195600
H	3.37965200	-0.82731200	1.17243300
C	5.34140700	-0.89401400	0.28456100
H	6.00541200	-0.26417200	-0.31457800
H	5.85858000	-1.13483100	1.21648800
H	5.18576600	-1.82827000	-0.26282400
H	2.19177900	1.86276300	-0.01335900
H	1.07177400	0.09705900	1.34737000

E(RM062X) = -721.529986447
Zero-point correction= 0.206787 (Hartree/Particle)
Thermal correction to Energy= 0.220792
Thermal correction to Enthalpy= 0.221737
Thermal correction to Gibbs Free Energy= 0.164202
Sum of electronic and zero-point Energies= -721.323200
Sum of electronic and thermal Energies= -721.309194
Sum of electronic and thermal Enthalpies= -721.308250
Sum of electronic and thermal Free Energies= -721.365785
Frequency: -505.7600

Precursors TS3_C4H9

C	3.00769300	0.86241100	-0.68350300
C	-0.85138700	1.17919500	-0.33262300
N	-0.54656900	1.71877000	0.80600900
N	-0.27233300	2.17626500	1.79446100
C	2.73309900	2.16131800	-0.77356200
H	3.67906200	0.41075700	-1.41349400
H	-0.31061500	1.48456400	-1.21518400
H	3.16537300	2.78244700	-1.55095200
C	-1.96799100	0.21477100	-0.36211000
F	-2.38226500	-0.10475700	0.87269300
F	-3.05033600	0.67628900	-1.02641100
F	-1.63257700	-0.92879000	-0.98633900
C	2.44006200	-0.06312500	0.35052500
H	1.75862100	0.49052800	1.00456300
H	3.24997400	-0.43913800	0.98739800
C	1.71576900	-1.26313400	-0.27134000
H	2.44949300	-1.91379400	-0.76151200
H	1.03146800	-0.91515100	-1.05333200
C	0.92713200	-2.06192800	0.76307100
H	0.13139200	-1.42438600	1.16789900
H	1.58303400	-2.31129900	1.60489700
C	0.32624100	-3.33940100	0.18434700
H	1.11394000	-4.01677000	-0.15790500
H	-0.27400100	-3.87172400	0.92624700
H	-0.31691800	-3.11652000	-0.67100300
H	2.07438200	2.64991900	-0.05922700

E(RM062X) = -721.560819215
Zero-point correction= 0.205522 (Hartree/Particle)
Thermal correction to Energy= 0.221402
Thermal correction to Enthalpy= 0.222346
Thermal correction to Gibbs Free Energy= 0.159304
Sum of electronic and zero-point Energies= -721.355297
Sum of electronic and thermal Energies= -721.339418
Sum of electronic and thermal Enthalpies= -721.338473
Sum of electronic and thermal Free Energies= -721.401515

TS3_C4H9

C	0.31034700	1.15272400	-0.71254700
C	-1.81024800	0.33690800	-0.61183500
N	-2.23946900	1.31735900	0.21024200
N	-1.90493700	2.30635800	0.67639200
C	0.14168400	2.38997400	-0.12999400
H	0.25060800	1.07940500	-1.79510400
H	-2.20110400	0.42677800	-1.62257300
H	-0.11030100	3.25943600	-0.72737800
C	-1.94061700	-1.03748700	-0.04112600
F	-1.43590900	-1.12360200	1.19778500
F	-3.21308100	-1.48204900	0.04129800
F	-1.28704900	-1.91214100	-0.81760000
C	1.13874900	0.09216800	-0.02130200
H	0.86349100	-0.90767000	-0.36631800
H	0.94780400	0.12703500	1.05676900
C	2.63696700	0.29682200	-0.27628100
H	2.93542000	1.28148100	0.10132200
H	2.82189400	0.30671300	-1.35743900
C	3.49722800	-0.78358300	0.37559100
H	3.19305500	-1.76458000	-0.00559000
H	3.30314600	-0.79432000	1.45385400
C	4.98763600	-0.57344600	0.12343000
H	5.31781400	0.39138800	0.51900400
H	5.58844700	-1.35305400	0.59795100
H	5.20718700	-0.58663600	-0.94805600
H	0.54726900	2.58364900	0.85816300

E(RM062X) = -721.527548589
Zero-point correction= 0.206688 (Hartree/Particle)
Thermal correction to Energy= 0.220696
Thermal correction to Enthalpy= 0.221640
Thermal correction to Gibbs Free Energy= 0.164666
Sum of electronic and zero-point Energies= -721.320861
Sum of electronic and thermal Energies= -721.306853
Sum of electronic and thermal Enthalpies= -721.305909
Sum of electronic and thermal Free Energies= -721.362882
Frequency: -491.0357

Precursors TS4_C4H9

C	0.96643700	1.01902100	0.64646400
C	-1.88615800	0.05422500	-1.00674000
N	-2.20760100	1.25302100	-0.63249600
N	-2.47534200	2.29318600	-0.30729900
C	0.40623900	2.15132500	1.06498000
H	-1.70945300	-0.13791200	-2.05297300
H	0.36452700	3.02976900	0.42583700
C	-1.81967800	-0.99766600	0.02467400
F	-1.99226300	-0.50201000	1.25784600
F	-2.75567400	-1.95871700	-0.14278200
F	-0.64233500	-1.65329800	0.01274900
H	-0.03078500	2.23941700	2.05458800
C	1.60337200	0.83290900	-0.70116700
H	1.20252200	-0.07533600	-1.16417500
H	1.33118100	1.67213400	-1.34871300
C	3.13646400	0.72852900	-0.62831200
H	3.51909400	0.55023600	-1.63941400
H	3.53754500	1.69321400	-0.30055200
C	3.65144900	-0.37281100	0.30266300
H	4.74445100	-0.38479300	0.25270500
H	3.39466100	-0.12958400	1.33894900
C	3.11285400	-1.75920100	-0.04433400
H	3.30790500	-2.00197000	-1.09379800
H	3.58435700	-2.52935900	0.57075900
H	2.03360500	-1.82323600	0.11698300
H	0.98756800	0.16220100	1.31868000

E(RM062X) = -721.560120604
Zero-point correction= 0.205658 (Hartree/Particle)
Thermal correction to Energy= 0.221535
Thermal correction to Enthalpy= 0.222479
Thermal correction to Gibbs Free Energy= 0.158963
Sum of electronic and zero-point Energies= -721.354463
Sum of electronic and thermal Energies= -721.338586
Sum of electronic and thermal Enthalpies= -721.337641
Sum of electronic and thermal Free Energies= -721.401157

TS4_C4H9

C	0.63698500	0.74759300	0.28177300
C	-1.28051200	0.25541400	-0.73454800
N	-1.86162200	1.43381100	-0.45244500
N	-1.68936000	2.42982000	0.07925200
C	0.38921400	1.98353900	0.83404100
H	-1.07839400	0.08508700	-1.78825000
H	0.77424800	2.88256200	0.36180500
C	-1.88065200	-0.90549100	-0.01162300
F	-2.09304600	-0.63005500	1.28333600
F	-3.06150700	-1.32592400	-0.50830600
F	-1.04560500	-1.95127100	-0.08056400
H	0.05309400	2.08076100	1.86009500
C	1.54743800	0.59320500	-0.91969700
H	1.34232900	-0.35398200	-1.42680300
H	1.32909700	1.39034100	-1.63827200
C	3.03454400	0.65571400	-0.53956500
H	3.63099800	0.49660200	-1.44528400
H	3.26507700	1.66327800	-0.17804300
C	3.45238900	-0.36621600	0.52162200
H	4.52893500	-0.27010000	0.69208000
H	2.96769300	-0.12413200	1.47329200
C	3.12695200	-1.80805800	0.13630500
H	3.54730400	-2.05537000	-0.84370800
H	3.53812200	-2.51168600	0.86409000
H	2.04778700	-1.97815000	0.08786700
H	0.55386000	-0.11958800	0.93345400

E(RM062X) = -721.529339936
Zero-point correction= 0.207013 (Hartree/Particle)
Thermal correction to Energy= 0.220801
Thermal correction to Enthalpy= 0.221745
Thermal correction to Gibbs Free Energy= 0.165196
Sum of electronic and zero-point Energies= -721.322327
Sum of electronic and thermal Energies= -721.308539
Sum of electronic and thermal Enthalpies= -721.307595
Sum of electronic and thermal Free Energies= -721.364144
Frequency: -507.8400

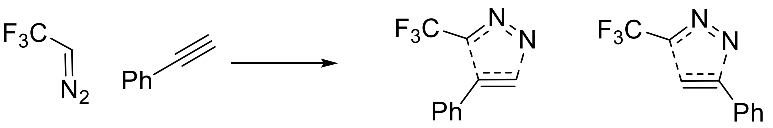

Precursors TS1_Acetylene

C	-1.32352200	-0.34943300	-1.14001900
N	-0.81056200	0.76697300	-1.55764500
N	-0.34803300	1.72587200	-1.91322500
H	-1.28179600	-1.21552600	-1.78118800
C	-2.07008600	-0.32748300	0.13178900
F	-1.75738200	0.74183900	0.87900000
F	-3.41499300	-0.29202100	-0.03029900
F	-1.83392300	-1.42866200	0.86386300
C	1.48076300	1.86735200	0.85749500
C	1.30377300	3.01810100	1.16229500
C	1.67035800	0.49577200	0.47923900
C	1.25822300	-0.53384900	1.33365000
C	2.24047500	0.18244700	-0.76197600
C	1.41219800	-1.85986100	0.94702600
H	0.81284000	-0.28671600	2.29038800
C	2.38254100	-1.14530600	-1.14367900
H	2.55746300	0.98350000	-1.41973700
C	1.96847200	-2.16799600	-0.29133800
H	1.08679900	-2.65277100	1.61036500
H	2.82037700	-1.38314300	-2.10629200
H	2.08126400	-3.20312200	-0.59270500
H	1.15072000	4.03899100	1.43528100

E(RM062X) = -794.123656231
Zero-point correction= 0.149626 (Hartree/Particle)
Thermal correction to Energy= 0.164220
Thermal correction to Enthalpy= 0.165164
Thermal correction to Gibbs Free Energy= 0.105725
Sum of electronic and zero-point Energies= -793.974030
Sum of electronic and thermal Energies= -793.959436
Sum of electronic and thermal Enthalpies= -793.958492
Sum of electronic and thermal Free Energies= -794.017931

TS1_Acetylene

C	-1.46372700	0.03051500	-0.79103900
N	-2.51927500	0.82601500	-0.57927500
N	-2.81893200	1.83863100	-0.14740900
H	-1.19202600	-0.11317300	-1.83315000
C	-1.45921500	-1.20807400	0.05176100
F	-1.69960300	-0.93361300	1.34105100
F	-2.38006200	-2.12009900	-0.31943900
F	-0.26892100	-1.80772700	-0.03070700
C	-0.07201100	1.51862500	0.13353500
C	-0.80554100	2.45507300	0.46725400
C	1.20064500	0.82435200	0.03340300
C	1.87385100	0.44567000	1.19971800
C	1.76069200	0.52271600	-1.21269600
C	3.08884900	-0.22635100	1.11664500
H	1.43567200	0.67358200	2.16468600
C	2.98131600	-0.13684600	-1.28835900
H	1.23640900	0.80933100	-2.11763000
C	3.64678400	-0.51825300	-0.12500300
H	3.60058200	-0.52117300	2.02568900
H	3.41163800	-0.35851400	-2.25832000
H	4.59419200	-1.04090000	-0.18702000
H	-1.09160900	3.40671500	0.86308300

E(RM062X) = -794.085178753
Zero-point correction= 0.150184 (Hartree/Particle)
Thermal correction to Energy= 0.163175
Thermal correction to Enthalpy= 0.164119
Thermal correction to Gibbs Free Energy= 0.108649
Sum of electronic and zero-point Energies= -793.934995
Sum of electronic and thermal Energies= -793.922004
Sum of electronic and thermal Enthalpies= -793.921060
Sum of electronic and thermal Free Energies= -793.976529
Frequency: -512.2848

Precursors TS2_Acetylene

C	-2.45913100	-0.02614700	-1.18515700
N	-1.26803600	0.31010800	-1.57351000
N	-0.23652300	0.59249100	-1.91360500
H	-3.13937600	-0.45907800	-1.90099100
C	-2.82715700	0.22594400	0.22028500
F	-1.84404000	0.84325700	0.89107400
F	-3.92900400	0.99724700	0.33079600
F	-3.12227400	-0.90191800	0.90011100
C	-0.27103500	-2.19950500	0.33448000
H	-1.12742300	-2.83623100	0.36822600
C	0.70323500	-1.49283700	0.30522800
C	1.85764200	-0.64037500	0.26605600
C	3.08247000	-1.13217700	-0.20122500
C	1.75846200	0.69325200	0.68294500
C	4.19232300	-0.29729600	-0.24806400
H	3.15478400	-2.16400300	-0.52436800
C	2.87337100	1.52061900	0.63284300
H	0.80858800	1.07020000	1.04374100
C	4.09062100	1.02825800	0.16744100
H	5.13831700	-0.68263000	-0.61022200
H	2.79152100	2.55147400	0.95717700
H	4.95855600	1.67637100	0.12941700

E(RM062X) = -794.122144598
Zero-point correction= 0.149587 (Hartree/Particle)
Thermal correction to Energy= 0.164315
Thermal correction to Enthalpy= 0.165259
Thermal correction to Gibbs Free Energy= 0.103723
Sum of electronic and zero-point Energies= -793.972558
Sum of electronic and thermal Energies= -793.957830
Sum of electronic and thermal Enthalpies= -793.956885
Sum of electronic and thermal Free Energies= -794.018421

TS2_Acetylene

C	2.13245800	0.68657200	0.59805500
N	1.33987400	1.42821100	-0.18341300
N	0.26884700	1.57818900	-0.53311600
H	2.54838200	1.20024900	1.45944700
C	3.07691800	-0.20170600	-0.15132700
F	2.50160900	-0.73803500	-1.23368500
F	4.19264100	0.42022500	-0.57542200
F	3.47588100	-1.20073000	0.64535200
C	0.49233200	-0.56744200	1.26811700
H	1.07006000	-1.17200200	1.93894800
C	-0.55385100	-0.19694100	0.72708400
C	-1.93891800	-0.15745300	0.32847700
C	-2.68008900	1.02949100	0.39996300
C	-2.56369300	-1.32633500	-0.12861400
C	-4.02228600	1.03966200	0.04134800
H	-2.19630700	1.93888400	0.73775700
C	-3.90231700	-1.30426400	-0.50258500
H	-1.99202400	-2.24526000	-0.18770500
C	-4.63713800	-0.12448600	-0.41487400
H	-4.58769600	1.96203400	0.10907000
H	-4.37329100	-2.21331000	-0.85890200
H	-5.68185300	-0.11111800	-0.70297700

E(RM062X) = -794.088226867
Zero-point correction= 0.150256 (Hartree/Particle)
Thermal correction to Energy= 0.163282
Thermal correction to Enthalpy= 0.164226
Thermal correction to Gibbs Free Energy= 0.108431
Sum of electronic and zero-point Energies= -793.937971
Sum of electronic and thermal Energies= -793.924945
Sum of electronic and thermal Enthalpies= -793.924001
Sum of electronic and thermal Free Energies= -793.979796
Frequency: -505.4780

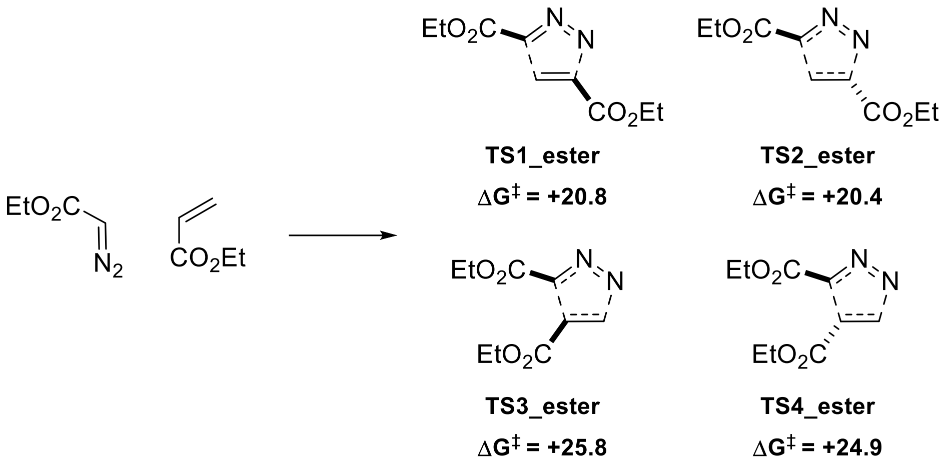

Precursors TS1_ester

C	-0.07000200	-1.70308900	0.79437000
C	1.41997200	1.28460700	0.75586000
N	0.19548800	1.53386300	0.37252800
N	-0.85349400	1.71933800	0.03121100
C	-1.30808300	-1.29053500	1.04635300
H	0.28868700	-1.78918900	-0.22686200
H	0.61039700	-1.96378800	1.59726500
H	1.70893100	1.60274100	1.74469100
H	-1.69366300	-1.18210500	2.05364200
C	2.25620500	0.58837600	-0.20403400
O	1.91443500	0.26265700	-1.32071600
O	3.46649300	0.34959300	0.31179800
C	4.39767900	-0.34216200	-0.54633200
H	3.96851000	-1.30839700	-0.81864000
H	4.53587300	0.24467800	-1.45637900
C	5.68630700	-0.49637000	0.22420200
H	5.52682000	-1.07688600	1.13483400
H	6.41771100	-1.01905200	-0.39536000
H	6.09586900	0.47891500	0.49393700
C	-2.21925500	-0.91702000	-0.06655500
O	-1.98324900	-1.06297100	-1.24232300
O	-3.35128300	-0.38106300	0.39721100
C	-4.28856900	0.08854900	-0.59165200
H	-4.58165700	-0.75284100	-1.22268400
H	-3.78649400	0.83066200	-1.21640700
C	-5.46698300	0.67705900	0.14557000
H	-6.19849600	1.04625900	-0.57597900
H	-5.94901400	-0.07654500	0.77132800
H	-5.15222500	1.51092700	0.77599100

E(RM062X) = -761.649953953
Zero-point correction= 0.230935 (Hartree/Particle)
Thermal correction to Energy= 0.249206
Thermal correction to Enthalpy= 0.250150
Thermal correction to Gibbs Free Energy= 0.180636
Sum of electronic and zero-point Energies= -761.419019
Sum of electronic and thermal Energies= -761.400748
Sum of electronic and thermal Enthalpies= -761.399804
Sum of electronic and thermal Free Energies= -761.469318

TS1_ester

C	0.17827000	0.15219600	1.51796800
C	1.28686000	1.35917700	0.25269200
N	0.24671100	1.88132600	-0.43748000
N	-0.87951300	1.92678600	-0.53016900
C	-1.16209600	0.41189600	1.28906200
H	0.58019900	-0.78641200	1.14705600
H	0.64380000	0.54129300	2.41592600
H	1.77229900	2.08141600	0.90064400
H	-1.71337500	1.12953600	1.88287000
C	2.14631900	0.45178100	-0.55152900
O	1.89513400	0.06928200	-1.66577400
O	3.19407300	0.07488500	0.17228600
C	4.07638200	-0.89786300	-0.43716700
H	3.49015700	-1.78497500	-0.68299800
H	4.47040000	-0.47098200	-1.36104800
C	5.16846700	-1.19793200	0.55898500
H	4.75153700	-1.61248400	1.47861100
H	5.85457400	-1.93042400	0.12943800
H	5.73316200	-0.29546100	0.80012600
C	-1.92808400	-0.44679900	0.38775800
O	-1.46686500	-1.31914300	-0.32143200
O	-3.24193300	-0.15794300	0.41445500
C	-4.08365300	-0.92698200	-0.46105900
H	-3.99046700	-1.98422300	-0.20325100
H	-3.73890600	-0.79409100	-1.48882700
C	-5.49855600	-0.43105700	-0.27834700
H	-6.16993600	-0.99529800	-0.92893600
H	-5.82587500	-0.56473500	0.75469400
H	-5.57468600	0.62690100	-0.53721700

E(RM062X) = -761.622613191
Zero-point correction= 0.232208 (Hartree/Particle)
Thermal correction to Energy= 0.248610
Thermal correction to Enthalpy= 0.249555
Thermal correction to Gibbs Free Energy= 0.186442
Sum of electronic and zero-point Energies= -761.390405
Sum of electronic and thermal Energies= -761.374003
Sum of electronic and thermal Enthalpies= -761.373059
Sum of electronic and thermal Free Energies= -761.436171
Frequency: -539.4429

Precursors TS2_ester

C	-0.09686000	-1.71122500	1.06560300
C	0.69294900	1.10929100	-0.61650500
N	-0.32289300	0.80558300	-1.37966500
N	-1.17929100	0.51484800	-2.03833500
C	-1.18896400	-2.06002900	0.39162800
H	0.74844800	-2.38708700	1.13716400
H	-0.02205700	-0.75369500	1.56897700
H	0.57836700	1.93597400	0.06610500
C	1.84055300	0.22717300	-0.71699900
C	-2.38348600	-1.19176900	0.25506700
H	-1.27972600	-3.02095400	-0.10185900
O	1.94432300	-0.69833000	-1.49333400
O	2.77767000	0.57137700	0.17308100
C	3.97553800	-0.23248600	0.17757100
H	4.43206800	-0.18425200	-0.81280800
H	3.69891300	-1.26885400	0.38142800
C	4.88575500	0.32640400	1.24400300
H	5.80719500	-0.25835900	1.27576700
H	5.14274900	1.36519900	1.02840000
H	4.41029000	0.27625000	2.22539900
O	-3.41593400	-1.55963800	-0.25532700
O	-2.20229700	0.03963900	0.74135300
C	-3.29095500	0.96718900	0.56398000
H	-4.12979100	0.64401100	1.18413700
H	-3.60465400	0.93995500	-0.48186300
C	-2.78501200	2.33282200	0.96138200
H	-3.59273200	3.06134300	0.86746300
H	-2.43657200	2.33346800	1.99600800
H	-1.96441700	2.64538400	0.31112900

E(RM062X) = -761.651125129
Zero-point correction= 0.231230 (Hartree/Particle)
Thermal correction to Energy= 0.249318
Thermal correction to Enthalpy= 0.250262
Thermal correction to Gibbs Free Energy= 0.182159
Sum of electronic and zero-point Energies= -761.419896
Sum of electronic and thermal Energies= -761.401807
Sum of electronic and thermal Enthalpies= -761.400863
Sum of electronic and thermal Free Energies= -761.468966

TS2_ester

C	0.07858100	-0.02224200	-1.11788800
C	-0.80954600	0.04502800	0.74801900
N	-0.03431500	-0.89447400	1.33628200
N	0.80522700	-1.63644300	1.17891300
C	1.07279300	-0.98833900	-1.08515100
H	-0.82475600	-0.24486600	-1.67715800
H	0.35997400	1.02401100	-1.08821600
H	-0.65248900	1.04658600	1.13323700
C	-2.20315800	-0.41675000	0.51296400
C	2.46613100	-0.67510500	-0.77512700
H	0.90246000	-1.98374900	-1.47461000
O	-2.56522500	-1.56417900	0.58582600
O	-2.95855500	0.60455600	0.12990100
C	-4.31731600	0.28688000	-0.25754500
H	-4.81463900	-0.18192900	0.59314500
H	-4.27970700	-0.42956200	-1.08013400
C	-4.98134800	1.57951600	-0.66114000
H	-6.01152100	1.37559100	-0.95934700
H	-4.99597600	2.28475800	0.17181800
H	-4.46108400	2.03687100	-1.50462900
O	3.40734300	-1.41711500	-0.97018000
O	2.61299100	0.54170800	-0.21247300
C	3.95465300	0.93414800	0.12425600
H	4.56386400	0.92349500	-0.78207200
H	4.37018300	0.20655200	0.82461100
C	3.88245400	2.31524300	0.73119000
H	4.88689200	2.64808800	1.00072000
H	3.46127400	3.02956800	0.02085200
H	3.26724900	2.31098500	1.63334800

E(RM062X) = -761.622438166
Zero-point correction= 0.232263 (Hartree/Particle)
Thermal correction to Energy= 0.248704
Thermal correction to Enthalpy= 0.249649
Thermal correction to Gibbs Free Energy= 0.186042
Sum of electronic and zero-point Energies= -761.390175
Sum of electronic and thermal Energies= -761.373734
Sum of electronic and thermal Enthalpies= -761.372790
Sum of electronic and thermal Free Energies= -761.436396
Frequency: -546.0967

Precursors TS3_ester

C	-1.97122000	0.85460000	-1.42726200
C	-0.34277300	-1.67373100	-0.24065100
N	-1.54864900	-1.89470300	0.22947900
N	-2.57436400	-2.06042500	0.63456800
C	-3.24155600	0.46807200	-1.48185300
H	-1.33809900	0.91171100	-2.30558300
H	-0.23261600	-1.76347500	-1.30855500
C	0.64062700	-1.29804400	0.76074900
H	-3.84757100	0.43080200	-0.58203600
O	0.39675900	-1.26270900	1.94860900
O	1.87002000	-0.97839400	0.33337500
C	2.18397600	-0.96277700	-1.07328700
H	1.61559300	-0.15720900	-1.54717000
H	1.90255600	-1.91965600	-1.51867900
C	3.67409700	-0.75110400	-1.20155200
H	3.94355700	-0.72097100	-2.25921400
H	3.98296100	0.18691900	-0.73805200
H	4.21694300	-1.57147400	-0.72854000
C	-1.35829700	1.23455200	-0.12688200
O	-1.91865000	1.18780600	0.94205600
O	-0.09644100	1.64699600	-0.28263800
C	0.58455700	2.09576400	0.90995300
H	0.62932800	1.26756900	1.61960200
H	0.00044400	2.90335000	1.35595900
C	1.95802800	2.55877300	0.49195500
H	2.51077800	2.89491100	1.37150800
H	2.51281900	1.74120100	0.02907900
H	1.89169400	3.38866900	-0.21446700
H	-3.70544400	0.18731300	-2.42041500

E(RM062X) = -761.647094271
Zero-point correction= 0.231617 (Hartree/Particle)
Thermal correction to Energy= 0.249368
Thermal correction to Enthalpy= 0.250312
Thermal correction to Gibbs Free Energy= 0.184996
Sum of electronic and zero-point Energies= -761.415478
Sum of electronic and thermal Energies= -761.397726
Sum of electronic and thermal Enthalpies= -761.396782
Sum of electronic and thermal Free Energies= -761.462099

TS3_ester

C	-1.14886700	1.24678800	-0.88048200
C	-1.41088000	-0.99614300	-0.57563400
N	-2.72722600	-0.82428100	-0.36585400
N	-3.52007200	0.00355800	-0.29860800
C	-2.44059700	1.69249300	-0.65003400
H	-0.77771700	1.13341900	-1.89143400
H	-1.17214800	-1.41005500	-1.54834400
C	-0.67604600	-1.40906600	0.65129400
H	-2.67751600	2.15064500	0.30412400
O	-1.19790300	-1.44773700	1.73620100
O	0.62664900	-1.66416900	0.52198700
C	1.24578400	-1.80676800	-0.77819500
H	1.16163500	-0.86243100	-1.31988800
H	0.73050800	-2.59683700	-1.32922400
C	2.68939600	-2.17318000	-0.53032200
H	3.21408600	-2.25027700	-1.48474200
H	3.18053600	-1.41125300	0.07767400
H	2.75858700	-3.13282500	-0.01509500
C	-0.11993100	1.43514800	0.16524200
O	-0.33637700	1.64162200	1.33636500
O	1.11482800	1.34778800	-0.35124100
C	2.21737300	1.43309000	0.57226400
H	2.19790600	0.54468300	1.20925100
H	2.08554000	2.31382800	1.20256100
C	3.48152200	1.51920300	-0.24988000
H	4.34895500	1.52778300	0.41335500
H	3.56951900	0.66406900	-0.92332400
H	3.49179800	2.43475600	-0.84468600
H	-3.06467900	1.97013700	-1.49101200

E(RM062X) = -761.611402716
Zero-point correction= 0.233348 (Hartree/Particle)
Thermal correction to Energy= 0.248963
Thermal correction to Enthalpy= 0.249907
Thermal correction to Gibbs Free Energy= 0.190424
Sum of electronic and zero-point Energies= -761.378055
Sum of electronic and thermal Energies= -761.362440
Sum of electronic and thermal Enthalpies= -761.361496
Sum of electronic and thermal Free Energies= -761.420978
Frequency: -526.3545

Precursors TS4_ester

C	-1.01779000	-1.16664900	1.41544300
C	1.10600300	-0.69245700	-1.04069700
N	0.69339500	-1.92068500	-1.25005100
N	0.33043700	-2.96486400	-1.39703300
C	-1.17160100	-2.48647500	1.44054700
H	0.72909200	0.05515700	-1.71906500
C	2.00396600	-0.54140400	0.09247600
H	-0.71642100	-3.09015800	2.21727900
O	2.40455200	-1.47682500	0.75220400
O	2.38900500	0.70204800	0.40936500
C	1.92591900	1.83428100	-0.35303900
H	0.83356900	1.85115500	-0.33601600
H	2.27302500	1.73484900	-1.38494700
C	2.49596100	3.07195600	0.29654100
H	2.16872200	3.95510900	-0.25572500
H	2.14993100	3.15820900	1.32811100
H	3.58699800	3.04301800	0.29010400
H	-1.76179700	-2.99103200	0.68219600
C	-1.62943400	-0.36060700	0.32960500
O	-2.22432200	-0.81044400	-0.62188100
O	-1.42925900	0.94769200	0.52997300
C	-1.98446500	1.85364100	-0.44548100
H	-1.41886200	2.77538000	-0.31202900
H	-1.79575300	1.45605200	-1.44410200
C	-3.46240800	2.07928500	-0.20348700
H	-3.83735600	2.81536600	-0.91802800
H	-4.02668400	1.15462300	-0.33159800
H	-3.62868200	2.46268800	0.80523100
H	-0.43932200	-0.63075600	2.15943900

E(RM062X) = -761.645558967
Zero-point correction= 0.231474 (Hartree/Particle)
Thermal correction to Energy= 0.249420
Thermal correction to Enthalpy= 0.250364
Thermal correction to Gibbs Free Energy= 0.182685
Sum of electronic and zero-point Energies= -761.414085
Sum of electronic and thermal Energies= -761.396139
Sum of electronic and thermal Enthalpies= -761.395195
Sum of electronic and thermal Free Energies= -761.462874

TS4_ester

C	-0.23557400	-1.17972800	0.83384300
C	1.27364200	-0.49970900	-0.70777100
N	1.64882400	-1.76954000	-0.92645300
N	1.47221300	-2.83242000	-0.52978000
C	0.04447300	-2.53588200	0.89821600
H	0.74917900	-0.03583100	-1.53508000
C	2.25367900	0.24476500	0.13231100
H	0.64918000	-2.91782400	1.71223500
O	3.14628000	-0.31411300	0.72037300
O	2.06795600	1.55393200	0.28247600
C	1.03376500	2.25942100	-0.44480400
H	0.06717700	1.80505700	-0.21824500
H	1.24014300	2.17832800	-1.51474400
C	1.07705000	3.69668500	0.01244800
H	0.31209400	4.26626900	-0.51897300
H	0.88106400	3.76553200	1.08380300
H	2.05138500	4.13996900	-0.20015200
H	-0.63606700	-3.23520900	0.42595700
C	-1.41335200	-0.74839300	0.04903800
O	-1.93680700	-1.39814000	-0.82739600
O	-1.83202300	0.47200500	0.41402700
C	-2.97694700	1.01184300	-0.27810800
H	-2.89693000	2.08881800	-0.13307700
H	-2.88713400	0.78474200	-1.34109500
C	-4.26742400	0.47277300	0.30189600
H	-5.11565200	0.94573700	-0.19805200
H	-4.33978400	-0.60625900	0.15811500
H	-4.32872200	0.69550800	1.36908900
H	0.10769200	-0.50124100	1.60559300

E(RM062X) = -761.610250201
Zero-point correction= 0.232675 (Hartree/Particle)
Thermal correction to Energy= 0.248857
Thermal correction to Enthalpy= 0.249801
Thermal correction to Gibbs Free Energy= 0.186980
Sum of electronic and zero-point Energies= -761.377575
Sum of electronic and thermal Energies= -761.361393
Sum of electronic and thermal Enthalpies= -761.360449
Sum of electronic and thermal Free Energies= -761.423271
Frequency: -541.4501

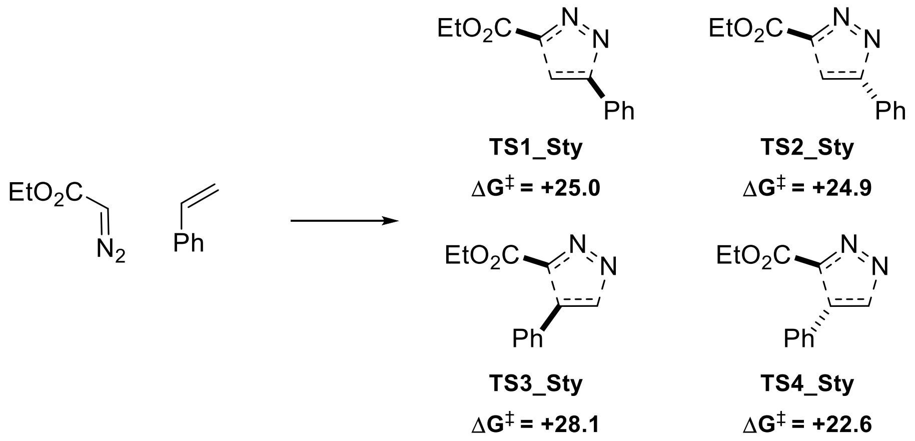

Precursors TS1_Sty

C	0.20587300	1.06238300	-1.72724300
C	-1.72682800	1.36354900	1.00532600
N	-0.48755700	1.41576600	1.42571300
N	0.57469800	1.44513200	1.77132600
C	1.47153600	1.28355400	-1.36871100
H	-0.28054500	0.10371900	-1.57497400
H	-0.37826000	1.84107600	-2.20492700
H	-2.30362900	2.27357400	1.04072400
H	1.91633300	2.25418300	-1.57747300
C	-2.18658900	0.07064200	0.54432500
O	-1.52480100	-0.94806800	0.54522600
O	-3.44927500	0.14172400	0.11152700
C	-4.02758100	-1.08971600	-0.36897100
H	-3.42169600	-1.45913700	-1.19857100
H	-3.99848800	-1.82592100	0.43656500
C	-5.44134100	-0.78209300	-0.79876900
H	-5.45059000	-0.03893200	-1.59833200
H	-5.91321700	-1.69432300	-1.16912200
H	-6.02855200	-0.40544700	0.04085700
C	2.37068600	0.30907900	-0.72017000
C	3.74749200	0.55689700	-0.70961300
C	1.89348700	-0.85487600	-0.10130700
C	4.63117000	-0.34242400	-0.11966100
H	4.12601500	1.46211700	-1.17378300
C	2.77571800	-1.75135500	0.48879300
H	0.82636100	-1.04823700	-0.05978400
C	4.14800700	-1.50159800	0.47930700
H	5.69538900	-0.13543500	-0.12584200
H	2.39111100	-2.64483000	0.96790000
H	4.83260500	-2.20218500	0.94362900

E(RM062X) = -725.500120897
Zero-point correction= 0.240714 (Hartree/Particle)
Thermal correction to Energy= 0.257737
Thermal correction to Enthalpy= 0.258681
Thermal correction to Gibbs Free Energy= 0.192814
Sum of electronic and zero-point Energies= -725.259407
Sum of electronic and thermal Energies= -725.242384
Sum of electronic and thermal Enthalpies= -725.241440
Sum of electronic and thermal Free Energies= -725.307306

TS1_Sty

C	0.10233800	-1.97608400	1.21603200
C	-1.54577100	-1.75544400	-0.12369800
N	-0.79733500	-1.71520000	-1.25020200
N	0.28508400	-1.72515500	-1.60923600
C	1.24419700	-1.77333500	0.45874300
H	-0.25177500	-1.20900200	1.89690300
H	-0.15311700	-2.98959000	1.50359500
H	-2.13287300	-2.65875000	-0.00665100
H	1.81557600	-2.63766700	0.13759100
C	1.92112600	-0.47257700	0.32064300
C	3.17847300	-0.41336400	-0.29774900
C	1.34672700	0.73043400	0.75812400
C	3.84278300	0.79661900	-0.46439800
H	3.63502700	-1.33316900	-0.65009300
C	2.01077100	1.94042200	0.59101700
H	0.37162600	0.72688500	1.23308600
C	3.26243600	1.98243100	-0.02046900
H	4.81553400	0.81359000	-0.94339400
H	1.54690900	2.85693500	0.93934900
H	3.77723000	2.92735100	-0.15020300
C	-2.19547000	-0.48340200	0.26723600
O	-1.78716900	0.54626400	-0.47048800
O	-2.94514700	-0.40941600	1.21059300
C	-2.25005700	1.86052300	-0.08426100
H	-1.51196500	2.53736500	-0.51204900
H	-2.22141400	1.93581900	1.00335800
C	-3.63530500	2.12975800	-0.63098400
H	-4.36658500	1.44292200	-0.20227700
H	-3.92993600	3.15046100	-0.37737800
H	-3.64344700	2.02867100	-1.71802100

E(RM062X) = -725.465888684
Zero-point correction= 0.242221 (Hartree/Particle)
Thermal correction to Energy= 0.257223
Thermal correction to Enthalpy= 0.258167
Thermal correction to Gibbs Free Energy= 0.198380
Sum of electronic and zero-point Energies= -725.223668
Sum of electronic and thermal Energies= -725.208666
Sum of electronic and thermal Enthalpies= -725.207721
Sum of electronic and thermal Free Energies= -725.267508
Frequency: -552.8928

Precursors TS2_Sty

C	0.48947500	-2.27119100	0.86647200
C	0.74779900	1.31643000	1.03798000
N	-0.30131600	1.90253900	0.51780200
N	-1.18783000	2.39440900	0.04801000
C	-0.26027100	-1.93991500	-0.18502500
H	1.38517100	-2.86894900	0.73910400
H	0.25181900	-1.95813900	1.87822100
H	0.85129700	1.33465200	2.11075500
C	1.63847900	0.67540300	0.09259300
O	1.48918700	0.67352900	-1.11130600
O	2.65568200	0.08376900	0.73196800
C	3.59635000	-0.64043200	-0.08508800
H	4.05404700	-1.35374900	0.59944700
H	3.04780400	-1.18489500	-0.85495000
C	4.63092400	0.28806500	-0.68589800
H	5.15580700	0.83536100	0.09987300
H	5.36369800	-0.29830600	-1.24509000
H	4.16633100	1.00157500	-1.36800000
H	0.05238100	-2.26600100	-1.17449700
C	-1.49631700	-1.13369100	-0.16281700
C	-2.05064600	-0.71503100	-1.37749700
C	-2.14933200	-0.77638200	1.02575900
C	-3.22353500	0.03440800	-1.41120100
H	-1.55274700	-0.98397400	-2.30380700
C	-3.31728400	-0.02569300	0.99337500
H	-1.75061700	-1.09827700	1.98154300
C	-3.86176100	0.38052900	-0.22538200
H	-3.63565800	0.34759200	-2.36380300
H	-3.81188100	0.23792300	1.92173900
H	-4.77567200	0.96315800	-0.24613700

E(RM062X) = -725.502199671
Zero-point correction= 0.240816 (Hartree/Particle)
Thermal correction to Energy= 0.257663
Thermal correction to Enthalpy= 0.258607
Thermal correction to Gibbs Free Energy= 0.194111
Sum of electronic and zero-point Energies= -725.261383
Sum of electronic and thermal Energies= -725.244536
Sum of electronic and thermal Enthalpies= -725.243592
Sum of electronic and thermal Free Energies= -725.308089

TS2_Sty

C	-0.03019600	0.17044400	-1.19778100
C	-1.14017000	0.23398700	0.62436400
N	-0.42314000	-0.72311600	1.25352700
N	0.46116700	-1.42735400	1.10649100
C	1.05857600	-0.65116500	-0.95539000
H	-0.83474200	-0.21558800	-1.81521100
H	0.09035200	1.24816900	-1.22431600
H	-1.01578100	1.22808900	1.03904100
C	2.36785100	-0.15276000	-0.50375600
C	3.50543300	-0.96063400	-0.64053600
C	2.52022600	1.10441900	0.10058600
C	4.75287800	-0.52279100	-0.20962000
H	3.40322400	-1.94112100	-1.09488800
C	3.76794000	1.54182000	0.53112100
H	1.65616100	1.74570000	0.24123500
C	4.89216300	0.73290600	0.37713900
H	5.61866000	-1.16429800	-0.33167600
H	3.86258800	2.51764800	0.99472900
H	5.86336800	1.07566700	0.71469800
H	1.04293100	-1.66526400	-1.34016200
C	-2.49357600	-0.20377800	0.21423500
O	-2.87826200	-1.34766500	0.21912300
O	-3.18368300	0.83740200	-0.24608600
C	-4.50383600	0.56637100	-0.77308400
H	-4.71007700	1.41418300	-1.42405800
H	-4.46164400	-0.34450000	-1.37067600
C	-5.52031100	0.46208900	0.34307300
H	-6.51348700	0.31754600	-0.08786700
H	-5.30155400	-0.38441000	0.99564200
H	-5.53249200	1.37812300	0.93698000

E(RM062X) = -725.465674967
Zero-point correction= 0.241884 (Hartree/Particle)
Thermal correction to Energy= 0.257059
Thermal correction to Enthalpy= 0.258003
Thermal correction to Gibbs Free Energy= 0.197291
Sum of electronic and zero-point Energies= -725.223791
- Sum of electronic and thermal Energies= -725.208616
Sum of electronic and thermal Enthalpies= -725.207672
Sum of electronic and thermal Free Energies= -725.268384
Frequency: -556.3401

Precursors TS3_Sty

C	0.78853000	1.83618800	-1.04637700
C	1.64017500	-0.82694300	0.90069200
N	2.78360800	-0.18668500	0.91855100
N	3.75953700	0.35519000	0.90186600
C	1.76072200	2.52676000	-0.45017800
H	0.95692200	1.45760600	-2.05186600
H	1.07372900	-0.81598200	1.81686900
C	1.29756900	-1.40879500	-0.38706600
H	1.66164300	2.93468400	0.55109600
O	2.02822200	-1.35353900	-1.35446700
O	0.12438200	-2.04630900	-0.48417700
C	-0.79895400	-2.05833400	0.62220000
H	-1.04743400	-1.02751900	0.88800900
H	-0.32765300	-2.55168600	1.47645400
C	-2.02389800	-2.81697800	0.17203900
H	-2.76680600	-2.81379900	0.97264700
H	-2.46225800	-2.34887900	-0.71088900
H	-1.77152100	-3.85243200	-0.06475200
H	2.70093000	2.70493700	-0.95974200
C	-0.53808100	1.51763500	-0.48130600
C	-1.46737700	0.84682200	-1.28391700
C	-0.91198300	1.86180700	0.82588800
C	-2.74084700	0.54507600	-0.80779300
H	-1.18522100	0.56382300	-2.29319600
C	-2.17947200	1.55568100	1.30410500
H	-0.20859400	2.37292500	1.47402200
C	-3.10208300	0.89885100	0.48797800
H	-3.44822100	0.03240700	-1.44989800
H	-2.45222100	1.83139900	2.31670400
H	-4.09196300	0.66513300	0.86318700

E(RM062X) = -725.497552251
Zero-point correction= 0.241153 (Hartree/Particle)
Thermal correction to Energy= 0.257908
Thermal correction to Enthalpy= 0.258852
Thermal correction to Gibbs Free Energy= 0.194914
Sum of electronic and zero-point Energies= -725.256399
Sum of electronic and thermal Energies= -725.239644
Sum of electronic and thermal Enthalpies= -725.238700
Sum of electronic and thermal Free Energies= -725.302639

TS3_Sty

C	-0.94217700	0.80530900	-1.33974300
C	-1.69581800	-0.97668700	-0.12182300
N	-2.82897100	-0.32918200	0.22526200
N	-3.36500500	0.66840100	0.03597900
C	-2.06278200	1.62209000	-1.26971600
H	-0.82621500	0.17139000	-2.21362800
H	-1.85462000	-1.75787300	-0.85623500
C	-0.75318300	-1.16151500	1.00354500
O	-0.94041700	-0.69471100	2.10142000
O	0.38062600	-1.81483100	0.74227100
C	0.60158200	-2.43551900	-0.54163700
H	-0.09897200	-3.26733700	-0.65320400
H	0.42461900	-1.70590200	-1.33457500
C	2.03249200	-2.91424700	-0.56091000
H	2.20738300	-3.63734400	0.23768300
H	2.24201300	-3.39532300	-1.51842900
H	2.71674400	-2.07317000	-0.43298500
H	-2.03325400	2.54003400	-0.69344000
C	0.31672400	1.13587600	-0.63359700
C	1.54581600	0.77360400	-1.19705600
C	0.31725800	1.80441700	0.59706700
C	2.74261200	1.07844400	-0.55642500
H	1.56019600	0.25568300	-2.15146400
C	1.51272700	2.10779400	1.23787900
H	-0.62545000	2.07071000	1.06411400
C	2.73073900	1.74650800	0.66497200
H	3.68457600	0.79382200	-1.01220700
H	1.49364100	2.61962300	2.19354000
H	3.66138300	1.98107500	1.16877100
H	-2.79182200	1.58996300	-2.07148800

E(RM062X) = -725.457923370
Zero-point correction= 0.242043 (Hartree/Particle)
Thermal correction to Energy= 0.256847
Thermal correction to Enthalpy= 0.257792
Thermal correction to Gibbs Free Energy= 0.199990
Sum of electronic and zero-point Energies= -725.215881
Sum of electronic and thermal Energies= -725.201076
Sum of electronic and thermal Enthalpies= -725.200132
Sum of electronic and thermal Free Energies= -725.257934
Frequency: -540.3281

Precursors TS4_Sty

C	-2.63275000	0.19570000	-1.30460900
C	0.61871500	1.14806200	1.11176500
N	-0.59176800	1.58917100	1.35941000
N	-1.62027600	1.97038900	1.56608300
C	-3.70840800	0.78499100	-0.78156000
H	1.10989000	0.63907900	1.92435900
H	-4.29813900	1.47679600	-1.37220500
H	-4.03531900	0.61024100	0.23834400
C	-1.73337000	-0.75347800	-0.62143500
C	-1.98405100	-1.24602000	0.66681300
C	-0.56881400	-1.16769000	-1.27829400
C	-1.08687300	-2.11079100	1.28108400
H	-2.88543600	-0.95231500	1.19354600
C	0.32970600	-2.03556500	-0.66573000
H	-0.36255600	-0.78921900	-2.27428700
C	0.07565800	-2.50734600	0.61898700
H	-1.29571300	-2.48204800	2.27829000
H	1.22921700	-2.33843400	-1.19067800
H	0.77307200	-3.18353900	1.10080400
H	-2.35231100	0.43112000	-2.32857700
C	1.07510100	1.35995200	-0.25087600
O	0.43758600	1.97640500	-1.07853800
O	2.26741800	0.84760800	-0.58739200
C	3.05133100	0.11470200	0.37304300
H	2.46316100	-0.72685600	0.74827300
H	3.30674600	0.77729700	1.20442400
C	4.29285300	-0.36619000	-0.33895500
H	4.91689100	-0.92839000	0.35855200
H	4.02949700	-1.01857800	-1.17363600
H	4.87069600	0.47787200	-0.71961300

E(RM062X) = -725.497027856
Zero-point correction= 0.240878 (Hartree/Particle)
Thermal correction to Energy= 0.257692
Thermal correction to Enthalpy= 0.258636
Thermal correction to Gibbs Free Energy= 0.194969
Sum of electronic and zero-point Energies= -725.256150
Sum of electronic and thermal Energies= -725.239336
Sum of electronic and thermal Enthalpies= -725.238391
Sum of electronic and thermal Free Energies= -725.302059

TS4_Sty

C	0.51215700	0.77205500	1.04354500
C	-0.70309200	0.93631000	-0.82733600
N	-0.52576500	2.26630300	-0.91386300
N	-0.07801100	3.14022300	-0.31892000
C	0.68615800	2.10943800	1.36549600
H	-0.20121400	0.34186000	-1.58136900
H	-0.00586200	2.58751900	2.04932100
H	1.65980000	2.57653700	1.27103800
C	1.61602200	-0.06039800	0.50966400
C	2.64748400	0.49203300	-0.26067200
C	1.63207800	-1.43700000	0.75725900
C	3.67351300	-0.30819900	-0.74994800
H	2.64087300	1.55370900	-0.48745400
C	2.65948700	-2.23833500	0.26757200
H	0.83209400	-1.87869400	1.34326200
C	3.68626100	-1.67720400	-0.48641800
H	4.46382800	0.13650400	-1.34450800
H	2.65698400	-3.30248000	0.47579100
H	4.48665900	-2.29954700	-0.86959300
H	-0.27419100	0.22095600	1.55176000
C	-2.03646400	0.53743100	-0.33908600
O	-2.84356300	1.28620400	0.15847800
O	-2.18564500	-0.78206000	-0.44202800
C	-3.39133900	-1.33616300	0.13035800
H	-4.25006300	-0.88636700	-0.37129000
H	-3.42612900	-1.06811300	1.18812100
C	-3.33714200	-2.83033200	-0.07212800
H	-4.23490200	-3.28447000	0.35175000
H	-3.29379500	-3.07639100	-1.13470700
H	-2.46472000	-3.25768400	0.42592000

E(RM062X) = -725.463450864
Zero-point correction= 0.241782 (Hartree/Particle)
Thermal correction to Energy= 0.256965
Thermal correction to Enthalpy= 0.257909
Thermal correction to Gibbs Free Energy= 0.197364
Sum of electronic and zero-point Energies= -725.221669
Sum of electronic and thermal Energies= -725.206486
Sum of electronic and thermal Enthalpies= -725.205542
Sum of electronic and thermal Free Energies= -725.266087
Frequency: -548.3
